# Multiple Lineages of Ancient CR1 Retroposons Shaped the Early Genome Evolution of Amniotes

**DOI:** 10.1093/gbe/evu256

**Published:** 2014-12-11

**Authors:** Alexander Suh, Gennady Churakov, Meganathan P. Ramakodi, Roy N. Platt, Jerzy Jurka, Kenji K. Kojima, Juan Caballero, Arian F. Smit, Kent A. Vliet, Federico G. Hoffmann, Jürgen Brosius, Richard E. Green, Edward L. Braun, David A. Ray, Jürgen Schmitz

**Affiliations:** ^1^Institute of Experimental Pathology (ZMBE), University of Münster, Germany; ^2^Department of Evolutionary Biology (EBC), Uppsala University, Sweden; ^3^Department of Biochemistry, Molecular Biology, Entomology and Plant Pathology, Mississippi State University; ^4^Institute for Genomics, Biocomputing and Biotechnology, Mississippi State University; ^5^Department of Biological Sciences, Texas Tech University; ^6^Genetic Information Research Institute, Mountain View, California; ^7^Institute for Systems Biology, Seattle, Washington; ^8^Department of Biology, University of Florida; ^9^Department of Biomolecular Engineering, University of California; ^10^Department of Biology and Genetics Institute, University of Florida; ^11^Present address: Cancer Prevention and Control Program, Fox Chase Cancer Center, Philadelphia, PA; ^12^Present address: Department of Biology, Temple University, Philadelphia, PA

**Keywords:** transposable elements, chicken repeat 1, phylogenomics, comparative genomics, crocodilian genomes, amniotes

## Abstract

Chicken repeat 1 (CR1) retroposons are long interspersed elements (LINEs) that are ubiquitous within amniote genomes and constitute the most abundant family of transposed elements in birds, crocodilians, turtles, and snakes. They are also present in mammalian genomes, where they reside as numerous relics of ancient retroposition events. Yet, despite their relevance for understanding amniote genome evolution, the diversity and evolution of CR1 elements has never been studied on an amniote-wide level. We reconstruct the temporal and quantitative activity of CR1 subfamilies via presence/absence analyses across crocodilian phylogeny and comparative analyses of 12 crocodilian genomes, revealing relative genomic stasis of retroposition during genome evolution of extant Crocodylia. Our large-scale phylogenetic analysis of amniote CR1 subfamilies suggests the presence of at least seven ancient CR1 lineages in the amniote ancestor; and amniote-wide analyses of CR1 successions and quantities reveal differential retention (presence of ancient relics or recent activity) of these CR1 lineages across amniote genome evolution. Interestingly, birds and lepidosaurs retained the fewest ancient CR1 lineages among amniotes and also exhibit smaller genome sizes. Our study is the first to analyze CR1 evolution in a genome-wide and amniote-wide context and the data strongly suggest that the ancestral amniote genome contained myriad CR1 elements from multiple ancient lineages, and remnants of these are still detectable in the relatively stable genomes of crocodilians and turtles. Early mammalian genome evolution was thus characterized by a drastic shift from CR1 prevalence to dominance and hyperactivity of L2 LINEs in monotremes and L1 LINEs in therians.

## Introduction

More than three decades ago, chicken repeat 1 (CR1) elements were the first transposable elements (TEs) to be identified in a genome of a nonmammalian land vertebrate (Stumph et al. 1981, 1984). CR1 elements are a family of long interspersed elements (LINEs) (Burch et al. 1993) that mobilize via an RNA intermediate and retropose via target-primed reverse transcription (Haas et al. 1997) similar to other LINEs (Luan et al. 1993). Full-length elements contain two open reading frames (ORFs) that encode the Gag-like ORF1p protein with a zinc finger-like motif (Haas et al. 1997, 2001; Kajikawa et al. 1997; Kapitonov and Jurka 2003), as well as the Pol-like ORF2p protein with endonuclease and reverse transcriptase (RT) domains (Burch et al. 1993; Haas et al. 1997, 2001; Kajikawa et al. 1997). The vast majority of CR1 insertions are heavily 5′-truncated elements (Vandergon and Reitman 1994; Hillier et al. 2004; Wicker et al. 2005) and thus functionally “dead on arrival” (Petrov and Hartl 1998), which hampers the reconstruction of full-length CR1 subfamilies and the confident determination of their exact 5′-UTR sequences (Kajikawa et al. 1997; Haas et al. 2001; Wicker et al. 2005). The extent of 5′-truncations appears to be more severe than in L1 LINEs and implies a lower processivity of CR1 reverse transcription (Hillier et al. 2004). On the other hand, the 3′-UTR of CR1 elements has been suggested to serve as a recognition site for the CR1-encoded RT (Kajikawa et al. 1997; Haas et al. 2001) and its hairpin structure and octamer microsatellite motif are highly conserved across various amniote CR1 subfamilies (Suh, Paus, et al. 2011).

CR1 retroposons are a ubiquitous genomic component that is present in all lineages of amniotes (Shedlock 2006; Suh, Paus et al. 2011), including mammals (Lovšin et al. 2001; Kapitonov and Jurka 2003; Shedlock 2006; Suh, Paus, et al. 2011) (but contra [Kordiš 2009]), and appear to constitute a Metazoa-specific, ancient clade of LINEs (Lovšin et al. 2001). In birds, many studies have shown that multiple lineages of CR1 elements were active in parallel, and some of them throughout long timespans of avian evolution (Hillier et al. 2004; Wicker et al. 2005; Kriegs et al. 2007; Abrusán et al. 2008; Liu et al. 2009; Suh, Paus, et al. 2011; Suh et al. 2012). On the other hand, the total CR1 element sequence from nonavian amniotes was only under 12 million basepair (Mb) of genomic data (Shedlock 2006; Shedlock et al. 2007), neglecting mammalian CR1 subfamilies. This paucity of comparative analyses is surprising, given that CR1 retroelements are “the major genome component” (Kordiš 2009) in birds (Hillier et al. 2004; Warren et al. 2010), crocodilians (Green et al. 2014), snakes (Castoe et al. 2013) and other lepidosaurs (Shedlock 2006), and turtles (Shaffer et al. 2013), and are thus crucial for understanding the genome evolution within Amniota.

Here, we present the first genome-wide study of CR1 retroelements across all major lineages of amniotes. First, we inferred the presence or absence of CR1 insertions across the crocodilian phylogeny. These data provided another important line of evidence that was able to resolve crocodilian phylogeny, a classic example of conflict between phylogenetic reconstructions using molecular versus morphological data (e.g., [Harshman et al. 2003; Janke et al. 2005]). Then we used whole-genome data to conduct a de novo characterization of crocodilian CR1 subfamilies in three genomes and complemented the whole-genome data by survey sequencing eight additional crocodilian species. Finally, phylogenetic reconstruction of the relationships among amniote CR1 lineages permitted us to infer the CR1 diversity in the ancestral amniote genome and reconstruct the subsequent events of CR1 expansion or inactivation that led to the pronounced differences among the repetitive landscapes of extant amniote genomes.

## Materials and Methods

### TE Subfamily Prediction

As part of the collaborative efforts to annotate crocodilian genomes (St John et al. 2012; Green et al. 2014), consensus sequences of CR1 and other TE subfamilies from American alligator (*Alligator mississippiensis*), saltwater crocodile (*Crocodylus porosus*), and gharial (*Gavialis gangeticus*) were generated in the laboratories of DAR, JJ, and AFS. All subfamilies were predicted de novo using complementing methods implemented in RepeatModeler (http://www.repeatmasker.org/RepeatModeler.html last accessed January 13, 2015), followed by procedures that are described in detail elsewhere (Dasmahapatra et al. 2012; Green et al. 2014). Briefly, RepeatModeler was initially used to analyze the *A. mississippiensis* genome draft, after which manual work was necessary to confirm or extend the consensus sequences for each repeat by first querying the entire *A. mississippiensis* draft using BLAST (version 2.2.23 [Altschul et al. 1990]). Up to 50 of the top hits for each putative consensus were extracted along with up to 1,000 bp of flanking sequence. The extracted sequences were aligned with their respective RepeatModeler-generated partner using MUSCLE (version 4.0 [Edgar 2004]) and a majority-rule consensus sequence was created. To be considered “complete”, a consensus sequence must exhibit highly variable flanking sequences at the 5′- and 3′-termini of the putative consensus, indicating insertion of an element at multiple distinct loci. If this condition was not met, the process was repeated by extending the flanking sequences. RepeatModeler analysis of *Cr. porosus* was followed by comparison to the resulting *A. mississippiensis* library to identify elements predicted from both genomes. Unique putative repeats from *C. porosus* were used to query the *C. porosus* assembly and the BLAST/extract/align process was repeated. Finally, the process was repeated once more for *G. gangeticus* elements. After combining all three crocodilian repeat libraries, UCLUST (Edgar 2010) was used to group consensus sequences of potential subfamilies at 95% sequence identity thresholds. Consensus sequences with more than 95% identity were merged into a single subfamily consensus, whereas the remaining were defined as consensus sequences of separate subfamilies and named according to the UCLUST groupings.

### Presence/Absence Analyses

We extracted a total of approximately 12 million retroposon loci from the genome assemblies of saltwater crocodile, gharial, and American alligator, and selected approximately 30,000 cases with TE-free flanking sequences of 750 bp. These loci were BLASTn screened against a set of 26,637 short introns (<1.5 kb) from anole lizard and 28,713 short introns from chicken, yielding 122 TE candidate loci in alligator, 112 loci in crocodile, and 56 loci in gharial. Three-way alignments of the three crocodilians were compiled for all these loci in order not to bias the outcome of our experimental screening toward one of the two competing hypothesis regarding the position of the gharial. After we found no conflict among those three-way presence/absence patterns, we generated oligonucleotide primers (supplementary table S2, Supplementary Material online) for a total of 73 retroposon loci for subsequent experimental presence/absence screening.

We experimentally screened our marker candidate loci using standard procedures previously used in avian retroposed element (RE) presence/absence screenings (Suh, Paus, et al. 2011) across a taxon sampling comprising all crocodilian genera and the key species within the *Crocodylus* radiation sensu Oaks (2011). Briefly, we amplified all samples via touchdown polymerase chain reaction (PCR), followed by PCR product purification and direct sequencing (Suh, Paus, et al. 2011). All sequences were aligned per locus using MAFFT (Katoh and Toh 2008) (E-INS-I, version 6, http://mafft.cbrc.jp/alignment/server/index.html last accessed January 13, 2015), manually realigned, and presence/absence states carefully scored following the strict criteria of Suh, Paus, et al. (2011). That is, orthology of a phylogenetically informative retroposon insertion requires identity of RE target site, RE orientation, RE subtype, and (if present) target site duplication, as well as a clear absence (empty RE insertion site) in other species. In total, this was the case for 36 RE insertion loci (supplementary table S1, Supplementary Material online) that are all available as supplementary material (supplementary data S1, Supplementary Material online). As part of analyzing these loci, we also noted five non-TE indels that were phylogenetically informative and supplemented our phylogeny.

### Transposition in Transposition Analyses

We estimated chronologies of CR1 activity probabilities using the transposition in transposition (TinT) method (Kriegs et al. 2007; Churakov et al. 2010) (http://www.compgen.uni-muenster.de/tools/tint/ last accessed January 13, 2015; default parameters for CR1 elements) on 6,752 nested CR1 in Chinese alligator, 8,816 nested CR1 in American alligator, 9,100 nested CR1 in saltwater crocodile, and 8,628 nested CR1 in gharial. The resultant graphs contain successions of probable retroposon activity periods on a relative timescale, where ovals represent 75%, vertical lines 95%, and horizontal lines 99% of the normal distribution.

### Survey Sequencing

Our survey sequencing sampling comprises *Alligator sinensis*, *Caiman latirostris*, *Caiman yacare*, *Crocodylus acutus*, *Crocodylus niloticus*, *Mecistops cataphractus*, *Melanosuchus niger*, *Osteolaemus tetraspis*, and *Tomistoma schlegelii*. We isolated DNA from blood and generated standard TruSeq Illumina libraries with insert sizes of 263 bp and bar codes for each taxon. All nine libraries were sequenced as 100-bp reads on a single lane of an Illumina GAIIx genetic analyzer and yielded an average of approximately 0.2× coverage per genome. We then applied the strategy from Diez et al. (2014) and conducted BLASTn searches of the resultant unassembled reads against a library of all crocodilian CR1 subfamilies. After selecting hits longer than 29 bp, cumulative quantities of CR1-derived reads were calculated for each CR1 subfamily and survey sequencing library. We then compared the CR1 representation in the Chinese alligator survey sequences with the recently published genome assembly (Wan et al. 2013) and, for each CR1 subfamily, derived coefficients for conversion of the remaining eight survey sequencing libraries into genome-wide estimates of CR1 quantities. This was possible because CR1 TinT patterns (fig. 2*A*) and CR1 landscapes (fig. 4) from the four genome assemblies (together spanning the breadth of crocodilian diversity) suggest similar age distributions (and quantities) for most CR1 subfamilies, which implies that old, diverged CR1 elements are probably equally abundant in the survey sequenced species due to subfamily activity in the last common ancestor of Crocodylia. Consequently, the detection of not only young (i.e., elements with near-identical sequence and thus high detectability), but also these older CR1 fragments is probably equivalent across the survey sequenced species. The resultant CR1 quantities (fig. 2*B*) support this assumption, as many CR1 subfamilies have nearly equal amounts of masked bases in all 12 sampled crocodilians.

### Phylogenetic Analyses

We compiled a sampling of 119 CR1 subfamilies from various amniote genomes in RepBase (Jurka et al. 2005) (http://www.girinst.org/repbase/index.html last accessed January 13, 2015), including the crocodilian CR1 consensus sequences generated for this study. As many of these subfamily predictions feature 5′-truncations, we focused on analyzing the 3′-part of the ORF2 RT domain together with the adjacent 3′-UTR. Note that we only used *Xenopus* CR1 subfamilies as our outgroup, because nontetrapod CR1 elements (e.g., from zebra fish) could not be aligned unambiguously at the nucleotide level. We also did not sample L2 LINEs, as they are closely related to nontetrapod CR1-like elements (Kapitonov and Jurka 2003) and likewise problematic for unambiguous alignment to amniote CR1 elements. All sampled sequences were first aligned using MAFFT (E-INS-I, version 6), manually realigned, and then ambiguously aligned sites at the boundary of ORF2 and 3′-UTR were excluded from the alignment. We then conducted maximum likelihood-based sequence analysis using RAxML (8.0.0 [Stamatakis et al. 2008], GTRCAT model, 1,000 bootstrap inferences) on the CIPRES Science Gateway (Miller et al. 2010) (https://www.phylo.org/portal2/login!input.action last accessed January 13, 2015). The full alignment is available as supplementary material (supplementary data S2, Supplementary Material online).

### Landscape Analyses

We created per-taxon custom CR1 libraries and used these for masking genomes of amniote representatives in a local installation of RepeatMasker (Smit et al. 1996-2014). Distances from the consensus sequence were calculated using the Kimura 2-parameter model (Kimura 1980) in the calcDivergenceFromAlign.pl script that is part of the RepeatMasker program package (Smit et al. 1996-2014). This Perl script removes hypermutable CpG sites during the calculation of Kimura 2-parameter distances between sequence pairs and converts the RepeatMasker “.align” output file (containing per-sequence pair transition–transversion ratios) into a table file (Pagán et al. 2010). We then generated CR1 landscape plots using the total base pairs of CR1-annotated sequence per CR1 group in bins of size 1% in the range of 0–50% divergence.

## Results and Discussion

### CR1 Markers Resolve the Early Branches of the Crocodilian Tree of Life

**Fig. 1.—:**
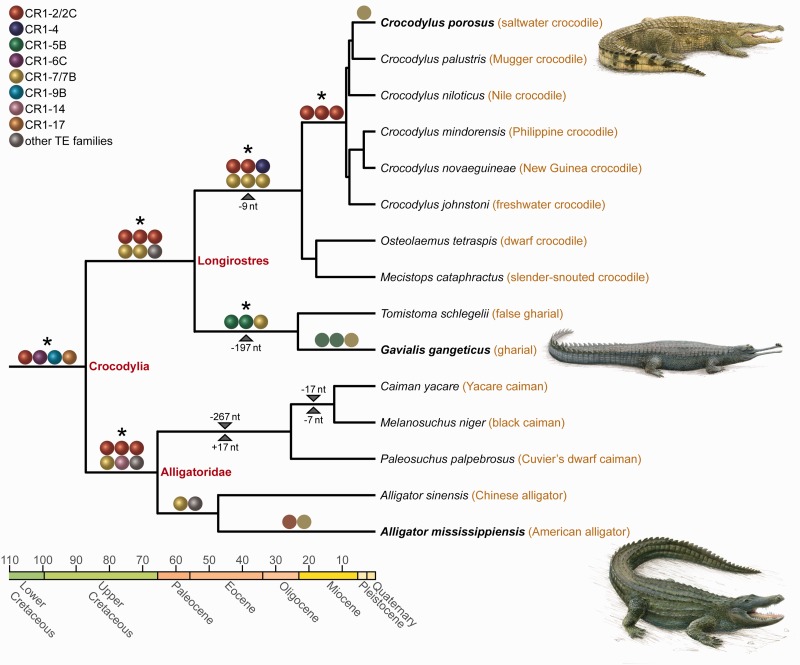
CR1 retroposons resolve the early branches in the crocodilian tree of life. Retroposon markers (colored balls) and non-TE indel markers (triangles with number of inserted/deleted nucleotides) are mapped on Oaks’s (Oaks 2011) dated tree of crocodilians (colors on the time axis correspond to the respective geological epoch in the International Stratigraphic Chart; http://www.stratigraphy.org/ICSchart/StratChart2010.pdf last accessed January 13, 2015). Presence/absence states (supplementary table S1, Supplementary Material online) are based on per-locus alignments (supplementary data S1, Supplementary Material online) and species-specific autapomorphic retroposon insertions are depicted as colored circles. Statistically significant retroposon support sensu Waddell et al. (2001) is highlighted with asterisks. Since we did not observe any conflict, this corresponds to those branches with at least three insertions. Species names in bold letters are the three genome assemblies that were used as starting points for CR1 presence/absence screenings; names in red letters refer to higher-ranking taxa. Note that despite the fact that crocodilians diverged from their avian outgroup more than 219 Ma (Shedlock and Edwards 2009), making it difficult to align neutrally evolving DNA, we identified four CR1 markers for crocodilian monophyly. These could be aligned with chicken and zebra finch sequences, where they exhibit an orthologous insertion site without the CR1 insertion.

To reconstruct the temporal impact of CR1 LINEs on crocodilian genomes, we first studied CR1 presence/absence patterns among different crocodilian species. In addition to providing direct evidence for the timing of a TE insertion event, presence/absence patterns of CR1 and other REs constitute powerful, nearly homoplasy-free phylogenetic markers (Shedlock et al. 2004; Ray et al. 2006; Han et al. 2011). As they have been successfully used to resolve long-standing phylogenetic controversies among avian relationships (Kaiser et al. 2007; Kriegs et al. 2007; Suh, Kriegs, et al. 2011; Suh, Paus, et al. 2011; Haddrath and Baker 2012; Liu et al. 2012; Suh et al. 2012; Baker et al. 2014; Jarvis et al. 2014), they promised to be equally valuable markers for reconstructing the phylogeny of crocodilians, the extant sister taxon of birds. Using the genome assemblies of saltwater crocodile (*C. porosus*), gharials (*G. gangeticus*), and American alligator (*A. mississippiensis*) as starting points for retroposon marker search (see Materials and Methods), we experimentally tested 73 loci via high-throughput PCR on a set of taxa comprising all extant crocodilian genera and the most distantly related species within *Crocodylus* (Oaks 2011). We sequenced all PCR amplicons of the sampled taxa and obtained 32 RE presence/absence patterns that span crocodilian phylogeny (fig. 1 and supplementary table S1, Supplementary Material online) and constitute the hitherto first RE presence/absence analysis in this amniote taxon. Most of these insertions (29 of 32; supplementary table S1, Supplementary Material online) correspond to CR1 insertions, although there were also two insertions of Penelope LINEs and one of an endogenous retrovirus. We also identified four CR1 markers that constitute unequivocal insertion events in the common ancestor of Crocodylia, as we could determine that the insertion was absent (i.e., empty insertion site) in the avian outgroup. Notably, all RE markers are fully congruent with each other and also conflict-free when compared with the sequence-based multilocus phylogeny of Oaks (2011). This suggests that, in sharp contrast to the situation for the early divergences of neoavian birds (Suh, Paus, et al. 2011; Jarvis et al. 2014) and placental mammals (Churakov et al. 2009; Nishihara et al. 2009), incomplete lineage sorting has not confounded the inference of crocodilian phylogeny.

We obtained three or more markers for most of the early branches in the crocodilian tree, thus constituting statistically significant support for each branch, respectively, according to the Waddell et al. (2001) likelihood ratio test for retroposon data. This includes six retroposon markers that provide a third, independent perspective on the long-standing “molecules versus morphology conflict” (Harshman et al. 2003) regarding the phylogenetic position of the gharial. In this conflict, virtually all morphological analyses place the gharial as the basal taxon to the remaining extant crocodilians (e.g., Gatesy et al. 2003; Delfino et al. 2008; Sereno and Larsson 2009; Holliday and Gardner 2012; Scheyer et al. 2013). In contrast, molecular sequence-based analyses suggest Alligatoridae (alligators and caimans) as sister to all other crocodilians and support a gharial + false gharial clade (e.g., Gatesy et al. 2003; Harshman et al. 2003; Janke et al. 2005; Meganathan et al. 2011; Oaks 2011). Our conflict-free retroposon markers add to the resolution of this controversy by unequivocally substantiating the latter hypothesis that includes the grouping of true crocodiles, gharial, and false gharial as “Longirostres” (Harshman et al. 2003).

### Low Diversity of Active CR1 Elements during Lineage-Specific Crocodilian Evolution

**Fig. 2.—:**
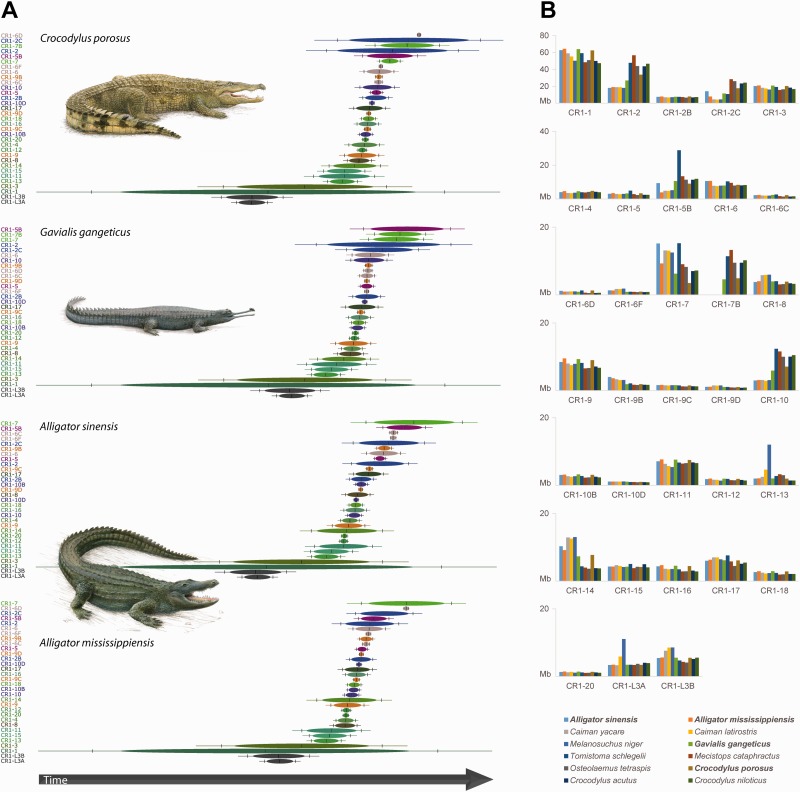
Activity of CR1 elements in 12 crocodilian genomes. (*A*) Estimates of CR1 activity in genome assemblies of saltwater crocodile, gharial, Chinese alligator, and American alligator using the TinT model (Kriegs et al. 2007; Churakov et al. 2010). Congruent successions of predicted activities of merged CR1 subfamilies (see supplementary figure S1, Supplementary Material online, for unmerged TinT patterns) between the four genomes suggest that the core activity periods of most subfamilies lie in the common ancestor of crocodilians. Normal distributions of activity period estimations are shown as ovals (Churakov et al. 2010). (*B*) Comparison of CR1-derived base pair quantities in four assembled genomes (bold names) and eight unassembled survey sequence libraries. All CR1 subfamilies with the exception of CR1-7B appear to be present across the breadth of the sampled crocodilian species diversity. Nevertheless, quantitative differences within several subfamilies suggest differential activities in crocodilian genomes; for example, an extended activity of CR1-2 in Longirostres (all crocodilians except alligators and caimans (Harshman et al. 2003)) and of CR1-5B in *T. schlegelii*. Note that some of the lineage-specific differences in CR1 quantities might either be accounted to potential differences in rate of neutral sequence evolution or cryptic CR1 subfamilies that were not identified in our CR1 predictions (as they are based on the genome assemblies of saltwater crocodile, gharial, and American alligator). English names of the 12 genomes are (in the order of their appearance in the legend) Chinese alligator, American alligator, Yacare caiman, broad-snouted caiman, black caiman, gharial, false gharial, slender-snouted crocodile, dwarf crocodile, saltwater crocodile, American crocodile, and Nile crocodile.

We assigned our CR1 markers to specific CR1 subfamilies sensu Green et al. (Green et al. 2014) and most belong to subfamilies CR1-2/2C, CR1-5B, and CR1-7/7B (fig. 1). This provides direct evidence for the activity of these CR1 subfamilies through large parts of crocodilian evolution, with CR1-7/7B activity having the widest temporal extent. This is because, in figure 1, CR1-7/7B activity can be identified in almost all branches since the last common ancestor of Crocodylia (>87 Ma [Oaks 2011]), including species-specific activity as recent as less than 9 Ma (Oaks 2011) in the saltwater crocodile. We independently verified these observations with an estimation of CR1 activity in the TinT model that considers relative frequencies of insertions of different RE subtypes nested within each other (Kriegs et al. 2007; Churakov et al. 2010). The resulting TinT chronology of CR1 succession (fig. 2*A* and supplementary fig. S1, Supplementary Material online) corroborates that the aforementioned CR1 subfamilies exhibit a long period of activity, including recent activity in the genomes of saltwater crocodile, gharial, Chinese alligator, and American alligator. Furthermore, the TinT patterns are largely congruent among these four genomes, suggesting activity of most CR1 subfamilies in the common ancestor of crocodilians, while only few subfamilies were active since the divergence of Alligatoridae and Longirostres. The same observation is evident when comparing the average intrasubfamily sequence divergence among CR1 copies (supplementary fig. S2, Supplementary Material online) among saltwater crocodile, gharial, Chinese alligator, and American alligator.

To test whether this trend of low lineage-specific CR1 diversity is a common feature of extant crocodilians, we conducted survey sequencing in Chinese alligator (*A. sinensis*) and eight additional species that span the breadth of crocodilian diversity, namely three caimans (*Ca. yacare*, *Ca. latirostris*, *Mel. niger*), false gharial (*T. schlegelii*), and four crocodiles (*C. acutus*, *C. niloticus*, *M. cataphractus*, *Osteolaemus tetraspis*). Given that TE identification in short sequences (e.g., survey data, see Materials and Methods) likely has a reduced reliability than in long sequences, we estimated the whole-genome CR1 content in these species (fig. 2*B*) by normalizing our unassembled survey sequencing libraries with a set of coefficients that was derived from comparing the representation of each CR1 subfamily in our own Chinese alligator survey sequences with the corresponding CR1 subfamily content of a recently published conspecific genome assembly (Wan et al. 2013). We emphasize that these are rough estimates compared with the CR1 quantities measured in the three assembled genomes. Nevertheless, direct comparison of the number of bases assigned to specific CR1 subfamilies in the total of 12 crocodilian genomes (fig. 2*B*) yields many subfamilies with similar amounts of annotated bases among all sampled species. This suggests that the survey sequences are comparable estimates of CR1 quantities and again reveals that most CR1 subfamilies were likely active before the divergence of extant lineages of Crocodylia. On the other hand, subfamilies such as CR1-2, CR1-7B, and CR1-10 appear to exhibit an increased or extended activity common to Longirostres (Crocodylidae + Gavialidae [Harshman et al. 2003]), and we even find evidence for lineage-specific expansion of a CR1 subfamily in the false gharial (CR1-5B), as well as in the black caiman (e.g., CR1-13). These lineage-specific CR1 activities in unassembled genomes might potentially constitute novel subfamilies, given that our de novo predictions of CR1 subfamilies are based on the assembled genomes of saltwater crocodile, gharial, and American alligator.

In addition to our findings, reduced TE diversity within Crocodylia since the divergence of Longirostres from Alligatoridae (∼87 Ma [Oaks 2011]) is also suggested by other collaborative efforts within the International Crocodilian Genomes Working Group. Green et al. (2014) estimated that only approximately 5% of all TE copies were deposited in crocodilian genomes within that timeframe, suggesting an overall decline of both the rate as well as the diversity of TE activity. This applies not only to CR1 activity as described in this study, but also to the activity of DNA transposons that has declined to an even more extreme degree (Green et al. 2014). Instead, retrovirus-like elements constitute about two-thirds of the younger TE-derived DNA in crocodilian genomes (Green et al. 2014) and Chong et al. (2014) suggest that this is the result of multiple infection events of various unrelated retroviral lineages.

### Phylogeny of CR1 Elements Suggests Multiple Ancient Lineages within Amniotes

**Fig. 3.—:**
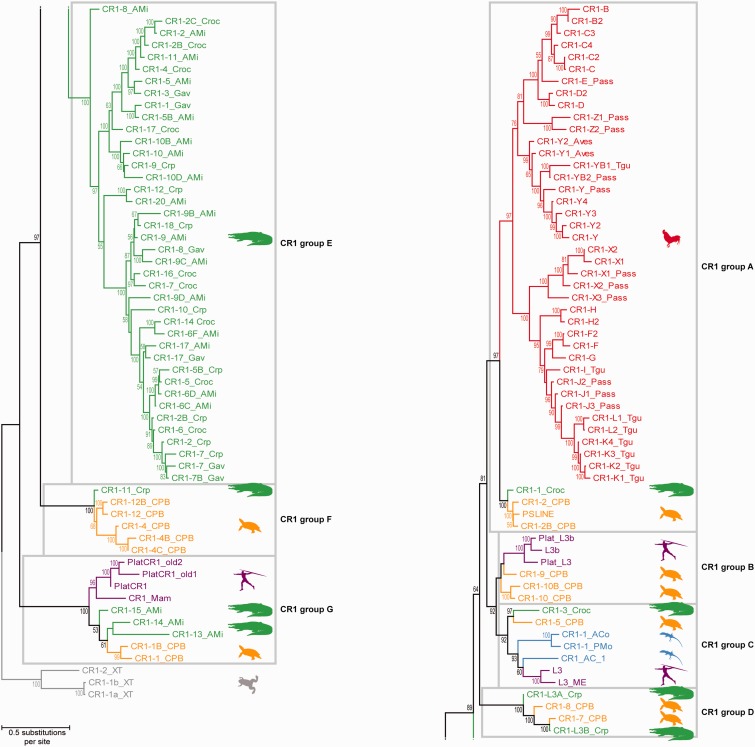
Phylogenetic relationships among amniote CR1 elements. Maximum likelihood nucleotide sequence analysis (RAxML 8.0.0 (Stamatakis et al. 2008), GTRCAT model, 1,000 bootstrap inferences) of part of the ORF2 RT domain + 3′-UTR of all CR1 subfamily consensus sequences available in RepBase (Jurka et al. 2005) suggests a complex tree topology of avian (red), crocodilian (green), turtle (orange), lepidosaurian (blue) and mammalian (purple) CR1 elements. Given this topology and the parsimonious assumption that CR1 retroposons are inherited vertically, we infer a minimum number of seven ancient CR1 lineages (groups A–G; see also supplementary table S3, Supplementary Material online, for a group classification of all analyzed CR1 subfamilies) that were present in the common ancestor of amniotes. The tree was rooted using amphibian CR1 subfamilies from *Xenopus* as outgroup, as they were the only nonamniote CR1 elements alignable to ingroup CR1s on the nucleotide level. Note that, in contrast to the merged CR1 subfamily definitions of figures 1 and 2, we included all CR1 consensus sequences generated in the individual TE annotations of the saltwater crocodile, gharial, and American alligator genomes. Given the short internodes and high sequence similarity among the unmerged crocodilian CR1 subfamilies of group E, we refrain from renaming these CR1 subfamilies according to the topology of the present tree, until additional crocodilian genome assemblies are available. The present nomenclature of crocodilian CR1 subfamilies is therefore solely based on the UCLUST grouping of their consensus sequences (see Materials and Methods). Unlabeled nodes received a bootstrap support of less than 50%.

We reconstructed the phylogenetic relationships of crocodilian CR1 subfamilies based on maximum likelihood analyses of part of the ORF2 RT domain + 3′-UTR of all amniote CR1 subfamily consensus sequences available in RepBase (Jurka et al. 2005). These consensus sequences represent an approximation of the (most often long extinct) master genes that gave rise to the paralogous TE copies visible in genomes. The resultant CR1 tree (fig. 3) was rooted to an amphibian outgroup and exhibits a topology with crocodilian, mammalian, and turtle CR1 subfamilies grouping not according to their hosts, but with many highly diverged species groups which are dispersed among multiple CR1 lineages. Considering this topology together with the phylogenetic relationships among amniotes (Shedlock and Edwards 2009; Shaffer et al. 2013; Green et al. 2014) and assuming vertical transmission as the usual mode of RE inheritance among hosts, the most parsimonious explanation for this is that at least seven CR1 lineages were present in the common ancestor of amniotes (CR1 groups A–G), although it is possible that this cautious estimate could expand with further sampling of host genomes and CR1 subfamilies. It is likely that the complex branching pattern of crocodilian and turtle CR1 subfamilies within CR1 groups D and G is the result of ancient activity in their common ancestor, a hypothesis consistent with their high intrasubfamily divergence levels, the highest among CR1 elements in crocodilian genomes (supplementary fig. S2, Supplementary Material online). On the other hand, the topology within CR1 group C suggests that L3 elements of placental and marsupial mammals (therians) are most closely related to lepidosaurian CR1 subfamilies or even nested within these, which might be the result of multiple independent extinctions of multiple ancient CR1 lineages in most amniote genomes.

Our study is the first genome-scale exploration of CR1 diversity across the breadth of amniote phylogeny. The phylogenetic relationships among amniote CR1 lineages have been previously studied only by Shedlock (2006) and Shedlock et al. (2007) who analyzed 1.5–3.7 Mb of genomic sequences per bacterial artificial chromosome (BAC) library from three birds, one crocodilian, and two lepidosaurs. Those studies suggested that the genomes of nonmammalian amniotes exhibit several ancient CR1 lineages. Our analyses suggest that the same is also the case for mammalian genomes, while our CR1 phylogeny (fig. 3) contains no evidence for nonmonophyly of detectable CR1 subfamilies in birds, which is contrary to BAC-scale analyses that sampled about two dozens of CR1 copies per amniote genome (Shedlock 2006; Shedlock et al. 2007). This is striking because the avian CR1 consensus sequences sampled herein were predicted in the two independent, high-quality TE annotations of chicken (Hillier et al. 2004) and zebra finch (Warren et al. 2010), yet all form a monophyletic clade within the CR1 group A. To explain this discrepancy, we assumed that CR1 elements unrelated to group A have extremely low copy numbers in birds and were thus not detected as distinct TE subfamilies in the aforementioned TE annotations. To test this, we conducted BLASTn (Altschul et al. 1990) searches of representative crocodilian or turtle CR1 consensus sequences from groups B to G against the chicken and zebra finch genomes and analyzed the phylogenetic affinities of all sequence hits in the framework of the CR1 tree of figure 3. The resultant tree (supplementary fig. S3, Supplementary Material online) groups most of these hits within CR1 group A, yet three hits from chicken and three hits from zebra finch cluster within CR1 group F. We therefore propose that, while the sampled bird genomes contain only CR1 group A elements in large copy numbers, there are a handful of CR1 group F elements (supplementary fig. S3, Supplementary Material online) still detectable as remnants of ancient group F activity in the last common ancestor of Archosauria. The high intrasubfamily divergence of CR1 group F copies in crocodilian genomes (supplementary fig. S2, Supplementary Material online) suggests that these elements might have been inactive in Crocodylia for an equally long time, but many copies are still discernible due to the much slower rate of molecular evolution in crocodilians compared with birds (Shaffer et al. 2013; Green et al. 2014).

### Ancient CR1 Lineages Were Differentially Retained within Amniotes

**Fig. 4.—:**
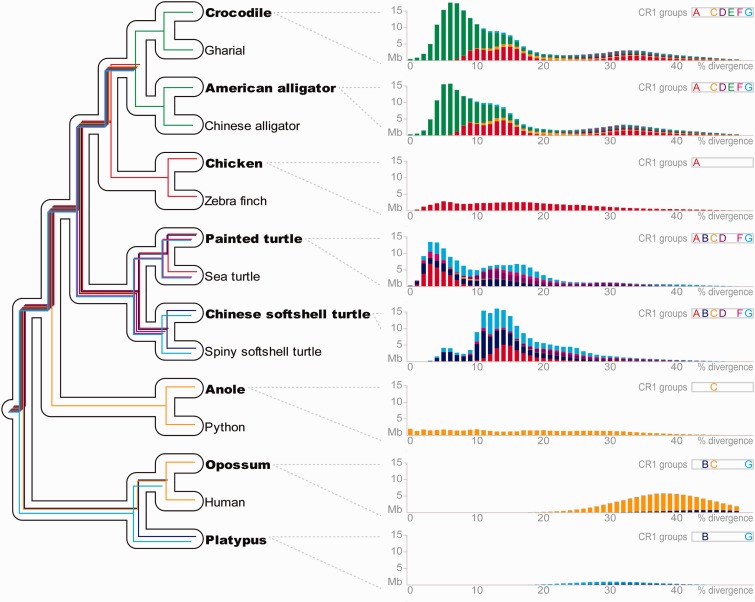
Differential retention of ancient CR1 lineages during amniote evolution. Considering the consensus phylogeny of amniotes (Shedlock and Edwards 2009; Shaffer et al. 2013; Green et al. 2014) (left panel), comparison of divergence landscape plots (right panel; species designations are in bold letters on the left panel) of CR1 groups A–G from figure 3 (see also supplementary table S3, Supplementary Material online) suggests retention of ancient CR1 diversity in crocodilian and turtle genomes, while most CR1 lineages became extinct in the ancestors of other amniote taxa, in particular birds and Lepidosauria (anole and python). We used the distribution of relics of CR1 groups among amniote genomes to parsimoniously infer durations of CR1 lineage retention (branches not to scale). These approximate durations are illustrated using colored lines corresponding to the respective CR1 group (from the right panel). Divergences of CR1 copies to their respective consensus sequences were calculated excluding CpG dinucleotide sites. See supplementary figure S4, Supplementary Material online, for CR1 landscape plots of those species not shown here.

Our classification of amniote CR1 elements into groups A–G according to their aforementioned phylogenetic relationships permitted us to study the impact of these ancient CR1 lineages during amniote evolution. This was accomplished by analyzing their temporal successions and quantitative distributions in the genomes of four crocodilians (Wan et al. 2013; Green et al. 2014), two birds (Hillier et al. 2004; Warren et al. 2010), four turtles (Shaffer et al. 2013; Wang et al. 2013), three lepidosaurs (Alföldi et al. 2011; Castoe et al. 2013; Vonk et al. 2013), and three mammals (Lander et al. 2001; Mikkelsen et al. 2007; Warren et al. 2008). The resultant CR1 landscape plots (fig. 4, right panel; supplementary fig. S4, Supplementary Material online) illustrate the cumulative quantities of CR1 bases plotted against the level of divergence to their respective consensus sequences, which roughly corresponds to a relative time axis, and suggests differential retention of CR1 lineages throughout early amniote evolution (fig. 4, left panel). The four crocodilian genomes have retained all ancient CR1 lineages except group B and the crocodilian CR1 landscapes are almost identical, which suggests that only CR1 group E was active after the onset of the diversification of extant Crocodylia. This is in line with the large amount of closely related CR1 subfamilies within group E, and the fact that all of our CR1 markers for crocodilian phylogeny belong to group E (fig. 1 and supplementary table S1, Supplementary Material online). On the other hand, the avian sister group of crocodilians retained only CR1 group A activity (but see supplementary fig. S3, Supplementary Material online, for low-copy relics of CR1 group F). Similar to the situation in crocodilians, turtle genomes appear to exhibit all CR1 lineages but group E, yet their genomes show a diversity of several ancient CR1 lineages that were active during the evolution of extant turtles. Specifically, CR1 groups A, B, and G were active in the painted turtle and sea turtle lineages, whereas groups B and G were recently active in softshell turtles. Notably, the CR1 landscapes of lepidosaurs contain only CR1 group C elements, and thus bear resemblance to therian mammal genomes that exhibit a predominance of group C elements and low levels of group B and G activity. In contrast to this, CR1 retention in monotreme mammals only comprises detectable elements from CR1 groups B and G.

Our CR1 landscape analyses illustrate contrasting fates of CR1 activity among amniote lineages (fig. 4 and supplementary fig. S4, Supplementary Material online). Although CR1 elements have probably been long extinct since the early evolution of mammals, several of the nonmammalian genomes studied herein suggest ongoing, very recent CR1 activity in some amniote lineages. Evidence for this has so far been limited to the anole lizard (Novick et al. 2009; Alföldi et al. 2011) and one bird lineage, grebes (Suh et al. 2012). In-depth studies of chicken CR1 retroposons suggest that the lineage leading to the chicken recently lost CR1 activity (Hillier et al. 2004; Wicker et al. 2005; Abrusán et al. 2008), which is corroborated by our chicken CR1 landscape (fig. 4) and is similar to the fate of CR1 activity in the lineage leading to the zebra finch (supplementary fig. S4, Supplementary Material online). Our amniote-wide genome analyses suggest extant, “ongoing” CR1 activity in several lineages, as we detected the presence of more than 0.1 Mb of very young CR1 elements (i.e., no sequence divergence from the consensus) in the genomes of anole lizard, painted turtle, sea turtle, and four crocodilians, respectively. Notably, the gharial lineage exhibits the highest extant activity of crocodilian CR1 retroposons with a total of approximately 2 Mb of virtually identical CR1-derived sequences in the gharial genome (supplementary fig. S4, Supplementary Material online). This could be a major part of the explanation of why the gharial genome assembly exhibits the lowest scaffold N50 value of all four assembled crocodilian genomes (Wan et al. 2013; Green et al. 2014). Thus, availability of the gharial genome promises to provide full-length, intact CR1 retroposon sequences for future in vitro studies of the mechanism of amniote CR1 proliferation.

### Conclusions

This study is the first to infer crocodilian RE presence/absence patterns and demonstrates that these cladistic markers provide a conflict-free resolution of deep crocodilian phylogeny, including the unambiguous grouping of gharial and false gharial and the placement of that clade sister to the crocodiles. We provide comparative genomic evidence from 12 crocodilian genomes that, while there was some degree of recent CR1 diversification and succession of activity of CR1 subfamilies throughout crocodilian evolution despite their relative genomic stability (Green et al. 2014), most CR1 diversity and activity was present before the diversification of extant Crocodylia. Our genome-wide analyses of CR1 retroposons across amniote phylogeny revealed that both crocodilians and turtles contain a rich repertoire of ancient CR1 integrations that provide unique insights into the early genome evolution of amniotes. We conducted the hitherto first amniote-wide analyses of CR1 subfamilies and inferred that the genome of the amniote ancestor was impacted by the activity of at least seven CR1 subfamilies that subsequently gave rise to the CR1 groups that are detectable in extant amniotes. Both crocodilians and turtles have retained six of these ancient, mostly long-extinct CR1 groups, respectively. This probably reflects the exceptional genome stability and slow molecular evolution in turtles and crocodilians (Shaffer et al. 2013; Green et al. 2014) that makes these and other nonfunctional sequences (e.g., endogenous hepadnaviruses [Suh et al. 2014]) recognizable even after >200 Myr of neutral decay.

CR1 retroelements are the most abundant, dominant group of TEs in some of the major lineages of amniotes (Shedlock et al. 2007). This applies to the genomes of birds (Hillier et al. 2004; Warren et al. 2010), crocodilians (Green et al. 2014), turtles (Shaffer et al. 2013), and snakes (Castoe et al. 2013), whereas mammalian genomes exhibit dominance of L1 or L2 LINEs (Lander et al. 2001; Mikkelsen et al. 2007; Warren et al. 2008) and the anole lizard genome contains various nearly equally dominant TEs (i.e., CR1 LINEs, L1 LINEs, L2 LINEs, LTR retroposons, DNA transposons) (Novick et al. 2009; Alföldi et al. 2011; Tollis and Boissinot 2011). Considering the phylogenetic relationships among CR1 lineages as well as their abundance in most amniote genomes, it is parsimonious to assume that dominant CR1 activity already existed in the ancestral amniote genome (Janes et al. 2010) (i.e., >320 Ma [Shedlock and Edwards 2009]) and subsequently persisted throughout early amniote evolution. We hypothesize that the diversity and copy numbers of ancient CR1 lineages in the genomes of crocodilians and turtles reflect this ancestral genome organization which therefore must have been preserved in their common ancestor that lived more than 230 Ma (Shedlock and Edwards 2009). This is congruent with studies on amniote genome size evolution that reconstruct an ancestral amniote genome size comparable to that of crocodilians and turtles (Organ et al. 2007, 2011). Consequently, we propose that the smaller genomes of birds and lepidosaurs are the result of genome size reduction in their respective common ancestor via purging of ancient TE copies through rapid molecular evolution, accompanied by reduced TE expansion via inactivation of all but one of the multiple ancestral CR1 lineages. On the other hand, during early evolution of mammals, CR1 activity was replaced by a massive expansion of L2 LINEs in monotremes (Warren et al. 2008) and L1 LINEs in therians (Kordiš et al. 2006), which led to a drastic change in noncoding genome organization and a slight increase in genome size as the result of accumulation of hundreds of thousands of L1/L2 LINEs and L1/L2-mobilized SINEs after decay of ancient TE copies. We therefore conclude that the stable genomes of crocodilians and turtles constitute unique windows into the distant past of early amniote genome evolution and the processes that gave rise to the dissimilar genomic landscapes of mammalian and nonmammalian TEs.

## Supplementary Material

Supplementary data S1 and S2, tables S1–S3, figures S1–S4 are available at *Genome Biology and Evolution* online (http://www.gbe.oxfordjournals.org/).

Supplementary Data
