# Supplementary material for: Multiple Lineages of Ancient CR1 Retroposons Shaped the Early Genome Evolution of Amniotes
Source: Genome Biol Evol. 2014 Dec 11;7(1):205–17. doi: 10.1093/gbe/evu256 (PMC4316615; doi:10.1093/gbe/evu256)
Supplement: Supplementary Data [file supp_7_1_205__index.html]

Multiple lineages of ancient CR1 retroposons shaped the early genome evolution of amniotes — Multiple Lineages of Ancient CR1 Retroposons Shaped the Early Genome Evolution of Amniotes — Supplementary Data 

# Multiple Lineages of Ancient CR1 Retroposons Shaped the Early Genome Evolution of Amniotes

## Supplementary Data

files

**Files in this Data Supplement:**

- Supplementary Data - pdf file
