# Supplementary material for: Multiple Lineages of Ancient CR1 Retroposons Shaped the Early Genome Evolution of Amniotes
Source: Genome Biol Evol. 2014 Dec 11;7(1):205–17. doi: 10.1093/gbe/evu256 (PMC4316615; doi:10.1093/gbe/evu256)
Supplement: Supplementary Data [file supp_evu256_Suh_CR1_supplements.pdf]

Supplementary Information

A

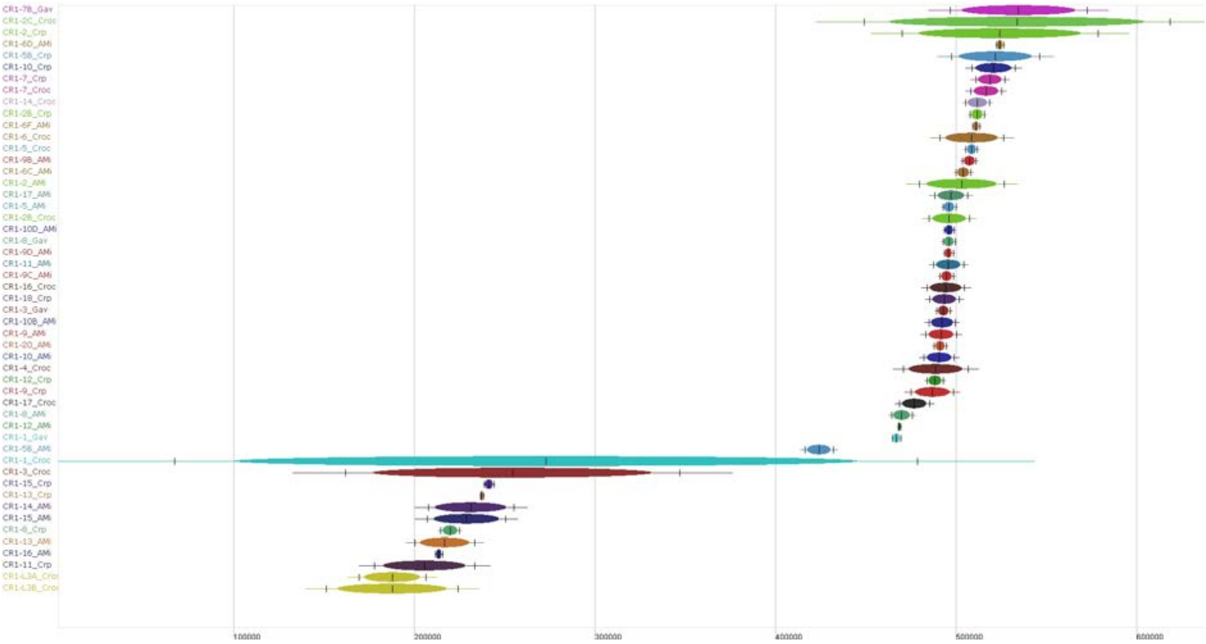

B

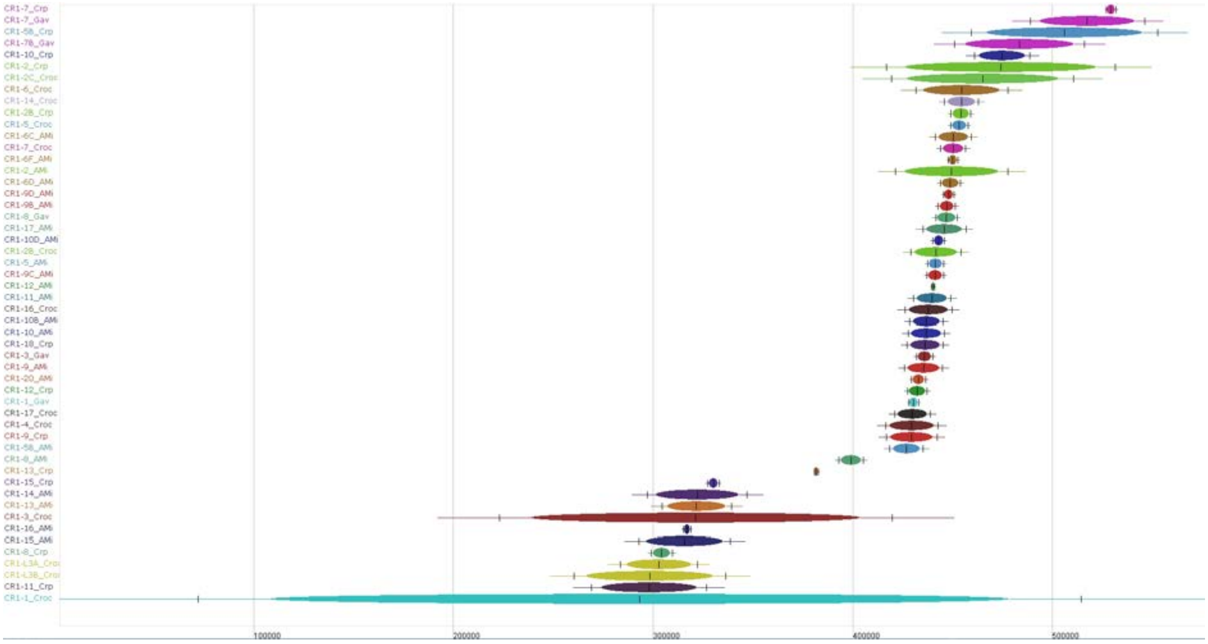

Fig. S1. TinT patterns of unmerged CR1 subfamilies within four crocodilian genomes. The transposition in transposition graphs from (*A*) saltwater crocodile, (*B*) gharial, (*C*) Chinese alligator, and (*D*) American alligator correspond to the computational estimates of CR1 activity in fig. 2A, but comprise all unmerged CR1 subfamily sequences from fig. 3.

C

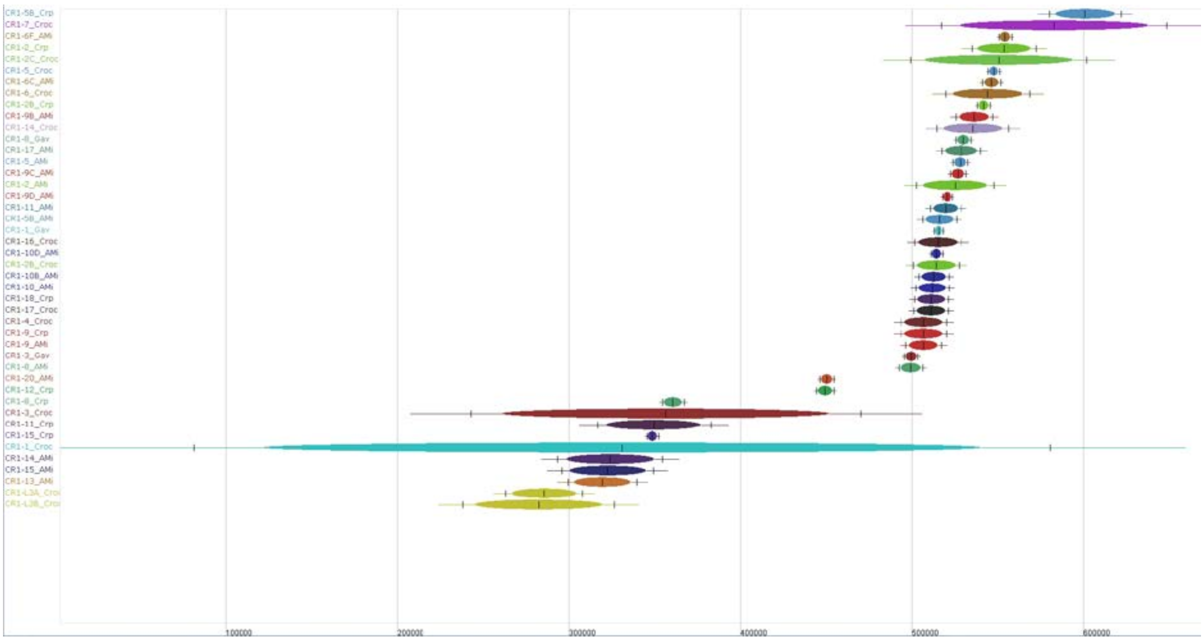

D

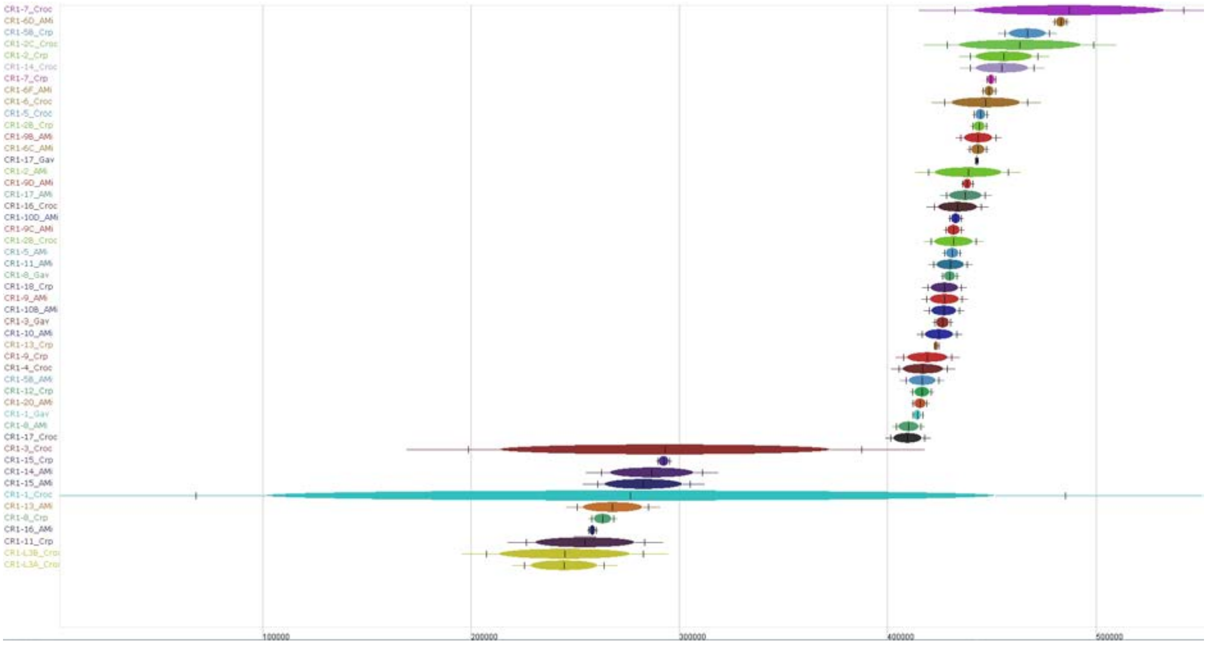

Fig. S1. Continued.

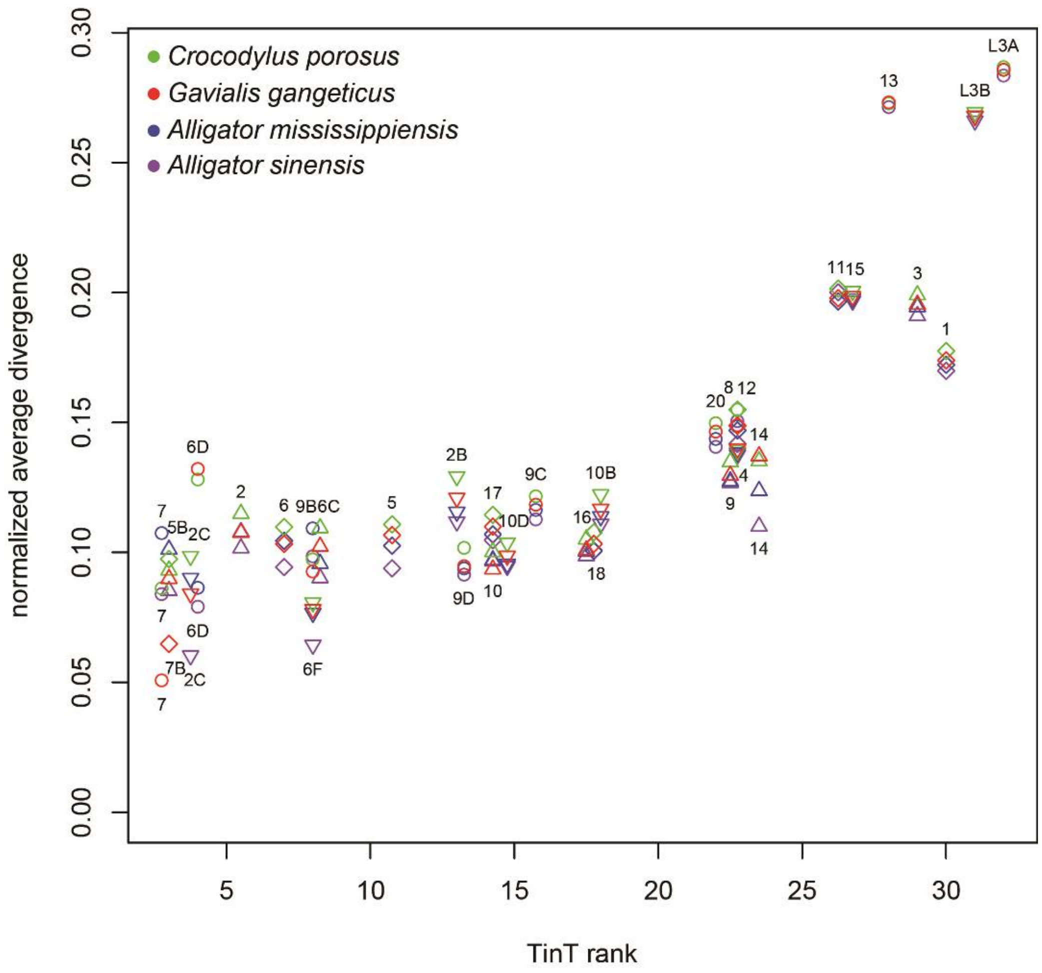

Fig. S2. Average divergence among CR1 subfamilies within four crocodilian genomes. While most CR1 subfamilies exhibit similar average divergences, a few (e.g., CR1-7) show variation between the Saltwater crocodile, gharial, American alligator, and Chinese alligator, suggesting lineage-specific differences in activity as also indicated by fig. 2. Note that the divergence value of each CR1 insertion was normalized for the length of the insertion. The TinT rank is derived from the computational estimation of activity successions of CR1 subfamilies in the transposition in transposition model, wherein recent retropositional activity is reflected by a low TinT rank.

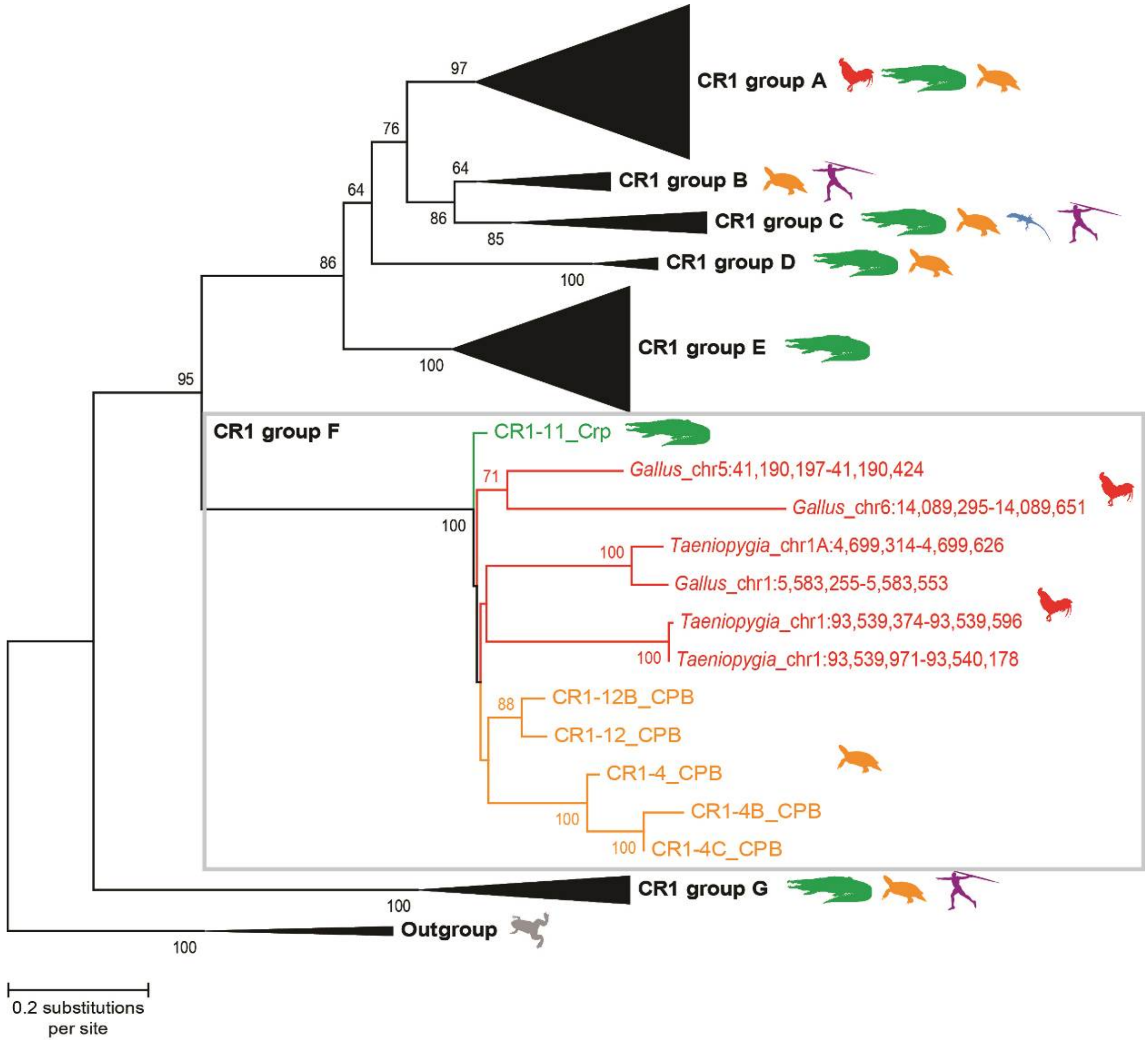

Fig. S3. Low copy number of CR1 group F-like elements in bird genomes. Maximum likelihood nucleotide sequence analysis of the same data set as in fig. 3, but including all BLASTn hits (representatives from crocodilian/turtle CR1 groups B–G as queries, no cutoff e-value, hits >200 bp) from chicken and zebra finch genomes. Unlabeled nodes received a bootstrap support of <50%.

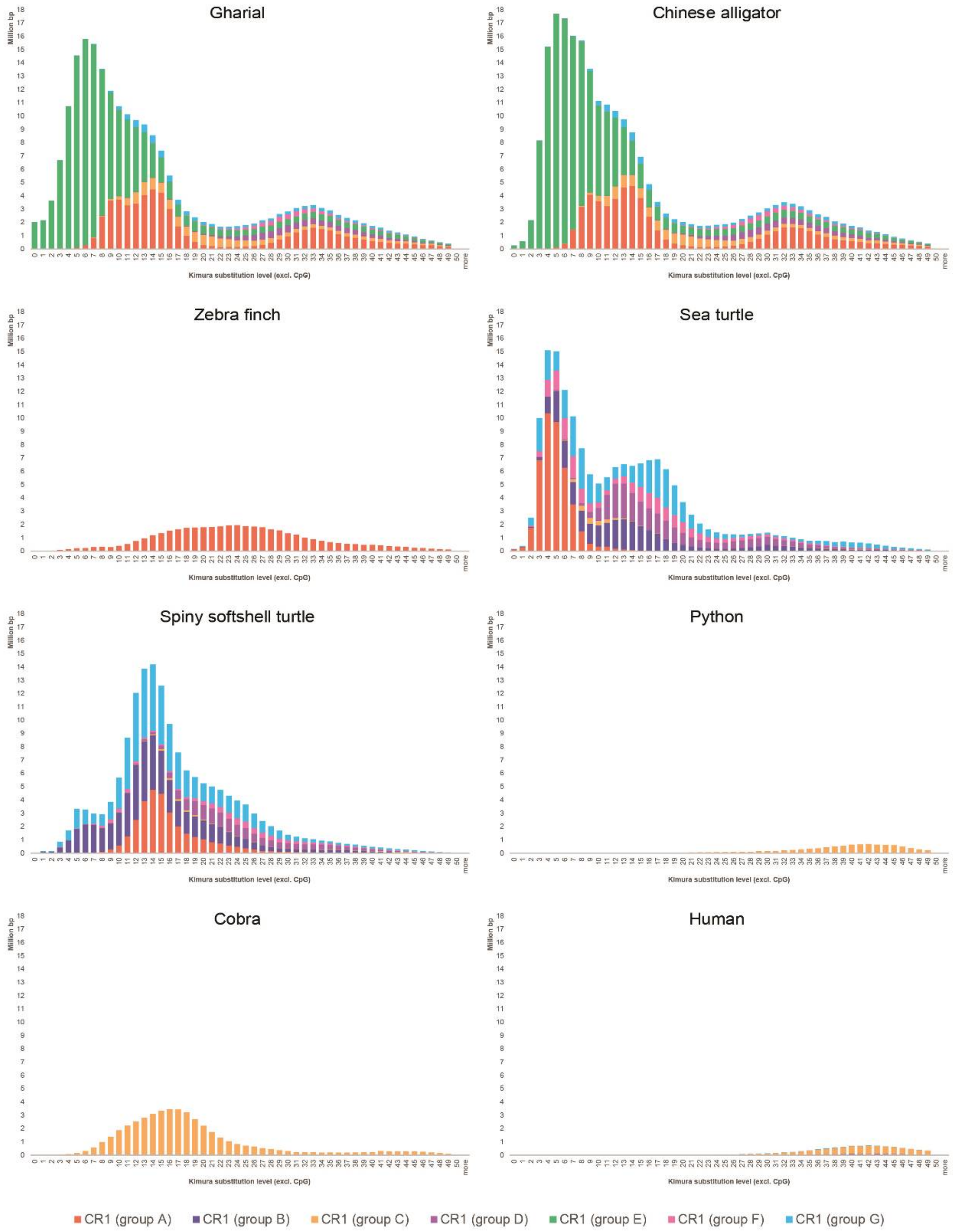

Fig. S4. CR1 divergence landscape plots of amniote genomes not shown in fig. 4. Definitions of CR1 groups A–G correspond to fig. 3 and divergences of CR1 copies to their respective consensus sequences were calculated excluding CpG dinucleotide sites.

Table S1. Presence/absence of RE markers among orthologous loci in crocodilian genomes.

marker	*Cr. porosus*	*Cr. palustris*	*Cr. niloticus*	*Cr. mindoensis*	*Cr. novaeguineae*	*Cr. johnstoni*	*Os. tetraspis*	*Me. cataphractus*	*To. schlegelii*	*Ga. gangeticus*	*Ca. yacare*	*Me. niger*	*Pa. palpebrosus*	*Al. sinensis*	*Al. mississippiensis*	RE subfamily	target site duplication	comments
**AM02**	-								?	-	+	+	+	+	+	Penelope-1_Crp	TTTCAG	*
**AM05**	-								-	-	-	-	-	+	+	Penelope-1_Crp	TGCATCAGTG	
**AM06**	-								-	-	+	+	+	+	+	CR1-7_Croc	GTT	
**AM07**	-								-	-	-	-	-	+	+	CR1-7_Croc	AACTT	
**AM09**	-									-				-	+	CR1-7_Croc	TT	**
**AM15**	-									-	-		-	-	+	CR1-2C_Croc	GAAAT	
**AM21**	-								-	-	+	+	+	+	+	CR1-2_Crp	GACT	***
**AM22**	-								-	-	+	+	+	+	+	CR1-2_Crp	A	
**AM24**	-								-	-	+	?	+	+	+	CR1-14_Croc	AAA	
**AM25**	-								-	-	+	+	+	+	+	CR1-2_Crp	AC	
**CP01**	+		+		+	+	-	-	-	-	-			-	-	CR1-2C_Croc	GAC	
**CP06**	+		+		+	+	-	-	-	-	-			-	-	CR1-2C_Croc	ATATC	
**CP07**	+		+		+	+	-	-	-	-	-			-	-	CR1-2C_Croc	TG	
**CP08**	+				+		+	+	-	-	-			-	-	CR1-4_Croc	T	
**CP10**	+				+		+	+	-	-	-			-	-	CR1-7B_Gav	AAC	
**CP13**	+				+		+	+	?	-	-			-	-	CR1-2C_Croc	CTTAT	
**CP14**	+				+		+	+	-	-	-			-	-	CR1-7B_Gav	AAT	
**CP15**	+	-		-						-				-	-	CR1-7_Crp	G	
**CP18**	+				+		+	+	-	-	-			-	-	CR1-2C_Croc	GTTT	
**CP19**	+				+		+	+	-	-	-			-	-	CR1-7B_Gav	TGTAAT	
**GC01**	+						+	+	+	+	-			-	-	CR1-2_Crp	AACC	****
**GC03**	+						+	+	+	+	-			-	-	CR1-2_Crp	CATGT	
**GC04**	+						+	+	+	+	-			-	-	CR1-7B_Gav	TATTTC	
**GC08**	+						+	+	+	+	-			-	-	CR1-2_Crp	AACC	
**GC09**	+						+	+	+	+	-			-	-	CR1-7B_Gav	AAA	
**GC12**	+						+	+	+	+	-			-	-	ERV1-4_Crp-LTR	GTTG	
**GG01**	-						-	-	+	+	-			-	-	CR1-7B_Gav	ATGAAT	
**GG03**	-								-	+				-	-	CR1-5B_Crp	ATTTCA	
**GG10**	-								-	+				-	-	CR1-7_Gav	AGAGG	
**GG12**	-						?	-	+	+				-	-	CR1-5B_Crp	TCTTG	
**GG13**	-						-	-	+	+				-	-	CR1-5B_Crp	AATATA	
**GG14**	-								-	+				-	-	CR1-5B_Crp	AGCTTC	
**CG_0062**	+									+				+	+	CR1-2_AMi	?	
**CG_0066**	+									+				+	+	CR1-9B_AMi	CCA	
**CG_0077**	+									+				+	+	CR1-17_AMi	?	
**CG_0087**	+									+				+	+	CR1-6C_AMi	?	

Character states are '**+**' for RE presence, '**-**' for absence, and '**?**' for missing data. Blank cells: not analyzed.

*****: 17 nt insertion in Caimaninae and 17 nt deletion in *Caiman + Melanosuchus* . ******: TE most similar to CR1-derived insertion within Gypsy-38_AMi-I-int consensus.
*******: 267 nt deletion in Caimaninae and 7 nt deletion in *Caiman + Melanosuchus* . ********: 9 nt deletion in Crocodylidae and 197 nt deletion in Gavialidae.

Table S1. Continued.

marker	locus scaffold in allMis1	locus orientation in allMis1	locus start in allMis1	locus end in allMis1
**AM02**	JH739241	+	386608	387618
**AM05**	JH734058	+	123912	124245
**AM06**	JH733477	+	407117	407956
**AM07**	JH734288	+	182819	183242
**AM09**	JH732763	+	178094	178907
**AM15**	JH733358	+	947797	948668
**AM21**	JH734499	+	399867	400499
**AM22**	JH734753	+	766093	767197
**AM24**	JH734874	+	172032	172987
**AM25**	JH735357	+	331471	332118
**CP01**	JH734442	+	536671	537582
**CP06**	JH732326	-	1186970	1187967
**CP07**	JH735857	-	551518	552142
**CP08**	JH737363	+	3079328	3079611
**CP10**	JH739179	+	157198	157874
**CP13**	JH738577	+	133118	133833
**CP14**	JH731454	-	464555	465406
**CP15**	JH731391	+	1188298	1189162
**CP18**	JH734773	+	889393	889951
**CP19**	JH739451	+	243914	244427
**GC01**	JH737872	+	271884	272456
**GC03**	JH737792	-	6210	6986
**GC04**	JH737792	-	146993	147574
**GC08**	JH732327	+	620975	621602
**GC09**	JH733358	-	462882	463085
**GC12**	JH736113	-	1205940	1206409
**GG01**	JH732121	-	128337	128746
**GG03**	JH731430	+	399807	400518
**GG10**	JH733284	+	84786	84936
**GG12**	JH738572	+	46606	46823
**GG13**	JH736752	+	50716	51014
**GG14**	JH737086	-	985458	985734
**CG_0062**	JH731248	-	267734	269121
**CG_0066**	JH731145	-	355472	356412
**CG_0077**	JH731451	+	5082	6528
**CG_0087**	JH734874	+	258990	260122

allMis1: American alligator assembly in UCSC Genome Browser (http://genome.ucsc.edu/cgi-bin/hgBlat?command=start).

Table S2. Oligonucleotide primer sequences.

Marker	forward primer (5'-3')	reverse primer (5'-3')
AM02	AGGTGGGATGGAAAACAAC	TGGTCAACGTAACAGAACTGG
AM05	TTGTGCGTCCAGAACAGC	GGTTGCCTGGGATCAGAG
	CAGAACAGCCCTCAGGAAG	
AM06	GTTGGAGATTCTCATCTTGGAG	TCATTATCGTAGATGGTGTAGTAGC
AM07	ACACAGGAACAACCCATCG	ACCTGGCAAAGCTGGATG
		TGGCAAAGCTGGATGACA
AM09	ATGGACCAAGATGAGGAGG	CAAAGAGAATCAGTCCCAAAG
AM15	TCATCTCTGGTGACAATGCTG	CTCTCCTCAGGGGTGAAGTC
	ATCTCTGGTGACAATGCTGAG	CTGTGACTGCCGTGGAAG
AM21	TTCAAGTCCAAGAAGAAGGTG	GCTCAGCTTGTGGCACAC
	CCAACAACTCCTTCAGGTG	
AM22	TTCGGACAGGCTATCAAGG	AATGCCCTCTGAGTTTCCTC
	CCCTCAAAGCCTCTTCTACC	GACCATCACCTCTCCTGTCTC
AM24	AAGTTTCCATGATTGGCTACAG	CCCACCAGAACCTAGTGACAG
AM25	TGCCAGAGCACTTAACAGTTG	CAGATCCCCACCTTCCAAG
CP01	TTTGCCCTCTCCAACTCC	CTACTACTCCATCCATCAGATAGG
CP06	ATAATCTTGCTGGGCTATGC	TCTGGAGATGTGACACTGTTATG
CP07	AACATCCCCAACATCATCC	GAGGCGATTCTCCTGTCAC
	CCCCAACATCATCCTTACAG	TCCTGTCACTGGAATCAAGAG
CP08	AAGATTATGAGCAAGTAGCAGG	GTTCAATGTGCTTTAGGAAGG
CP10	GACTGCTGAAGAGAGAGACTGC	ATGAGCTTTGGCTTCTTGG
CP13	CATGGGCAGATATTGGAGC	TGGATTCCGAACAGGTGC
CP14	GCTTGTCCTGTTTTGTTACTCC	TCTGAATGTATGGTGAAGGTCTG
CP15	GGAGGAAGTTGGTGTTGAAC	CCACTGTTGTGTCCTTAATATCTC
CP18	AGCACATGCCAACAAACC	CTGCTGAATTGACGAATGC
CP19	ACAGATGAAGCCCTGGTAAG	GCACAGCAGCTACAGTGAAAG
GC01	ACATCAGGGCTTGCTTCTC	CGTGAAGATGCTCAAGTGTG
GC03	GCCTACAGCAGTGACACACC	AATCAGTTTGTTCAGTGGTTCC
GC04	GAGACCCTCAGACCTTCAATC	AGGAAGACAGAGAAGATCACAAG
GC08	CATCACAGTGCGATCTCCAG	ACATTGCTTTCTTCTCCACTAAC
GC09	AGTAGGGTTCATGGAACATAGC	ACAGCATTCAAAGCCTTTTC
GC12	CTTTCCAGCCGTGTGAGAG	GGTGTTCCATCGCTTGAAG
GG01	GCATCATCAAAAGCTGTAATG	GGTAGTGAAGAGGTTGCCTG
		CAGATATGTGAACCTACTGAAAAG
GG03	CATCCTCACTGAAATCTCACAG	TCTTGTCAATGATCTCCATCTC
GG10	TCACACTGAACTCTGGACAGG	CTCTGTGGGCTCCTCATTC
GG12	GCTTGGGATACACAGGAGG	GGAGGGTTGTCTTCTGTTCTG
GG13	CTTGTTTTACAGCAAGGAAGG	GAGATATTAAGAAGGAGACCAGG
GG14	CCCTAATTTCTTTGCCTAGATAG	TGATGAAGCCTTACTGGTGC

Table S3. CR1 group classification of all analyzed amniote CR1 subfamilies.

CR1 group A	CR1 group B	CR1 group C	CR1 group D	CR1 group E	CR1 group F	CR1 group G
CR1-B	CR1-9_CPB	CR1-3_Croc	CR1-L3A_Croc	CR1-1_Gav	CR1-11_Crp	CR1-13_AMi
CR1-B2	CR1-10_CPB	CR1-5_CPB	CR1-L3B_Croc	CR1-10_AMi	CR1-12_CPB	CR1-14_AMi
CR1-C	CR1-10B_CPB	CR1-1_ACo	CR1-7_CPB	CR1-10_Crp	CR1-12B_CPB	CR1-15_AMi
CR1-C2	L3b	CR1-1_PMo	CR1-8_CPB	CR1-10B_AMi	CR1-4_CPB	CR1-1_CPB
CR1-C3	Plat_L3	CR1_AC_1		CR1-10D_AMi	CR1-4B_CPB	CR1-1B_CPB
CR1-C4	Plat_L3b	L3		CR1-11_AMi	CR1-4C_CPB	CR1_Mam
CR1-D		L3_ME		CR1-12_AMi		PlatCR1
CR1-D2				CR1-12_Crp		PlatCR1_old1
CR1-E_Pass				CR1-13_Crp		PlatCR1_old2
CR1-F				CR1-14_Croc		
CR1-F2				CR1-15_Crp		
CR1-G				CR1-16_AMi		
CR1-H				CR1-16_Croc		
CR1-H2				CR1-16_Crp		
CR1-I_Tgu				CR1-17_AMi		
CR1-J1_Pass				CR1-17_Croc		
CR1-J2_Pass				CR1-17_Crp		
CR1-J3_Pass				CR1-17_Gav		
CR1-K1_Tgu				CR1-18_Crp		
CR1-K2_Tgu				CR1-2_AMi		
CR1-K3_Tgu				CR1-2_Crp		
CR1-K4_Tgu				CR1-20_AMi		
CR1-L1_Tgu				CR1-2B_Croc		
CR1-L2_Tgu				CR1-2B_Crp		
CR1-X1				CR1-2C_Croc		
CR1-X1_Pass				CR1-3_Gav		
CR1-X2				CR1-4_Croc		
CR1-X2_Pass				CR1-5_AMi		
CR1-X3_Pass				CR1-5_Croc		
CR1-Y				CR1-5B_AMi		
CR1-Y_Pass				CR1-5B_Crp		
CR1-Y1_Aves				CR1-6_Croc		
CR1-Y2				CR1-6C_AMi		
CR1-Y2_Aves				CR1-6D_AMi		
CR1-Y3				CR1-6F_AMi		
CR1-Y4				CR1-7_Croc		
CR1-YB1_Tgu				CR1-7_Crp		
CR1-YB2_Pass				CR1-7_Gav		
CR1-Z1_Pass				CR1-7B_Gav		
CR1-Z2_Pass				CR1-8_AMi		
CR1-1_Croc				CR1-8_Crp		
PSLINE				CR1-8_Gav		
CR1-2_CPB				CR1-9_AMi		
CR1-2B_CPB				CR1-9_Crp		
				CR1-9B_AMi		
				CR1-9C_AMi		
				CR1-9D_AMi		

Note that font colors correspond to the taxon designations of fig. 3.

Supplementary data S1

36 loci of crocodilian retroposon markers in FASTA format. Consensus sequences of retroposed elements represent the first sequence. The second sequence indicates exonic regions (Exons; if available). Experimentally derived sequences are labeled in a 3-letter code for species names (e.g., Cya for *Caiman yacare*). Other sequences were extracted from genomic scaffolds or from the NCBI GenBank (GB). Intronic sequences and inserted retroposons (and target site duplications) are separated by 3-dashes. Ns denote uncharacterized nucleotides. The loci are sorted by names of genomes that were used for primer design (*Alligator mississippiensis* (AM), *Crocodylus porosus* (CP), *Gavialis* plus *Crocodylus* (GC), *Gavialis gangeticus* (GG) and electronic search for monophyly markers (CG)).

AM02

```
>Penelope-1
--------------------------------------------------------------------------------
--------------------------------------------------------------------------------
--------------------------------------------------------------------------------
--------------------------------------------------------------------------------
--------------------------------------------------------------------------------
------------------------------TTTTCACTGACTGCCTGTCTTGTATGCAGACCAGCCCAGCCTCTGGCTTC
TTTACTTTTCATTCCATCCAGGAAGAGCACACACCAACTGCTGAAACTTCCTTAGCCTGACGAAGGGTTTTTGAACCCGA
AAGCTTGCTTAATAATTGTTTTCCAACTATTTAAGTTGGTCTAATAAAAGATATCAGATTCACCCAAAGAACCTTGTCTG
--------------------------------------------------------------------------------
--------------------------------------------------------------------------------
--------------------------------------------------------------------------------
--------------------------------------------------------------------------------
--------------------------------------------------------------------------------
------------------------------------
>Exons
CCGTGAATATTACACATTCATGTGGGCACCTTTGCCCAGCAGCCTTGACAGCGATGCAGTGCTGCAGCAAGAAGTTCAGT
TTAAAG--------------------------------------------------------------------------
--------------------------------------------------------------------------------
--------------------------------------------------------------------------------
--------------------------------------------------------------------------------
--------------------------------------------------------------------------------
--------------------------------------------------------------------------------
--------------------------------------------------------------------------------
--------------------------------------------------------------------------------
--------------------------------------------------------------------------------
--------------------------------------------------------------------------------
--------------------------------------------------------------------------------
--------------------------------------------------------------------------------
-----CCTACTACCTGCCAAAAGATGATGAATATTA
>Cpo_scaffold04922
CCGTGAATATTACACATTCATGTGGGCACATTTGCCCAGCAGCCTTGACAGCAATGCAGTGCTGCAGCAAGAAGTTCAGT
TTAAAG---GTTGGCTGGGGAAGGATGGGTGTC---------------------------------------CGTGCACA
CGTGCAGATAGATACACACACACGCAC-ACTATCACATACCAAATTTCTCTAAAGGCTAGGT-TACATGTTACAGC----
--------------CTGAGGCTGTAGAACTATTGAATA-TTTCATCCTGTTATACGCTGTG-TTTAGACAATCTGTA-TT
TCAGGAACTATGGCTATCAAGCAATCTCAAAATAATAGCCACCAAATTTCTACCCAGGGTTTCTTCCACCAGCCTGTCAG
TCTGCTGGTGCTGTCAGTGTAGATCAGCCTTGCATGAGTGCCCCCTGCTGGTAGAACAGAAACATATCT---TTTCAG--
--------------------------------------------------------------------------------
--------------------------------------------------------------------------------
-------------TACATTCACTTCTGAGGCCTGTGTTACCAGCGCACTACAAACCCAACCTAGGGGGAATACCCCTTTG
GAGGTCATAGTAACCTGGGTGTGACCATTCATAATGTTTTAGTGATAGGGCCAAGTTCATCACTGCCATGCTGCTTGTGG
AGTGTATTGTGACACAAAATGCCAAGGTTGTAATTTGGAAGCGTTTTTGGCACAATTTGCACTGGTGTAAAAGATCACAC
AAGGAGTAGGCAATGCTGAGCCCATAATGGCTATATTGCTTTGTATTCAGTACTTCTGCTTTTTGTAGATTTAAGCTTTC
TGTCCCATTCACTGAAGAAGAGGCAATAATAATTAGAGACACCCCATGGTGCAAC---TTTTGTTTGTTTGTTTGTTTTA
AG---CCTACTACCTGCCAAAAGATGACGAATATTA
>Gga_scaffold4577
CCGTGAATATTACACATTCATGTGGGCACCTTTGCCCAGCAGCCTTGACAGCGATGCAGTGCTGCAGCAAGAAGTTCAGT
TTAAAG---GTTGGCTGGGGAAGGATGGGTG-TCCATACACATGTG----------------------------------
------CACATGTGCAGATACACACAT-ACTATGACATACCAAATTTCTCTAAAGGCCAGGTATACATACATGTTACAGC
--------------CTGAGGCTGTAGAACTATTGAATA-TTTCATCCTGTTATGCACTGTG-TTTAGACGATCTGTA-TT
TCAGGAACCGTGGCTATCAAGCAATCTCAAAATAATAGCCACCAAATTTCTACCCAGGGTTTCCTCCACCAGCCTGTCAG
TCTCCTGGTGCTGTCAGTGTAGATCAGCCTTGCATGAGTGCCCCCTGCTGGTAGAACAGAAACTTATCC---TTTCAG--
--------------------------------------------------------------------------------
--------------------------------------------------------------------------------
-------------TACATTCACTCCTGAGGCCTGTGTTACCAGCGCACCACAAACCCAACCTAGAGGGAATACCCCTTTG
GAGGTCATAGTAACCTGGGTGTGACCATTCATAATGTTTTAGTGATAGGGCCAAGTTCATCACTGCCATGCTGCTTGTGG
```

AGTGTATTGTGACACAAAATGCCAAGGTTGTGATTTGGAAGCGTTTTTGGCACAATTTGCGCTGGTGTGAAAGATCACAC
AAGGAGTAGGCAATGCTGAGCCCATAATGGCTATATTGCTTTGTATTTGGTACTTCTGCTTTTTGTAGATTTTAGCTTTC
TGTCCCATTCGCTGAAGAAGAGGCAATAATAATTAGAGACACCCCATGGTGCGATTTTGTTTGTTTGTTTGTTTGTTTTA
AG---CCTACTACCTGCCAAAAGATGACGAATATTA
>Cya
---------------------------------GCCTAGCAGCCTTGACAGCGATGCAGTGCTGCAGCAAGAAGTTCCAT
TCAGAG---GTTGGCTGGGGAAGGATGGGTGGTCCATACACATGTGCACACATGCA----------------GATACACA
CACACACACACACACACACACACACGC-AAAATGACATATCAAATTTCTCTAAAAGGGACGTATATATGTAACAACTCTG
TATAAA-AGTTGGGCTCACGCTGTAAAACAGTTGAATA-TGTCTGACTGTTGG--GCTGTG-GTCAGACAGGCTGTA-TT
TCAGGAGCCATGGCTATATAGAAATCTCAAAAAAATAGCCACCAGATTTGTCCCCGGGGTTTTCTCTCCCCCCCTCTCAC
GCTGTTTGTGCTCTCTGTGGATATAACCCTCTTGTGAGAGCCCCCTGTTGTGAGAAAACAAATGTGTCC---TCTCAC--
-AAATATATCTTCTCCCCCAGGAAGAGAACACACCCACTGCGGAAACTTCCTTAGCCTGACGAAAGGTTTTTGAACCCAA
AAGCTTGCTTAATAACTTTTCTCCAATTATTTGGGTTGGTCTAATAAAAGATATCAAATTCACCCAAGGAACCTTGTCTC
C---TTTCAG---TACATTCACTCCTGAGGGCTGTGTTACTAGCACACCACAAAC-----------------CCCCTTTG
GAGGTTGTAGTAACCTGGGTCTGACCATTCATAATGTTTTAGTGATAGGGTCAAGTTCATCACTGCCATGCTGCTTGTGG
AGTGTATTGTGACACAAAATGCCCAGGTTGTGATTTGGAAGTGTTTTTGGCACAATTTGCACTGGTGTGAAAGATCACAC
AAGGAGTAGGCAATGCTGAGCCCATAATGGCTATATTGCTTTGTATTCAGTACTTCTGCTTTTTGTAGATTTAAGCTTTC
TGTCCCATTCACTGTAGAAGAGGCAATAATAATTAGAGACACCCCATGGTGTGATTTTTGTGT-----------------
----
>Mni
CCGTGANTATTACACATTCATGTGGGCACCTTTGCCTAGCAGCCTTGACAGCGATGCAGTGCTGCAGCAAGAAGTTCCAT
TCAGAG---GTTGGCTGGGGAAGGATGGGTGGTCCATACACATGTG--------------------------CACACATG
CAGATATACACACACACACACACACAC-AAAATGACATATCAAATTTCTCTAAAGGCCAGGTATACATGTTACAACTCTG
TAGAAC-AATTGTTCTCAGGCTGTAGAACAATTGAATA-TTTCATCCTGTTGT--GCTGTG-CTTAGGCAATCTGCA-TT
TCAGGAACCATGGCTATCAAGCAGTCTCAAAATAATAGCCACCAAATTTCTACCCAGGGTTTCCTCTACCAGCCTGTCAG
GCTGCTTGTGCTGTCAGTGTAGATCAGCCTTGCATGAGTGCCCCCTGCTGGTAGAACAGAAATGTGTCC---TTTCAG--
-AAATGTATCCTTTCCTCCAGG--
---GTCTC
C---TTTCAG---TACATTCACTCCTGAGGGCTGTGTTACTAGCACACCACAAAC-----------------CCCCTTTG
GAGGTTGTAGTAACCTGGGTCTGACCATTCATAATGTTTTAGTGATAGGGTCAAGTTCATCACTGCCATGCTGCTTGTGG
AGTGTATTGTGACACAAAATGCCCAGGTCGTGATTTGGAAGTGTTTTTGGCACAATTTGCACTGGTGTGAAAGATCACAC
AAGGAGTAGGCAATGCTGAGCCCATAATGGCTATATTGCTTTGTATTCAGTACTTCTGCTTTTTGTAGATTTAAGCTTTC
TGTCCCATTCACTGTAGAAGAGGCAATAATAATTAGAGACACCCCATGGTGTGATTTTTG---------TGTTTGTTTTA
AG---CCTACTACCTGCCAAAAGATGATGAATATTA
>Ppa
---------------------------------CCTNGNAAATGCTAACGGCTATTCAAAGCTGCAGCAAGAAGTTCCAT
TTAAAC---GTTGGCTGGGGAAGGATGGGTGGTCCATACAACATCTGCACACATG-------------------------
------GCAGATACACACACACGCAC--AAAATGACATACCAAATTTCTCTAAAGGCA-----TACATGTTACAACTCTG
TAGAACAAATTGTTCTCAGGCTATAGAACAATTGAATATTTTCATCCTGATGT--GCTGTG-CTTAGGCAATCTGCA-TG
TCGAGGACCATCGGGGTCAAAATGTCTCGAAATGATAGCCGCGAAAGTTCTACCCAGATTTTCCTCGACCTGCTGGTCAG
GCTGGCTCTGCTGTCAGTGAAGATCAGCCTTGCATGGGTGCCCCCTGCTGGTATAACAGAAATGTGTCC---TTTCAT--
-AAATGTATCCTTTCCTCCAGGAAGAGCACACACCAACTGCTGAAACTTCCTTAGCCTGAGGAAGGGTTTTTGAACCCAA
AAGCTTGCTTAATAACTTTTCTCCAATTATTTGGGTTGGTCTAATAAAAGATATCAAATTCACCCAAGGAACCTTGTCTC
C---TTTCAG---TACATTCACTCCTGAGGGCTGTGTTACTAGCACACCACAAACCCAGCCTAAGGGAAGTACCCCTTTG
GAGGTTGTAGTAATCTGGGTCTGACCATTCATAATGTTTTAGTGATAGGGTCAAGTTCATCACTGCCATGCTGCTTGTGG
AGTGTATTGTGACACAAAATGTCCAGGTTGTGATTTGGAAGCGTTTTTGGCACAATTNGCNTGGGTGTGAAAGATCCCTC
AAGGAGTAGGCAATGCAGAGCCCATAATGGCTATATTGCTNTGTATTCAGTANNAAAGCTTTTGGCAGATTTGAACTTTC
TCTTCCATTCANTGGAGAAGAGGCAATTATAAGTTGA---

>Asi_533315122
CCGTGAATATTACACATTCATGTGGGCACCTTTGCCCAGCAGCCTTGACAGCGATGCAGTGCTGCAGCAAGAAGTTCAGT
TTAAAG---GTTGGCTGGGGAAGGATGGGTGGTCCATACACATGTGCGCGCGCGGTCGCGCACACACACACGCACACACA
CACACACACGCACACACACACACACAC-ACAATCACATACCAAATTTCTCTAAAGGCCAGGTATACATGTTACAAC----
--------------CTGAGGCTGTAGAACTATTGAATA-TTTCATCCTGTTGT--GCTGTG-CTTAGGCAATCTGCA-TT
TCAGGAACCATGGCTATCAAGCAATCTCAAAATAATAGCCACCAAATTTCTACCCAGGGTTTCCTCTACCAGCCTGTCAG
TCTGCTTGTGCTGTCAGTGTAGATCAGCCTTGCATGAGTGCCCCCTGCTGGTAGAACAGAAATGTATCC---TTTCCT--
-----------------CCAGGAAGAGCACACACCAACTGCTGAAACTTCCTTAGCCTGAGGAAGGGTTTTTGAACCCGA
AAGCTTGCTTAATAACTTTTCTCCAACTGTTTGGGTTGGTCTAATAAAAGATATCAAATTCACCCAAGGAACCTTGTCTC
C---TTTCAG---TACATTCACTCCT-------GTGTTACCAGCGAACTACAAACCCAACCTAGGGGAAATACCCCTTTG
GAGGTTGCAGTAACCTGGGTGTGATCATTCATAATGTTTTAGTGATAGGGTCAAGTTCATCACTGCCATGCTGCTTGTGG
AGTGTATTGTGACACAAAATGCCCAGGTTGTGATTTGGAAGCGTATTTGGCACAATTTGCACTGGTGTGAAAGATCACAC
AAGGAGTAGGCAATGCTGAGCCCATAATGGCTATATTGCTTTGTATTCAGTACTTCTGCTTTTTGTAGATTTAAGCTTTC
TGTCCCATTCACTGTAGAAGAGGCAATAATAATTAGAGACACCCCATGGTGTGATTTTTG---------TGTTTGTTTTA
AG---CCTACTACCTGCCAAAAGATGATGAATATTA
>Ami_13548_386350-387900
CCGTGAATATTACACATTCATGTGGGCACCTTTGCCCAGCAGCCTTGACAGCGATGCAGTGCTGCAGCAAGAAGTTCAGT
TTAAAG---GTTGGCTGGGGAAGGATGGGTGGTCCATACACATGTGAGCATGCGCGCG-------------CACACACAC
ACACA-CACACACACACACACACACAA-AAAATCACATACCAAATTTCTCTAAAGGCCAGGTATACACGTTACAAC----
--------------CTGAGGCTGTAGAACTATTGAATA-TTTCATCCTGTTGT--GCTGTG-CTTAGGCAATCTGCA-TT
TCAGGAACCATGGCTATCAAGCAATCTCAAAATAATAGCCACCAAATTTCTACCCAGGGTTTCCTCTACCAGCCTGTCAG
TCTGCTTGTGCTGTCAGTGTAGATCAGCCTTGCATGAGTGCCCCCTGCTGGTAGAACAGAAATGTATCC---TTTCAG--
-AAATGTATCCTTTCCTTTAGGAAGAGCACACACCAACTGCTGAAACTTCCTTAGCCTGAGGAAGGGTTTTTGAACCTGA
AAGCTTGCTTAATAACTTTTCTGCAACTATTTGGGTTGGTCTAATAAAAGATATCAAATTCACCCAAGGAACCTTGTCTC
C---TTTCAG---TACATTCACTCCTAAGGACTGTGTTACCAGCGCACCACAAACCCAACCTAGGGGAAATACCCCTTTG
GAGGTTGTAGTAACCTGCTTGTGATCATTCATAATGTTTTAGTGATAGGGTCAAGTTTATCACTGCCATGCTGCTTGTGG
AGTGTATTGTGACACAAAGTGCCCAGGTTGTGATTTGGAAGCGTATTTGGCACAATTTGCACTGGTGTGAAAGATCACAC
AAGGAGTAGGCAATGCTGACCCCATAATGGCTATATTGCTTTGTATTCAGTACTTCTGCTTTTTGTAGATTTAAGCTTTC
TGTCCCATTCACTGTAGAAGAGGCAATAATAATTAGAGACACCCCATGGTGTGATTTTTG---------TGTTTGTTTTA

AG---CCTACTACCTGCCAAAAGATGATGAATATTA
>Anolis_GB
TCGCGAATATTACACTTTCATGTGGGCACCTTTACCAAGCAATGCCAACAGCAGTGATGCTGAACTTCAACAGGTGCTTT
TCAAAG---GTCAGTGGGAAGAAGGGGAGGAATTTGATGGAGCAGGTATGCAGTCTTGTCCTA--------TTTGTTATA
GTCTTATCTTGCAAAAAAAAATAAAAATAAAAACACTTGGTAACCTTTGGTT----------------------------
--------------TTGAAGATTAGAAAGAATTGATTA-TAGAAAAAATGTTATCTTATGGTTAGAGGAGGTATGAATAT
TAGTATGTATAACAGTTGCAAGGATTATCAGAAGTCAAATTTCTTTCTTTTTGTTATTTTCTCTTTTTCATGTCTTTTTT
ATTTTTCTGTATATCATTTAGTGTGATGTTGAAATATCTAATTAAAAAAGAAAGGAAAAAACAG-ATCG---TCATAA--
----

----------------GTTGGAAACAACCTTGAAGGTGCTGCAA------------------------------------
----

-----CCAATAAAGTAGCTCCAATGTTTTTGCATGTTTAAGAGGCTAGAAATGAGATTTG--------TTTTGTCCTTTT
AG---CTTACTACTTGCCAAAGGATGATGAATATTA
>Gallus_GB
CCGAGAATATTACACGTTCATGTGGGCTCCTCGGCCCAGTGATGTCCATGGTGATAGTGCTGTGCAGCAAGAGATCCAGT
TCAAAG---GTGGGCTGAG--AGGTTGGGTAACTCACGGTA------------------------------TACATGTGC
AGGTGTACACATAGGCGTGCACACAT-AAATTCCCACACTACTGC-----------------------------------
-------------CACTAATAACTATTATATTCAGAA-TTACAGACTATCTTAGTAATGTGCTTTAGTAAACTTCGAATC
CCGACAACCTAACAGACAAAAAAAATCTTCAAATCTACTGGGTTCTGCAAGCCTGTCTGAATGATTTT------------
--------TTGTTTGTGTTCCAATCACTGTCACAAAGAA---

-------------CTAGTAATGGTGGAGGAACAATATTAAAGTAAGATGTCT----------------------------
----

--GTTTTCTTAGCTTCTGACTTTC
TGT--GCTATATAGTTAAGAGACTC-AAATAATTAGAAGCTGCGTATGACAGTGTTGTTG---------TGTTTGTCTGA
AG---CTTACTACCTGCCCAAAGATGATGAGTATTA

AM05

>Penelope-1
--GTCTTGTATGCAGACCAGCCCAGCCTCTGGCTTCTT
TACTTTTCATTCCATCCAGGAAGAGCACACACCAACTGCTGAAACTTCCTTAGCCTGACGAAGGGTTTTTGAACCCGAAA
GCTTGCTTAATAA-----------TTGTTTTC---------------CAACTATTTAAGTTGGTCTAATAAAAGATATCA
GATTCACCCAAAGAACCTTGTCTG--

>Exons
CCTCAGGAAGCCCGGAATATGCTGCTCCAGAACCCTCAGCTGGCTTATGCCCTGCTGCAAGCCCAGGTGGTGATGAGAAT
TGTTGATCCAGAGATCGCTCTG--

AAAATTCTGCATCGCCAGACCAGCGTTCCAC
>Cpo_scaffold08291 orig_len=84638
CCTCAGGAAGCCCGAAATATGCTGCTCCAAAACCCTCAGCTGGCTTATGCCCTGCTGCAAGCCCAGGTGGTGATGAGGAT
TGTTGATCCAGAGATCGCTCTG---GTATGTATGATTCTCTTCCCCCATTTAGGAGTGATGAGTACACTAATATGGTGAG
GTGGGGCACGTGTGGATTTCAGGAGTAGCTG---TGCATCAGTG------------------------------------
---------------------------------------GGGTACTCGCAGCTTCTTTCTCTCTCTTCTGTCCTTAG---
AAAATTCTGCATCGCCAGACCAGTGTTCCAC
>Tsc
CCTCAGGAAGCCCGGAATATGCTGCTCCAAAACCCTCAGCTGGCTTATGCCCTGCTGCAAGCCCAGGTGGTGATGAGGAT
TGTTGATCCAGAGATCGCTCTG---GTATGTATGATTCTCTTCCTCCATTTGGGAGTGATGAGTACGCTAATCTGGTGAG
GTGGGGCACGTGTGGATTCCAGGAATAGCTG---TGCATCAGTG------------------------------------
---------------------------------------GAGTACTCACAGCTTCTTTCTCTCTCTTCTGTCCTTAG---
AAAATTCTGCATCGCCAGACCAGTGTTCCAC
>Gga_scaffold18345
CCTCAGGAAGCCCGGAATATGCTGCTCCAAAACCCTCAGCTGGCTTATGCCCTGCTGCAAGCCCAGGTGGTGATGAGGAT
TGTTGATCCAGAGATCGCTCTG---GTATGTATGATTCTCTTCCTCCATTTGGGAGTGATGGGTATGCTAATCTGGTGAG
GTGGGGCACGTGTGGATTCCAGGAATAGCTG---TGCATCAGTG------------------------------------
---------------------------------------GAGTACTCACAGCTTCTTTCTCTCTCTTCTGTCCTTAG---
AAAATTCTGCATCGCCAGACCAGTGTTCCAC
>Cya
CCTCAGGAAGCCCGGAATATGCTGCTCCAGAACCCTCAGCTGGCTTATGCCTTGCTGCAAGCCCAGGTGGTGATGAGAAT
TGTTGATCCGGAGATCGCTCTG---GTATGTATAATTTTCTTCCTCCATTTGGGAGTGATGGGTACACTAATCTGGTGAG
GTGGGGCACGTGTTGATTCCAGGAGTTGCTT---TGCATCAGTG------------------------------------
---------------------------------------GGGTACTCACAGCTTCTTTCTCTCTCTTCTGTCCTTAG---
AAAATTCTGCATCGCCAGACCAGCGTTCCAC
>Mni
CCTCAGGAAGCCCGGAATATGCTGCTCCAGAACCCTCAGCTGGCTTATGCCTTGCTGCAAGCCCAGGTGGTGATGAGAAT
TGTTGATCCGGAGATCGCTCTG---GTATGTATAATTCTCTTCCTCCATTTGGGAGTGATGGGTACACTAATCTGGTGAG
GTGGGGCACGTGTTGATTCCAGGAGTTGCTG---TGCATCAGTG------------------------------------
---------------------------------------GGGTACTCACAGCTTCTTTCTCTCTCTTCTGTCCTTAG---
AAAATTCTGCATCGCCAGACCAGCGTTCCAC
>Ppa
CCTCAGGAAGCCCGGAATATGCTGCTCCAGAACCCTCAGCTGGCTTATGCCCTGCTGCAAGCCCAGGTGGTGATGAGAAT

```
TGTTGATCCAGAGATTGCTCTG---GTATGTATAATTCTCTTCCTCCATTTGGGAGTGATGGGTAAACTAATCTGGTGAG
GTGGGGCACGTGTTGATTCCAGGAGTTGCTG---TGCATCAGTG------------------------------------
---------------------------------------GGGTACTCACAGCTTCTTTCTCTCTCTTCTGTCCTTAG---
AAAATTCTGCATCGCCAGACCAGCGTTCCAC
>Asi_533330529
CCTCAGGAAGCCCGGAATATGCTGCTCCAGAACCCTCAGCTGGCTTATGCCCTGCTGCAAGCCCAGGTGGTGATGAGAAT
TGTTGATCCAGAGATCGCTCTG---GTATGTATAATTCTCTTCCTCCATTTGGGAGTGATGGGTACACTAATCTGGTGAG
GTGGGGCACGTGTGGATTCCAGGAGTTGCTG---TGCATCAGTG---CAGCCATTTGAGTTGGTCTAATAAAAGATATCA
AATTCACCCAAGGAACCTTGTCTG--TGCATCAGTG---GGGTACTCACTGCTTCTTTCTCTCTCTTCTGTCCTTAG---
AAAATTCTGCATCGCCAGACCAGCGTTCCAC
>Ami_4925_123350-124900
CCTCAGGAAGCCCGGAATATGCTGCTCCAGAACCCTCAGCTGGCTTATGCCCTGCTGCAAGCCCAGGTGGTGATGAGAAT
TGTTGATCCAGAGATCGCTCTG---GTATGTATAATTCTCTTCCTCCATTTGGGAGTGATGGGTACACTAATCTGGTGAG
GTGGGGCACATGTAGATTCCAGGAGTTGCTG---TGCATCAGTG---CAACCATTTGGGTTGGTCTAATAAAAGATATCA
AATTCACCCAAGGAACCTTGTCTG--TGAATCAGTG---GGGTACTCACCGCTTCTTTCTCTCTCTTCTGTCCTTAG---
AAAATTCTGCATCGCCAGACCAGCGTTCCAC
>Gallus_GB
CCCCAGGAAGCCAGGAACATGCTGCTGCAGAACCCCCAGCTGGCTTATGCTCTGCTACAGGCCCAGGTGGTAATGAGGAT
CGTCGACCCAGAGATCGCACTG---GTTTGT-------CCCTCACTCTCTTG------------------------ACTG
GGGCTGGTAGAACATTGGCAGCAGCCTGCTG---TGAAGTGGAG------------------------------------
---------------------------------------AGCAGAAGATAACTCCTTTCTCCTCCTTCTGCTTCTAG---
AAAATCCTGCATCGTCAGACCAGCGTTCCTC
```

AM06

```
>CR1-7
--------------------------------------------------------------------------------
-----------------------------------------------------------------------------TAG
ATTCATAGA----TTCATAGATGTTAGGGTCGGAAGGGACCTCAATAGATCATCGAGTCCGACCCCCTGCATAGGCAGGA
AAGAGTGCTGGGTTCAGATGACCCCAGCCAGATGCCTATCTAACCTCCTCTTGAAGACCCCCAG----------------
-----------------------------GGTAGGGGAGAGCACCACCTCCCTTGGGAGCCCATTCCAGATTTTGGCCAC
TCTAACTGTGAAGAAGTTCTTCCTAATGTCCAGTCTAAATCTGCTCTCTGCTAGCTTATGGCCATTATTTCTTGTGACCC
CCAGGGGCGCCTTGGTGAGTAGAGCCTCACCAATTCCCTTCTGCGCCCCCATGATGAATTTATAGGCAGC----------
--------------------------------------------------------------------------------
--------------------------------------------------------------------------------
--------------------------------------------------------------------------------
--------------------------------------------------------------------------------
--------------
>Exons
AG------------------------------------------------------------------------------
--------------------------------------------------------------------------------
--------------------------------------------------------------------------------
--------------------------------------------------------------------------------
--------------------------------------------------------------------------------
--------------------------------------------------------------------------------
--------------------------------------------------------------------------------
--------------------------------------------------------------------------------
--------------------------------------------------------------------------------
--------------------------------------------------------------------------------
---------------------------------------------------------------ATTTGGGTGAATAGTCC
GCATACAGCCAGTG
>Cpo_scaffold03069
AG---GTAGGTGCTGATTT-AATTGTTATCGGGAGTTTCTCTGCTGCTCACTGGCTGAGCTG-TTTTATTTCCATCTGTG
TAGCTTTATTTGCTCTGTTACTCAATTTGTTAAGCTACTGGCCAACAGAATGAACCTGCTCAGGC-TTTATTATTAATGC
TGCTGTGCAG----------------------------------------------------------------------
-------------------------------------------------------------------TTGTTGAAGTGCT
CAGAGCATTTTTATGAGTGGTGCTTTTCATGTCAAACCTAGAGAATGAGAAATCATTTTAGAACTGAAAGGCTCACTGTA
GCAGATCTCACTTTGCTTTGGGGACCATTTTCTCAGGCTGGGTTTCCAAACCGTCAGTCTAGTTTGTAA-TTATTCTGAT
TGCTTTGATTACACTTAAGTATATTTC-ATCATAGTTAAACTTGCTCTGATAGGAACTGTTGGGGCTCGGAGGTAGTAAG
GCCTTGTAGCCAGTTCATCCACTTACTCTTGCAGGTACAGGGGACAGGCAGTTGCCAGGGGCATTTTGAGACTCTGCAAG
ATAGATTTGG----CCCTTGGGTGGCACTTCTGGAATCTTGAACCAAGGAAATCTTAGTTGCAAAGAGAGCTTCTTATTA
CAGTTCAGTCCCTTAATTATTCCCTTGGGAAACA--GTGCTTTT-AATCTTCTTAT-----------------TCAAAGA
ATGCCCTGCTTCCATGGGGC----ACAGGTCTGAGTAT-----TGTATTATTTCCTTTAG---ATTTGGGTGAATAGTCC
ACATACAGCCAGTG
>Tsc
AG---GTAGGTGCTGATTT-AATTGTTATTGGGAGTTTCTCTGCTGCTCACTGGCTGAGCTG-TTTTATCTCTATCTCTG
TAGCTTTATTTGCTCTGTTACTTGATTTGTTAAGCTACTGGCCAACAGAATGAACCTGCTCAGGC-TTTATTATTAATGC
TGCTGTGCAG----------------------------------------------------------------------
-------------------------------------------------------------------TTGTTGAAGTGCT
CAGAGCATTTTTATGAGTGGTGCTTTTCATGTCAAACCCAGAGAATGAGAAATCATTTTAGAACTGAAAGGCTCACTGTA
GCAGATCTCACTTTGCTTTGGGGACCATTTTCTCAGGCTGGGTTTCCAAACTGTCAGTCTAGTTTGCAA-TTATTCTGAT
TGCTTTGATTACACTTCAGTACATTTC-ATCATAGTTAAACTTGCTCTGATAGGAACTGTTGGGGTTCACAGGCAGTAAG
GCCTTGCAACCAGTTCATCCACTTACTCTTGCAGGTACACGGGACAGGCAGTTGCCAGGGGCATTTTGAGACTCTGCAAG
ATAGATTTGG----CCCTTGGGTGGCACTTCTGGAATCTTGAACCAAGGAAATCTTAGTTGCAAAGAGAGCTTCTTATTA
CAGTTCAGTCCCTTAATTATTCCCTTGGGAAACA--GTGCTTTT-AATCTTCTTAT-----------------TCAAAGA
ATGCCCTGCTTCCACGGGGC----ACAGGTCTGAGTAT-----TTTATTATTTCCTTTAG---ATTTGGGTGAATAGTCC
ACATACAGCCAGTG
>Gga_scaffold19764
```

AG---GTAGGTGCTGATTT-AA---TTATTGGGAGTTTCTCTGCTGCTCACTGGCTGAGCTG-TTTTATCTCTATCTCTG
TAGCTTTATTTGCTCTGTTACTTGATTTGTTAAGCTACTGGCCAACAGAATGAACCTGCTCAGGC-TTTATTATTAATGC
TGCTGTGCAG--
---TTGTTGAAGTGCT
CAGAGCATTTTTATGAGTGGTGCTTTTCATGTCAAACCTAGAGAATGAGAAATCATTTTAGAACTGAAAGGCTCACTGTA
GCAGATCTCACTTTGCTTTGGGGACCATTTTCTCCGGCTGGGTTTCCAAACCATCAGTCTAGTTTGCAA-TTATTCTGAT
TGCTTTGATTACACTTCAGTACATTTC-ATCATAGTTAAACTTGCTCTGATAGGAACTGTTGGGGTTCACAGGCAGTAAG
GCCTTGCAACCAGTTCATCCACTTACTCTTGCAGGTACATGGGACAGGCAGTTGCCAGGGGCATTTTGAGACTCTGCAAG
ATAGATTTGG----CCCTTGGGTGGCACTTCTGGAATCTTGAACCAAGGAAATCTTAGTTGCAAAGAGAGCTTCTTATTA
CAGTTCAGTCCCTTAATTATTCCCTTGGGAAACA--GTGCTTTT-AATCTTCTTAT-----------------TCAAAGA
ATGCCCTGCTTCCATGGGGC----ACAGGTCTGAGTAT-----TTTATTATTTCCTTTAG---ATTTGGGTGAATAGTCC
ACATACAGCCAGTG
>Cya
AG---GTAAGTGCTGATTT-AATTGTTATTGAGAGTTTGTCTGCTGCTCACTGGCTGAGCTG-TTTTATCTCTATCTCTG
TAGCTTTATTTGCTCTGTTACTTGATTTGTTAAGCTGCCGGCCAACAGAATGAACCTGCTCAGGC-TTTATTATTAATGC
TGCTGTGCAG---TTCATAGATATTAGGACTGGAAGGGACCTCAGTAGATCATCGAGTCCGACCCCCTGCATAGGCAGGA
AAGAGTGCTGGGTTCAGATGACCCCAGCCAGATGCCCATCTAACCTCCTCTTGAAGACCCTCAG---TTGTTGAAGTGCT
CAGACTATTTTTCTGAGTGGTGCTTTTCATGTCAAACCTAGAGAAAGAGAAATTGTTTTAGAATTGAAAGGCTCACTGTG
GCAGATCTCACTTTGCTTTGGGGACCATTTTCTCAGGCTGGGTTTCCAAACAGTCAGTCTAGTTTGCAG-TTATTCTGAT
TGCTTTGATTACACGTAAGTATATTTC-ATCATAGTTAAACTTGCTCTGATAGGAACTGTT-GGGTTCGCAGGCAGTAAG
GCCTTGCAGCCACTTCATCCACTTACTCATGCAGGCAC--GGAACAGGCAGCTGCCAGGGGCATTTTGAGA--CTGCAAG
ATAGATTTGG----CCCTTGGGTGGCACTTCTGGAATTCTGAACCAAGGAAATCTTAGTTGTAAAGAGAACTTCTTATTA
CAGTTTAATCCCTCAATGATTTTCTTGGGAAACACGGTGCTTTT-AATCCTCTTAT-----------------TCAAAGA
GTGCCCTACTTCCACTGGGC----ACAGGTTTCAGTAT-----TGTATTATTTCCTTTAG---ATTTGGGTGAATAGTCC
GCATACAGCCAGTG
>Mni
AG---GTAAGTGCTGATTTAAATTGTTATTGAGAGTTTGTCTGCTGCTCACTGGCTGAGCTG-TTTTATCTCTATCTCTG
TAGCTTTATTTGCTCTGTTACTTGATTTGTTAAGCTGCCGGCCAACAGAATGAACCTGCTCAGGC-TTTATTATTAATGC
TGCTCTGCAG---TTCATAGATATTAGGGCTGGAAGGGACCTCAGTAGATCATCGAGTCCGACCCCCTGCATAGGCAGGA
AAGAGTGCTGGGTTCAGATGACCCCAGCCAGATGCCCATCTAACCTCCTCTTGAAGACCCTCAG---TTGTTGAAGTGCT
CAGACTATTTTTCTGAGTGGTGCTTTTCATGTCAAACCTAGAGAAAGAGAAATTGTTTTAGAACTGAAAGGCTCACTGTG
GCAGATCTCACTTTGCTTTGGGGACCATTTTCTCAGGCTGGGTTTCCAAACAGTCAGTCTAGTTTGCAG-TTATTCTGAT
TGCTTTGATTACACGTAAGTATATTTC-ATCATAGTTAAACTTGCTCTGATAGGAACTGTT-GGGTTCGCAGGCAGTAAG
GCCTTGCAGCCACTTCATCCACTTACTCATGCAGGCACAGGGGACAGGCAGCTGCCAGGGGCATTTTGAGA--CTGCAAG
ATAGATTTGG----CCCTTGGGTGGCACTTCTGGAATCCTGAACCAAGGAAATCTTAGTTGCAAAGAGAACTTCTTATTA
CAGTTTAATCCCTCAATGATTTTCTTGGGAAACACGGTGCTTTT-AATCCTCTTAT-----------------TCAAAGA
GTGCCCTACTTCCACCGTGC----ACAGGTTTCAGTAT-----TGTATTATTTCCTTTAG---ATTTGGGTGAATAGTCC
GCATACAGCCAGTG
>Ppa
AG---GTAAGTGCTGATTTAAATTGTTATTGAGAGTTTGTCTGCTGCTCACTGGCTGAGCTGTTTTTATCTCTATCTCTG
TAGCTTTATTTGCTCTGTTACTTGATTTGTTAAGCTGCTGGCCAACAGAATGAACCTGCTCAGGCTTTTATTATTAATGC
TGCTGTGCAG---TTCATAGATGTTAGGGCTGGAAGGGACCTCAGTAGATCATCGAGTCCGACCCCCTGCATAGGCAGGA
AAGAGTGCTGGGTTTAGATGACATCATCCAAATGCCCATCTAACCTCCTCTTGAAGCCCTTGAG---TTGTTGAAGTGCT
GATAGCATTTTTCTGAGTGAGACTTTTCATGTCAAACATATAGAAAGAGAAATTGTTTAAGAACTGAAAGGCTCGTTGTG
GCAGATCTCGGTTTGTTTTGGGGACCATTTTTTCAGGGAGGGTTTCCAAACAGTCAGTAGAAATTGCAG-TTATTCTGAG
TATCTTGATTACATGTAAGTATACCTC-ATCATAGTTAAACTTGCTCTGATCGGATGTGTT-GGGTTCGCAGGCAGTAAG
GCCGGGCAGCCACTTCATCCACTTACTCCTGCAGGTACAGGGGACAGTCAGCTCCCAGGGGCATAGCGAGA--GTGCGAG
ATAAATTTGG----CCCCAGGGTGGCCCTTGATGACTCTTGAACCAAGGAAATCTTTGTGGAAAAGAGAACTTCTGAGTT
CAGTTTAATCCCGGAATGATTTTCCATCGAACCACGGAACAGTTAATGGAACTTAT-----------------TCAGAGA
GTGCCCTACGTCCGCCCGGC----ACAGGTTACCGCAT-----TGTATTACTTCCA-------------------------
----
>Asi_533250755
AG---GTAAGTGCTGATTT-AATTGTTATTGACAGTTTGTCTGCTGCTCACTGGCTGAGCTG-TTTTATCTCTATCTCTG
TAGCTTTATTTGCTCTGTTACTTGATTTGTTAAGCTGCTGGCCAACAGAATGAACCTGATCAGGC-TTTATTATTAATGC
TGCTGTGCAG---TTCATAGATGTTAGGGCTGAAAGGGACCTCAGTAGATCATTGAGTCCGACCCCCTGCATAGGTAGGA
AAGAGGGCTGGGTTCAGATGACCCCAGCCAGATGCCCATCTAACCTCCTCTTGAAGACCCTCAG---TTGTTGAAGTGCT
CAGAGCATTTTTCTGAGTGATGCTTTTCATGTCAAACCTAGAGAAAGAGAAATCGTTTTAGAACTGAAAGGCTCACTGTA
GCAGATGTCACTTTGCTTTGGGGACCATTTTCTCAGGCTGGGTTTCCAAACCCTCAGTCTAGTTTGCAA-TTATTCTGAT
TGCTTTGATTACACTTAAGTATATTTC-ATCATAGTTAAACTTGCTCTGATAGGAACTGTT-GGGGTTGCAGGCAGTAAG
GCCTTGCAGCCAGTTCATCCACTTACTCGTGCAGGTACAGGGGACAGGCAGCTGCCAGGGGCATTTTGAGA--CTGCAAG
ATAGATTTGG----CCCTTGGGTGGCACTTCTGGAATCCTGAACCAAGGAAATCTTAGTTGCAAAGAGAACTTCTTATTA
CAGTTCAATCCCTCAATGATTTCCTTGGGAAACACGGTGCTTTT-AATCCTCTTAT-----------------TCAAAGA
GTGCCCTACTTCCACCGGGC----ACAGGTTTCAGTAT-----TGTATTATTTCCTTTAG---ATTTGGGTGAATAGTCC
GCATACAGCCAGTG
>Ami_3948_406600-408150
AG---GTAAGTGCTGATTT-AATTGTTATTGAGAGTTTGTCTGCTGCTCACTGGCTGAGCTG-TTTTATCTCTATCTCTG
TAGCTTTATTTGCTCTGTTACTTGATTTGTTAGGCTGCTGGCCAACAGAATGAACCTGCTCAGGC-TTTGTTATTAATGC
TGCTGTGCAG---TTCATAGATGTTAGGGCTGGAAGGGACCTCAATAGATCATCGAGTCCGACCCCCTGCATAGGCAGAA
AAGAGTGCTGGGTTCAGATGACCCCAGCCAGATGCCCATCTAACCTCCTCTTGAAGACCCTCAG---TTGTTGAAGTGCT
CAGAGCATTTTTCTGAGTGATGCTTTTCATGTCAAACCTAGAGAAAGAGAAATCATTTTAGAACTGAAAGGCTCACTGTA
GCAGATCCCACTTTGCTTTGGGGACCATTTTCTCAGGCTGGGTTTCCAAACCGTCAGTCTAGTTTGCAA-TTATTCTGAT
TGCTTTGATTACACTTAAGTATATTTC~ATCATAGTTAAACTTGCTCTGATAGGAACTGTT-GGGGTCGCAGGCAGTAAG
GCCTTGCAGCCAGTTCATCCACTTACTCGTGCAGGTACAGGGGACAGGCAGCTGCCAGGGGCATTTTGAGA--CTGCAAG
ATAGATTTGG----CCCTTGGGTGGCACTTCTGGAATCTTGAACCAAGGAAATCGTAGTTGCAAAGAGAA---CTTATTA
CAGTTCAATCCCTCAATGATTTCCTTGGGAAACACGGTGCTTTT-AATCCTCTTAT-----------------TCAAAGA
GTGCCCTACTTCCACCGGGC----ACAGGTTTCAGTAT-----TGTATTATTTCCTTTAG---ATTTGGGTGAATAGTCC
GCATACAGCCAGTG
>Gallus_GB

AG---GTAAGCACAGATTTTACTTCTTGG-GTAGCTTTTCCTGCTGTCTACTTCTTGATCTG-TATTCTCTCTTTGATTG
TTGAAGTTAATGGTAAGCAGCAATACCTGTGGGCCTTTT------TAAGTGGAATTAGTACTGG----------------
----

---------------AGTGTTACTGCTCATAT--
------------------------------------GACTGGCTTCCAAA--------TCTAATTTATATTTTGCTCTGC
TTATCTCAATAACACACCACTATATTCA-ACCTGTGTTGAAGCTATAAAAAT----------------------------
--CCACGGGTTTACCATATCCTCATTTCAATGTTATTAGCGGAGAATAG------------------------CTGCAAG
ACCAGTTT--------------TAGGATGTTTGGCAGTTTGAGCTA-------------TATGTAAAAAATCTTTGGACC
TGG---AAGGCCTTAGCTATGTTCATACTTAACAAGATCCTCCT-TGTTAGTTTATCTGCTTTATTTAAACACTCAAAAG
ATGCCCTTTTGATGTAATGT----GTGGATCTT----------TTTTTCCACTTCTGCAG---ATTTGGGTGAACAGCCC
ACACACAGCCAGTG
>Anolis_GB
AG---GTATTTGCTGCCTT-TGCTCTTGGTTTATATCTGTGTAT----------CTGGTC---------CTCTACTGCAG
TTACCATTTGTTTCAAAACAAGAAATCTCTGGGGTCATAGGAGAGCTAATGGGACTTGTTTTGG----------------
----

------ATCTTACATTTAGTAGATAAGTTTTAGAAAAATATAGCCACTTTGAATAAAGATGCATTTTAAGG---------
CTCTACTTTATTCCTCTTATTGATAAATTAGCATTCAAAGAGATGCATCTGTTTTCTATGTTA----GAATGCATT-ATT
CTCCCTTCATTCTGTTCCTGTATACGGTGGATTTAAAAAAAAACCAGCCTCT-G--------------------------
---TTTGATCTTCTTTAAACATTACT--TTGCAAGT
CTAATGTGGGAGCCAGTGGTGTGTAGTGGTTTAAGTGTTGGACTA--------GCACTCTGGAGGAGAA----------G
GGTTTAAATCCCCATTGG-AATTCAT-------------TTGCT-GAACATTATATGGGTTCATCTTAGGATCTCCAGAA
ACAATAACTCAAAGTCATGCAGCAATGAGTCTAATGGCTTGCGTTTAATCACTTTCACAG---ATCTGGGTGAATAGTCC
TCACACTGCCAGTG

AM07

>CR1-7

---TAGATTCATAGATTCATAGATGTTAGGGTCGGA
AGGGACCTCAATAGATCATCGAGTCCGACCCCCT----------------GCATAGGCAGGAAAGAGTGCTGGGTTCAGA
TGACCC--

>Exons_rc
AGGC--

---CTGTTGATCATGT
>Cpo_scaffold13841
AGGC---CTGAATTAGGAAAATAAAA---------ACACACAC--------------AAAAAAGACCAAAAATCCCTGCA
TTTTAATTGTTTGTCAAATACTGATCTTT---
-------------------------------------AACTT---GTGCCTTTAAAAACAATTATTCCTAATTAAA---C
TGCTGAAGGCAGCCCACTTTGGAAAACCTTTTGTAAATCACAATTTCAGTTCTTACTGCCCTTTCATTTTGAATACTATA
TTAACAAAAATAATTTAATTTTGATAAGAAATCCAACTAAACAGAACGTGATTCCAGTCTTGTGTGTAATATTA------
-TTGACCAATAA-CTAATGACATCAGAA-ACAGTGCTTAAGTCTTAAAA----TAC---CTGTTGATCATGT
>Tsc
AGGC---CTGAATTAGGAAAATAAAA-----------ACACAC--------------AAAAAAGACCAAAAATCCCTGCA
TTTTAATTGTTTGTCAAATACTGATCTTT---
-------------------------------------AACTT---GTGCCTTTAAAAACAATTATTCCTAATTAAA---C
TGCTGAAGGCAGCCCACTTTGGAAAACCTTTTGTAAATCACAATTTCAGTTCTTACTGCCCTTTCATTTTGAATACTATA
TTAACAAAAATAATTTAATTTTGATAAGAAATCCAATTAAACAGAACGTGATTCCAGTCTTGTGTGTAATATTAAAGAGA
TTTGACAAATAA-CTAATGACATCAGAA-ACAGTGCTTAAGTCTTAAAA----TAC---CTGTTGATCATGT
>Gga_scaffold19390
AGGC---CTGAATTAGGAAAATAAAA---------ACACACAC--------------AAAAAAGACCAAAAATCCCTGCA
TTTTAATTGTTTGTCAAATACCGATCTTT---
-------------------------------------AACTT---GTGCCTTTAAAAACAATTATTCCTAATTAAA---C
TGCTGAAGGCAGCCCACTTTGGAAAACCTTTTGTAAATCACAATTTCAGTTCTTACTGCCCTTTCATTCTGAATACTATA
TTAACAAAAATAATTTAATTTTGATAAGAAATCCAATTAAACAGAACGTGATTCCAGTCTTGTGTGTAATATTAACGAGA
TTTGACAAATAA-CTAATGACATCAGAA-ACAGTGCTTAAGTCTTAAAA----TAC---CTGTTGATCATGT
>Cya
AGGC---CCGAAAGGGGGGGGGGGGGGGCCCGGCAACCCAAAA-----------AAAAAAAAAGACCAAAAATCCCTGCA
TTTTAAT--TTTGTCAAATACTGATCTTT---
-------------------------------------AACTT---GTGCCTTTAAAAACAATTATTCATAATTAAA---C
TGCTGAAGGCAGCCCACTTTGGAAAACCTTTTGTAAATCACAATTTAAATTCTTACTGCCCTTTCATTTTGAATACTATA
TTAACAAAAGTAATTTAATTTTGGTAAGAAATCCAACTAAACAGAACGTGATTCTAGTCTTGTGTGTAATATTAAAGAGT
TTTGACCAATAA-CTAATGACACCAGAA-ACAGGGCTAA---------------------------------
>Mni
AGGC---CTGAATGGGGGGGGGGGATGACCCGACAA--CACAA-----------AAAAAAAAAGACCAAAAATCCCTGCA
TTTTAAT--TTTGTCAAATACTGATCTTT---
-------------------------------------AACTT---GTGCCTTTAAAAACAATTATTCATAATTAAA---C
TGCTGAAGGCAGCCCACTTTGGAAAACCTTTTGTAAATCACAATTTAAATTCTTACTGCCCTTTCATTTTGAATACTATA
TTAACAAAAGTAATTTAATTTTGGTAAGAAATCCAATTAAACAGAACGTGATTCTAGTCTTGTGTGTAATATTAAAGAGT
TTTGACCAATAA-CTAATGACACCAGAA-ACAGTGCTTAAGTCTTAAAA----TAC---CTGTTGATCATGT
>Ppa

```
AGGC---CTGAATAAGTGGGGGGACG---------------AC-----------ACAAAAAAAGACCAAAAATCCCTGCA
TTTTAAT--TTTGTCAAATACTGATCTTT---------------------------------------------------
-------------------------------------AACTT---GTGCCTTTAAAAACAATTATTCATAATTAAA---C
TGCTGAAGGCAGCCCACTTTGGAAAACCTTTTGTAAATCACAATTTAAATTCTTACTGCCCTTTCATTTTGAATACTATA
TTAACAAAAGTAATTTAATTTTGGTAAGAAATCCAATTAAACAGAACGTGATTCTAGTCTTGTGTGTAATATTAAAGAGA
TTTGACCAATAA-CTAATGACACCAGAA-ACAGTGCTTAAGTCTTAAAA----TAC---CTGTTGATCATGT
>Asi_533327014
AGGC---CTGAATTAGGGGAGAAAAA---AAAAAAACACACAC-------------AAAAAAAGACCAAAAATCCCTGCA
TTTTAAT--TTTGTCAAATACTGATCTTT---AACTTA---GATTCACAGATGTTAGGTTTCATAGATGTTAGGGTTGAA
AGGGACCTCAATAGATCATCGAGTCCGACCCCCC---AACTT---GTGCCTTTAAAAACAATTATTCCTAATTAAA---C
TGCTGAAGGCAGCCCACTTTGGAAAACCTTTTGTAAATCACAATTTAAATTCTTACTGCCCTTTCATTTTGAATACTATA
TTAA--AAAGTAATTTAATTTTGATAAGAAATCCAATTAAACAGAACGTGATTCTAGTCGTGTGTGTAAT---AAAGAGA
TTTGACCAATAA-CTAATGACACCAGAA-ACAGTGCTTAAGTCTTAAAA----TAC---CTGTTGATCATGT
>Ami_5323_182200-183750
AGGC---CTGAATTAGGGGGGGAAAA-----AAAAAACCACAC--------------AAAAAAGACCAAAAATCCCTGCA
TTTTAAT--TTTGTCAAATACTGATCTTT---AACTTA---GATTCATAGATGTTAGGTTTCATAGATGTTAGGGTCGGA
AGGGACCTCAATAGATCATCGAGTCCGACCCCTC---AACTT---GTGCCTTTAAAAACAATTATTCCTAATTAAA---C
TGCTGGAGGCAGCTCACTTTGGAAAACCTTTTGTAAATCACAATTTAAATTCTTACTGCCCTTTCATTTTGAATACTATA
TTAACAAAAGTAATTTAATTTTGATAAGAAATCCAATTAAACAGAATGTGATTCTAGTCTTGTGTGTAATATTAAAGAGA
TTTGACCAATAA-CTAATGACACCAGAA-ACAGTGCTTAAGTCTTAAAA----TAC---CTGTTGATCATGT
>Gallus_GB
AGGC---CTATATTCAAAAACAAAACAAAGCCATGCATCTCAACTGTGCTTCTTAAAAAAAAAATTAAAAAAATGAGGAA
T---CACTGTTCCTTTTTAA-TGAAAGG----------------------------------------------------
------------------------------------------------TAAAAAAAAATACGCCTTTTTCAAGGCC---C
TGATAAAGGCT--CCACTTTCAAAAAACCC--ACAAAGCCCGATATGAG----------CATTTCTTTCAAACTCTAACC
CAATAAGAAGTATTTCA---------AGAAAACTGGTGAATCAGA--GAAGCACAAATCCTGCTTCGACTATCAAGGTAT
TTTCACAAATAATCTACACGCACTGGAAAAAAGGCATTAAGTGTGAAAG----TAC---CTGTTGATCATGT
>Anolis_GB
AGGT---CTAAGAAAACAGTAGGACATAATATTTCAATGTTAC---------------ATGAAATCCAAAAA-CTGTCAA
TTTTCAGTGTTTCTATC---------------------------------------------------------------
-------------------------------------TAGAA---AAGATGGAATAAGTTTGAACCACAAATTACAGCAC
TGCTTCAGGAAAAAAACT-------------------------------------------ATACTTCTTAGAAATCCTT
AAAGGTGAAATATTTTATATTTCCAAAAATTGTACAAGAGAGACATTTTGAATACCACAAGGGATGAAATGGATAGCAAA
TATTTATAATTTACTATAAACAGAAAAAAATGAATCATGGCCTTTGAAGAACTAAC---CTGTTAATCATGT
```

AM09

```
>Gypsy-38
--------------------------------------------------------------------------------
--------------------------------------------------------------------------------
--------------------------------------------------------------------------------
--------------------------------------------------------------------------------
--------------------------------------------------------------------------------
--------------------------------------------------------------------------------
--------------------------------------------------------------------------------
--------------------------------------------------------------------------------
--------------------------------------------------------------------------------
--------------------------------------------------------------------------------
----------------------------------------------------------TTCATAGATTCATAGATGTTAG
GGTTGGAAGGGACCTCAATAGATCATCGAGTCCGACCCCCTGCATAAGCAGGAAAGAGTGCTGGGTCTAGATGACCCCAG
CTAGATACTCATCTAA---CCTCTTGAA----------------------------------------------------
------------------------------------------------------------
>Exons
AAGAATCTGTTCTTACACCAATGCCAGCCTATGCCAGGCATACAGGACAAGTGGGGACCAAACTTTACATGAGCCCAGAA
CAG-----------------------------------------------------------------------------
--------------------------------------------------------------------------------
--------------------------------------------------------------------------------
--------------------------------------------------------------------------------
--------------------------------------------------------------------------------
--------------------------------------------------------------------------------
--------------------------------------------------------------------------------
--------------------------------------------------------------------------------
--------------------------------------------------------------------------------
--------------------------------------------------------------------------------
--------------------------------------------------------------------------------
--------------------------------------------------------------------------------
--------------------ATTTATGGGAACACATATTCACACAAAGTGGACATCTTCT
>Cpo_scaffold07263
AAGAATCTGTTCTTACACCAATGCCAGCCTATGCCAGGCACACAGGACAAGTGGGGACTAAACTTTACATGAGCCCAGAA
CAG---GTGAGGCTTTTCTCAAACT--------TCACACATGGAAGTAACTAAGA-------------------------
----------AAAGCACTTGTAA-TTGGGGCTTCACTGTCTTCCTGGCCATGGACATGTAGAAATACAGTGTCTTCTGGG
TGATCGCAGAGAATTCTTAGTAACGATAGATAATAGGTTGCCCATTGAATGTACGGTGA---CTGTGATAGCTGCCCTCA
TGTAATAGCAGCACCCCTGAATATTGCTTCAACATGTTGAAAAATAACTTCGTGTTGTAACGACCACATACATATTTCAG
AAGAGAGGGATTCAAAAAAGATTTCTCTTAAAACAGGAGCGTCAAACACAACCGGCCCTATGTACCATCTGAGAAAGTAG
GTTGTTGCATATAAAAGCTCATGTCTTTCTGAACTAGTAATTCTATTAAGGTGCCATGCTGTCCTGCCTTTTGCCTGTTC
TTAGTTTGGAATATTTTAATTTGATCTGATAAGA------------ATTAGTCTTGTGCTTCAGATGGGGCCGTC-----
--AGTCTG-AATTGAATTCTGATACTGCTAGTTTCAGCACTCTTTAATTATTGTAAAAATTAGGTTTGTGAAAATAATGT
ACCTGAGTAGAGATGATTATTTTGATCCTGTCTTAAAACAGGACAGGAAGGTAAAACCTGAGTAGTATGATAGACTCCAT
```

CAGCCCTTCACAGAATTAATCATTTGGATGTGTTTAACTGACTGGTGGTGTATGA-------------------------
----
-------------------------------TTTTTATGTGTGTTGCTGAATTCTGTGAGCTGTGCCATTAACATGAACT
TGATTCTGGCTTTCCAG---ATTTATGGGAACACATATTCACACAAAGTGGATATCTTCT
>Gga_scaffold10078
AAGAATCTGTTCTTACACCAATGCCAGCCTATGCCAGGCACACAGGACAAGTGGGGACTAAACTTTACATGAGCCCAGAA
CAG---GTGAGGCTTTTCTTAAACT--------TAACACATGGAAGTAACTAAGA-------------------------
----------AAAGTACTTGTAA-TTGGGTCTTCACTGTCTTCCTGGCCATGGATATGTAGAAATACAGTGTCTTCTGGG
TGATCGCAGAGAATTCTTAGTAACAATAGATAATAGGTTGCCCATTGAATGTACGGTTGACTTTGTGATAGCTGCACTTA
TGTAATAGCGGCACCCCTGAATATTGCTTCAACATGTTGAAAAATAACTTCGTGTTGTAATGACCACATACATATTTCAG
AAGAGACGGATTCAAAAAACATTTCTCTTAAAAAAGGAGTGTCAAACACAACCGGCCCTATGTACCAACTGACAAAGTAG
GTTGTCGCATATAAAAGCTCATGTCTTTCTGAACTAGTAATTCTCTTAAGATGCCATGCTGTCCTGCCTTTAGCCTGTTC
TTAGTTTGGAATATTTTAATTTGATCTGATAAGA------------ATTAGTCACGTGCTTCAGATGGGGCCGTC-----
--AGTC---AAATGAATTCTGATACAGCTATTTTCAGCACTCTTTAATTATTGTAAAAATTAGCTTTGTGAAAATAATGT
ACCTGAGTAGAGATGATTATTTTGATCATCTCTTAAAACAGGACAGGAATGTAAAACCTGAGTAGTATGATAGACTCCAT
CAGCCCTTCACAGAATTAATCATTTGGGTGTGTTTAACTGACTGGTGGTGTATGA---T---------------------
----
-------------------------------TTTTTGTGTGTGTTGCTGAATTCTGTGAGCTATGTCATTAACATGAACT
TGATTCTGGTTTTCCAG---ATTTATGGGAACACATATTCACACAAAGTGGATATCTTCT
>Asi_533464261
AAGAATCTGTTCTTACACCAATGCCAGCCTATGCCAGGCATACAGGACAAGTGGGGACCAAACTTTACATGAGCCCAGAA
CAG---GTGAGGCTTTTTTCAAGCT--------TAACACATGGAAGTAACTAAGA-------------------------
----------AAAGTACTTGTAACTTGGGGCTTCACTTTCTTCCTGGCCATGGACATGTAGAAATACAGTGTCTTCTGGG
TGATCAC--AGAATTCTTAGTAATGAAAGAAGATAGGTTGCCCGTTGAATGTATGGTTGACTTTGTGATAGCTGCACTCG
TGTAATA---

--GCCACGCTGTCCTGCCTTTTGCCTGTTC
TTAGTTTGGAAGATTTTAATTTGATCTGCTAAGA------------ATTAGTCACGTGCTTCAGATGGGGCCATC-----
--AGTCTG-AATTGAATTTTGATACAGGTATTTTCAGCACTTTTTAATTATTGTAAAAATTAGGTTTGTGAAAATAATGT
ACCTGAGTAGAGATGATTATTTTGATCCTCTCTTAAAACAGGACAGGAAGGTAAAACCTGAGTAGTATGATAGACTCCAT
CAGCCCTTCACAAAATTAATCATGTGGGTGTGTTTAACTGACTAGTGTTGTATGA-------------------------
----
-------------------------------TTTTTATGTGCGTTGCTGAATTCTGTCAGCTATGCCATTAACGTGAACC
TGATTCTGGTTTTCCAG---ATTTATGGGAACACATATTCACACAAAGTGGATATCTTCT
>Ami_2809_177950-179500
AAGAATCTGTTCTTACACCAATGCCAGCCTATGCCAGGCATACAGGACAAGTGGGGACCAAACTTTACATGAGCCCAGAA
CAG---GTGAGGCTTTT-TCAAGCT--------TAACACATGGAAGTAACTAAGA-------------------------
----------AAAGTACTTGTAACTTGGGGCTTCACTTTCTTCCTGGCCATGGACATGTAGAAAT--AGTGTCTTCTGGG
TGATCAC--AGAATTCTTAGTAATGAAAGAAGATAGGTTGCCCGTTGAATGTATGGTTGACTTTGTGATGGCTGCACTCG
TGTAATA---

--GCCACGCTGTCCTGCCTTTTGCCTGTTC
TTAGTTTGGAAGATTTTAATTTGATCTGTTAAGA------------ATTAGTCACGTGCTTCAGATGGGGCCATC-----
--AGTCTG-AATTGAATTTTGATACAGGTATTTTCAGCACTTTTTAATTATTGTAAAAATTAGGTTTGTGAAAATAATGT
ACCTGAGTAGAGATGATTATTTTGATCCTCTCTTTAAACAGGACAGGAAGGTAAAACCTGAGTAGTAT-ATAGACTCCAT
CAGCCCTTCACAGAATTAATCATGTGGGTGTGTTTAACTGACTACTGTTGTATGA---TTCATAGATTCATAGATGTTAG
GGTCGGAAGGGACCTCACTAGATCATCGAGTCCAACCCCCTGCATAAGCAGGAAAGAGTGCTGGGTCTAGATGACCCCAG
CTAGATACTCATCTAACCTCCTCTTGAA---TTTTTATGTGCGTTTCTGAATTCTGTGAGCTATGCCATTAACGTGAACC
TGATTCTGGTTTTCCAG---ATTTATGGGAACACATATTCACACAAAGTGGACATCTTCT
>Gallus_GB
AGGAATTGGTACTTACCCCAATGCCAGCTTACGCCAGACACACAGGACAAGTAGGGACCAAACTCTACATGAGTCCAGAA
CAG---GTTTGTGTTCTCTAAAGCTATTTCCAGCACCACCCGGAAGACACTTGAATTCCTGGGAGTTCAAGGATCAAGTA
GGCTTTCTAGTAAACTGTTGCAA-TCAGACTTACATTATTTCTCATCAAAGTAGAATTTTGAGCTAAACTGAATTCCAAA
ATA------AATATTTTTAACATAAAATAGCAAAAACAAACACAAAAAAAGGGT--------TAATGATGTGTACTTTGG
TGCATTG---

---ATTTATCTTGCTC
TTAGTGTATTAGGCTGTGTAATATTCAAGTCAGAAAAGAATGCAGTATTGTACAGGAGGTTTAAATTGAAACGTTGTGGT
TAGGTCTGCAAAGGAGAGCTGATGATAGGAGCTTGTTTACTCATACTCACTTGCTAAAGTTTGACGTATCAAAAGGCTGT
TAC---AGTTAGCACCTGTTCTGTGC--------TTTT
CTGCTCTGCACAGAGCTAATCACTTCCATGTTTGGGA--------AGTTGTTTGA-------------------------
----
-------------------------------TTATACTGTATG----TAATTCCAGTGACTAGACTGGCTGACCTGACCT
GAATTCTGAATTTTCAG---ATCTGTGGGAACACCTATTCTCACAAAGTGGATATATTTT

AM15

>CR1-2C
TGCGTCCAGTTTTGGGCTCCACAATTCAAAAAGGATGTGGAGAAGCTTGAGAGAGTCCAGAGAAGAGCCACGCGCATGAT
CAGAGGTCAGGAAAACAGACCTTACGATGACAGGCTGAGAGCCATGGGGCTCTTTAGCC-TGGAAAAGCGCAGGCTCAG-
--------GG------GTGATCTGATGGCCACCTATAAGTTTATCAGGGGTGACCACCAGGATCTGGGGGAACGTTTGTT
CACCAGAGCGCCCCAAGGGATGACGAGGTCGAACGGTCATAAACTACTGCAAGACCGTTTCAGGCTGGACATAAGGAAGA
ATTTCTTTACTGTCCGAGCCCCCAAGGTCTGGAACAGCCTGCCATCGGAGGTGGTTCAAGCACCTACATTGAACACCTTC
AAGAGCAAATTGGATGCTTATCTTGCTGGGATCCTATGACCCCAGCTGACTTCCTGCCCTTCGGGCGGGGGGCTGGACTC
GATGATCTTCCGAGGTCCCTTCCAGCCCTAATGTCTATGAAATCTAT------GAAA-----------------------
----

>Exons
AGATTGACCTCGAGGACCTGAA--

--GAAACACACCGTGTACTACGGC
GGCTTCCACGGCAGTCACAGGGTCATCATCTGGCTTTGGGACATCCTAGCCAAT
>Cpo_scaffold04434
AGATTGACCTTGAGGACCTGAA---GTGAGCGGGTTGTACCCT----------------AGGCCGGTCCCTCATTTCTGT
AATGCTCTTAGCACAGGAAACTAACGCTTTCCAACCCTTCTGCTTCTTCCAATCAAAAG-TCAAATGACAGCCCGGGAGT
GGAAGGTTGG--

---GAAATAGC-ATGGCCTGTAACATGGGA
GTGGGCAAATTGACC-----TCAGGGAACAGCTGTACCCAGGAGACAAGTACCAAATATCTGTAGCTCCTTCAATGCTCT
GGCAAGCCTGCCGGACATGGTGGAT------TGCAGAGAGCAGGAGCGGAAGCCTCTCTTTATAACTGAGGGGGTGGGGA
GGTCTCTGGGGTGGATTGGAGAGCTGCAGC--------------------------------CACCTATCCGCAGGTGAG
CAGGATGCTGTGACCTTGTCTGATTATGAGCTTTTTGTG----TCCTGTCCTCAG---GAAACACACAGTGTACTATGGT
GGCTTCCACGGCAGTCACAGGGTCATCATCTGGCTTTGGGACATCCTAGCCAAT
>Gga_scaffold16932
AGATTGACCTCGAGGACCTGAA---GTGAGTGGGCTGCACCTT----------------AGGCTGGTCCCTCATTTCTGT
AATGCTCTTAGCACAGGAAACTAACGCTTTCCAACCCTTCTGCTTCTTCCAATCAAAAG-TCAAATGACAGCCTGGGAGT
GGAAGGTTGG--

---GAAATAGCTATATCCTATAACATGGGA
GTGGGCAAATTGACC-----TCAGGGAACAGCTGTACCCAGGAGACAAGTACTAAATATCTGTAGCTCCTTCAATGCTCT
GGCAAGCCTGCCGGACATGGTGGA-------TGCAGAGAGCAGGAGCGGAAGCCTCTCTTTATAACTGAGGGGGTGGGGA
GGTCTCTAGGGTGGATTGGAGAGCTGCAGC--------------------------------CACCTATCTGCAGGCGAG
CGGGATGCTATGACCTTTTCTGA-TATGAGCTTTTTGTG----TCCTGTCCTCAG---GAAACACACAGTGTACTATGGC
GGCTTCCACGGCAGTCACAGGGTCATCATCTGGCTTTGGGACATCCTAGCCAAT
>Cya
AGATTGACCTCGAGGACTTGAA---GTGAGTGGGCTGCACCTTTCCCTTACTGGCCAGCAGGCCGGTCCCTCATTTCTGT
AATGCTCTTAGCACAGGAAACTACCATTTTCCAACCCTTCGGCTTCTTCCAATC-AAAG-CCAAACGACAGCCTGGGAGT
GGAAGGTTGG--

---GAATTAGC-TTGTCCTATAACATGGGA
GTGGGCAACTTGACC-----TCAGGGAACAGTTGTACCCGGGAGACGAGGACCAGGTACCTGTAGCTCCTTCAGTGCTCT
GGCAAGCCTGCTGGACATGGTGGATGGTATTTGCAGAGAGCAGGAGCGGAAGCCCC----TATAACTGAGGGGGT-GGGA
GGTCTCTAGGGAGGACTGGAGAGCTGCAGC--------------------------------CACCCATCCGCAAGCCAG
CAGGATGCTGTGACCTTGTCTGA-TAGGAGCTTTTTGTG----TCCTGTCCTCAG---GAAACACACCGTGTACTACGGC
GGCTTCCACGGCAGTCACAGGGTCATCATCTGGCTTTGGGACATCCTAGCCAAT
>Ppa
AGATTGACCTCGAGGACTTGAA---GTGAGTGGGCTGCACCTTTCCCTTACTGGCCAGCAGGCCGGTCCCTCATTTCTGT
AATGCTCTTAGTGCAGGAAACTAACATTTTCCAACCCTTCGGCTTCTTCCAATC-AAAG-CCAAACGGCAGCCTGGGAGT
GGAAGGTTGG--

---GAATTAGC-TTGTCCTATAACATGGGA
GTGGGCAACTTGACC-----TCAGGGAACAGTTGTACCCAGGAGACAACGACCAGGTACCTGTAGCTCCTTCAGTGCTCT
GGCAAGCCTGCTGGACATAGTGGATGGTATCTGCAGAGAGCAGGAGCGGAAGCCCC----TATAACTGAGGGGGT-GGGA
GGTCTCTAGGGAGGATTGGAGAGCTGCAGC--------------------------------CACCCATCCGCAAGCCAG
CAGGATGCTGTGACCTTGTCTGA-TAGGAGCTTTTTGCG----TCCTGTCCTCAG---GAAACACACCGTGTACTACGGC
GGCTTCCACGGCAGTCACAGGGTCATCATCTGGCTTTGGGACATCCTAGCCAAT
>Asi_533308314
AGATTGACCTCGAGGACCTGAA---GTGAGTGGGCTGCACCTTTCCCTTACTGTCCAGCAGGCCGGTCCCTCATTTCTGT
AATGCTCTTAGCACAGGAAACTAACCCTTTCCAACCCTTCGGCTTCTTCCAGTCAAAAG-CCAAATGACAGCCTGGGAGT
GAAAGGTTGG--

---GAAATAGC-ATGTCCTATAACATGGGA
GCGGGCAAATTGACC-----TCAGGGAACAGTTGTACCCAGGAGACAACGACCAGGTCTCTGTAGCTCCTTCAGTGCTCT
GGCAAGCCTGCTGGACATGGTGGATGGTATCTGCAGAGAGCAGGAGTGGAAGCCTCTATTTATAACTGAGGGGGTGGGGA
GGTCTCTAGGGTGGACTGGAGAGCTGCAGC--------------------------------CACCCATCCGCAAGCAAG
CAGGATGCTGTGACCTTGTCTGA-TAGGAGCTTTTTGTG----TCCTGTCCTCAG---GAAACACACCGTGTACTACGGC

```
GGCTTCCACGGCAGTCACAGGGTCATCATCTGGCTTTGGGACATCCTAGCCAAT
>Ami_3783_947350-948900
AGATTGACCTCGAGGACCTGAA---GTGAGTGGGCTGCACCTTTCCCTCACTGTCCAGCAGGCCGGTCCCTCATTTCTGT
AATGCTCTTAGCACAGGAAACTAACCCTTTCCAACCCTTCGGCTTCTTCCAGTCAAAAG-CCAAATGACAGCCTGGGAGT
GGAAGGCTGG------GAAATCTGATGGCCACCTACAAATTTATCAGGGGTGACCACCAGTATCTGGGGGAACGTTTGTT
CACCAGAGCGCCCCAAGGGATGACGAGGTCGAATGGTCACAAACTACTGCAAGACCGTTTCAGGCTGGACATAAGGAAGA
CTTTCTTTACTGTCCGAGCCCCCAAGGTCTGGAACAGCCTGCCACCGGAGGTGGTTCAAGCGCCTNCATTGAACACCTTC
AAGANGAAACTGGATGCTTATCTTGCTGGGATCCTATGACCCCAGCTGACTTCCTGCCCTTTGGGCAGGGGGCTGGGCTC
GATGATCTTCCGAGGTCCCTTCCAGCCCTAATGTCTATGAAATCTAT------GAAATAGC-ATGTCCTTTAACATGGGA
GTGGGCAAATTGACC-----TCAGGGAACAGTTGTACCCAGGAGACAACGACCAGGTCTCTGTAGCTCCTTCAGTGCTCT
GGCAAGCCTGCTGGACATGGTGGATGGTATCTGCAGAGAGCAGGAGTGGAAGCCTCTATTTATAACTGAGGGGGTGGGGA
GGTCTCTAGGGTGGATTGGAGAGCTGCAGC--------------------------------CACCCATCCGCAAGCGAG
CAGGATGCTGTGACATTGTCTGA-TAGGAGCTTTTTGTG----TCCTGTCCTCAG---GAAACACACCGTGTACTACGGC
GGCTTCCACGGCAGTCACAGGGTCATCATCTGGCTTTGGGACATCCTAGCCAAT
>Gallus_GB
AGATTGACCTCGAGGACTTAAA---GTAAGTGACCAACCAAGCCTGCTGGTC----------------------------
-----TTCTCATTCAGCAAAGCAAGCTGTGTTACTGCGTGGTTATTTGCAGGAGGCTGACCCTTTCCCAGCTGTCTGTTC
ATCCTAATGG----------------------------------------------------------------------
--------------------------------------------------------------------------------
--------------------------------------------------------------------------------
--------------------------------------------------------------------------------
-----------------------------------------------------GAATTAGG-----AGCAGATGGAAGCA
AATGGAATGAACAGCAAGTTACTTAAAGCTAATCTAGACACAGTATTAATGAATGGAAAGGGTCCTTTCCTCCACTGGCA
TGCAGTGGTTCCCAAGAGCAGAATTTCTGTCCTTTCCTTTCCTCTGATCAAGCACCCAGTAAGCATTT-GTGGGGCTCTG
TGCTGGCAGCGTTTGCTATAGCAGTGAACCTTGACTCGTGGCAGCAGTATTGATCCTTCCTGCACTCGTGTGCTGGCAGC
TGTGACCCTGCTGCTGCAGTGGGATGCAAACCTGTCACAACCTTTTTCTCCCTAG---AAAACACACAGTGTACTATGGG
GGCTTCCATGGGAGTCATCGGGTCATTATCTGGTTGTGGGACATCCTAGCCAAT
```

AM21

```
>CR1-6F
--------------------------------------------TTCCCCCCAGCAGCACCTGGGGTGACGAGGAACAAT
GGCCAGAAGCTGCTGGAGAATAGGTTCAGGTT---AGAGATTAGGAGGCACTATTTCACTGTCAGGGTGGCTAGGATCTG
GAACCAACTTCCTAGGGAAGTGGTCCTCGCTCCTACCTTGGGTAAATTCAAAAAGAGGTTGGATGAACACCTGTCTGGGG
TCGTGTGATCCCAGCGTGTGCTCCGGCCAGTGGCGGGGGGTTAGACTAGATGATCTATTCAGGTCCCTTCTGACCCCTAA
CTACTATGAAACTATGAAC---TATGAAACTA------------------------------------------------
--------------------------------------------------------------------------------
--------------------------------------------------------------------------------
--------------------------------------------------------------------------------
------------------------
>Exons
--------------------------------------------------------------------------------
--------------------------------------------------------------------------------
--------------------------------------------------------------------------------
--------------------------------------------------------------------------------
--------------------------------------------------------------------------------
--------------------------------------------------------------------------------
--------------------------------------------------------------------------------
-----------------------------------------------------------AGCCAGGCCATGCGGGTGGTG
CGGACGGTGGGCCAGGCTTTCGAG
>Cpo_scaffold00067
CATGTTGCCAGGAGCGTGGTAGGGGTACGGGCCCATGGGCAGGGTGCAGGAGAGGGTCCCCCACCCCGAAGCAGCACAGG
GCAGAGCTAAGGGGAGAAGTCTGCCGAGGACT------------------------------------------------
--------------------------------------------------------------------------------
--------------------------------------------------------------------------------
----------------------CGTCCCGACTGCCCAGAGATGGAGGCATAGTCTAGACCCCATCCCTGCCAGCGCGAGC
AAGCAGCTGGGGAGTCCGGCTGGTGTCCACAGCCCAGCACGCCTCCTCCCAGCCGTCAGCAGGCCGCGGGGCAGCAGCTG
CACGAGGCAATGCGTGTCAGACTGCTGCCTGCCTGGGCG-CCCCAGCATCTCCTCCATGGCAAGGTGGATGGGAGCAGGA
CCCCGTGCCAGCCAGCCCCATCCCACAGACATCTGCCCCCCGGTCTCGTCCCGCAG---AGCCAGGCCATGCGGGTGGTG
CGGACGGTGGGCCAGGCTTTCGAG
>Tsc
CATGTTGCCAGGAGCGTGGTAGGGGTACGGGCCCATGGGCAGGGTGCAGGAGAGGGTCCCCCACCCCGAAGCAGCACGGG
GCAGAGCTAAGGGGAAAAGTCTGCCGAGGACT------------------------------------------------
--------------------------------------------------------------------------------
--------------------------------------------------------------------------------
----------------------CGTCCCGACTGCCCAGAGATGGAGGCATAGTCTAGACCCCGTCCCCGCCAGTGCGAGC
AAGCAGCTGGGGAGTCCGGCTGGTGTCCACAACCCTGCACGCCGCCTCCCAGCCGTCAGCAGGCCGCAGGGCAGCAGCTG
CATGTGGCAATGCATGTCAGACTGCTGCCTGCCTGGGCT-CCCCAGCATCTCCTCCACGGCAAGGTGGATGGGACCAGGA
CCCCATGCCAGCCAGCCCCGTCCCACAGACATCTGCCCCCTGGTCTCGCCCCGCAG---AGCCAGTCCATGCGGGTGGTG
CGGACGGTGGGCCAGGCTTTCGAG
>Gga_scaffold41681
CATGTTGCCAGGAGTGTGGTAGGGGTATGGGCCCGTGGGCAGGGTGCAGGAGAGGGTCCCCCACCCCGAAGCAGCACGGG
GCAGAGCTAAGGGGAAAAGTCTGCCGAGGACT------------------------------------------------
--------------------------------------------------------------------------------
--------------------------------------------------------------------------------
----------------------CGTCCCGACTGCCCAGAGATGGAGGCATAGTCTAGACCCCATCCCCGCCAGTGCGAGC
AAGCAGCTGGGGAGTCCAGCTGGTGTGCACAACCCTGCATGCCACCTCCCAGCCGTCAGCAGGCCGCAGGGCAGCAGCTG
CACGCGGCAATGCATGTCAGACTGCTGCCTGCCTGGGCT-CCCCAGCATCTCCTCCACGGCAAGGTGGATGGGACCAGGA
CCCCATGCCAGCCAGCCCCGTCCCACAGACATCTGCCCCCTGGTCTCGCCCCGCAG---AGCCAAGCCATGCGGGTGGTG
```

CGGACGGTGGGCCAGGCTTTTGAG
>Cya
---------------------------CGGGCCCGTGGGCAGGGTGCAGGAGAGGGTCCCCTGCCCCGAAGCAGCACGGG
GCAGAGCTAAGGGGAGAAGTCTGTCGAGAATT--

---AGACATCAG
GAGCAGCTGGGGAGTCCGGCTGGTGTCCACAGCCCAGCGCGCCTCCTCCCAGCCATCAGCAGGCCGCGGGGCAGCAGCTG
CACGAGGCAATGCATGTCAGACTGCTGCCTGCCTGGGCTCCCCCAGCATCTCCTCCATGGCAAGATGGATGGGAG-AGGA
CCCCAT----GCCAGCCCTGCCCCACAGACCTCTGCC-------CTCTCCCTGGAG---AACCGGGCCTGGCGGGGGGGG
GGAACGGGGGGCCAGGTTTTCAAG
>Mni
CGTGTTGCCAGGAGCTTGTTAGGGGTACGGGCCCGTGGGCAGGGTGCAGGAGAGGGTCCCCTGCCCCGAAGCAGCACGGG
GCAGAGCTAAGGGGAGAAGTCTGTCGAGAATT--

---AGACATCAG
GAGCAGCTGGGGAGTCCGGCTGGTGTCCACAGCCCAGCGCGCCTCCTCC---------GCAGGCCGCGGGGCAGCAGCTG
CACGAGGCAATGCATGTCAGACTGCTGCCTGCCTGGGCTCCCCCAGCGTCTCCTCCATGGCAAGATGGATGGGAG-AGGA
CCCCAT----GCCAG-CCTGCCCCACAGACCTCTGCC-------CTCGCCCTGCAG---AGCCAGGCCATGCGGGTGGTG
CGGACGGTGGGCCAGGCTTTCGAG
>Ppa
------------------------------------GGGCAGGGTGCAGGAGAGGGTCCCCTGCCCCGAAGCAGCACAGG
GCAGAGCTAAGGGGAGAAGTCTGTCGAGAATT--

---AGACATCAG
GAGCAGCTGGGGAGTCCGGCTGGTGTCCACAGCCCAGCGCGCCTCGTCCCAGCCATCAGCAGGCCGCGGGGCAGCAGCTG
CACGAGGCAATGCATGTCAGACTGCTGCCTGCCTGGGCTCCCCCAGCATCTCCTCCATGGCAAGATGGATGGGAG-AGGA
CCCCAT----GCCAGCCCTGCCCCACAGACATCTGCCCTCTGCCCTCGCCCTGCAG---AGCCAGGCCATGCGGGTGGTG
CGGACGGTGGGCCAGGCTTTTGAG
>Asi_533456113
CGTGTTGCCAGGAGCTTGTTAGGGGTACGGGCCCGTGGGCAGGGTGCAGGAGAGGGTCCCCTGCCCCGAAGCAGCATGGG
GCAGAGCTAAGGGGAGAAGTCTGCCGAGGACT---AGACATCAGGAGGCGCGACTTCACAGTCAGGGCAGCTAGGATCTG
GAACCAACTTCCAAGCGAAGTGGTGCTGGCTCCTACCCTAGGG---GTCTTTAAGAAGTTAGACAAACACCTTGCTGGGG
TCGTTTGACCCCAGTACTTTTTCCTGCCA-TGGCAGGGGGTCGGACTTGATGATCTGCTCAGGTCCCTTCCGACCCG-AC
CAACTATGAAACTATGACT---CGTCCTGACTGCCCAGAGATGGAGGCATAGTCTAGACCCCGTCCCCCCCAGGAAGAGC
AAGCAGCTGGGGAGTCCGGCTGGTGTCCACAGCCCAGCACACCTCCTCCCAGCCATCAGCAGGCCAGGGGGCAGCAGCTG
CACGAGGCAATGCATGTCAGACTGCTGCCTGCCTGGGCTCCCCCAGTGTCTCCTCCATGGCAAGATGGATGGGAG-AGGA
CCCCAT----GCCAGCCCTGCCCCACAGACATCTGCCCTCCGGTCTCGCCCTGCAG---AGCCAGGCCATGCGGGTGGTG
CGGACGGTGGGCCAGGCTTTCGAG
>Ami_5653_399300-400850
NGTGTTGCCAGGAGCTTGTTAGGGGTACGGGCCCGTGGGCAGGGTGCAGGAGAGGGTCCCCTGCCCCAAAGCAGCACGGG
GCAGAGCTAAGGGGAGAAGTCTGCCGAGGACT---AGACATCAGGAGGCGCTACTTCACAGTCAGGGCGGCTAGGATCTG
GAACCAACTTCCAAGCAAAGTGGTGCTGGCTCCTACCATGGG----GTCTTTAAGAGGTTAGACGAACATCTTGCCAGGG
TCATTTGACCCCAGTACTTCTTCCTGCCA-TGGCAGGGGGTCAGACTTGATGATCTGCTCAGGTCCCTTCCGACCCG-AC
CAACTATGAAACTATGACT---CGTCCTGACTGCCCAGAGATGGAGGCATAGTCTAGACCCCGTCCCCCCCAGGGAGAGC
AAGCAGCTGGGGAGTCCGGCTGGTGTCCACAGCCCAGCACGCCTCCTCCCAGCCATCAGCAGGCCGCGGGGCAGCAGCTG
CATGAGGCAATGCACATCAGACTGCTGCCTG-----------CCAGTGTCTCCTCCATGGCAAGATGGATGGGAG-AGGA
CCCCAT----GCCAGCCCTGCCCCACAGACATCTGCCCTCTGGTCTCGCCCTGCAG---AGCCAGGCCATGCGGGTGGTG
CGGACGGTGGGCCAGGCTTTCGAG
>Gallus_GB
--AGGATGCC--------------------
----------GGGGGTGGCTGTGCCGGGACCT--

--GGCATCAC
GA--------------TGGCTTGCATTCTGAGCT------------------------GCAGTC------GCAGTGGCGA
TGCGCGGAAA-----GCCGGGCAGCTGCTCACC---ATGG
GCTGAT----------------------GCCTGTGCTGCTCTCCCTCCCCCTGCAG---AGCCAGGCCATGCGCATCGTG
CGGACGGTGGGACAGGCGTTTGAG

AM22

>CR1-2

------------------------GCAGTGTGGGGATGCAGTCAGGAAGGCC---AA----CCGCACCTTGTCATGCATC
CACAGATGCATCTCGAGCAGGTCCAAGGAGGTGATCCTCCCCCTCTATGCGGCACTGGTCAGGCCGCAGCTGGAGTACTG
CGTCCAGTTCTGGGCGCCGCACTTCAAGAGGGATGTGGACAACATGGAGAGGGTCCAGAGGAGGGCCACTCGCATGATCA
GGGGGCAGCAGGGCAGGCCCTACGAGGAGAGGCTACGGGACCTGAACCTGTTCAGCCTCCACAAGAGAAGGCTGAGGGGG
GATCTAGTGGCCTGTTACAAACTAGTCAGGGGGGACCAGCAGGCACTGGGGGAGTCCCTGTTCCCCCGAGCACTGCCAGG
AGTGACCAGGAAATAACGGTCACAAGCTGGCAGAGGGTAGATTCAGATTAGACATCAGGAGGCGCTACTTCACTGTCAGG
GCGGCTAGGATCTGGAACCAACTTCCAAGCGAAGTGGTGCTG--GCTCCTACCCTGGGGGTCTTTAAGAGGAGGCTTGAC
GAWCACCTTGCTGGGGTCGTTTGACCCCAGTACTCTTTCCTGCCATGGCAGGGGGTCGGACTTGATGATCTGCTCAGGTC
CCTTCCGACCCTACTAACTATGAAACTATGA---AACTATG---------------------------------------
----

```
-------------------
>Exons
--------------------------------------------------------------------------------
--------------------------------------------------------------------------------
--------------------------------------------------------------------------------
--------------------------------------------------------------------------------
--------------------------------------------------------------------------------
--------------------------------------------------------------------------------
--------------------------------------------------------------------------------
--------------------------------------------------------------------------------
--------------------------------------------------------------------------------
--------------------------------------------------------------------------------
--------------------------------------------------------------------------------
--------------------------------------------------------------------------------
--------------------------------------------------------------------------------
------------------------------------------GTGACACACCGCGAGACAGGAGAGGTGATGGTCATGAA
GGAGCTGATCCGCTTTGAT
>Cpo_scaffold05753
CATCTCCTTGCCCAGGAAGTCCACAGCTCTGGGGAAGGATCTTATGCCTCCATGAACCAGCAGACAC-GTTAGTCCTCTT
CCTATCTCTCTCACATGGGGCAGGAACTTCATTCCTCAGAGACACCTCCCCTCCCTGCACCAGCTGACCCCAAGAAGGCA
GGGATGATCCCCCTTAGGCCCCCTCAAAGCCTCTTCTACCCTAGGACTGACCAAGGCCCTGGCTTGTCACTACAGATCCA
-TCTGCC-------CTACAAACTTT-AACATCCCA-----------AGTATAATGGTTGCTTCTGTCTCACTGAGGGTGG
GATCTGCCA-GGTACTGCCCCTG-CCCAGCTTGTGCTACACAGAGCATCAGGTTGGA-----------------------
--------------------------------------------------------------------------------
--------------------------------------------------------------------------------
--------------------------------------------------------------------------------
--------------------------------------------------------------------------------
--------------------------------------------------------------------------------
--------------------------------------------------------------------------------
--------------------------------------------------------------------------------
----------------------------------CTTGCTCTGAAGCTTAGAGGAGCTCAAGGCAGCAAGGAGCCTTTTG
TGTGTGGCTGCCACCCCAACTCCTG-CCCCCTCCCCCAG---GTGACACACCGGGAGACAGGAGAGGTGATGGTCATGAA
GGAGCTGATCCGCTTTGAT
>Tsc
--------------------------------------------------------------------------------
--------------------------------------------------------------------------------
-------------------TCCCTCAAAGCCTCTTCTACCCTAGGACTGACCAAGGCCCTGGCTTCTCACTACAGATCCA
-TCTGCC-------CTGCAAACTTT-AACATCCCA-----------AGTATAATGGTTGCTTCAGTCTCACTGAGGGTGG
GATCTGCCA-GGTACTG-CCCTG-CCCAGCTTGTGCTACACAGAGCATCAGGTCGGA-----------------------
--------------------------------------------------------------------------------
--------------------------------------------------------------------------------
--------------------------------------------------------------------------------
--------------------------------------------------------------------------------
--------------------------------------------------------------------------------
--------------------------------------------------------------------------------
--------------------------------------------------------------------------------
----------------------------------CTTGCTCTGAAGCTTAGAGGAGCTCAAGGCAGCAAGGAGCCTTTTG
TGTGTGGCTGCCACCCCAACTCCTG-CCCCCTCCCCCAG---GTGACACACCGGGAGACAGGAGAGGTGATGGTC-----
-------------------
>Gga_scaffold10445
CATCTCCATGCCCAGGAAGTCCACAGTTCTGGGGAAGGATCTTATGCCTCCATGAGCCAGCAGACAC-GTTAGTCCCCTT
CCTATCTCTCTCACATGGGGCAGGAACTTCACTCCTCAGAGACACCTCCCCTCCCTGCACCAGCTGACCCCAAGAAGGCA
GGGATGATCCCCCTCAGGCCCCCTCAAAGCCTGTTCTACCCTAGGACTGACCAAGGCCCTGGCTTCTCACTACAGATCCA
-TCTGCC-------CTGCAAACTTT-AACATCCCA-----------AGTATAATGGTTGCTTCCATCTCACTGAGGGTGG
GATCTGCCA-GGTACTG-CCCTG-CCCAGCTTGTGCTACACAGAGCATCAGGTCGGA-----------------------
--------------------------------------------------------------------------------
--------------------------------------------------------------------------------
--------------------------------------------------------------------------------
--------------------------------------------------------------------------------
--------------------------------------------------------------------------------
--------------------------------------------------------------------------------
--------------------------------------------------------------------------------
----------------------------------CTTGCTCTGAAGCTTAGAGGAGCTCAAGGCAGCAAGGAGCCTTTTG
TGTGTGGCTGCCACCCCAACTCCTG-CCCCCTCCCCCAG---GTGACACACCGGGAGACAGGAGAGGTGATGGTCATGAA
GGAGCTGATCCGCTTTGAT
>Cya
-------------------------------------------ATGCCTTCATGAGCCAGCATATAC-GTTAGTCCCCTT
CCTATGTCTCTCACATGGGGCAGGAACTTCACTCCTCAGAGACATCTCCCCTCCCTGTACCAGCTGACCCCAAGAAGGCA
GGTATGATCCCCCTCAGGCCCCCTCAAAGCCTCTTCTACCTTAGGGCTGACCAAGGCCCTGGCTTCTCACTACACATCCC
-TCTGTCCTTCATTNTGCAAACTT--AACATCCCC-----------AGTATAATGGTTGCTTCAGTCTCACTGAGGGTGG
GATCTGCCAGGGTGCTGCCCCTTCCCCAGCTTGTGCTCCACAGAGCGTCAGGTTGAA----CCACNCCTTGTCATGCATC
CACAGATGCATCATGAGTAGGTCCAAGGAGGTGATCCTCCCCCTCTATACGGCACTGGTCAGGCCGCAGTTGGAGTCCTG
TGTCCAGTTCTGGGCGCTGCACTTCAGGAGAGATGTGGACAGCATTGAGAGGGTTCAGAGGAGGGCCACCTGCATGATC-
GGGGGCAGCAGGGCAGGCCCTACAAGGAGAGGCTGAGAGACCTGAACCTGTTCAGCCTACACAAGAGAAGGCTGAGAGGG
GATCTAGTGGCCTGTTGCAAACTAGTCAGAGGGGACCAGCAGGCATTGGGGGAGTCCCTATTCCCCTGAGCACTACCAGG
AGTGACTAGA----AATAGTCACAAGCTGGGAGAGGGTAGATTCAGACTAGACATCAGGAGGCACTACTTCACTGTCAGG
GCAGCTAGGATCTGGAGCCAACTTCCAAGAGAAGTGGTGCTG--GCTCCTATCCTGGGGGTCTGTAAGAGGAGGTTAGAC
AAATACCTCGCTGGGGTCATTTGACCTCAGTACTTTTTCCTGACATGGCAGGGGGTCAGACTTGATGATCTGCTCAGGTC
```

```
CCTTCTGACCCTATCAACTATGAAACTATGA---TTTGCTCTGAGGCTTAGAGGAGCTCATGGCAGCAAGGAGCCTTTTG
TGTGTGGCTGCCACCCCAACTCCTGCCCCCCTCCTCCAG---GTGACACACCGCGAGACAGG------------------
-------------------
>Mni
----------------------------------------------------TGAGCCAGCATATACGGTTAGTCCCCTT
CCTACGTCTCTCACATGGGGCAGGAACTTCACTCCTCAGAGACATCTCCCCTCCCTGTACCAGCTGACCCCAAGAAGGCA
GGTATGATCCCCCTCAGGCCCCCTCAAAGCCTCCTCTACCTTAGGACTGACCAAGNCCCTGGTTTCTCACTACACATCCC
-TCTGTCTTTCATTCTGCAAACTTTAACATCCCCC-----------AGTATAATGGTTGCTTCAGTCTCACTGAGGGTGG
GATCTGCCA-GGTGCTGCCCCTT-CCCAGCTTGTGCTCCACAGAGCGTCAGGTTGAA----CCACACCTTGTCATGCATC
CACAGATGCATCATGAGTAGGTCCAAGGAGGTGATCCTCCCCCTCTATGCGGCACTGATCAGGCCGCAGTTGGAGTCCTG
TGTCCAGTTCTGGGCACTGCACTTCAGGAGAGATGTGGACAGCATTGAGAGGATTCAGAGGAGGGCCACCTGCATGATC-
GGGGGCAGCAGGGCAGGCCCTACAAGGAGAGGCTGAGAGACCTGAACCTGTTCAGCCTACACAAGAGAAGGCTGAGAGGG
GATCTAGTGGCCTGTTGCAAACTAGTCCGAGGGGACCAGCAGGCATTGGGGGAGTCCCTGTTCCCCCGAGCACTACCAGG
AGTGACTAGA----AATGGTCACAAGCTGGGAGAGGGTAGATTCAGACTAGACATTAGGAGGCACTACTTCACTGTCAGG
GCGGCTATGATCTGGAGCCAACTTCCAAGAGAAGTGGTGCTG--GCTCCTATCCTGGGGGTCTGTAAGAGGAGGTTAGAC
AAACTCCTCGCTGGGGTCATTTGACCCCAGTACTTTTTCCTGACATGGCAGGGGGTCAGACTTGATGATCTGCTCAGGTC
CCTTCCAACCCTATCAACTATGAAACTATGA---TTTGCTCTGAGGCTTAGAGGAGCTCAAGGCAGCAAGGAGCCTTTTG
TGTGTGGCTGCCACCCCAACTCCTG-CCCCCTCCTCCAG---GTGACACACCGCGAGACA--------------------
-------------------
>Ppa
--------------------------TCTTGGGGGAAGATCTTATGCCTCCATGAGCCAGCATATAC-GTTAGTCCCCTT
CCTACCTCTCTCACATGGGGCAGGAACCTCACTCCTCAGAGACATCTCCCCTCCCTGTACCAGCTGACCCCAAAAAGGCA
GGTATGATCCCCCTCAGG-CCCCTCAAAGCCTCTTCTA------------------CCCTGGCTACTCACTACACATCCC
-TCTGCCCACCATTCAGCACACTGT-AACNNNNNNNNNNNNNNNNNNNNNNNNNNNNNNNNNNNNNNNNNNNNNNNNNNN
NNNNNNNNNNNNNNNNNNNNNNNNNNNNNNNNNNNNNNNNNNNNNNNNNNNNNNNNNN---NNNNNNNNNNNNNNNNNNN
NNNNNNNNNNNNNNNNNNNNNNNNNNNNNNNNNNNNNNNNNNNNNNNNNNNNNNNNNNNNNNNNNNNNNNNNNNNNNNNN
NNNNNNNNNNNNNNNNNNNNNNNNNNNNNNNNNNNNNNNNAGCATTGAGACGGTTCAGAGGAGGGCCACCGGCACGATC-
GGAGGCAGCAGGGCAGCCCCTCCAAGGAGACAAGGAGAGACCTGAACCCCTTCACCCTCCCCAAGTCAAGNNTGAGAGGG
GATCTAGTGGCCTGTTGCAAACTAGTCAGAGGGGACCAGCAGGCATTGGGGGAGTCCCTGTTCCCCTGAGCACTACCAGG
AGTGACTAGA----AATGGTCACAAGCTGGGAGAGGGTAGACTCAGACTAGACATCAGGAGGTGCTATTTCACTGTCAGG
GCGGCTAGGATCTGGAGCCAACTTCCAAGAGAAGTGGTGCTG--GCTCCTATCCTGGGGGTCTTTAAGAGGAGGTTAGAC
AAATACCTCGCTGGGGTCATTTGACCCCAGTACTTTTTCCTGACGTGGCAGGGGGTCAGACTTGATGATCTGCTCAGGTC
CCTTCCGACCCTATCAACTATGAAACTATGA---TTTGGTCTGAGGCTTAGAGGAGCTCAAGGCAGCAAGAAGCCTTTTG
TGTGTGGCTGCCACCTCAACTCCTG-CCCCCTCCCCCAG---GTGACACACCGCGAG------------------------
-------------------
>Asi_533241635
TATTGCCATGCCCTGCAAGTCCACGGTTCTGGGGAAAGATCTTATGCCTCCATGAGCCAGCATATAC-GTTAGTCCCCTT
CCTATCTCTCTCACATGGGGCAGGAACTTCACTCCTCAGAGACATCTCCCCTCCCTGTACCAGCTGACCCCAAGAAGGCA
GGTATGATCCCCCTCAGGCCCCCTCAAAGCCTCTTCTACCCTAGGACTGACCAAGGCCCTGGCTTCTCACTACACATCCC
-TCTGTCCTTCATTCTGCAAACTTT-AACATCCCC-----------AGTATAATGGTTGCTTCAGTCTCACTGAGGGTGG
GATCTGCCA-GGTGCTGCCCCTG-CCCAGCTTGTGCTACACAGAGCATCAGGTTGAA----CCACACCTTGTCATGCATC
CACAGATGCATCATGAGCAGGTCTAAGGAGGTGATCCTCCCCCTCTATGCAGCACTGGTCAGGCCGCAGTTGGAGTACTG
CGTCCAGTTCTGGGCACCGCACTTCAGGAG-GATATGGACAGCATTGAGAGGGTCCAGAGGAGGGCCACCCGCATGATCA
GGGGGCAGCAGGGCAGGCCCTACGAGGAGAGGCTGAGAGACCTGAACCTGTTCAGCCTCCACAAGAGAAGGCTGAGGGGG
GATCTAGTGGCCTGTTACAAACTAGTCAGAGGGGACCAGCAGGCATTGGGGGAGTCCCTGTTCCCCCGAGCACTACCAGG
AGTGACTAGA----AATGGTCACAAGCTGGGAGAGGGTAGATTCAGACTAGACATCAGGAGGCAGTACTTCACTGTCAGG
GCGGCTAGGATCTGGAACCAACTTCCAAGTGAAGTGGTGCTGGCGCTCCTATCCTGGGGGTCTTTAAGAGGAGGTTAGAC
AAATACCTCACTGGGGTCGTTTGACCCCAGTACTTTTTCCTGCCATGGCAGGGAGTCGGACTTGAGGATCTGCTCAGGTC
CCTCCCGACCCTATCAACTATGAAACTATGA---TTTGCTCTGAGGCTTAGAGGAGCTCAAGGCAGCAAGGAGCCTTTTG
TGTGTGGCTGCCACCCCAACTCCTG-CCCCCTCCCCCAG---GTGACACACCGTGAGACAGGAGAGGTGATGGTCATGAA
GGAGCTGATCCGCTTTGAT
>Ami_6070_766000-767550
TATTGCCATGCCCTGCAAGTCCACGGTTCTGGGGAAAGATCTTATGCCTCCATGAGCCAGCATATAC-GTTAGTCCCCTT
CCTATCTCTCTCACATGGGGCAGGAACTTCACTCCTCAGAGACATCTCCCCTCCCTGTACCAGCTGACCCCAAGAAGGCA
GGTATGATCCCCCTCAGGCCCCCTCAAAGCCTCTTCTACCCTAGGACTGACCAAGGCCCTGGCTTCTCACTACACATCCC
-TCTGTCCTTCATTCTGCAAACTTT-AACATCCCC-----------AGTATAATGGTTGCTTCAGTCTCACTGAGGGTGG
GATCTGCCA-GGTGCTGCCCCTG-CCCAGCTTGTGCTACACAGAGCATCAGGTTGAA----CCACACCTTGTCATGCATC
CACAGATGCATCATGAGCAGGTCCAAGGAGGTGATCCTCCCCCTCTATGCAGCACTGGTCAGGCCGCAGTTAGAGTACTG
CATCCAGTTCTGGGCGCTGCACTTCAGGAG-GATATGGACAGCATTGAGAGGGTTCAGAGGAGGGCCACCCGCATAATCA
GGGGGCAGCAGGGCAGGCCTTACGAGGAGAGGCTGAGAGACCTGAACCTGTTCAGCCTCCACAAGAGAAGGCTGAGGGGG
GATCTAGTGGCCTGTTACAAACTAGTCAGAGGGGACCAGCAGGCATTGGGGGAGTCCCTGTTCCCCCGAGCACTACCAGG
AGTGACTAGA----AATGGTCACAAGCTGGGAGAGGGTAGATTCAGACTAGACATCAGGAGGTGCTACTTCACTGTCAGG
GCGGCTAGGATCTGGAACCAACTTCCAAGCAAAGTGGTGCTG--GCTCCTATCCTGGGGGTCTTTAAGAGGAGGTTAGAC
AAATACCTTGCTGGGGTCGTTTGACCCCAGTACTTTTTCCTGCCATGGCAGGGAGTCGGACTTGATGATCTGCTCAGGTC
CCTTCCAACCCTATCAACTATAAAACTATGA---TTTGCTCTGAGGCTTAGAGGAGCTCAAGGCAGCAAGGAGCCTTTTG
TGTGTGGCTGCCATCCCAACTCCTG-CCCCCTCCCCCAG---GTGACACACCGCGAGACAGGAGAGGTGATGGTCATGAA
GGAGCTGATCCGCTTTGAT
>Gallus_GB
--------------------------------------------------------------------------------
--------------------------------------------------------------------------------
----------------------------------------------------------------------------TTCG
GCCAGGCCATCAAGGTGCAGTGCCTTGCCATGCCATTTGTGTTATGAGCATGGTGG---TCTTAGCTGTGTGGGGTGGG
GATGTG----AGTGATGCGTCGC---------------------------------------------------------
--------------------------------------------------------------------------------
--------------------------------------------------------------------------------
--------------------------------------------------------------------------------
--------------------------------------------------------------------------------
--------------------------------------------------------------------------------
```


---AGAGGTGCTGAGTGCC---------------
--------------------------TGCCTTCCGCCAG---GTGACGCACAGGGAAACGGGCGAGGTGATGGTTATGAA
GGAGCTGATCCGCTTTGAT

AM24

>CR1-14

------------------------------------GCTGGGGAGTTCCCC---TCTTGAAAGCACAGAGGCGGAAAGGG
ATCTTGGAGTCATTATTGACTCCAAGATGAACATGAGCCGCCAATGCCAGACCGCAGCCAGCAAGGCCAGCCATACCTTG
TCATGCATCCAAAGATGCATCTCAAGCCGGTCCAGAGAGGTGATACTCCCCCTCTATGCGACTTTGGTCAGGCCGCAGTT
GGAGTACTGCGTCCAGTACTGGGCGCCGCACTTCAAAAGGGATGTGGCCAGCCTGGAGAGGGTTCAGAGGAGGGCCACCC
GCTTGGTGAGAGGGCAGC-AGGACAGGCCCTATGAGGAGAGACTGAGGGACCTGAACCTGTTCAGCCTCAGCAAGAGGAG
GCTGAGGGGGGACCTGGTGGCTGCCTACAAACTCATCAGGGGAGATCAACAGCAAATAGG-AGAGCCCTTTTCTCCCCAG
CACCACCTGGGGTGACGAGGAACAATGGTAATAAGCTGATGGAGAATAGGTTTAGGTTAGAGATCAGAAGGCAATATTTT
ACAGTTAGGGTGGCCAAAATCTGGAACCAACTTCCCAGGGAAGTGGTCCTCGCCCCTACCTTGGGC-AAATTCAAGAGGA
GGTTGGATGATCACCTGTCTGGGGTCTTGTGAACCCAGCATTCATTCCTGCCTGTGGCAGGGGGTCAGG--CTAGATGAT
CTGTTCAGGTCCCTCCTGACCCTAGCTACTATGAAACTA---

>Exons_rc

---------GTTCAAACTTCACCC
>Cpo_scaffold00130
T----GAAGAAAGAGGAAAAGGTGAGATTTGCAAATAGG---AATGACAGTTTTTCTGCTGTTTACAGTAAACTACAGCC
CTATTTGGCTTGGAGGCAAATTTTATGCTCTGTGGTTGTGCTGCAAACAA------------------------------
----

---ACTGTTTTATGTATCTACTGCACATT--CAG
TCATCCAAGCCACATAAGCACCAATGAGCTAGGAGAAATGCTAGCTAGG--AAACAGACCCAGGAGGTGGA-GGGGAGAT
ACTCAC---GTTCAAACTTCACCC
>Tsc
T----GAAGAAAGAGGAAAAGGTGAGATTTGCAAATAGG---AATGACAGTTTTTCTGCTGTTTACAGTAAACTACAGCC
CTATTTGGCTTGGAGGCAAATATTATGCTCTGTGGTTGTGCTGCCAACAA------------------------------
----

---ACTGTTTTATGCATCTACTGCACATTCACAG
TCATCCAAGCCACATAAGCACCAATGAGCTAGGAGAAATGCTAACTAGG--AAACAGACCCAGGAGGTGGA-GGGGAGAT
ACTCAC---GTTCAAACTTCACCC
>Gga_scaffold39444
T----GAAGAAAGAGGAAAAGGTGAGATTTGCAAATAGG---AATGACAGTTTTTCTGCTGTTTACAGTAAACTACAGCC
CTATTTGGCTTGGAGGCAAACATTATGCTCTGTGGTTGTGCTGCCAACAA------------------------------
----

---ACTGTTTTATGCATCTACTGCACATT--CAG
TCATCCAAGCCACATAAGCACCAATGAGCTAGGAGAAATGCTAGCTAGG--AAACAGACCCAGGAGGTGGA-GGGGAGAT
ACTCAC---GTTCAAACTTCACCC
>Cya

--TGACACTTATCTTACTATTTACAGCAAACTATAGCC
CTATTTGGGTTGGAGGCAAAAGTTATGCTCTGTGGTTGTGATGCCAACAAA---CTTTGAAAGCACAGAAGCAGAACAGG
ATCTTGGAGTCAGTAATGACTCCAAGATGAACACGAGCCGCCAAAGCCACACCACAGCCAGCAAGGCTAGCCATACCGTG
TCATGCATCCAAAGGTGCATCCCAAGCCAATCCAGAGAGGTGATCC----CCCCTATGTGACTTTGGTCAAGCTGCAGAT
GGAGTACTGCGTCCAGTACTGGGCACCGCACTTCAAAAGGGATGTGGCCTGCCTG--GAGGGTTCAAAGGAGGGCCACCC
GCTTGGTAAGAGAGCAGCAAGGACAGGCCCTATGAGGAGAGACTGAGGGACCTGAACTTGTTCAGCCTCAGCAAGAGGAG
GCTGAGGGGGGACCTGGTGGCTGCCTACAAACTCATCAGGGGAGATCAACAGCAAATAGGTAGAGCCCTTTTCTCCCCAG
CATCACCTGGGGTGACAAGGAACAATGGTCATAAGCTGATGGAGAATAGGTTTAGGTTACAGATCAGAAGGCAATATTTC
ACAGTTAGGGTAACCAAAATCTGGAACCAACTTCCTAGGGAAGTGGTCCTCACCCCTACCTTGGTCAAAATTCAAGGGGA
GGTTGGATGATCACTTGTCTGGGGTCTTGTAAACCCAGCATTCATTCCTGCCTGTGGCAGGGGCTCAGGGCTAGANTGGT
CTGTTCAAGTCCCTCCTGACCCTAGATACTATGAAACTACTATGAA---ACTGTTTTATGTGTCTACTGCACATT--CAG
TCATCCAAGACACATCAGCACCAACAAGCTAGGAGAAGTGCTAGCTAGG--AAACAGACCCAGGAGGTAGAGGGGNANAT
ACTCAC---GTTTCAACTTCACCC
>Ppa
---------------------------------AACAGA---AATGACACTTATCTTACTGTTTACAGCAAACTACAGCC
CTATTTGGCTTGGAGGCAAAAGTTATGCTCTGTGGTTGTGATGCCAACAAA---CTTTGAAAGCACAGAAGCAGAACGGG
ATGTTGGAGTCATTACTGACTCCAAGATGAACATGAGCCGCCAATGCCACACCACAGCCAGCAAGGCTAGCCATACTGTG
TCATGCATCCAAAGGTGCATCCCAAGCCAATCCAGAGAGGTGATCC-CCCCCTCTATGTGACTTTGGTCAGGCAGCAGTT
GGAGTACTGCGTCCAGTACTGGGTGCCGCACTTCAAAAGGGATGTGGCCAGCCTGGAGAGGGGTCAGAGGAGGGCCACCC
GCTTGGTGAGAGGGCAGC-AGGACAGGCCCTATGAGGAGAGACTGAGGGACCTGAACCTGTTCAGCCTCAGCAAGAGGAG
GCTGAGGGGGGACCTGGTGGCTGCCTACAAACTCATCAGGGGAGATCAACAGCAAATAGGTAGAG----------CCCAG
CACCACTTGGGGTGACAAGGAACAATGGTCATAAGCTGATGGAGAATAGGTTTAGGTTACAGATCAGAAGGCAATATTTC
AC--

>Asi_533273516
T-GAAGAAGAAAGAAGAAAAGGTGAGATTTGCAAACAGG---AATGACAGTTCTTCTGCTGTTTACAGTAAACTACAGCC
CTATTTGGCTTGGAGGCAAAAGTTATGCTCTGTGGTTGTGGTGCCAACAAA---CTTTGAAAGCACAGAAGCGGAACGGG
ATCTTGGAGTCATTACTGACTCCAAGATGAACATGAGCCGCCAATGCCAGACTGCAGCCAGCAAGGCCAGCCATACCTTG
TCATGCATCCAAAGGTGCATCTCCAGTCGGTCCAGAGAGGTGATACTCCCCCTCTATGTGACTTTGGTCAGGCCGCAGTT
GGATTAATGTGTCCAGTACTGGGCGCCGCACTTCAAAAGGGATGTGGCCAGCCTGGAGTGGGTTCAGAGGAGGGCCACCC
GCTTGCTGAGAGGGCAGC-AGGACAGGCCCTATGAGGAGAGACTGAGGGATCTGAACCTGTTCAGCCTCAGCAAGAGGGG
GG---------ACCTGGTGGCTGCCTACAAACTCATCAGGGGAGATCAACAGCGAATAGGTAGAGCCCTTTTCTCCCCAG
CACCACCTGGGGTGACAAGGAACAATGGTCATAAGCTGATGGAGAATAGGTTTAGGTTAGAGATCAGAAGGCAATATTTC
ACAGTTAGGGTGGCCAAAATCTGGAACCAACTTCCCAGGGAAGTGGTCCTCGCCCCTACCTTGGTC-AAATTCAAGAGGA
GGTTGGACGATCACCTGTCTGGGGTCTTGTGAACCCAGCATTCATTCCTGACTGTGGCAGGGGGTCAGG--CTAGATGAT
CTGTTCAGGTCCCTCCTGACCCTAGCTACTACGAAACT--------------GTTTTATGTATGTACTGCACAGT--CAT
CCAA----GCCGCATCAGCACCAATAAGCTAGGAGAAATGCTAGCTAGG--AAACAGACCCAGGAGGTAGA-GGGGAGAT
ACTCAC---GTTCAAACTTCACCC
>Ami_6279_171750-173300
T-GAAGAAGAAAGAAGAAAAGGTGAGATTTGCAAACAGG---AATGACAGTTCTTCTGCTGTTTACAGTAAACTACAGCC
CTATTTGGCTTGGAGGCAAAAGTTATGCTCTGTGGTTGTGGTGCCAACAAA---CTTTGAAAGCACAGAAGCGGAACGGG
ATCTTGGAGTCATTACTGACTCCAAGATGAACATGAGCCGCCAATGCCAGACCACAGCCAGCAAGGCCAGCCATACCTTG
TCATGCATCCAAAGGTGCATCTCAAGCCAGTCCAGAGAGGCGATCCTCCCCCTCTATGCAACTTTGGTCAGCCCGCAGTC
ATAGTACTGCATCCAGTACTGGGCGCCGCACTTCAANAGGGATGTGGCCAGCCTGGAGAGGGTTCAGAGGAGGGCCACCC
ACTTGGTGAGAGGGCAGC-AGGACAGGCCCTATGAAGAGAGACTGAGGGACCTGAACCTGTTCAGCCTCAGCAAGAGGAG
GCTGAGGGGGGACCTGGTGGCTGCCTACAAACTCATCAGAGGAGATCAACAGCAAATAGGCAGAGCCCTTTTCTCCCCAG
CACCACCTGGGGTGACAAGGAACAATGGTCATAAGCTGATGGAGAACAGGTTTAGGTTACAGATCAGAAGGCAATATTTC
ACAGTTAGGGTGGCCAAAATCTGGAACCAACTTCCCAGGGAAGTGGTCCTTGCCCCTACCTTGGTC-AAATTCAAGAGGA
GGTTGGATGATCACCTGTCTGGGGTCTTGTGAACCCAGCATTCATTCCTGCCTGTGGCAGGGGGTCAGG--CTAGATGAT
CCGTTCAGGTCCCTCCTGACCCTAGCTACTATGAAACTACTATGAA---ATTGTTTTATGTATCTACTGCACATT--CAC
TCATCCAAGCCACATCAGCACCAATAAACTAGGAGAAATGCTAGCTAGG--AAACAGACCCAGGA-------GGGGAGAT
ACTCAC---GTTCAAACTTCACCC
>Gallus_GB
TTGAGGAAAAAAAAGGGAGAAATGAGTTTTGCAAATAAATACATTGACAAATCTTCTA----CCACTACTCAGTACAGAT
CCTCCTGATCTAAG--

---ATGTGTTTTT------------------GAG
TTGCCCCATTCCCTGCCACCTCAGTAGA---GGAAAGGCAGAAATAAGGAACATCAGA--------------CAGAAGGT
ACTTAC---GTTCAAATTTCACCC

AM25

>CR1-2
-----GGCGCCGCACTTCAAGAGGGATGTGGACAACATGGAGAGGGTCCAGAGGAGGGCCACTCGCATGATCAGGGGGCA
GCAGGGCAGGCCCTACGAGGAGAGGCTACGGGACCTGAACC---TGTTC---AGCCTCCACAAGAGAAGGCTGAGGGGGG
ATCTAGTGGCCTGTTACAAACTAGTCAGGGGGGACCAGCAGGCACTGGGGGAGTC--CCTGTTCCCCCGAGCACTGCCAG
GAGTGACCAGGAAATAACGGTCACAAGCTGGCAGAGGGTAGATTCAGATTAGACATCAGGAGGCGCTACTTCACTGTCAG
GGCGGCTAGGATCTGGAACCAACTTCCAAGCGAAGTGGTGCTGGCTCCTACCCTGGGGGTCTTTAAGAGGAGGCTTGACG
AWCACCTTGCTGGGGTCGTTTGACCCCAGTACTCTTTCCTGCCATGGCAGGGGGTCGGACTTGATGATCTGCTCAGGTCC

CTTCCGACCCTACTAACTATGAAACTA---TGAAACTATG--

>Exons_rc

---------------CTCATCTACCCCGTAGAGTAACAGAAATTGCTCCTTGTTATACTTGTCCATACACTGTGCAAAGT
CCAAGAGGTCTGTGTCACTGTATTTCACGC
>Cpo_scaffold02171
CTAGTTGGTGAAAGACAAAGT-GTTTTACTTCATATAATAGAAA-CAAAAAGGAAGTAGAAGCAGTAAAAGTACAAGGCA
TTACAATGTTTGGGATTA-AATTCCATTCTGAGTGGCAATTGTTCTGAC-------------------------------
----

-------------------------------ACAACACATCTTAGCATCAGCCATATGATTTTAGAGTATTGCTAGAAGG
GGAGGCCCTTAC---CTCATCCACCCCATAGAGTAACAGAAATTGCTCCTTGTTATACTTGTCCATACACTGCGCAAAGT
CCAAGAGGTCTGTGTCACTGTATTTCACGC
>Tsc
CTAGTTGGTGAAAGACAAAGT-GTTTTACTTCATATAATAGAAA-CAAAAAGGAAGTAGAAGCAGTAAAGGTACAAGGCA
TTACAACGTTTGGGATTA-AATTCCACTCTGGGTGGCAATTGTTCCGAC-------------------------------
----

-------------------------------ACAACACATCTTAGCATCAGCCATATGCTTTTAGAGTATTGCTAGAAGG
GGAGGCCCTTAC---CTCATCCACCCCGTAGAGTAACAGAAATTGCTCCTTGTTACATTGGTCCATACACTGCGCAAAGT
CCAAGAGGTCTGTGTCACTGTATTTCACGC
>Gga_scaffold7196
CTAGTTGGTGAAAGACAAAGT-GTTTTACTTCATATAATAGAAA-AATAAAGGAAGTAGAAGCAGTAAAGGTACAAGGCA
TTACAACGTTTGGGATTA-AATTCCACTCTGGGTGGCAACTGTTCTGAC-------------------------------
----

-------------------------------ACAACACATCTTAGCATCAGCCATATGCTTTTAGAGTATTGCTAGAAGG
GGAGGCCCTTAC---CTCATCCACCCCGTAGAGTAACAGAAATTGCTCCTTGTTATACTTGTCCATACACTGCGCAAAGT
CCAAGAGGTCTGTGTCACTGTATTTCACGC
>Cya
CTAGTTGGTGAAAGATAAAGTGGTTTAACTTCATATAATACAGA-CAAAAAGAAAGTAGAAGCAGTAAAAGTACAAGACA
TTACAACATTTGGGATTA-AATTCTACTTTGGGTGGCAATTGTCCAGAC---TGGAGCCACAAGAGAAGGCTGAGGGGGG
ATCTAGTGGTCTGCTACAAACTAGTCAGAGGGGACCAGCAGGCACTGGGG--AAGTCCCTGTTCCCCCGAGCACTGCCAG
GAGTGACTAG-AAATAACAGTTACAAGCTAGCAGAAGGTAAATTCAGACTAGACATCAGGAGGCGCTACTTCAGAGTCAG
GGCAGCTAGGATCTGGAACCAAATTTCAAGAGAAGTGGTGCTGGCTCCTATCCTGGGGGTCTTTAAGAGGAGGTTAGACG
AACACCTTGCTGGGGT-GTTTGACCCCAGTACTTTTTCCTGCCATGCCAGGGGGTCGGACTTGATGATCTGCTCAGTTCC
CTTCCGACCCTACCAACTATGAAACTG---TGAAACACATGTTAGCATCAGCCATATGCTTTTAGAGTATTGCTAGAAGG
GGAGGTCCTTAC---CTCATCTACCCCGTAGAGTAACAGAAATTGCTCCTTGTTATACTTGTCCATACACTGTGCAAAGT
CCAAAAGGTCTGTGTCACTGTATTTCACGC
>Mni
CTAGTTGGTGAAAGATAAAGTGGTTTAACTTCATATAATACAGA-CAAAAAGAAAGTAGAAGCAGTAAAAGTACAAGACA
TTACAACATTTGGGATTA-AATTCTACTTTGGGTGGCAATTGTCCAGAC---TGGAGCCACAAGAGAAGGCTGAGGGGGG
ATCTAGTGGCCTGCTACAAACTAGTCAGAGGGGACCAGCAGGCACTGGGG--AAGTCCCTGTTCCCCCGAGCACTGCCAG
GAGTGACTAG-AAATAACGGTTACAAGCTAGCAGAAGGTAAATTCAGACTAGACATCAGGAGGCGCTACTTCAGAGTCAG
GGCAGCTAGGATCTGGAACCAAATTTCAAGAGAAGTGGTGCTGGCTCCCATCCTGGGGGTCTTTAAGAGGAGGTTGGATG
AACACCTTGCTGGGGT-GTTTGACCCCAGTACTTTTTCCTGCCATGCCAGGGGGTCGGACTTGATGATCTGCTCAGTTCC
CTTCCGACCCTACCAACTATGAAACTG---TGAAACACATGTTAGCATCAGCCATATGCTTTTAGAGTATTGCTAGAAGG
GGAGGTCCTTAC---CTCATCTACCCCGTAGAGTAACAGAAATTGCTCCTTGTTATACTTGTCCATACACTGTGCAAAGT
CCAAAAGGTCTGTGTCACTGTATTTCACGC
>Ppa
CTAGTTGGTGAAAGATAAAGTGGTTTAACTTCATATAATACAGA-CAAAAAGAAAGTAGAAGCAGTAAAAGTACAAGACA
TTACAACATTTGGGATTA-AATTCTACTTTGGGTGGCAATTGTCCAGAC---TGGAGCCACAAGAGAAGGCTGAGGGGGG
ATCTAGTGGCCTGCTACAAACTAGTCAGAGGGGACCAGCAGGCACTGGGG--AAGTCCCTGTTCCCCCCAGCACTGCCAG
GAGTGACTAG-AAATAACGGTTACAAGCTAGCAGAGGGTAGATTCAGACTAGACATCAGGAGGCGCTACTTCAGAGTCAG
GGCAGCTAGGATCTGGAACCAAATTTCAAGAGAAGTGGCGCTGGCTCCTACCCTGGGGGTCTTTAAGAGGAGGTTACACG
AACACCTTGCTGGGGT-GTTTGACCCCAGTACTTTTTCCTGCCATGGCAGGGGGTCGGACTTGATGATCTGCTCAGGTCC
CTTCCGACCCTACCAACTA-----------TGAAACACATGTTAGCATCAGCCATATGCTTTTAGAGTATTGCT------
--AGGTCCTTAC---CTCATCTACCCCGTAGAGTAACAGAAATTGCTCCTTGTTATACTTGTCCATACACTGTGCAAAGT
CCAAAAGGTCTGTGTCACTGTATTTCACGC
>Asi_533322796|gb|AVPB01085615.1|_10889-11042
CTAGTTGGTGAAAGACAAAGTAGTTTAACTTCATATAATACAGA-CAAAAAGAAAGTAGAAACAGTAAAAGTACAAGGCA
TTACAACGTTTGGGATTA-AATTCCACTTTGGGTGGCAATTGTCCAGAC---TGGAGCCACAAGAGAAGGCTGAGGGGGG
ATCTAGTGGCCTGCTACAAACTAGTCAGAGGGGACCAGCAGGCACTGGGGGAGAGTACCTGTTCCCCCAAGCACTACCAG
GAGTGACTAG-AAATAACGGTCACAAGCTGGCAGAGGGTAGACTCAGACTAGACATCAGGAGGCGCTACTTCACAGTCAG

GGCAGCTAGGATCTGGAACCAAATTCCAAGAGAAGTGGTGCTGGCTCCTACCCTGGGGGTCTTTAAGAGGAGGTTAGACA
AACACCTTGCTGGGGT-GTTTGACCCCACTACTTTTTCCTGCCATGGCAGGGGGTCGGACTTGATGATCTGCTCGGGTCC
CTTCCGACCCTACCAACTATGAAACTA---TGAAACACATCCTAGCATCAGCCATATGCTTTTAGAGTATTGCTAGAAGG
GGAGGCCCTTAC---CTCATCTACCCCGTAGAGTAACAGAAATTGCTCCTTGTTATACTTGTCCATACACTGTGCAAAGT
CCAAGAGGTCTGTGTCACTGTATTTCACGC
>Ami_7024_331000-332550
CTAGTTGGTGAAAGACAAAGTAGTTTAACTTCATATAATACAGA-CAAAAAGAAAGTAGAAGCAGTAAAAGTACAAGGCA
TTACAACGTTTGGAATTA-AATTCCACTTTGGGTGGCAATTGTCCAGAC---TGGAGCCACAAGAGAAGGCTGAGGGGGG
ATCTAGTGGCCTGCTACAAACTAGTCAGA-GGGACCAGCAGGCACTGGGGGAGAGTACCTGTTCCCTCGAGCACTACCAG
GAGTGACTAG-AAATAACGGTCACAAGCTGGCAGAGGGTAGACTCAAACTAGACATCAGGAGGCGCTACTTCACAGTCAG
GGCAGCTAGGATCTGGAACTAAATTCCAAGAGAAGTGGTGCTGGCTCCTACCCTGGGGGTCTTTAAGAGGAGGTTAGACA
AACACCCTGCTGGGGT-GTTTGACCCCAGTACTTTTTCCTGCCATGGCAGGGGGTCGGACTTGATGATCTGCTCGGGTCC
CTTCCGACCTTACCAACTA-----------TGAAACACATTCTAGCATCAGCCACATGCTTTTACAGTATTGCTAGAAGG
GGAGGTCCTTAC---CTCATCTACCCCGTAGAGTAACAGAAATTGCTCCTTGTTATACTTGTCCATACACTGTGCAAAGT
CCAAGAGGTCTGTGTCACTGTATTTCACGC
>Gallus_GB
CTAGTTTAGGGTGGGAGGAGA-----AAGAGAATGTTAAATAAATAATCATAGATGCACGTTCAAGAAGAGAGAAACCCC
CTGGGACATGCAGGATGGCAGGTGGACCTCGGCCCACAGGGATGTGGTT---AGTGGGCAGTATGGGGGGTAGGTGGACG
GTTGGACTAGATGATCTTAGAGACCTTTTCCAACTTTAATGAGTCTATGATTCTATGATTTTTACTTCAATTAACAAAAG
AAAGGGGGAA-AAAAAAAAG--
---AGAGAGGAGGACAGC
GAAACTTCCTGGCACT-GTATGTCCAGGGTTATTTTTTTCCTCAAGGCTGAAGCA-------------------------
-------------------------------AGAGAATAGCTTTGAATAAATCAT--------------------TAAAA
GGAGCCTCTTAC---CTCATCTACCCCATAGAGAAACACAAACTGTTCTCTCTCCTTCTTGTTTATGCACTGTGCAAGGT
CCAAGAGGTCCGTGTCGCTGAACTTCACAC

CP01

>CR1-8

---------------CTCAGCAGTATTCCTGCCCAGAGCAGGGGGGT---------------------TGGACTCGATGA
TCTTTCGAGGTCCCTTCCAGCCCTTAAT---------TTCTATGATTCTA---TGATT----------------------
----

>Exons_rc
CATCTGGG--

>Cpo_1469_112500-114050
CATCTGGG---CTGCAGCAGGGAAACAAAACCA----AGTCAGTGAGTGATTTCACACC-TTCCT-----CCTGTCATCA
GGATCCAGCATCATTAGATGAATTT-ACTAGGAACAGTTAT-AACTTGGAAACACTTTGAGGTCATCCTGGGGCATGAAG
AATAGAATACCTTGTGGAACTCAGTGG---CCCCT-CTAGTTAA-CCAAAGTCATACTGATAGGCCTCC-AAAAGAAGTC
TCAGATATCCTCTG-TTGGGACAGAGATGGGAGGATACAGCCAAATCCTCAGGAGTCAATAAGGGGAC------------
-AAAAGGGAAGAGGCCAAGAGCTACAGAGGCCTCACAGGAAACACTATTT--------------AAAAGCAATCCTGTT-
TATGCAGCACAGTTCCCTATTTCTAGAGGTGC---CAAGTAGTCACTCTTCTCAGTAACTGGAAATAGGTGCTCATCACC
TCCCAGTCAAGCCTGCTGTATTTCTGTCCTTTGCTTTTCCTCCAAAAAC------------AGGATCTTTGTGGAGCGTG
TGTGCTGGACCTGCCTGTTTCTCTACCAATCATCTGATCA-----GCAAGATCCTACTTAGCTGC---TGGACTCGATGA
TCTCCCGAGGTCCCTTCCAGCCCTAATG---------TCTATGAAATCTA---TGA---CAGAACTGTGTCACCACACTC
TC-TTGCGCAAGAATTTTCACCCCAGGCTACGTGAAGC--GAGACAGAAAGAACAAGGGAGACAATGCTTAAATTAGGAA
GAAAAGTTGCA-------------TTGGATAGACACTG-TTTTCATGCTTCTGCTGTACT-CCCTAAAATCCCTTACAAC
TCTTCAGTAGTTTTTAGACAATGTCATTCACACT-GGGCAACAC-AAATACTTACAAGCTGACTGCAGTATTT-AGCAAG
A---TATTATTATTATTGCTGCTCTACTAAAATAGACCTGAAAAA-CCAGTTATTGCTCTGTCATATTTATGGAAACCCA
AGACAAGATACTCCCATAT-GCTATTAATATCC----T-CTTA
>Cni
CATCTGGG---CTGCAGCAGGGAAACAAAACCA----AGTCAGTGAGTGATTTCACACCTTTCCT-----CCTGTCATCA
GGATCCAGCATCATTAGATGTATTT-ACTAGGAACAGTTAT-AACTTGGAAACACTTTGAGGTCATCCTGGGGCATGAAG
AATAGAATACCTTGTGGAACTCAGTGG---CCCCT-CTAGTTAA-CCAAAGTCATACTGATAGGCCTCC-AAAAGAAGTC
TCAGATATCCTCTG-TTGGGACAGAGATGGGAGGATACAGCCAAATCCTCAGGAGTCAATAAGGGGAC------------
-AAAAGGGAAGAGGCCAAGAGCTACAGAGGCCTCACAGGAAACACTATTT--------------AAAAGCAATCCTGTT-

TATGCAGCACAGTTCCCTATTTCTAGAGGTGC---CAAGTAGTCACTCTTCTCAGTAACTGGAAATAGGTGCTCATCACC
TCCCAGTCAAGCCTGCTGTATTTCTGTCCTTTGCTTTTCCTCCAAAAAC-----------AGGATCTTTGTGGAGCGTG
TGTGCTGGACCTGCCTGTTTCTCTACCAATCATCTGATCA-----GCAAGATCCTACTTAGCTGC---TGGACTCGATGA
TCTCCCGAGGTCCCTTCCAGCCCTAATG---------TCTATGAAATCTA---TGA---CAGAACTGTGTCACCACACTC
TC-TTGCGCACGAATTTTCACCCCAGGCTACGTGAAGC--GAGACAGAAAGAACAAGGGAGACAATGCTTAAATTAGGAA
GAAAAGTTGCA------------TTGGATAGACACTG-TTTTCATGCTTCTGCTGTACT-CCCTAAAATCCCTTACAAC
TCTTCAGTAGTTTTTAGACAATGTCATTCACACT-GGGCAACAC-AAATACTTACAAGCTGACTGCAGTATTT-AGCAAG
A---TATTATTATTATTGCTGCTCTACTAAAATAGACCTGAAAAA-CCAGTTATTGCTCTGTCATATTTATGGAAACCCA
AGACAAGATACTCCCATAT-GCTATTAATATCC----T-CTTA
>Cno
CATCTGGG---CTGCAGCAGGGAAACAAAACCA----AGTCAGTGAGTGATTTCACACC-TTCCT-----CCTGTCATCA
GGATCCAGCATCATTAGATGTATTT-ACTAGGAACAGTTAT-AACTTGGAAACACTTTGAGGTCATCCTGGGGCATGAAG
AATAGAATACCTTGTGGAACTCGGTGG--CCCCCT-CTAGTTAA-CCAAAGTCATACTGATAGGCCTCC-AAAAGAAGTC
TCAGATATCCTCTG-TTGGGACAGAGATGGGAGGATACAGCCAAATCCTCAGGAGTCAATAAGGGGAC-----------
-AAAAGGGAAGAGGCCAAGAGCTACAGAGGCCTCACAGGAAACACTATTT-------------AAAAGCAATCCTGTT-
TATGCAGCACAGTTCCCTATTTCTAGAGGTGC---CAAGTAGTCACTCTTCTCAGTAACTGGAAATAGGTGCTCATCACC
TCCCAGTCAAGCCTGCTGTATTTCTGTCCTTTGCTTTTCCTCCAAAAAC-----------AGGATCTTTGTGGAGCGTG
TGTGCTGGACCTGCCTGTTTCTCTACCAATCATCTGATCA-----GCAAGATCCTACTTAGCTGC---TGGACTC---GA
TCTCCCGAGGTCCCTTCCAGCCCTAATG---------TCTATGAAATCTA---TGA---CAGAACTGTGTCACCACACTC
TC-TTGCGCAAGAATTTTCACCCCAGGCTACGTGAAGC--GAGACAGAAAGAACAAGGGAGACAATGCTTAAATTAGGAA
GAAAAGTTGCA------------TTGGATAGACACTG-TTTTCATGCTTCTGCTGTACT-CCCTAAAATCCCTTACAAC
TCTTCAGTAGTTTTTAGACAATGTCATTCACACT-GGGCAACAC-AAATACTTACAAGCTGACTGCAGTATTT-AGCAAG
A---TATTATTATTATTGCTGCTCTACTAAAATAGACCTGAAAAA-CCAGTTATTGCTCTGTCATATTTATGGAAACCCA
AGACAAGATACTCCCATAT-GCTATTAATATCC----T-----
>Cjo
CATCTGGG---CTGCAGCAGGGAAACAAAACCA----AGTCAGTGAGTGATTTCACACC-TTCCT-----CCTGTCATCA
GGATCCAGCATCATTAGATGTATTT-ACTAGGAACAGTTAT-AACTTGGAAACACTTTGAGGTCATCCTGGGGCATGAAG
AATAGAATACCTTGTGGAACTCAGTGG---CCCCT-CTAGTTAA-CCAAAGTCATACTGATAGGCCTCC-AAAAGAAGTC
TCAGATATCCTCTG-TTGGGCCAGAGATGGGAGGATACAGCCAAATCCTCAGGAGTCAATAAGGGGAC-----------
-AAAAGGGAAGAGGCCAAGAGCTACAGAGGCCTCACAGGAAACACTATTT-------------AAAAGCAATCCTGTT-
TATGCAGCACAGTTCCCTATTTCTAGAGGTGC---CAAGTAGTCACTCTTCTCAGTAACTGGAAATAGGTGCTCATCACC
TCCCAGTCAAGCCTGCTGTATTTCTGTCCTTTGCTTTTCCTCCAAAAAC-----------AGGATCTTTGTGGAGCGTG
TGTGCTGGACCTGCCTGTTTCTCTACCAATCATCTGATCA-----GCAAGATCCTACTTAGCTGC---TGGACTCGATGA
TCTCCCGAGGTCCCTTCCAGCCCTAATGTCTATGAAATCTATGAAATCTA---TGA---CAGAACTGTGTCACCACACTC
TC-TTGCGCAAGAATTTTCACCCCAGGCTACGTGAAGC--GAGACAGAAAGAACAAGGGAGACAATGCTTAAATTAGGAA
GAAAAGTTGCA------------TTGGATAGACACTG-TTTTCATGCTTCTGCTGTACT-CCCTAAAATCCCTTACAAC
TCTTCAGTAGTTTTTAGACAATGTCATTCACACT-GGGCAACAC-AAATACTTACAAGCTGACTGCAGTATTT-AGCAAG
A---TATTATTATTATTGCTGCTCTACTAAAATAGACCTGAAAAA-CCAGTTATTGCTCTGTCATATTTATGGAAACCCA
AGACAAGATACTCCCATAT-GCTATTTAATNATC---CTCTTA
>Ote
CATCTGGG---CTGCAGCAGGGAAACAAAACCA----AGTCAGTGAGTGATTTCACACC-TTCCT-----CCTGTCATCA
GGATCCAGCATCATTAGATGTATTT-ACTAGGAACAGTTAT-AACTTGGAAACACTTTGAGGTCATCCTGGGGCATGAAG
AATAGAATACCTTGTGCAACTCAGTGG---TCCCT-CTAGTCAA-CCAAAGTCATACTGATAGGCCTCC-AAAAGAAGTC
TCAGATATCCTCTG-TTGGGACAGAGATGGGAGGATAAAGCCAAATCCTCAGGAGTCAATAAGGGGAC-----------
-AAAAGGGAAGAGGCCAAGAGCTACAGAGGCCTCACAGGAAACACTATTT-------------AAAACCAATCCTGTT-
TATGTAGCACAGTTCCCTATTTCTAGAGGTGC---CAAGTAGTCACTCTTCTCAGTAACTGGAAATAGGTGCTCATCACC
TCCCAGTCAAGCCTGCTGTATTTCTGTCCTTTGCTTTTCCTCCAAAAAC-----------AGGTTCTTTGTGGAGCGTG
TGTGCTGGACCTGCCTGTTTCTCTACCAATCATCTGATCA-----GCAAGATCCTACTTAGCTGC--------------
---TGCAGACAGAAC--TGTCACCACACTC
TC-TTGCGCAAGAATTTTCACCCCAGGCTACGTGAAGT--GAGACAGAAAGAACAAGGGAGACAATGCTTAAATTAGGAA
GAAAAGTTGCA------------TTGGATAGACACTG-TTTTCATGCTTCTGCTGTACT-CCCTAAAATCCCTTACAAC
TCTTCAGTAGTTTTTAGACAATGTCATTCACACT-GGGCAACAC-AAATACTTACAAGCTGACTGCAGTATTT-AGCAAG
ATATTATTATTATTATTGCTGCTCTACTAAAATAGACCTGAAAAA-CCAGTTATTGCTCTGTCACATTTATAGAAACCCA
AGACAAGATACTCCCATGT-GCTATTAATATCC----T-CTTA
>Tsc
--TTTCACNACCTTTCCTTC-----CTGGTCGTCA
GGATCCAGCATCATTAGATGCATTT-ACTAGGAACAGTTAT-AACTTGGAAACACTTTGAGGTCATCCTGGGATGTGAAG
AATAGAATACCCTGTGCAACTCAGTGG---TCCCT-CTAGTCAA-CCAAAGTCATACTGATAGGCCTCC-AAAAGAAGTC
TCAGACATCCTCTG-TTGGGACAGAGATGGGAGGATACAGCCAAATCCTCAGGAGTCAGTAAGGGGAC-----------
-AAAAGGGAAGAGGCCAAGAGCTACAGAGGCCTCACAGGAAATGCTATTT-------------AAAAGCAATCCTGTT-
TATACAGCACAGTTCCCTATTTCTAGAGGTGC---CAAGTAGTCACTCTTCTCAGTAACTGGAAATAGGTGCTCATCACC
TCCCAGTCAAGCCTGCTGTATTTCTGCCCTTTGCTTTTCCTCCAAAAAC-----------AGGATCTTTGTGGAGCATG
TGTGCTGGACCTGCCTGTTTCTCTACCAATCATCTNATCA-----GCAAGATCCTACTTAGCTGC--------------
---TGCAGACAGAACTGTGTCACCACACTC
TC-TTGCGCAACAATTTTCACCCCAGGCTACGTGAAGCGAGAGACAGAAAGAACAAGGGAGACAATGCTTAAATTAGGAA
GAAAAGTTGCA------------TTGGATAGACACTG-TTTTCATGCTTCTGCTGTACT-TCCTAAAATCCCTTACAAC
TCTTCAGTAGTTTTTAGACAATGTCATTCACACT-GGGTGACAC-AAATACTTACAAGCTGACTGCAGTCATT-AGCAAG
G---TATTATTATTATTACTACACTACTAAAATAGACNTGAAAAA-CCAGTTATTGCTCTGTCACATTTATGGAAACCCA
AGACAAGNCACTC-----------------------------
>Gga_scaffold29835
CATCTGGG---CTGCAGCAGGGAAACAAAACCA----AGTCAGTGAGTGATTTCACACC-TTCCT-----CCTGTCGTCA
GGATCCAGCATCATTAGATGCATTT-ACTAGGAACAGTTAT-AACTTGGAAACACTTTGAGGTCATCCTGGGGTGTGAAG
AATAGAATACCTTGTGCGACTCAGTGGTCCCCCCT-CTAGTCAA-CCAAAGTCATACTGATAGGCCTCC-AAAAGAAGTC
TCAGACATCCTCTG-TTGGGACAGAGATGGGAGGATACAGCCAAATCCTCAGGAGTCAGTAAGGGGAC-----------
-AAAAGGGAAGAGGCCAAGAGCTACAGAGGCCTCACAGGAAATGCTATTT-------------AAAAGCAATCCTGTT-
TATACAGCACAGTTCCCTATTTCTAGAGGTGC---CAAGTAGTCACTCTTCTCAGTAACTGGAAATAGGTGCTCATCACC
TCCCAGTCAAGCCTGCTGTATTTCTGCCCTTTGCTTTTCCTCCAAAAAC-----------AGGATCTTTGTGGAGCATG
TGTGCTGGACCTGCCTGTTTCTCTACCAATCATCTGATCA-----GCAAGATCCTACTTAGCTGC--------------

```
-----------------------------------------------------TGCAGACAGAACTGTGTCACCACACTC
TC-TTGCACAAGAATTTTCACGCCAGGCTACGTGAAGCGAGAGACAGAAAGAACAAGGGAGACAATGCTTAAATTAGGAA
GAAAAGTTGCA-------------TTGGATAGACACTG-TTTTCATGCTTCTGCTGTACT-TCCTAAAATCCCTTACAAC
TCTTCAGTAGTTTTTAGACAATGTCATTCACACT-GGGTGACAC-AAATACTTACAAGCTGACTGCAGTCATT-AGCAAG
G---TATTATTATTATTACTGCACTACTAAAATAGACCTGAAAAA-CCAGTTATTGCTCTGTCACATTTATGGAAACCCA
AGACAAGACACTCCCATGTGCTTATTAATATCT----T-CTTA
>Cya
CATCTGGG---CTGTAGCAGGNAAACAAAACGAAGTCAGTCAGTGAGTGATTCCACACCTT--CT-----TCCTCTGTCA
GAATGCAGCGTCATTAGATGCATTT-ACTAAGAACAGTTATAAACTTGGGAACACTTAGGGGTCATCCTGGGGTGTGAAG
AGTAGAATACCTTTTGCAAGTCAGTGG---TCCCTCCTAGTCAA-CCAAAGTCATACTGATAGGCCTAC-ACAAGGAGTC
TCAGATATCCTGTG-TTGTGGTAGAGATGGGAGGATACAGCCAGATTCTCAGGAGTCAGTAAGGGGAC------------
-AAAAGGGAAGAGGCCAAGAGCTACAGATGCCTTGTAGGAAACACTATGT--------------AAAAACAATCCTGTT-
TATGCAGCACAGTTCCCTATTTCTAGAGGTGC---CAAGCAGTCACTCTTTTCAGTAACTGGAAATAGGTGCTCATCACC
TCCCAGTCAAGCCTGCTGTATTTCTGCCCTTTGCTTTTCCTCCAAAAAC------------AGGATCTTTGTGGAGTGTG
TGTGCTGGTACTGCCTATTTCTCTACCAATCATCTGATCA-----GCATGATCCTACTTAGCTGC---------------
-----------------------------------------------------TGCAGACAGAACTGCGTCACCACACTC
TC-TTGCACAAGAATTTTCACTCCAGGCTACGTGAAGCAAGAGACAGAAAGAACACGGGAGACAATGCCTAAATTA-GAA
GAAAAGTTGCA-------------TTGGATAGATACTG-TTTTCATGCTTCTGCTATACT-TCCTAAAATCCCTTACAAC
TCTCGAGTAGTTTTTAGACAATGTCATTCACAAT-GGGCAACAC-AAATACTTACAAGCTGACTGCAGTCATT-AGCAAG
G---TATTATTAATATTGCTGCATTACTAAAATAGACCTGAAAAA-CCAGTTATTGCTCTTTCACATTTGNGGAAACCCA
AGACAAGACAC--------------------------------
>Mca
CATCTGGG---CTGCAGCAGGGAAACAAAACCA----AGTCAGTGAGTGATTTCACACC-TTCCT-----CCTGTCATCA
GGATCCAGCATCATTAGATGTATTT-ACTAGGAACAGTTAT-AACTTGGAAACACTTTGAGGTCATCCTGGGGCATGAAG
AATAGAATACCTTGTGCAACTCAGTGG---TCTCT-CTAGTCAA-CCAAAGTCATACTGATAGGCCTCC-AAAAGAAGTC
TCAGATATCCTCTG-TTGGGACAGAGATGGGAAGATACAGCCAAATCCTCAGGAGTCAACAAGGGGAC------------
-AAAAGGGAAGAGGCCAAGAGCTACAGAGGCCTCACAGGAAACACTATTT--------------AAAAGCAATCCTGTT-
TATGCAGCACAGTTCCCTATTTCTAGAGGTGC---CAAATAGTCACTCTTCTCAGTAACTGGAAATAGGTGCTCATCACC
TCCCAGTCAAGCCTGCTGTATTTCTGTCCTTTGCTTTTCCTCCAAAAAC------------AGGATCTTTGTGGAACATG
TGTCCTGGACCTGCCTGTTTCTCTACCAATCATCTGATCA-----GCAAGATCCTACTTAGCTGC---------------
-----------------------------------------------------TGCAGACAGAACTGTGTCACCACACTC
TC-TTGCGCAAGAATTTTCACCCCAGGCTACGTGAAGC--GAGACAGAAAGAACAAGGGAGACAATGCTTAAATTAGGAA
GAAAAGTTGCA-------------TTGGATAGACACTG-TTTTCATGCTTCTGCTGTACT-CCCTAAAATCCCTTACAAC
TCTTCAGTAGTTTTTAGACAATGTCATTCACACT-GGGCAACAC-AAATACTTACAAGCTGACTGCAGTATTT-AGCAAG
ATATTATTATTATTATTGCTGCTCTACTAAAATAGACCTGAAAAA-CCAGTTATTGCTCTGTCACATTTATGGAAACCCA
AGACAAGATACTCCCATGT-GCTATTAATATCC----T-CTTA
>Asi_533473771
CATCTGGG---CTGTAGCAGGGAAACAAAACCAAGTCAGTCAATGAGTGATTCCACACCTT-CCT-----CCTGTTGTCA
GAATGCAGCATCATTAGATGCATTT-ACTAGGAACAGTTAT-AACTTGGGAACACTTTGGGGTCATCCTGGGGTGTGAAG
AGTAGAATACCTTGTGCGACTCAGTGG---TCCCT-CTAGTCAA-CCAAAGTCATACTGACAGGCCTCC-AAAAGGAGTC
TCAGATATCCTGTG-TTGCGGTAGAGATGGGAGGATACAGCCAGATCCTCAGGAGTCAGTAAGGGGAC------------
-AAAAGGGAAGAGGCCA--------AGATGCCTCATAGGAAACACTATGT--------------CAAAGCAATCCTGTT-
TATGCAGCATAGTTCTCTATTTCTAGAGGTGC---CAAGTAGTCACTCTTCTCAGTAACTGGAAAT----------CACC
TCCCAGTCAAGCCTGCTGTATTTCTGCCCTTTGCTTTTCCTCCAAAAAC------------AGGATCTTTGTGGAATGTG
TGTGCTGGTCCTGCCTATTTCTCTACCAATCATCTGATCA-----GCAAGATCCTACTTAGCTGC---------------
-----------------------------------------------------TGCAGACAGAACTGTGTCACCACACTC
TC-TTCCGCAAGAATTTTCACCCCAGGCTACGTGAAGCGAGAGACAGAAAGAACAAGGGAGACAATGCTTAAATTA-GAA
GAAAAGTTGCA-------------TTGGATAGACACTG-TTTTCATGCTTCTGCTGTACT-TCCTAAAATCCCTTACAAC
TCTTGAGTAGTTTTTAGACAATGTCATTCACAAT-GGGCGACAC-AAACACTTACAAGCTGACTGCAGTCATT-AGCAAG
G---TATTATTATTATTGCTGCACTACTAAAATAGACCTGAAAAA-CCAGTTATTGCTCTTTCACATTTATGGAAACCCA
AGACAAGACACTCCCATGTGCTTATTAATATCC----T-CTTA
>Ami_scaffold-5556
CATCTGGG---CTGTAGCAGGGAAACAAAACCAAGTCAGTCAGTGAGTGATTCCACACCTT-CCT-----CCTGTTGTCA
GAATGCAGCATCATTAGATGCATTT-ACTAGGAACAGTTAT-AACTTGGGAACACTTTGGGGTCATCCTGGGGTGTGAAG
AGTAGAATACCTTGTGCGACTCAGTGG---TCCCT-CTAGTCAA-CCAAAGTCATACTGATAGGCCTCC-AAAAGGAGTC
TCAGATATCCTGTG-TTGCGGTAGAGATGGGAGGATACAGCCAGATCCTCAGGAGTCAGTAAGGGGAC------------
-AAAAGGGAAGAGGCCA--------AGATGCCTCATAGGAAACACTATGT--------------AAAAGCAATCCTGTT-
TATGCAGCACAGTTCCCTATTTCTAGAGGTGC---CAAGTAGTCACTCTTCTCAGTAACTGGAAATAGGTGCTCATCACC
TCCCAGTCAAGCCTGCTGTATTTCTGCCCTTTGCTTTTCCTCCAAAAAC------------AGGATCTTTGTGGAATGTG
TGTGCTGGTCCTGCCTATTTCTCTACCAATCATCTGATCA-----GCAAGATCCTACTTAGCTGC---------------
-----------------------------------------------------TGCAGACAGAACCGTGTCACCACACTC
TC-TTGCGCAAGAATTTTCACCCCAGGCTATGTGAAGCGAGAGACAGAAAGAACAAGGGAGACAATGCTTAAATTA-GAA
GAAAAGTTGCA-------------TTGGATAGACACTG-TTTTCATGCTTCTGCTGTACT-TCCTAAAATCCCTTACAAG
TCTTGAGTAGTTTTTAGACAATGTCATTCACAAT-GGGCAACAC-AAATACTTACAAGCTGACTGCAGTCATT-AGCAAG
G---TATTATTATTATTGTTGCACTACTAAAATAGACCTGAAAAA-CCAGTTATTGCTCTTTCAAATTTATGGAAACCCA
AGACAAGACACTCCCATGTGCTTATTAATATCC----T-CTTA
>Gallus_GB
CATCTGGG---CTGCATGACAAGAATAAAAAAA-ATAATTTAGCAAAGAATTTCATCTACACTATGCTCCTTCTTAACCA
GGATT-------------TTTATTTCACAGAGAAGAGATCT-CACTTGGAAACACTCCAGTGTCA---------------
-------------GTAGAAAGCAGTAA-CCCCT-----------------------TTCATGGGTTACT-----GTGGTG
TCAG------------------------TGGAAGAGACAAGTAGATCCTCAATTACTGTAAAGCAAATTTTGTCCAACGG
GGAAATAGAGAAGTCCAAATGGCAATGTTACCTCAGGAGAAACCGTTTTTGGTGTGTGATTATCAAGAACACCCCTGTTA
CGTGACACATTGTTCACTGCAGATGAAGCTATAGGTATCTTAACACTTCATAGAAAAAATTAAAACAGG-----------
-----------------------CTGTCTTTTTCTTTTGCCAGGGAAATTTATATTGAACGGAGGTCCTTCCTTAGTCTA
ACTGCTACTTCTGAAAATACCTTCATCCAGCAGCCTTCTCACGTTACAAGGTCTTG------------------------
---------------------------------------------------------GTGAGAACAGCAGCCACATGCTT
TT------------CTCTCACCT-----------------TAGCTAAATAGGATACAGAAACTGATGCTGAATATAACCT
AAAGCATTACACAAACCTGAAGGTCTGAATGGAGCCAAG----------------------AAGAAAAATCCATCACAAC
```

TCATAA--------TAAAGAATGGTAGTTTCAG------------ATTATATCAGAAAATAAGCACTGTGTCAGAAAATA
ACCCGCTAGGTTTTTCAGCTAAGCTACAGAAATAAACATGAAATG-ATGGTCTTTGCTCAC----------AGGAAACTC
AGACAAGATATTCCCCCCACTTCTTGGCAACCAGCATCCTTA
>Anolis_GB
CATCTGGG---CTGCAAAAAGCAAATTGTTCCA-TAATATCACCCAAGAATTATTCCATCCATTAAAGA-TTCTTAACCA
AAGTAGTAAAACGTGAGGTTTCCTATA---------------ATTCTGCAAAGACTC--------------ATTAAAATA
ATTATAACATATTTCAGAAACCAAGCACCTCTTTG-CTTGTGAA-AGAAAGCACATTAGAAAGGTTTGGAGAAATAAATG
TTAACAATTTTGTC------------------CAAAAAAACCCATTTTGCATATATTGAAGAACATAT-------ATGAA
AAAATGTGGGGAAAATGTTGTACTCTGTAAGGG---
-----------------------------------AGATGTGATTTTATCAAGTGAAGCACAAGCCAT------GTGAGT
TGCCATGTGAATCTGTCA-----TCATTCTTTGATGTTTTAATAAAAAC------------ATTCTATTGATGTAAGATG
TAAG---ATGAGAACCGACTTTGCA-----------------
---AGATGCACATAAGGAACACATAC
TTGTCACTAAAAAAATGTCTTCTGGGTAAGAGGAAAGTCTTGGGAAGAAATGCTGGAGAAGAAAAGTACTGGGCTAGCTG
GGGAAGCTACTCAATTGAAA--------CTAGACAGTA-TCAG-------ATGCTCAGAAGAAAGAATTAGTATCCAAAT
ACAGTCCATATTTTTTCATGTTGAAACAGTG--------------GTCTGTTTGAAAAATAAAATCAG------------
---------------AAGACTACATTCTGATATTGACTACAATATTAAAGGAATATGAAGG----------TGGAACACT
TGAAAGAACACAA-----TGCAAGCAAAAA-------T-CTTA

CP06

>CR1-L3A

-----------------TTC---ATAGATT--CATAGATNNTAGGGCCGG-AAGGGACCTCNGNAGATCATCTAG---TC
CGA---CCCCC---

>Exon_rc
ATACAAA---

>Cpo_914_78650-80200
ATACAAA---CTGAAAAGCATCATCAAGCAGAGATATTAAAAGATACAGAATCTTTAAAAA----------GGCTGTCTG
GATTTACAATACATGTACTTTATTTTTTATGGCAGGTGACTAACTTCTCAACCTTAGTGGATCAAATTTACCTTTCCATA
AGTTCAACTAGAATAAACCATTAAACAAAAAATGGTGATAAATTCCTCACTGATTTTTGGTTTAGAAGAGCGTCAAGACT
TAAGATGATTGAATTCAAGGTGACTTAATATTGGCACAGAA---GTTTTCATA---------------------------
------------------------------TATTGTATTTCCCGATTTGCTGATCCCCAGAAAAAAGATAATTCAGAAAT
ATTCTTCATGGCAACACCATGGTTTTGGTCCTC-TCTGGGGCTCAATA----------AGCTAATTTTCACTTTATATTC
TTGGAACAGATATATT-AAGTCAACCATTAAGAGAACTTTAACATTAACATATTAATTTAGGTTA-CAAAGAAATTAATA
TTCCTTAGATAAAACAACTGAGTTTCCCTTTGCCATCACACAGCATTTTAAAGAAAGCTGTTGCCATTATGAAAAATTAA
ATTACTGTTTAACTGAAATTAATTTACAACTGGAACAGAAGTAAT-AAAAAATGCT----ACACACACACTAAATGGCAC
TACTATTCCACTTGGATGATTCAAGTAATGTGACTACTCCCTATATTTTGCTCTTAG-AAATATCAGATTGCATCTATTC
TGTACTTTTTTC---ATGTC---ATAGATTT-CATAGACATTAGGGCTGG-AAGGGACCTCGGGAGATCATCGAG---AT
ATC---CGATATGAAAAAATT-ATTTGAACCTTATCATTGAAAACATAAGAATACAATT-AATGAAGAATGAACTGTA-A
TTTTTA------AAGAAGCAGAAT--TTG-TCATTAGGA--TTATTAGCAAAACATAAGGGTACTGATAAGTGATAATAT
CCTGCTCCTTCCCTCCACCTTTTTCCCATGATTCATCCTAAGACACAGCATC-TTTGAGATGCG-CTGTCTTTGAGGGGA
AAAATAAAAGGTTAG--ATATCTGTTCTTTAATGTAGTTCAAAATAAATGTAGTCCTT--ATCCCTCCAATGCCTTTA
>Cni
ATACAAA---CTGAAAAGCATCATCAAGCAGAGATATTAAAAGATACAGAATCTTTAAAAA----------GGCTGTCTG
GATTTACAATACATGTACTTTATTTTTTATGGCAGGTGACTAACTTCTCAACCTTAGTGGATCAAATTTACCTTTCCATA
AGTTCAACTAGAATAAACCATTAAACAAAAAATGGTGATAAATTCCTCACTGATTTTTGGTTTAGAAGAGTGTCAAGACT
TAAGATGATTGAATTCAAGGTGACTTAATATTGGCACAGAA---TTTTTCATA---------------------------
------------------------------TATTGTATTTCCCGATTTGCTGATCCCCAG-AAAAAGATAATTCAGAAAT
ATTCTTCATGGCAACACCACGGTTTTGGTCCTT-TCTGGGGCTCAATA----------AGCTAATTTTCACTTTATATTC
TTGGAACAGATATATT-AAGTCAACCATTAAGAGAACTTTAACATTAACATATTAATTTAGGTTA-CAAAGAAATTAATA
TTCCTTAGATAAAACAACTGAGTTTCCCTTTGCCATCACACAGCATTTTAAAGAAAGCTGTTGCCATTATGAAAAATTAA
ATTACTGTTTAACTGAAATTAATTTACAACTGGAACAGAAGTAAT-AAAAAATGCT----ACACACACACTAAATGGCAC

```
TACTATTCCACTTGGATGATTCAAGTAATGTGACTACTCCCTACATTTTGCTCTTAG-AAATATCAGATTGCATCTATTC
TGTACTTTTTTC---ATATC---ATAGATTT-CATAGACATTAGGGCTGG-AAGGGACCTCGGGAGATCATCGAG---AT
ATC---CGATATGAAAAAATT-ATTTGAACCTTATCATTGAAAACATAAGAATACAATT-AATGAAGAATGAACTGTA-A
TTTTTA------AAGAAGCAGAAT--TTG-TCATTAGGA--TTATTAGCAAAACATAAGGGTACTGATAAGTGATAATAT
CCTACTCCTTCCCTCCACCTTTTTCCCATGATTCATCCTAAGACACAGCATC-TTTGAGATGCG-CTGTCTTTGAGGGGA
AAAATAAAAGGTTAG--ATATCTGTTCTTTAATGTAGTTCAAAATAAATGTAGTCCTT--ATCCCTCCAATGCCTTTA
>Cno
-------------AAAAGCATCATCAAGCAGAGATATTAAAAGATACAGAATCTTTAAAAA----------GGCTGTCGG
GATTTACAATACATGTACTTTATTTTTTATGGCAGGTGACTAACTTCTCAACCTTAGTGGATCAAATTTACGTTTCCATA
AGTTCAAGTAGAATAAACCATTAAACAAAAAATGGTGATAAATTCCTCACTGATTTTTGGTTTAGAAGAGCGTCAAGACT
TAAGATGATTGAATTCAAGGTGACTTAATATTGGCACAGAA---TTTTTCATA---------------------------
------------------------------TATTGTATTTCCCGATTTGCTGATCCCCAG-AAAAAGATAATTCAGAAAT
ATTCTTCATGGCAACACCATGGTTTTGGTCCTT-TCTGGGGCTCAATA----------AGCTAATTTTCACTTTATATTC
TTGGAACAGATATATT-AAGTCAACCATTAAGAGAACTTTAACATTAACATATTAATTTAGGTTA-CAAAGAAATTAATA
TTCCTTAGATAAAACAACTGAGTTTCCCTTTGCCATCACACAGCATTTTAAAGAAAGCTGTTGCCATTATGAAAAATTAA
ATTACTGTTTAACTGAAATTAATTTACAACTGGAACAGAAGTAAT-AAAAAATGCT----ACACACACACTAAATGGCAC
TACTATTCCACTTGGATGATTCAAGTAATGTGACTACTCCCTATATTTTGCTCTTAG-AAATATCAGATTGCATCTATTC
TGTACTTTTTTC---ATATC---ATAGATTT-CATAGACATTAGGGCTGG-AAGGGACCTCGGGAGATCATCGAG---AT
ATC---CGATATGAAAAAATT-ATTTGAACCTTATCATTGAAAACATAAGAATACAATT-AATGAAGAATGAACTGTA-A
TTTTTA------AAGAAGCAGAAT--TTG-TCATTAGGA--TTATTAGCAAAACATAAGGGTACTGATAAGTAATAATAT
CCTGCTCCTTCCCTCCACCTTTTTCCCATGATTCATCCTAAGACACAGCATC-TTTGAGATGCG-CTGTCTTTGAGGGGA
AAAATAAAAGGTTAG--ATATCTGTTCTTTAATGTAGTTCAAAATAAATGTAGNCCTTTATTCCCTNCGATGCCTTTA
>Cjo
----------------------------------------------CAGAATCTTTAAAAA----------GGCTGTCTG
GATTTACAATACATGTACTTTATTTTTTATGGCAGGTGACTAACTTCTCAACCTTAGTGGATCAAATTTACCTTTCCATA
AGTTCAACTAGAATAAACCATTAAACAAAAAATGGTGATAAATTCCTCACTGATTTTTGGTTTAGAAGAGCGTCAAGACT
TAAGATGATTGAATTCAAGGTGACTTAATATTGGCACAGAA---TTTTTCATA---------------------------
------------------------------TATTGTATTTCCCGATTTGCTGATCCCCAG-AAAAAGATAATTCAGAAAT
ATTCTTCATGGCAACACCATGGTTTTGGTCCTC-TCTGGGGCTCAATA----------AGCTAATTTTCACTTTATATTC
TTGGAACAGATATATT-AAGTCAACCATTAAGAGAACTTTAACATTAACATATTAATTTAGGTTA-CAAAGAAATTAATA
TTCCTTAGATAAAACAACTGAGTCTCCCTTTGCCATCACACAGCATTTTAAAGAAAGCTGTTGCCATTATGAAAAATCAA
ATTACTGTTTAACTGAAATTAATTTACAACTGGAACAGAAGTAAT-AAAAAATGCT----ACACACACACTAAATGGCAC
TACTATTCCACTTGGATGATTCAAGTAATGTGACTACTCCCTATAGTTTGCTCTTAGAAAATATCAGATTGCATCTATTC
TGTACTTTTTTC---ATATC---ATAGATTTCCATAGACATTAGGGCTGGAAAGGGACCTCGGGAGATCATCGAG---AT
ATC---CGATATGAAAAAATT-ATTTGAACCTTATCATTGAAAACATAAGAATACAATT-AATGAAGAATGAACTGTA-A
TTTTTA------AAGAAGCAGAAT--TTG-TCGTTAGGA--TTATTAGCAAAACATAAGGGTACTGATAAGTGATAATAT
CCTGCTCCTTCCCTCCACCGTTTTCCCATGATTCTTCCTAAGACACAGCATC-TTTGAGATGCG-CTATCTTTGAGGGGA
AAAATAAAAGGTTAG--ATATCTGTTCTTTAATGTAGTTCAAAA-----------------------------------
>Ote
ATACAAA---CTGAAAAGCATCATCAAGCAGAGATATTAAAAGATACAGAATCTTTAAAAA----------GGCTGTCTG
GATTTACAATACATGTACTTTATTTTTTATGGCAGGTGACTAACTTCTCAACCTTAGTGGATCAAA-TTGCCTTTCCATA
AGTTCAACTAGAATAAACCATTAAACAAAAAATGGTGATAAATTCCTCACTGA-TTTTGGNTTAGAAGAGTGTCAAGACT
TAAGATGATTGAATTCAAGGTGACTTAATATTGGCACAGAA---TTTTTCATA---------------------------
------------------------------TATTGTATTTCCCGATTTGCTGATCCCCAG----AAGATAATTCAGAAAT
ATTCTTCATGGCAACACCATGGTTTTGGTCCTT-TCTGGGGCTCAATA----------AGCTAATTTTCACTTTATATTC
TTGGAACAGATATATT-AAGTCAACCATTAAGAGAACTTTAACATTAACATATTAATTTAGGTTA-CAAAGAAATTAATA
TTCCTTACATAAAACAACTGAGTTTCCCTTTGCCATCACACAACATTTTAAAGAAAGCTGTTGCCATTATGAAAAATTAA
ATTACTGTTTAACTGAAATTAATTTACAACTGGAACAGAAGTAAT-AAAAAATGCT----ACACACACACTAAATGGCAC
TACTATTCCACTTGGATGATTCAAGTAATGTGACTACTCCCTATATTTTGCTCTTAG-AAATATCAGATTGCATCTATTC
TGTACTTTTTTC------------------------------------------------------------------AT
ATC---CGATATGAAAAAATT-ATTTGAACCTTATCACTGAAAACATAAGAATACAATT-AATGAAGAATGAACTATA-A
TTTTTA------AAGAAGCAGAAT--TTG-TCATTAGGA--TTATTAGCAAAACATAAGGGTACTGATAAGTGATAATAT
CCTGCTCCTTCCCTCCACCTTTTTCCCATGATTTACCCTAAGACACAGCATC-TTTGAGATGCG-CTGTCCTTGAGGGGA
AAAATAAAAGGTTAG--ATATCTGTTCTTTAAAGTAGTTCAAGATAAATGTAGTCCTT--ATCCCTCCAATGCCTTTA
>Mca
ATNCAAA---CTGAAAAGCATCATCAAGCAGANATATTAAAAGATNCAGAATCTTTAAAAA----------GGCTGTCTG
GATTTACAATACATGTACTTTATTTTTTATGGCAGGTGACTAACTTCTCAACCTTAGTGGATCAAATTTGCCTTTCCATA
AGTTCAACTAGAATAAACCATTAAACAAAAAATGGTGATAAATTCCTCACTGATTTTTGGTTTAGAAGAGTGTCAAGACT
TAAGATGATTGAATTCAAGGTGACTTAATATTGGCACAGAA---TTTTTCATA---------------------------
------------------------------TATTGTATTTCCCGATTTGCTGATCCCCAG-AAGAAGATAATTCAGAAAT
ATTCTTCATGGCAACACCATGGTTTTGGTCCTT-TCTGGGGCTCAATA----------AGCTAATTTTCTCTTTATATTC
TTGGAACAGATCTATT-AAGTCAACCATTAAGAGAACTTTAACATTAACATATTAATTTAGGTTA-CAAAGAAATTAATA
TTCCTTAGATAAAACAACTGAGTTTCCCTTTGCCATCACACAGCATTTTAAAGAAAGCTGTTGCCATTATGAAAAATTAA
ATTACTGTTTAACTGAAATTAATTTACAACTGGAACAGAAGTAAT-AAAAAATGCT----ACACACACACTAAATGGCAC
TACTATTCCACTTGGATGATTCAAGTAATGTGACTACTCCCTATATTTTGCTCTTAG-AAATATCAGATTGCATCTATTC
TGTACTTTTTTC------------------------------------------------------------------CT
ATC---CGATATGAAAAAATT-ATTTGAACCTTATCATTGAAAACATAAGAATACAATT-AATGAAGAATGAACTGTA-A
TTTTTA------AAGAAGCAGAAT--TTG-TCATTAGGA--TTATTAGCAAAACATAAGGGTACTGATAAGTGATAATAT
CCTGCTCCTTCCCTCCACCTTTTTCCCATGATTTACCCTAAGACACAGCATC-TTTGAGATGCG-CTGTCTTTGAGGGGA
AAAATAAAAGGTTAGCATATCCTGTTCTTTAATGTAGTTCAAAATAAATGTAGTCCCTTATCCCNTCCAATGCCTTTA
>Tsc
ATACAAA---CTGAAAAGCATCATCAAGCAGAGATATTAAAAGATACAGAATCCTTAAAAA----------GGCTGTCTG
GATTTATAATACATGTACTTTATTTTTTATGGCAGGTGACTAACTTCTCAACCTTGGTGGATCAAATTTGCCTTTCCGTA
AGTTCAACTAGAATAAACCATTAAACAAAAAATGGTGATAAATTCCTCACTGATTTTTGGTTTAGAAGAGTGTCAAGACT
CAAGATGATTAAATTCAAGGTGACTTAATATTGGCACAGGA---TTTTTCATA---------------------------
------------------------------TATTGTATTTCCCGATTTGTTGATCCCCAG-AAAAAGATAATTCAGAAAT
ATTCTTCATGGCAACACTGTGGTTTTGGTCCTT-TCTGGGGCTCGATA----------AGCTAATTTTCACTTTATATTC
TTGGAACAGATATATT-AAGTCAACCATTAAGAGAACTTTAACATTAACATATTAATTTAGGTTA-CAAAGAAATTAATA
```

```
TTCCTTAGATAAAACAACTGAGTTTCCCTTTGCCATCACACAGCATTTTAAAGAAAGCTGTTGCCATTATGAAAAATTAA
ATTACTGTTTAACTGAAATTAATTTACAACAGGAACAGAAGTAATAAAAAAATGCT----ACACACACACTAAATGGCAC
TACTATTCCACTTGGATGATTCAAGTAATGTGACTATTCCCTATATTTTGTTCTCAG-AAATATCAGATTGCATCTATTC
TGTCCTTTTTTC------------------------------------------------------------------AT
ATC---CGATATGAAAAAATT-ATTTGAACCTCATCATTGAAAACATAAGAATACAATT-AATGAAGAATGAACTGTAAA
TTTTTA------AAGAAGTAGAAT--TTG-TCATCAGGA--TTATTAGCAAAACATAAGGGTACTGATAAGTGATAATAT
CCTGCTCCTTCCTTCCACCTTTTTCCCATGATTCATCCTAAGACACAGCATC-TTTGAGATGCA-TTGTCTTTGAGGGGA
AAAATAAAAGGTTAG--ATATCTGTTCTTTAATGTAGTCAAAATAAA--TTAGTCCTT--ATCCTTCCAATGTCTTTA
>Gga_scaffold11806
ATACAAA---CTGAAAAGCATCATCAGGCAGAGATATTAAAAGATACAGAATCCTTAAAAA----------GGCTGTCTG
GATTTACAATACATGTACTTTATTTTTTATGGCAGGTGACTAACTTCTCAACCTTGGTGGATCAAATTTGCCTTTCCGTA
AGTTCAACTAGAATAAACCATTAAACAAAAAATGGTGATAAATTCCTCACTGATTTTTGGTTTAGAAGAGTGTCAAGACT
CAAGATGATTAAATTCAAGGTGACTTAATATTGGCACAGGA---TTTTTCATA---------------------------
------------------------------TATTGTATTTCCCGATTTGTTGATCCCCAG-AAAAAGATAATTCAGAAAT
ATTCTTCATGGCAACACTGTGGTTTTGGTCCTT-TCTGGGGCTCAATA----------AGCTAATTTTCACTTTATATTC
TTGGAACAGATATATT-AAGTCAACCATTAAGAGAACTTTAACATTAACATATTAATTTAGGTTA-CAAAGAAATTAATA
TTCCTTAGATAAAACAACTGAGTTTCCCTTTGCCATCACACAGCATTTTAAAGAAAGATGTTGCCATTATGAATAATTTA
ATTACTGTTTAACTGAAATTAATTTACAACAGGAACAGAAGTAATAAAAAAATGCTACACACACACACACTAAGTGGCAC
TACTATTCCACTTGGATGATTCAAGTAATGTGACTATTCCCTATATTTTGTTCTTAG-AAATATCAGATTGCATCTATTC
TGTACTTTTTTC------------------------------------------------------------------AT
ATC---TGATATGAAAAAATT-ATTTGAACCTCATCACTGAAAACATAAGAAAACAATT-AATGAAGAATGAACTGTACA
TTTTTA------AAGAAGTAGAAT--TTG-TCATCAGGA--TTATTAGCAAAACATAAGGGTACTGATAAGTGATAATAT
CCTGCTCCTTCCCTCCACCTTTTTCCCATGATTCATCCTAAGACACAGCATC-TTTGAGATGCA-TTGTCTTTGAGGGGA
AAAATAAAAGGTTAG--ATCTCTGTTCTTTAATGTAGTTCAAAATAAATTTAGTCCTT--ATCCTTCCAATGTCTTTA
>Cya
---------------------------------------AAAGATACAGAGTCCTTAAAAA----------GGCTGTATG
GATTTACAATACGTGTACTTTAATTTTTATGGCAGGTAACTAGCTCCTCAACCTTGGTAGATCAAATTTGCCTTTCCGTA
AGTTCAAGTAGAATAAAGCATTAAACAAAAAATGGTGATAAAATTCCTCACTATTTTTGGTTTAGCAGTGTTTTAATACT
CCCGATGATTAAATTCAAGGCAACTTAATACTGGCACAGAATTTTTTTTTATT---------------------------
------------------------------TATTGGGGTTCCCGATTTGGTGAACCCCCA-AAAAAGATAATTCAGAAAT
ATTCTTCACAGAAACACCGTGGTTTTGGTCCTT-TCTGGGGCTTGATA----------AGCTAATTTTCACTTTATATTC
TTGGAACAGATATATTAAAGCTAGCCATTAAGAGAACTTTAACATTAACGT----ATTTAGGTTA-CATAGAAATTAATA
TCCCTTAGATAAAAGAAATAAGTTGCCCATTGACATCAGAAAGCATTTTAAAAAAAGCTGTTGCCATTATGAAAAATTAA
ATTACTGTTTAACTTTAATTAA---ACAGCAGGGACAGAAGTAAT--AAAAATGCT----ACACACACACTAAATAGCAC
TACTATTGCACTTGGATGAGTCAAGTAACGTGACTATTCCCAATATTTGGTTCTTAG-AAGTATCAAATTGCATCTAGTC
TGTACTTTTTTC------------------------------------------------------------------AT
ATC---CCATATGAAAAAATT-ATTTGAATCTAATCATTGAAAACACAAGGATACAATT-ACTGAAGAACGAACTGAA-G
TTTTTT------AAAAAGTAGGCT--T---TTATCAGGA--TTATTAGCCAATCATAAGGGTACTG---AGTGATAATAT
CCTGCTCCTTCCCCCTACCTTTTTGCCATGATTCATCCTAAGACACAGCATC------------------TTTGAGGGGA
AAAATGAGAGGCTAG--ATACCTGTTCTTTAGTGTAGT-CAAAATAAA------------------------------
>Asi_533457734|gb|AVPB01021305.1|_36473-36740
ATACAAA---CTGAAAAGCATCATCAAGCAAAGATATTAAAAGATACAGAGTCCTTAAAAA----------GGCTGTATG
GATTTACAATACATGTACTTTATTTTTTATGGCAGGTGACTAACTTCTCAACCTTGGTAGATCAAATTTGCCTTTCCATA
AGTTCAACTAGAATAAACCATTAAACAAAAAATGGTGATAAATTCCTCACTAATTTTTGGTTTAGCAGTGTGTTAATACT
CCGGATGATTAAATTCAAGGTGACTTAATATTGGCACAGAA--TTTTTTTATA---------------------------
------------------------------TATTGTATTTCCCGATTTGTTGATCTCCAG-AAAAAGATAATTCAGAAAT
ATTCTTCACAGAAACACCGTGGTTTTGGTCCTT-TCTGGGGCTCGATA----------AGCTAATTTTCACTTTATATTC
TTGGAACAGATATATT-AAGCTAGCCATTAAGAGAACTTTAACATTAATGTAATAATTTAGGTTA-CATAGAAATTAATA
TCCCTTAGATAAAAGAATTAAATTGCCCTTTGACATCA-ACAGCATTTTAAAGAAAGCTGTTGCCATTATGAAAAATTAA
ATTACTGTTTAACTTTAATTAA---ACAGCAGGGACAGAAGTAAT--AAAAATGCTACACACACAC----TAAATAGCAC
TACTATTCCACTTGGATGATTCAAGTAACGTGACTATTCCCAATATTTTGTTCTCAG-AAGTATCAAATTGCATCTACTC
TGTACTTTTTTC------------------------------------------------------------------AT
ATC---CGATATGAAAAAAATTATTTGAACCTAATCATTGAAAACATAAGGATACAATT-AATGAAGAATGAACTGTA-A
TTTTTT------AAAAAGTACGAT--TTT-TCATCAGGA--TTATTAGCAAAACATAAGGGTACTGATGAGTGATAATAT
CCTGCTCC-TCCCCCTACCTTTTTGCCATGATTCATCCTAAGACATAGCATC------------------TTTGAGGGGA
AAAATAAGAGGCTAG--ATATCTGTTCTTTGGTGTAGTTCAAAATAAATTTAGTCCTT--ATCCTTCCAATGTCTTCA
>Ami_scaffold-2097
ATACAAA---CTGAAAAGCATCATCAAGCAAAGATATTAAAAGATACAGAGTCCTTAAAAA----------GGCTGTATG
GATTTACAATACATGTACTTTATTTTTTATGGCAGGTGACTAACTTCTCAACCTTGGTAGATCAAATTTGCCTTTCTGTA
AGTTCAACTAGAATAAACCATTAAACAAAAAATGGTGATAAATTCCTCACTAATTTTTGGTTTAGCAGTGTGTTAATACT
CCCGATGATTAAATTCAAGGTGACTTAATATTGGCACAGAA---TTTTTTATA---------------------------
------------------------------TATTGTACTTCCCGATTTGTTGATCCCCAG-AAAAAGATAATTCAGAAAT
ATTCTTCACAGAAACACCGTGGTTTTGGTCCTT-TTTGGGGCTCGATA----------AGCTAATTTTCACTTTATATTC
TTGGAACAGATATATT-AAGCTAGCCATTAAGAGAACTTTAACATTAACGTATTAATTTAGGTTA-CATAGAAATTAATA
TCCCTTAGATAAAAGAATTAAGCTGCCCTTTGACATCA-ACAGCATTTTAAAGAAAGCTGTTGCCATTATGAAAAATTAA
ATTACTGTTTAACTTTAATTAA---ACAGCAGGGACACAAGTAAT--AAAAATGCTACACACACACACACTAAATAGCAC
TACTATTCCACTTGGATGATTCAAGTAACGTGACTATTCCCAATATTTTGTTCTTAG-AAGTATCAAATTGCATCTACTC
TGTACTTTTTTC------------------------------------------------------------------AT
ATC---CGATATGAAAAAATT-ATTTGAACCTAATCATTGAAAACATAAGGATACAATT-AATGAAGAATGAACTGTA-A
TTTTTT------AAAAAGTACGAT--TTT-TCATCAGGA--TTATTAGCAAAACATAAGGGTACTGTTGAGTGATAATAT
CCTGCTCC-TCCCCCTACCTTTTTGCCATGATTCATCCTAAAACATAGCA------------------TCTTTGAGGGGA
AAAATAAGAGGCTAG--ATATCTGTTCTTTGGTGTAGTTCAAAATAAATTTAGTCCTT--ATCCTTCCAATGTCTTTA
>Gallus_GB
AAACAAA---CTGGAAAGAATCATCACGCACCAGTATTAAAAGGTAAAGTCTCAAAAAAAAAAAACAAGATAAACATTTA
GATTTACGATGTAAATACCCTATCATCAAAAAAAGTTGTTCA-------AACTGCAGTCAGCCAAATTTTTCTTATCAGC
AGTTCAGCTGTAGTAAGCAGATGAATACATAGTACTTGCAA-------TACGATCTTGCAATTAGAG--------GAGCC
CAAGATTGATCAGAGCAAGATGATTTAGCATTACCAGCCATTTTTGTTCAGCAGCTACAACCGCCCTGCTTCTAGAGATG
AAGTTCAGTTTCCTATAGCTGATATTGTGCTGTTACGTTAGCTTTTTTGTGTGCTATTTA-AAAGGGATAAGAGAGA---
```

```
----TTGAAAGAAAAACTTTGCTTTTGTTTTTT-TTTGTTTGTGGTTATTACTATTTCAAATTATTTTCATT---TATTG
TTTAAA-----------AAGTCAATCATCAAGAAAACTATCATTTACTCCTTTTCATGAGAGCAACCACACAAACGAATC
AACTCTGGATTAAA-----------------------ACACACTGCAGGAAAAAAAGAAAGTTCTACGATCA--------
---TCTGCTTAGCCAAGAGTA----AGCACTAGAACAGATATCTT--AAGAATATTCCA-GCACCTACACTA--------
----------------------TTCAAATGTGACCACTCACTATAACTTGGAAAAAATGTATTTAAGTTTTATAACTTTT
TTTTTTTTTTTGCAA---------------------------------------------------------------AT
ATT---TGGGATTAAACTGTCTTCTTAAA-----------GAAACCTAGGTCATCAACAGAAAGGAAATTACAGTGTT-T
CTCTTATTTGACAAGTTGCAAAATCATTGTGTATGATGGTTTTGGCAAC-------AGGCGAACAGGCATGAGATTCCCA
GACAGAATTTCTCTCTCACTCTTCATAATTAATTTAGGTAA---------CTTTTCTGTTTCCAGCAGTTATTAGGAGCT
GAAA-CAGA--------GCTCCTACAATCTGGATTAGTCGCAGA----------------------------------
```

CP07

```
>CR1-2C
--------------------------------------------------------------------------------
---------------------------------------------------------------GCACCTACATTGAACAC
CTTCAAGAGCAAA-------------T---TG---GATGCTTATCTTGCTGGGATCCTATGACCCCAGCTGACTTCCTGC
CCTTCGGGCGGGGGGCTGGACTCGATGATCTTCCGAGGTCCCTTCCAGCCCTAATGTCTATGAAATCTA---TG------
AAA-----------------------------------------------------------------------------
--------------------------------------------------------------------------------
--------------------------------------------------------------------------------
--------------------------------------------------------------------------------
--------------------------------------------------------------------------------
--------------------------------------------------------------------------------
--------------------------------------------------------------------------------
----------------------
>Exon
TTACAG--------------------------------------------------------------------------
--------------------------------------------------------------------------------
--------------------------------------------------------------------------------
--------------------------------------------------------------------------------
--------------------------------------------------------------------------------
--------------------------------------------------------------------------------
--------------------------------------------------------------------------------
--------------------------------------------------------------------------------
--------------------------------------------------------------------------------
--------------------------------------------------------------------------------
--------------------------------------------------------------------------------
----------------------
>Cpo_933_60850-62400
TTACAG---GTAAGGCACTTACCAGCTCCGGGGAGAGGGGGTGAGTAAAGCTGTCCTC--TAGTGCTGGGGATCCACTTG
CGCTGCATGATGTGCCCAATCGGCAGGAGAAGGGAAGCATCTTGGTGCCTGTGGAGGTCACG-TTTGCTGCGTACAAATA
AGTGAGTTGTGTCAGCCGGACACCTTT---TG---GATGCTTATCTTGCTGGGATCCTATGACCCCAGCTGACTTCCTGC
CCTTTGGGCAGGGGGCTGGACTCAGTGATCTCCCGAGGTCCCTTCCAGCCCTAATGTCTATGAAATCTA---TG---CCT
TGTGGCTTGACCTCCTGTGTGGCCGCGGGAGCAGA------------------------------------------CAC
CACACGCAGTGACAGTGCTCGTTGCCG---------------CCCTCCTGTCCGTAGGTGCGTGAGGA----------GA
GCCCGGCCTTTACGGCCCTCAGGTTCTGCGCTGAGGC-TTGCCCCAAGAGGTTTGATATTTGTGCGGCTGCCATGGTGGC
------------------------------------CATCTTTGCGGGTCAGCTCAAGC----AGGACCTGGCTTGAGTT
TCACTTTTAAGATGCTTGAATAATGTCAAGTCCTTTATAATGAGGCTTTTGAGCGTGCTTTGAACCTGCAGCTC------
------ATGCAGGTAGACTCTGCTTTCCTCCCTTCCTAAGCCAGAGTCAGTTGCTCTGCTCCGTTTGCCAGCTGTT-GAT
TGCTTTACC-------TATAGCCGGAGGATTACACTAAGAATGGAGTGTTTTGGGACAGTAACGCAGACTTACTGAGGCT
TCTCTCTCCTCTCTTGATTCCA
>Cni
TTACAG---GTAAGGCACTTACCAGCTCCGGGGAGAGGGGGTGAGTAAAGCTGTCCTC--TAGTGCTGGGGATCCACTTG
CGCTGCATGATGTGCCCAATCAGCAGGAGAAGGGAAGCATCTTGGTGCCTGTGGAGGTCACG-TTTGCTGCATACAAATA
AGTGAGTTGTGTCAGCCGGACACCTTT---TG---GATGCTTATCTTGCTGGGATCCTATGACCCCAGCTGACTTCCTGC
CCTTTGGGCAGGGGGCTGGACTCAGTGATCTCCGGAGGTCCCTTCCAGCCCTAATGTCTATGAAATCTA---TG---CCT
TGTGGCTTGACCTCCTGTGTGGCCGCGGGAGCAGA------------------------------------------CAC
CACACGCAGTGACAGTGCTCGTTGCCG---------------CCCTCCTGTCCGTAGGTGCGTGAGGA----------GA
GCCCGGCCTTTACGGCCCTCAGGTTCTGTGCTGAGGC-TTGCCCCAAGAGGTTTGATATTTGTGCGACTGCCATGGTGGC
------------------------------------CATCTTTGCGGGTCAGCTCAAGC----AGGACCTGGCTTGAGTT
TCACTTTTAAGATGCTTGAATAATGTCAAGTCCTTTATAATGAGGCTTTTGAGCGTGCTTTGAACCTGCAGCTC------
------ATGCAGGTAGACTCTGCTTTCCTCCCTTCCTAAGCCAGAGTCAGTTGCTCTGCTCCGTTTGCCAGCTGTT-GAT
TGCTTTACC-------TATAGCCGGAGGATTACACTAAGAATGGAGTGTTTTGGGACAGCAGCGCAGACTTACTGAGGCT
TCTCTCTCCTCTCTTGATTCCA
>Cno
TTACAG---GTAAGGCACTTACCAGCTCCGGGGAGAGGGGGTGAGTAAAGCTGTCCTC--TAGTGCTGGGGATCCACTTG
CGCTGCATGATGTGCCCAATCGGCAGGAGAAGGGAAGCATCTTGGTGCCTGTGGAGGTCACG-TTTGCTGCGTACAAATA
AGTGAGTTGTGTCAGCCGGACACCTTT---TG---GATGCTTATCTTGCTGGGATCCTATGACCCCAGCTGACTTCCTGC
CCTTTGGGCAGGGGGCTGGACTCAGTGATCTCCCGAGGTCCCTTCCAGCCCTAATGTCTATGAAATCTA---TG---CCT
TGTGGCTTGACCTCCTGTGTGGCCGCGGGAGCAGA------------------------------------------CAC
CACACGCAGTGACAGTGCGTGTTGCCG---------------CCCTCCTGTCCGTAGGTGCGTGAGGA----------GA
GCCCGGCCTTTACGGCCCTCAGGTTCTGCGCTGAGGC-TTGCCCCAAGAGGTTTGATATTTGTGCGACGGCCATGGTGGC
------------------------------------CATCTTTGCGGGTCAGCTCAAGC----AGGACCTGGCTTGAGTT
TCACTTTTAAGATGCTTGAATAATGTCAAGTCCTTTATAATGAGGCTTTTGAGCGTGCTTTGAACCTGCAGCTC------
------ATGCAGGTAAACTCTGCTTTCCTCCCTTCCTAAGCCAGAGTCAGTTGCTCTGCTCCGTTTGCCAGCTGTT-GAT
TGCTTTACC-------TATAGCCGGAGGATTACACTAAGAATGGAGTGTTTTGGGACAGCAACGCAGACTTACTGAGGCT
TCTCTCTCCTCTCTTGATTCCA
>Cjo
```

```
TTACAG---GTAAGGCACTTACCAGCTCCGGGGAGAGGGGGTGAGTAAAGCTGTCCTC--TAGTGCTGGGGATCCACTTG
CACTGCATGATGTGCCCAATCGGCAGGAGAAGGGAAGCATCTTGGTGCCTGTGGAGGTCACG-TTTGCTGCGTACAAATA
AGTGAGTTGTGTCAGCTGGACACCTTT---TG---GATGCTTATCTTGCTGGGATCCTATGACCCCAGCTGACTTCCTGC
CCTTTGGGCAGGGGGCTGGACTCAGTGATCTCCCGAGGTCCCTTCCAGCCCTAATGTCTATGAAATCTA---TG---CCT
TGTGGCTTGACCTCGTGTGTGGCCGCGGGAGCAGA------------------------------------------CAC
CACACGCAGTGACAGTGCTCGTTGCCG---------------CCCTCCTGTCCGTAGGTGCGTGAGGA----------GA
GCCCGGCCTTTACGGCCCTCAGGTTCTGCGCTGAGGC-TTGCCCCAAGAGGTTTGATATTTGTGCGACTGCCATGGTGGC
------------------------------------CATTTTTGCGGGTCAGCTCAAGC----AGGACCTGGCTTGAGTT
TCACTTTTAAGATGCTTGAATAATGTCAAGTCCTTTATAATGAGGCTTTTGAGCGTGCTTTGAACCTGCGGCTC------
------ATGCAGGTAAACTCTGCTTTCCTCCCTTCCTAAGCCAGAGTCAGTTGCTCTGCTCCGTTTGCCAGCTGTT-GAT
TGCTTTACC-------TATAGCCGGAGGATTACACTAAGAATGGAGTGTTTTGGGACAGCAACGCAGACTTACTGAGGCT
TCTCTCTCCTCTCTTGATTCCA
>Ote
TTACAG---GTAAGGCACTTACCAGCTCCGGGGAGAGGGGGTGAGTAAAGCTGTCCTC--TAGTGCTGGGGATCCACTTG
CGCTGCATGATGTGCCCAATCGACAGGAGAAGGGAAGCATCTTGGTGCCTGTGGAGGTCACG-TTTGCTGCGTACAAATA
AGTGAGTTGTGTCAGCCGGACACCTTT-----------------------------------------------------
-------------------------------------------------------------------------TG---CCT
TGTGGCTTGACCTCCTGTGTGGCCGCGGGAGCAGA------------------------------------------CAC
CACACGCAGTGACAGTGCTCGTTGCCG---------------CCCTCCTGTCCGTAGGTGCGTGAGGA----------GA
GCCCGGCCTTTACGGCCCTCAGGTTCTGCGCTGAGGC-TTGCCCCAAGAGGTTTGATATTTGTGCGACTGCCATGGTGGC
------------------------------------CATCTTTGCAGGTCAGCTCAAGC----AGGACCTGGCTTGAGTT
TCACTTTTAAGATGCTTGAATAATGTCAAGTCCTTTATAATGAGGCTTTTGAGCGTGCTTTGAACCTGCAGCTC------
------ATGCAGGTAGACTCTGCTTTCCTCCCTTCCTAAGCC--AGTCAGTTGCTCTGCTCCGTTTGCCAGCTGTT-GAT
TGCTTTACC-------TATAGCCGGAGGATTACACTAAGAATGGAGTGTTTTGGGAAAGCAACGCAGACTTACTGAGGCT
TCTCTCTCCTCTCTTGATTCCA
>Mca
TTACAG---GTAAGGCACTTACCAGCTCCGGGGAGAGGGGGTGAGTAAAGCTGTCCTC--TAGTGCTGGGGATCCACTTG
CGCTGCATGATGTGCCCGATCGACAGGAGAAGGGAAGCATCTTGGTGCCTGTGGAGGTCACG-TTTGCTGCGTACAAATA
AGTGAGTTGTGTCAGCCGGACACCTTT-----------------------------------------------------
-------------------------------------------------------------------------TG---CCT
TGTGGCTTGACCTCCTGTGTGGCCGCGGGAGCAGA------------------------------------------CAC
CACACGCAGTGACAGTGCTTGTTGCCG---------------CCCTCCTGTCCGTAGGTGCGTGAGGA----------GA
GCCCGGCCTTTACGGCCCTCAGGTTCTGCGCTGAGGC-TTGCCCCAAGAGGTTTGATATTTGTGCGACTGCCATGGTGGC
------------------------------------CATCTTTGCGGGTCAGCTCAAGC----AGGACCTGGCTTGAGTT
TCACTTTTAAGATGCTTGAATAATGTCAAGTCCTTTATAATGAGGCTTTTGAGCGTGCTTTGAACCTGCAGCTC------
------ATGCAGGTAGACTCTGCTTTCCTCCCTTCCTAAGCCAGAGTCAGTTGCTCTGCTCCGTTTGCCAGCTGTT-GAT
TGCTTTACC-------TATAGCCGGAGGATTACACTAAGAATGGAGTGTTTTGGGAAAGCAACGCAGACTTACTGAGGCT
TCTCTCTCCTCTCTTGATTCCA
>Tsc
TTACAG---GTAAGGCACTTACCGGCTCTGGAGAAAGGGGGCGAGTAAAGCTGTCCTC--TAGTGCTGGCGATCCACTTG
TGCTGCATGATATGCCCAGTCGGCAGGAGAAGGGAAGCATCTTGGTGCCTGTGGAGGTCACG-TTCGCTGCGTACAAATA
AGCGAGTCGTGTCAGCCGAACACCTTT-----------------------------------------------------
-------------------------------------------------------------------------TG---CCT
TGTGGCTTGACCTCCTATGTGGCCGCGGGAGCAGA------------------------------------------CGC
TACACGCAGTGACAGTACTCGTTGCCG---------------CCCTCCTGTCCGTAGGTGCGTGAGAA----------GA
GCCCAGCCTTTATGGCCCTCAGGTTGTGTGCTGAGGC-TTGCCCCAAGAGGTTTGATATTCATGCGACTGCCATGGTGGC
------------------------------------CATCTTTGCGGGTCAGCTCAAGC----AGGACCCAGCTTGAGTT
TCAGTTTTAAGATGCTTGAATAATATCAGGTCCTTTACAATGAGGCTTTTGAGCATGCTTTGAACCTGCAGCTC------
------ATGCAGGTAGACTCTGCTTTCCTCCCTTCCTAAGCCAGAGTCAGTTGCTCTGCTCTGTTTGCCAGCTGTT-GAT
TGCTTTACC-------TGTAGCCGGAGGATTACACTAAGAATGGAGTGTTTTGGGAGAGCAACGCAGACTTACTGAGGCT
--TCTCTTCTCTCTTGATTCCA
>Gga_scaffold41614
TTACAG---GTAAGGCACTTACCGGCTCCGGGGAGAGGGGGCGAGTAAAGCTGTCCTC--TAGTGCTGGCGATCCACTTG
TGCTGCATGATATGCCCAGTCGGCAGGAGAAGGGAAGCATCTTGGTGCCTGTGGAGGTCACG-TTCGCTGCGTACAAATA
AGCGAGTCGTGTCAGCCGGACACCTTT-----------------------------------------------------
-------------------------------------------------------------------------TG---CCT
TGTGGCTTGACCTCCTGTGTGGCCGCGGGAGCAGA------------------------------------------CGC
TACACGCAGTGACAGTACTCGTTGCCG---------------CCCTCCTGTCCGTAGGTGCGTGAGAA----------GA
GCCCAGCCTTTATGGCCCTCAGGTTGTGTGCTGAGGCTTTGCCCCAAGAGGTTTGATATTCGTGCGACTGCCATGGTGGC
------------------------------------CATCTTTGTGGGTCAGCTCAAGC----AGGACCCAGCTTGAGTT
TCAGTTTTAAGATGCTTGAATAGTATCATGTCCTTTACAATGAGGCTTTTGAGCATGCTTTGAACCTGCAGCTC------
------ATGCAGGTAGACTCTGCTTTCCTCCCTTCCTAAGCCAGAGTCAGTTGCTCTGCTCTGTTTGCCAGCTGTT-GAT
TGCTTTACC-------TATAGCCGGAGGATTGCACTAAGAATGGAGTGTTTTGGGAGAGCAACGCAGACTTACTGAGGCT
--TCTCTCCTCTCTTGATTCCA
>Cya
TTACAG---GTAAGGCACTTACCGGCTCCGGGGAGAGGGGGCGAGTAAAGCTGTCCTCTGTAGTGCTGGGGATCCGCTTG
CGCTGCGTGATGTGCCCACTCGACAGGAGAAGGGAAGCGTCTTGGTGTCTGTGCAGGTCACG-TCCGCTGCGTACAAATA
AGCGAGTTGTGTTGGCTGGACACCTTT-----------------------------------------------------
-------------------------------------------------------------------------TG---CCT
TGCAGCTTGGCATCCTGTGCGGCCACGGGAGCAGA------------------------------------------CAC
TACACGCAGCAGTGGTGCTCGTTGCCG---------------CGCTCCTGTCCGTAGGTGCATGAGAA----------GA
GCCTGGCCTTTACGGCCCTCAGGCTCTGTGCCGAGAC-TTGCCCCGAGAGGTTTGATATTCGTGCGACTGCTGCGGTGGC
------------------------------------CATCTTTGCGGGTCAGCTCAAGG----AGGACCTGGCTTAGGTT
TTGCTTTTAAGATGCTTGAATAATGTCAGGTCCTTTACAATGAGGCTTTTGGGCGAGCTTTGAACCTGTAGCTC------
------ATGCAGGTAGACTCTGCTGTCCTCCCTTCCTAAGCC--GGTCGGTTGCTCTGCTCTGTTTGCCAGCTGTTCAGT
TGCTTTACC-------TATAGCCGGAGGATTACACTGAGGTTTGAGTGTTCTGGGAAAGCAGCGCAGACTGACTGAGGCT
TCTTTCTCCTCTCTTGCTTCCA
>Asi_533499093
```

TTACAG---GTAAGGCACTTACCGGCTCCGGGGAGAGGGGGCGAGTAAAGCTGTCCTCTGTAGTGCTGGGGATCCGCTTG
CGCTGCATGATGTGCCCAGTCAACAGGAGAAGGGAAGCATCTTGGTGCCTGCGCAGGTCACG-TCCGCTGCGTACAAATA
AGCGAGTCGTGTCAGCCGGACGCCTTG---
--TG---GCT
TG-------ACGTCCTGTGTGGCCGCAGGAGCAGA--TGC
TACACGCAGCAACGGTGCTCGTTGCCG---------------CCCTCCTGTCTGTAGGTGCGTGAGAA----------GA
GCCCGGCCTTTACGGCCCTCGGGCTCTGTGCCGAGAC-TTGCACCGAGAGGTTTGATATTCGTGCGACTGCTGCGGTGGC
------------------------------------CATCTTTGCTGGTCAGCTCAAGG----AGGACCTGGCTTGGGTT
TTGCTTTTAAGATGCTTGAATAATGTCAGGTCCTTTACAATGAGGCTTTTGGGCAAGCTTTGAACCTGCAGCTC------
------ATGCAGGTAGACTCTGCTGTCCTCCCTTCCTAAGCC--AGTCAGTTGCTCTGCTCTGTTTGCCAGCTGTTCGCT
TGCTTTACC-------TAGAGCCGGAGGATTACACTAAGAATTGAGTGTTTTGGGAAAGCAGCGCAGACTGACTGAGGCT
TCTCTCTCCCCTCTTGATTCCA
>Ami_scaffold-7861
TTACAG---GTAAGGCACTTACCGGCTCCGGGGAGAGGGGGCGAGTAAAGCTGTCCTCTGTAGTGCTGGGGATCCGCTTG
NGCTGCGTGATGTGCCCAGTCGACAGGAGAAGGGAAGCATCTTGGTGCCTGCGCAGGTCACG-TCCGCTGCGTACAAATA
AGCGAGTTGTGTCGGCCGGACACCTTT---
--CG---CCT
TGTGGCTTGACGTCCTGTGTGGCCGCAGGNGCAGN--TGC
TACACGCAGCAACGGTGCTCGTTGCCN---------------CCTTCCTGTCTGTAGGTGCGTGAGAA----------GA
GCCCGGCCTTCACGGCCCTCAGGCTCTGTGCCGAGAC-TTGCACCGAGAGGTTTGATATTCGTGCGACTGCTGC------
--TGTCAGCTCCAGG----AGGACCTGGCTTGGGTT
TTGCTTTTAAGATGCTTGAATAATGTCAAGTCCTTTACAGTGAGGCTTTTGGGCAAGCTTTGAACCTGCAGCTC------
------ATGCAGGTAGACTCTGCTGTCCTCCCTTCCTAAGCC--AGTCGGTTGCTCTGCTCTGTTTGCCAGCTGTTCGCT
TGCTTTACC-------TATAGCCGGAGGATTACACTAAGAATTGAGCGTTTTGGGAAAGCAGCGCAGACTGACTGAGGCT
TCTCTCTCCCCTCTTGATTGCA
>Gallus_GB
TGACAG---GTGAGCAAACCCCACGCCTTGCA----------GAGCCCAGCTGCCC----TGGTGCTG------CCTTTG
TGAGGGGAGA-----------GCCGGGGCGAGAGCCGGGCGTCAGTGCCCTTCGGGACTCTGTTTCACCTCCTGTCGGTG
CTGAGTTCCTGGCTGTGGGGCTCCTGCTCCAGCCCTGCCCTCAGCAC---------------------------------
--AG---CGC
TGCGTGCTGCTGACTCACGCATCCACGGGAGCTGAGCTCCTTCCTCTGCCCAGGCCTGCCAGCAAGGATTATTTCAGTGT
TCCATACGGTGGAGAATCTGGTCGCAGTTGGACATTTGGAAACGGTTTTCTGCATAGGTCACTGAAAAAGCTGGAGTTGA
ATCCATCCTTCA----------------GCCCGGGGC-TCATACCAATAAGTGTAATGC-----AAACTGAGGTTATGGC
TCCTAAAGCAAAGGGAGAGCAGAGACCCGAGTGTTGCCCCTTTGTAGCCCCACATACACTCAGATGAGCTGTGCCGTGTT
GTGCCGTGGAGGTGCTCGGACAG--ATCTGTAGTTCATCCGG
CTGGAAATGAGGATGGATTTGTCTCAGCTCCAGCTTTAGTTC--AGTAAACGATTCGGGAATTATAGCAGCCTGGT-TTT
TGCTGTATCCTAATGTTGTGCCTGGAGGAT-----------------ACCGGGGGCCAACACCGCTGCCTTTCTG-----
-------TCCCTCCTCCCCGCA

CP08

>CR1-L3A
---AGNTTACCAAG
GGANGTGGTGGANTCTCCATCNCTGGAGGTCTTTAAANCAAGATTGGATGCTTTTCTAGAAGACANGCTCTGGTTCAAAC
AGGAATTAATTCGGGGAAGTCCTGTGGCCTGCGTTA--
-----GCAGGGGGTCGGACTAGATGATCTNCNGAGGTCCCTTCCGGCCCTANNATCTATGAA-TCTATGAA---------
----

>Exons

---TTGCTGATATTCCTG
GTATAATAAGAGGAGCTCATCAAAACAAAGGCCTAGGGCTGA
>Cpo_981_303300-304850
TATGAGTTACAAAAGGCTGTACAT----------------------TACTGCTTCTTTACCCTTATTCTCTGACTTGGGA
TGT----GCATTATGCATGTCTGGTGAAATG---
----ATGTGCCTTTTCAGGGCTGCAAGAAACAAAGCAGCCAAGTTCATGCAGTCT-------------------GCTCAA
GT---GCAGGGGGCTGGACTCGATGATCTCTCGAGGTCCCTTCCAGCCCTGATGTCTATGAAATCTATAAA------TGG
TAGCTGGACAAGTATCTAGACAGATCAGTCACGTACTGTTCCTTGTTGTGTTA-AACATCTTCACAGTTGCA--------
--TTTCTCTTGTAG---TTGCTGATATTCCTG
GTATAATAAGAGGAGCTCATCAAAACAAAGGCCTAGGGCTGA
>Cno
TATGAGTTACAAAAGGCTGTACAT----------------------TACTGCTTCTTTACCCTTATTCTCTGACTTGGGA
TGT----GCAGTATGCATGTCTGGTGAAATG---
----ATGTGCCTTTTCAGGGCTGCAAGAAACAAAGCAGCCAAGTTCATGCAGTCT-------------------GCTCAA
GT---GCAGGGGGCTGGACTCGATGATCTCTCGAGGTCCCTTCCAGCCCTGATGTCTATGAAATCTATAAA------TGG
TAGCTGGACAAGTATCTAGACAGATCAGTCACGTACTGTTCCTTGTTGTGTTA-AACATCTTCACAGTTGCA--------
--TTTCTCTTGTAG---TTGCTGATATTCCTG
GTATAATAAGAGGAGCTCATCAAAACAAAGGCCTAGGGCTGA
>Ote
TATGAGTTACAAAAGGCTGTACAT----------------------TACTGCTTCTTTACCCTTATTCTCTGACTTGGGA
TGT----GCAGTATGCATGTCTGGTGAAATG---
----ATGTGCCTTTTCAGGGCTGCAAGAAACAAAGCAGCCAAGTTCATGCAGTCT-------------------GCTCAA
GT---GCAGGGGGCTGGACTCGATGATCTCTCGAGGTCCCTTCCAGCCCTGATGTCTATGAAATCTATGAA------TGG
TAGCTGGACAAGTATTTAGACAGATCAGTCACGTACTGTTCCTTGTTGTGTTA-AACATGTTCACAGTTGCA--------

--TTTCTCTTGTAG---TTGCTGATATTCCTG
GTATAATAAGAGGAGCTCATCAAAACAAAGGCCTAGGGCTGA
>Mca
TATGAGTTACAAAAGGCTGTACAT----------------------TACTGCTTCTTTACCCTTATTCTCTGACTTGGGA
TGT----GCAGTATGCATGTCTGGTGAAATG---
----ATGTGCCTTTTCAGGGCTGCAAGAAACAAAGCAGCCAAGTTCATGCAGTCT-------------------GCTCAA
GT---GCAGGGGGCTGGACTCGATGATCTCTCGAGGTCCCTTCCAGCCCTGATGTCTATGAAATCTATGAA------TGG
TAGCTGGACAAGTATTTAGACAGATCAGTCACGTACTGTTCCTTGTTGTGTTA-AACATGTTCACAGTTGCA--------
--TTTCTCTTGTAG---TTGCTGATATTCCTG
GTATAATAAGAGGAGCTCATCAAAACAAAGGCCTAGGGCTGA
>Tsc
TATGAGTTACAAAAGGCTGTACAT----------------------TACTGCTTCTTTACCCTTTTTCTCTGACTTAGGA
TGT----GCAGTATGCATGTCTGGTGAAATG---
----ATGTGCCTTTTCAGGGCTGCAAGAAACAAAGCAGCCAGGTTCATGCAGTCT-----------GCTCAAATGCTGCA
GC--GAATGG
TAGCTGGACAAGTATCTAGACAGATCAGTCACATACTGTTCCTTGTTGTGTTA-AACATGTTCACAGTTGCA--------
--TTTCTCTTGTAG---TTGCTGATATTCCTG
GTATAATAAGAGGAGCTCATCAAAACAAAGGCCTAGGGCTGA
>Gga_scaffold11696
TATGAGTTACAAAAGGCTGTACAT----------------------TACTGCTTCTTTACCCTTATTCTCTGACTTGGAA
TGT----GCAGTATGCATGTCTGGTGAAATG---
----ATGTGCCTTTTCAGGGCTGCAAGAAACAAAGCAGCCAGGTTCATGCAGTCT-----------GCTCAAATGCTGCA
GC--GAATGG
TAGCTGGACAAGTATCTAGACAGATCAGTCACATACTGTTCCTTGTTGTGTTA-AACATGTTCACAGTTGCA--------
--TTTCTCTTGTAG---TTGCTGATATTCCTG
GTATAATAAGAGGAGCTCATCAAAACAAAGGCCTAGGGCTGA
>Cya
TATGAGTTACAAAAAGCTGTACAT----------------------TACTGCTTCTTTACCCTTCTTCTCTGACTTGGGA
TGT----TAAGTATGCGTGTCTGGTGAAATG---
----ATGTGCCTTTTCAGGGCTGCAAGAAACAAAGTAGCCAAGTTAATGCAGTCT-----------GCTCAAATGCTACG
GT--GAATGG
TAGCTGGACAAGTATCTAGACAGATCAGTCACGTACTGTTCGTTGTTGTGTTA-AACATGTTTACAGTTGCA--------
--TTTCTCTTGTAG---TTGCTGATATTCCTG
GTATAATACGAGGAGCTCATCAAAACAAAGGCCTAGGGCTGA
>Asi_533347796
TATGAGTTACAAAAGGCTGTACAT----------------------TACTGCTTCTTTACCCTTATTCTCTGACTTGGGA
TGT----GAAGTATGCATGTCTGGTGAAATG---
----ATGTGCCTTTTCAGGGCTGCAAGAAACAAAGCAGCCAAGTTAATGCAGTCT-----------GCTCAAATGCTACA
GT--GAATGG
TAGCTGGACAAGTAGCTAGACAGATCAGTCACGTACTGTTCTTTGTTGTGTTA-AACATGTTCACAGTTGCA--------
--TTTCTCTTGTAG---TTGCTGATATTCCTG
GTATAATAAGAGGAGCTCATCAGAACAAAGGTCTAGGGCTGA
>Ami_scaffold-10386
TATGAGTTACAAAAGGCTGTACAT----------------------TACTGCTTCTTTACCCTTATTCTCTGACTTGGGA
TGT----GAAGTATGCATGTCTGGTGAAATG---
----ATGTGCCTTTTCAGGGCTGCAAGAAACAAAGCAGCCAAGTTAATGCAGTCT-----------GCTCAAATGCTACA
GT--GAATGG
TAGCTGGACAAGTATCTAGACAGATCAGTCACGTACTGTTC---------------CATGTTCACAGTTGCA--------
--TTTCTCTTGTAG---TTGCTGATATTCCTG
GTATAATAAGAGGAGCTCATCAAAACAAAGGCCTAGGGCTGA
>Gallus_GB
TGAAAATTATAGTACACTGAGTGTACCTTGATAGCAGTTTGTGTCACACAGCTTCATT----------------TTTGAA
TGCTGTAGAAGTTAGCGATTCTGTTGAATTATTTCTATGGAACTTAAAACTTGATGTAACAAAAACACTGAAAGCCCATT
TCACATTCACTTATACATAGCCTTAA-TATCAAACTAGCCATCTGAATTCTGTCTTCATTGGAATAGCTTAAATCTGAGG
GG---TGTAAGA
TAATAGCAGCCAGGTTTAACATGAGTTATCCTTAGCATTTATGTGTTGTGATGTAATTTTGTAACACTTGCAATGGGTTG
GGAAAGACTTTGACCTTGTTGTTAAGTGTGTTGGATTAACTGTGAAATTTTTCTCACTGTAG---TTGCTGACATTCCTG
GCCTAATAAGAGGTGCTCATCAGAACAGAGGCCTCGGGATGG

CP10

>CR1-7B
--------GACATCAGGAGGCGCTATTTCACGGTCAGGGCGGCTAGGGTCTGGAACCAACTTCCAAGGGAGGTGGTGCAG
GCTCCTACCCTGGGGGTTTTTAAGAGGA-----------------------------GGTTAGATGAACACCTTGCTGGG
GTCGTTTGACCCCAGTACTCTTTCCTGCCATGGCAGGGGGTCGGACTAGATGATCTGCTCAGGTCCCTTCCGACCCTAGA
AACTATGAAACTATGA---AAC---TA---

>Exons
ACTAG---

---GGCCCCTCCCAGACG
>Cpo_1835_183700-185250
ACTAG---GTAACAGATGGCTGCCATGGAGTTCCTGCTGGTCGGGCCCAGTGACTGGATTGTATTTAT------------
------------TTTTGTATTACTGAATG-AGTGTTCCCTGGATTGTG---AAC---GGTTAGATGCACATCTTGCTGCG
GTTGTTTGACCCCAGTATTGTTTCCTGCCATGGCGGGGGGTCGGACTAGATGATCTACTCGGGTCCCTTCCGACCCTAGA
AACTATGCAACTATGA---AAC---TGAG-----------GGGGGCATTTCCAAGATCCTTGTATAACTGAGCTTTATCA
GGGC-TAGAGTGTGCAAGGGGCTTCCAGAGAGTTGCAGTGGCTTCTCACCCTAGCAATATATCTCTGCATCTGGTGGATT
CAGTGTGTGGGGATTTTTGTTT---CCCCATGCAGAAAAGTTGGAGATGGTTGTTCAGCAGC-------------CAGAC
ACTGTGCAGAGATAACATGTCAGTTTGCTTCATGGGCAGACGTGTATGGACGTGCCACTTCTGCTCTGCTGAAATTCCCA
GTTAAGCTGGGAGGGTGTAATGAGTGTGTGGCTGTCACA--CCTCCGTTGCTTAAAGGTTGAACTCCTTTGCACTTGTTT
TTAAAGAGGGAGTGACTAGGACTTAT--TGGT
CGGGCCCACAGAATGGGCCT----GCTGTATTCCCACTGTGAGGCTGTACAAA-------------------------CC
CTGCTGTCACCAGGGGCTCCGTGAGATCCCAAAGCACTCGACTGTTTCTATCAGTGTGTCAGGTAAGCACAAGGTCATTA
ATGGCCATGCCCTCC-TTTTCCATTGGTCCTGCTCTAG---GTCCCCTCCCAGATG
>Cno
ACTAG---GTAACAGATGGCTGCCATGGAGTTCCTGCTGGTCGGGCCCAGTGACTGGATTGTATTTAT------------
------------TTTTGTATTACTGAATG-AGTGTTCCCTGGATTGTG---AAC---GGTTAGATGCACATCTTGCTGCG
GTTGTTTGACCCCAGTATTGTTTCCTGCCATGGCGGGGGGTCGGACTAGATGATCTACTCAGGTCCCTTCCGACCCTAGA
--------AACTATGA---AAC---TGAG-----------GGGGGCATTTCCAAGATCCTTGTATAACTGAGTTTTATCA
GGGC-TAGAGTTTGCAAGGGGCTTCCAGAGAGTTGCAGTGGCTTCTCACCCTAGCAATATATCTCTGCATCTGGTGGATT
CAGTGTGTGGGGATTTTTGTTT---CCCCATGCAGGAAAGTTGGAGATGGTTGTTCAGCAGC-------------CAGAC
ACTGTGCAGAGATAACATGTCAGTTTGCTTCATGGGCAGATGTGTATGGACGTGTCACTTCTGCTCTGCTGAAATTCCCA
GTTAAGCTGGGAGGGTGTAATGAGTGTGTGGCTGTCACA--CCTCCATTGCTTAAAGGTTGAACTCCATTGCACTTGTTT
TTAAAGAGGGAGTGACTAGGACTTAT--TGGT
CGGGCCCACAGAATGGGCCT----GCTGTATTCCCACTGTGAGGCTGTACAAA-------------------------CC
CTTCTGTCACCAGGGGCTCCATGAGATCCCAAAGCACTCGACTGTTTCTATCAATGTGTCGGGTAAGCACAAGGTCATTA
ATGGCCATGCCCTCC-TTTTCCATTGGTCCTGCTCTAG---GTCCCCTCCCAGATG
>Ote
ACTAG---GTAACAGATGGCTGCCATGGAGTTCCTGCTGGTCGGGCCCAGTGACTGGATTGTATTTAT------------
------------TTTTGTATTACTGAATG-AGTGTTCCCTGGATTGTG---AAC---AGTTAGATGCACATCTTGCTGCG
GTTGTTTGACCACAGTATTGTTTCCTGCCATGGCAGGGGGTCGGACTAGATGATCTACTCAGGTCCCTTCCGACCCTAGA
--------AACTATGA---AAC---TGAG-----------GGGGGCATTTCCAAGATCCTTGTATAACTGAGCTTTATCA
GGGC-TAGAGTTTGCAAGGGGCTTCCAGAGAGTTGCAGTGGCTTCTCACCCTAGCAATATATCTCTGCATCTGGTGGATT
CAGTGTGTGGGGATTTTTGTTT---CCCCATGCAGGAAAGTTGGAGATGGTTGTTCAGCAGC-------------CAGAC
ACTGTGCAGAGATAACAAGTCAGTTTGCTTCATGGGCAGACGTGTATGGACGTGCCACTTCTGCTCTGCTGAAATTCCCA
GTTAAGCTGGGAGGGTGTAATGAGTGTGTGGCTGTCACA--CCTCCATTGCTTAAAGGTTGAACTCCATTGCACTTGTTT
TTAAAGAGGGAGTGACTAGGACTTAC--TGGT
CGGGCCCACAGAATGGGCCT----GCTGTATTCCCACTGTGAGGCTGTACAAA-------------------------CC
CTGCTGTCACCAGGGGCTCCGTGAGATCCCAAAGCACTCGACTGTTTCTGTCAATGTGTCAGGTAAGCACAAGGTCATTA
ATTGCCATGCCCTCC-TTTTCCATTGGTCCTGCTCTAG---GTCCCCTCCCAGATG
>Mca
--------------------------TGCAGGTCTGCTGGTCGGGCCCAGTGACTGGATTGTATTTAT------------
------------TTTTCTATTACTGAATG-AGTGTTCCCTGGATTATG---AAC---GGTTAGATGCACATCTTGCTGCG
GTTGTTTGACCCCAGTATTGTTTCCTGCCATGGCGGGGG---

---------------------------------GTCATA-------------------------------------GTTT
CTAGAG--

--TCGGAAAGTCGGAAGGNN----

>Tsc
ACTAG---GTAACAGATGGCTGCCATGGAGTTCCTGCTGGTCAGGCCCAGTGGCTGGATTGTATTTAT------------
------------GTTTGTATTGCTGAATG-TGTGCTCCCTGGATTGTG--------------------------------
----
-------------------AAC---TGAG-----------GGGGGCATTTCCAAGATCCTTGTATAACTGAGCTTTATCA
GGGN-CGGCGTTTGCAAGGGGCTTCCCGAGAGTTGCAATGGCTTCTCACCCTAGCAATGTATCTCTGCATCTCGCCGATC
CCCTGTGCGGGGACTTTTGTTT---CCCCATGCAGCAAACTTGGAGGTGGTTGTTCAGCAGC-------------CAGAC
ACTGCCCAGAGATAACATGTCAGTTTGCTTCATGGGCAAACGTGTGTGGACGTGCCCCTTCTGCTCCCCCGAAATTCCCA
GTTAAGATGGGAGGGTGCCATGAGTGTGTGGCTGTCGCA--CCTCCATTCCCTGAAGGTTGAATTCCATTGCACTTGTTT
TTAAAGAGGCAGTGACTAGGACTTAT--GGGT
TGGGCCCATAGAATGGGCCT----GCTCTATTCCCACTGCGAGGCTGTACAAC-------------------------CC
TCGCCGTCACCAGGGCCTCAGTCAGATCCCAAAGCACTTGACTGTTTCTGCCAAGGCGTCAGGTAACCACAAGGTCATTA
ATGGCCATATCCTCC-TTTTCCATTGGTCCTGCTCTAG---GTCCCCTCCCAGATG
>Gga_scaffold9622
ACTAG---GTAACAGATGGCTGCCATGGAGTTCCTGCTGGTCAGGCCCAGTGGCTGGATTGTATTTAT------------
------------GTTTGTATTGCTGAATG-AGTGCTCCCTGGATTGTG--------------------------------
----
-------------------AAC---TGAG-----------GGGGGCATTTCCAAGATCCTTGTATAACTGAGCTTTATCA

```
GGGC-TAGAGTTTGCAAGGGGCTTCCAGAGAGTTGCAATGGCTTCTCACCCTAGGAATGTATCTCTGCATCTGGTGGATT
CAGTGTGTGGGGATTTTTGTTT---CCCCATGCAGGAAAGTTGGAGGTGGTTGTTCAGCAGC-------------CAGAC
ACTGTGCAGAGATAACGTGTCAGTTTGCTTCATGGGCAAACGTGTATGGACGTGCCACTTCTGCTCTGCTGAAATTCCCA
GTTAAGCTGGGAGGGTGTAATGAGTGTGTGGCTGTCGCA--CCTCCATTGCTTGAAGGTTGAATTCCATTGCACTTGTTT
TTAAAGAGGCAGTGACTAGGACTTAT--------------------------------------------------GGGT
TGGGCCCATAGAACGGGCCT----GCTGTATTCCCACTGTGAGGCTGTACAAA-------------------------CC
TTGCTGTCACCAGGGGCTCAGTGAGATCCCAAAGCACTTGAC--TTTCTGTCAAGGTGTCAGGTAAGCACAAGGTCATTA
ATGGCCATGTCCTCC-TTTTCCATTGGTCCTGCTCTAG---GTCCCCTCCCAGATG
>Cya
----------------------------------TGCTGGTCTGGCCCAGTGGCTGGATTGTATTTAT------------
------------GTTTGTGTTGCTGAGTGCAGTGTTCCCTGGATTGTG--------------------------------
--------------------------------------------------------------------------------
-------------------AAG---TGAGGGGGACAGTGTGGGGGCATTTCCAAGATCCTCATATAACTGAGCTTTATCG
GGGCTTAGAGTTTGCAGGGGGCTTCCAGAGAGATGCGATGGCCTCTCACCCTAGCAATGTATCCCTGCATCTGGTGGATT
CAGTGCGTGGGCATTTTGTTTCAAGTCCCATGCAGGAAAGCTGGAGACGATTGTTCAGCAGC-------------CAGAC
ACTGTGCAGAGATAATGTGTCAGTTTGCTTCATGGGCAAATGTGTGTAGGTGTGCCAGTTCTGCTCAGCTGAAGTTCCCA
GTTAAACTGGGAGGGTGTAATGAGAGTGTGGCTGTCCCA--CCTCCATTGCTTAAAGGTTGATCTCCATTGCACCTGTTT
TTAAAGAGGCAGTGACTAGGACTTAC--------------------------------------------------GGGT
CAGGCCCGTAGAATGGGTCT----GCTGTATTCCCACTGTGAGGCTGTACAAA-------------------------CC
TTGCTGTCACCTGGGGCTCCGTGAGATCCCAAAGCACTTGACTGTTTCTATCAAGGTGTCAGGTAAGCACAAGGTCATTA
ATGGCCATGCCCTCCTTTTTCCACTGCTCCTCCTCTAG---GTCCCCTCCCAGAC-
>Asi_533300831
ACTAG---GTAATAGATGGCTGCCATGGAGTTCCTGCTGGTCAGGCCCAGTGGCTGGATTGTATTTAT------------
------------GTTTGTATCGCTGAATGCATTGTTCCCTGGATCGTG--------------------------------
--------------------------------------------------------------------------------
-------------------AAG---TGAGGGGGACATTGTGGGGGCATTTCCAAGATCCTCATATAACTGAGCTTTATCG
GGGCTTAGAGTTTGCAGGGGGCTTCCAGAGAGATGCGATGGCTTCTCACCCTAGCAATGTATCCCTGCATCTGGTGGATT
CAGTGCGTGGGGATTTTGTTTCAAGTCCCATGCAGGAAAGTTGGAGACGATTGTTCAGCAGC-------------CAGAC
ACTGTGCAGAGATAACGTGTCAGTTTGCTCCATGGACAAACATGTGTAGGTGTGCCACTTCTGCTCTGCTGAAATTCCCA
GTTAAACTGGGAGGGTGTAGTGAGAGTGTGGCTGTCACA--CCTCCATTGCTTAAAGGTTGATCTCCATTGCACTTGTGT
TTAAAGAGGCAGTGACTAGGACTTAC--------------------------------------------------GGGT
TGGGCCCATAGAATGGGTCT----GCTGTATTCCCA------------------------------------------CC
TTGCTGTCACCAGGGGCTCCGTGAGATCCCAAAGCACTTGACTGTTTCTATCAAGGTGTCAGGTAAGCACAAGGTCATTA
ATGGTCATGCCCTCC-TTTTCCACTGGTCCTGCTCTAG---GTCCCCTCCCAGACG
>Ami_scaffold-13450
ACTAG---GTACTAGATGGCTGCCATGGAGTTCCTGCTGGTCAGGCCCAGTGGCTGGATTGTATTTAT------------
------------GTTTGTATTGCTGAGTGCAGTGTTCCCTGGATCGCG--------------------------------
--------------------------------------------------------------------------------
-------------------AAG---CGAGGGGGACAATGTGGGGGCATTTCCAAGATCCTCATATAACTGAGCTTTATCG
GAGCTTAGAGTTTGCCGGAGGCTTCCAGAGAGATGCGATGGCTTCTCACCCTAGCAATGTATCCCTGCATCTGGTGGATT
CAGTGTGTGGGGATTTTGTTTCAAGTCCCATGCAGGAAAGTTGGAGATGATTGTTCAGCAGC-------------CAGAC
ACTGTGCAGAGATAACGTGTCAGTTTGCTTCATGGGCAAACGTGTGTAGGTGTGCCACTTCTGCTCTGCTGAAGTTCCCA
GTTAAACTGGGAGGGTGTAATGAGAGTGTGGCTGTCACA--CCTCCATTGCTTAAAGGTTGATCTCCATTGCACTTGTTT
TTAAAGAGGCAGTGACTAGGATTTAC--------------------------------------------------GGGT
CGGGCCCATAGAATGGGTCT----GCTGTATTCCCACTGTGAGGCTGTACAAA-------------------------CC
TTGCTGTCACCAGGGGCTCCGTGAGATCCCAAAGCACTTGACTGTTTCTATCAAGGTGTCAGGTAAGCACAAGGTCATTA
ATGGTCATGCCCTCC-TTTTCCACTGGTCCTGCTCTAG---GGCCCCTCCCAGACG
>Gallus_GB
AATAG---GTGACACGTTTTTCAAGCAAGAATTACATATTTGGGGTTCTGCCCCTCGATTGCAATTTTAGCACGTGAATT
AGGTGGAAGCAGCCTTGCAGGGCTATTTGTAGCAGTCCTTCCTGTGTA--------------------------------
--------------------------------------------------------------------------------
----------------------------------------AGAACGGGTTGTACAGTGTTTGAGTGGGTGAGAA------
-------------------GGGCTTCTGTGAAGTGAGGTAGCTACTG---------------------------------
--------------------------------------TGCTGGGGATGGGTGTTCAACAGTGCTTAGAAACAAACAACA
AAAAAAAAGGGGCACAATATTACCTTGCTT--------------------------------------------------
-------TGCCCATTTGCTCTGAAAATGAAGTCCACATATGCTTTTGCTGTTGAGGACTGGAATTACTTTAATAAACTCA
ATGGAAGGAAAAAAAAAGGAACTGATATGAGAAGAGAGAGGCAGCTGAGTTGTAGTACTGAACATTGTTTCTTACATGTC
TGGAGCCCCAGCAGTGACTTAAGAGCTGTGTGCTTTGTTTTAAGGCATAGATGAGTAAAGATGTCATAGTCTGCGGGCCT
CAGCTGAGACCTGGGAATCCATTGAGTCCTAGAGCAATTGTCT-----TGCATGTGTAACAAGTAAGCATGGTATCACTA
ACCCTGTTGTCTTTCTCTTCC-------CTTGGCTTAG---GTCTCTTACCAGATG
```

CP13

```
>CR1-2C
--------------------------------------------------------------------------------
--------------------------------------------------------------------------------
--------------------------------------------------------------------------------
--------------------------------------------------------------------------------
-------------------------------------TTTCATAGATTTCATAGACATTAGGGCTGGAAGGGACCTCGGA
AGATCATCGAGTCCAGCCCCCCGCCCGAAGGGCAGGAAGTCAGCTGGGGTCATAGGATCCCAGCAAGATAAGCATCCAAT
TTGCTCTTGAAGGTGTTCAATGTAGGTGCTTGAACCACCTCCGATGGCAGGCTGTTCCAGACCTTGGGGGCTCGGACAGT
AAAGAAATTCTTCCTTATGTCCAGCCTGAAACGGTCTTGCAGTAG-TTTATGACCGTTCGACCTCGTCATCCCTTGGGGC
GCTCTGGTGAACAAACGTTCCCCCAGATCCTGGTGGTCACCCCTGATAAACTTATAGGTGGCCATCAGATCACCCCTGAG
CCTGCGCTTTTCCAGGCTAAAGAGCCCCATGGCTCTCAGCCTGTCATCGTAAGGT---------------CTGTTTTCCT
GACCTCTGATCATGCGCGTGGC----------------------------------------------------------
--------------------------------------------------------------------------------
--------------------------------------------------------------------------------
--------------------------------------------------------------------------------
--------------------------------------------------------------------------------
```

```
--------------------------------------------------------------------------------
-----------------------------------------------------
>Exons
TCTGGAGGACATTCGGGAGGAGCTTGCCATGGTTATACTG----------------------------------------
--------------------------------------------------------------------------------
--------------------------------------------------------------------------------
--------------------------------------------------------------------------------
--------------------------------------------------------------------------------
--------------------------------------------------------------------------------
--------------------------------------------------------------------------------
--------------------------------------------------------------------------------
--------------------------------------------------------------------------------
--------------------------------------------------------------------------------
--------------------------------------------------------------------------------
--------------------------------------------------------------------------------
--------------------------------------------------------------------------------
--------------------------------------------------------------------------------
--------------------------------------------------------------------------------
--------------------------------------------------------------------------------
-----------------------------------------------------
>Cpo_3905_100600-102150
TCTGGAGGACATTCGGGAGGAGCTTACCATGATTATACTG---GTAGGCATGAATGCCATTTT-----GAGTCCTGCTAG
GAAACATGATATCTAGCCAGAGGCAAAATCA--TGTTTCAGAAATCTGAACTTGATGTTCT---GTTCTGAAGTTTTACT
TC--AAATTGTCTGAAATCTT-GTTCGGTTGATCTTTTTGCTGTATCTTATTTAAAAAACCTTGTATTTTTGTGAATAAA
GGTAGACAGCTAATTTTTTTTCTTTGATTT-------------------------ATGTCTTCCTTTTCAATGACAAGAT
GTATTTAATGAGAGAG--------------CTTA---TTTCATAGATTTCATAGACATCAGGGCTGGAAGGGACCTCCGG
AG---ATCGAGTCCAGCCCCTTGCCCAAAGGGCAGGAAGTCAGCTGGGGTTATAGGATCCCAGCAAGATAAGCATCCAAT
TTATTCTTGAAGGTGTTCAATGTAGGTGCTTGAACCACCTCCGATGGCAGACTATTCCAGACCTTGGGGGCTTGGACAGT
AAAGAAATTCTTCCTTATGTCCAGCCTGAAACGGTCTTGCAGGAG-TTTATGGCCGTTCGACCTAGTCATCCCTTGGGGC
GCTCTGGTGAACAAACGTTCCCCCAGATCCTGGTGGTCACCCCTGATAAACTTATAGGTGGCCATCAGATCACCCCTGTG
CCTATGCTCTTCCAGGCTAAAGAGTCCCATGTTTCTCAGCCTATCATCGTAAGAT---CTTA---TAT-CAATGCTGCCC
TTAGAAATGTCTTTTGATCTGT---GTTTAATCAAAGACCTATCCTCA--------------------------------
-----------------------------------------------------------------------TTTTTTTTT
TTTTCTGACAA-TTTCCTAATTTAAAGAAAGTACATGCCTGTGTTTTAGTGTAC-TTTCCTCTTCCTCAACAA-CCTTCA
TGTAAGCTCACCATAGACACTCAGTTTAGGGAAAGAGTTTTTAATTACAAG-GCTCTACTGTCATTTTCTTCAGTTAA--
-------GCTGTATGTGAAAGCCATTTGCTACCACAAACAAGAAAGGGCCCATCTAGTTGTTTGATATGAAT-ATA----
------TGCTGGTTCAGCTCAGGTGGAAAGTTCTTTGTACCTT-TTGTTTTGGCTTCCGCTTTAGTTGCACCTTGTTGAA
AC-TTTAAGATCAGTGCTTCAGTCACTGTTATTTGTTTGGGTTTTTATCATAG
>Cno
-----------------------------------TACTG---GTAGGCATGAATGCCATTTT-----GAGTCCTGCTAG
GAAACATGATATCTAGCCAGAGGCAAAATCA--TGTTTCAGAAATCTGAACTTGGTGTTCT---GTTCTGAAGTTTTACT
TC--AAATTGTCTGAAATCTT-GTTCGGTTGATCTTTTTGCTGTATCTTATTTAAAAAACCTTGTATTTTTGTGAATAAA
GGTAGACAGCTAA-TTTTTTTCTTTGATTT-------------------------ATGTCTTCCTTTTCAATGACAAGAT
GTATTTAATGAGAGAG--------------CTTA---TTTCATAGATTTCATAGACATCAGGGCTGGAAGGGACCTCCGG
AG---ATCGAGTCCAGCCCCTTGCCCAAAGGGCAGGAAGTCAGCTGGGGTTATAGGATCCCAGCAAGATAAGCATCCAAT
TTATTCTTGAAGGTGTTCAATGTAGGTGCTTGAACCACCTCCGATGGCAGACTATTCCAGACCTTGGGGGCTTGGACAGT
AAAGAAATTCTTCCTTATGTCCAGCCTGAAACGGTCTTGCAGGAG-TTTATGGCCGTTCGACCTAGTCATCCCTTGGGGC
GCTCTGGTGAACAAACGTTCCCCCAGATCCTGGTGGTCACCCCTGATAAACTTATAGGTGGCCATCAGATCACCCCTGTG
CCTATGCTCTTCCAGGCTAAAGAGTCCCATGTTTCTCAGCCTATCATCGTAAGAT---CTTA---TAT-CAATGCTGCCC
TTAGAAATGTCTTTTGATCTGT---GTTTAATCAAAGACCTATCCTCA--------------------------------
-------------------------------------------------------------------------TTTTTTT
TTTTCTGANAA-TTTCCTAATTTAAAGAAAGTACATGCCTGTGTTTTAGTGTAC-TTTCCTCTTCCTCAACAA-CCTTCA
TGTAAGCTCACCATAGACACTCAGTTTAGGGAAAGAGTTTTTAATTACAAG-GCTCTACTGTCATTTTCTTCAGTTAA--
-------GCTGTATGTGAAAGCCATTTGCTACCACAAACAAGAAAGGGCCCATCTAGTTGTTTGATATGAAT-ATA----
------TGCTGGTTCAGCTCAGGTGGAAAGTTCTTTGTACCTTTTTGTTTTGGCTTCCGCTTTAGTTGCACCTTGTTGAA
AC-TTTAAGA-------------------------------------------
>Ote
-----------------------------TGATTATACTG---GTAGGCATGAATGCCATTTT-----GAGTCCTGCTAG
GAAACATGATATCTAGCCAGAGGCAAAATCA--TGTTTCAGAAATCTGAACTTGGTGTTCT---GTTCTGAAGTTTTACT
TC--AAATTGTCTGAAATCTT-GTTTGGTTGATCTTTTTGCTGTATCTTATTTAAAAAACCTTGTATTTTTGTGAATAAA
GGTAGACAGCTAATTTTTTTTCTTTGATTT-------------------------ATGTCTTCCTTTTCAATGACAAGAT
GTACTTAATGAGAGAG--------------CTTA---TTTCATAGATTTCATAGACATCAGGGCTGGAAGGGACCTCCAG
AGATCATCGAGTCCAGCCCCTTGCCCAAAGGGCAGGAAGTCAGCTGGGGTTATAGGATCCCAGCAAGATAAGCATCCAAT
TTATTCTTGAAGGTGTTCAGTGTAGGTGCTTGAACCACCTCCGCTGGCAGACTGTTCCAGACCTTGGGGGCTCGGACAGT
AAAGAAATTCTTCCTTATGTCCAGCCTGAAACAGTCTTGCAGGAGTTTTATGGCCATTCGACCTGGTCATCCCTTGGGGC
GCTCTGGTGAACAAACGTTCCCCCAGATCCTGGTGGTCACCCCTGATAAACTTATAGGTGGCCATCAGATCACCCCTGTG
CCTACGCTTTTCCAGGCTAAGGAGTCCCATGTTTCTCAGCCTATCATCGTAAGAT---CTTA---TAT-CAATGCTGCCC
TTAGAAATGTCTTTTGATCTGT---GTTTAATCAAAGACCTATCCTCA--------------------------------
-----------------------------------------------------------------------TTTTTTTTT
TTCCCTGACAATTTTCCTAATTTAAAGAAAGTACATGCCTGTG-TTTAGTGTAC-TTTCCTCTTCCTCAACAA-CCTTCA
TGTAAGCTCACCATAGACACTCAGTTTAGGGAAAGAGTTTTTAATTACGAGAGCTCTACTGTCATTTTCTTCAGTTAA--
-------GCTGTGTGTGAAAGCCATTTGCTACCACAAACAAGAGAAGGCCCATCTAGTTGTTTGATATGAAT-ATA----
------TGCTGGTTCAGCTCAGGTGGAAAGTTCTTTGTACCTT-T-ATTTTGGCTTCCACTTTAGTTGCACCTTGTTGAA
AC-TTTAAGATCAGTGCTCAGTCA-----------------------------
>Mca
-----------------------------------TACTG---GTAGGCATGAATGCCATTTT-----GAGTCCTGCTAG
GAAACATGATATCTAGCCAGAGGCAAAATCA--TGTTTCAGAAATCTGAACTTGGTGTTCT---GTTCTGAAGTTTTACT
TC--AAATTGTCTGAAATCTT-GTTTGGTTGATCTTTTTGCTGTATCTTATTTAAAAAACCTTGTATTTTTGTGAATAAA
```

```
GGTAGACAGCTAA-TTTTTTTCTTTGATTT-------------------------ATGTCTTCCTTTTCAATGACAAGAT
GTATTTAATGAGAGAG--------------CTTA---TTTCATAGATTTCATAGACATCAGGGCTGGAAGGGACCTCCAG
AGATCATCGAGTCCAGCCCCTTGCCCAAAGGGCAGGAAGTCAGCTGGGGTTATAGGATCCCAGCAAGATAAGCATCCAAT
TTATTCTTGAAGGTGTTCAATGTAGGTGCTTGAACCACCTCCGATGGCAGACTGTTCCAGACCTTGGGGGCTCGGACAGT
AAAGAAATTCTTCCTTATGTCCAGCCTGAAACGGTCTTGCAGGAG-TTTATGGCCGTTCGACCTGGTCATCCCTTGGGGC
GCTCTGGTGAACAAACGTTCCCCCAGATCCTGGTGGTCACCCCTGATAAACTTATAGGTGGCCATCAGATCACCCCTGTG
CCTACACTTTTCCAGGCTAAAGAGTCCCATGTTTCTCAGCCTATCATCGTAAGAT---CTTA---TAT-CAATGCTGCCC
TTAGAAATGTCTTTTGATCTGT---GTTTAATCAAAGACCTATCCTCA--------------------------------
---------------------------------------------------------------------------TTTTT
TTTTCTGACAA-TTTCCTAATTTAAAGAAAGTACATGCCTGTGTTTTAGTGTAC-TTTCCTCTTCCTCAACAA-CCTTCA
TGTAAGCTCACCATAGACGCTCAGTTTAGGGAAAGAGTTTTTAATTACGAGAGCTCTACTGTCATTTTCTTCAGTTAA--
-------GCTGTGTGTGAAAGCCATTTGCTACCACAAACAAGAAAGGGCCCATCTAGTTGTTTAATATGAAT-ATA----
------TGCTGGTTCAGCTCAGGTGGAAAGTTCTTTGTACCTT-T-GTTTTGGCTTCCGCTTTAGTTGCACCTTGTTGAA
AC-TTTAAGATCTGTGCTCAGTC------------------------------
>Gga_scaffold18450
TCTGGAGGACATTCGGGAGGAGCTTACCATGGTTATACTG---GTAGGCATGAATGCCATTTT-----GAGTCCTGCTAG
GAAACATGATATCTAGCTAGAGGCAAAATCATATGTTTCAGAAATCTGAACTTGGTGTTCT---GTTCTGAAGTTTTACT
TC--AAATTGTCTGAAATCTT-GTTTGGTTGATCTTTTTGCTGCATCTTATTTAAAAATCCTTGTATTTTTGTGAATTAA
GGTAGACAGCTAA-TTTTTTTCTTTGATTT-------------------------ATGTCTTCCTTTTCAGTGACAAGAT
ATATTTAATGAGAGAG----------------------------------------------------------------
--------------------------------------------------------------------------------
--------------------------------------------------------------------------------
--------------------------------------------------------------------------------
--------------------------------------------------------------------------------
----------------------------------------------------------CTTA---TAT-CAGTGCTGCCC
TTAGAAATGTCTTTTGATCTGT---GTTTAATCAAAGACCTATCCTCATTTTTTTTTTTTTTTTAATTTTTTTTTTTTTTT
AATTTTTTTTTTTTTTTTAATTTTTTTTTTTTTTTTAATTTTTTTTTTTTTTTTAATTTTTTTTTTTTTTTTAATTTTTTTTTTT
TTTTCTGACAA-TTTCCTAATTTAAAGAAATTACATGCCTGTGTTTTAGTGTACTTTTCCTCTTCCTCAGCAA-CCTTCA
TGTAAGCTCACCATAGACACTCAGTTTAGCGAAAGAGTTTTTAATTACGAAAGCTCTACTGTCATACAGCTTAACTGA--
-------AGAAAATGTGAAAGCCATTTGCTACCACAAGCAAGAAAGTGCCCATCTAGTTGTTTGATATGAAT-ATACCCA
TACAGTTGCTGGTTCAGCTCAGGTGGAAAGTTCTTTGTACCT--TTGTTTTGGCTTCCGCTTTAGTTGCACCTTGTTGAA
AC-TTTAAGATCAATGCTTCAGTCACTGTTAATTGTTTGGGTTTTTATCATAG
>Cya
TNTGGAGGACATTCGGGAGGAGCTTGCCATGGTTATACTG---GTAGGCATGAATGCCATTTA-----GAGTCTTGCTAG
GAAACATGATATCTAGCCAGAGGCAAAATCACTTGCTTTAG-AATTTGAACCTGGTGTTCC---GTTNTGAAGTTTTACT
TC--AAATTGTTTGAAATCTT-TTTTGATTGATCTTTTTGCTGCATGTTATCTAAAAATCCTTGTATTTTTGTGAATTAA
GGTAGACAGCTAA--TTTTGCCTTTGATTT-------------------------ATGTCTTCCTTTTCAGTGACAAGAT
GCATTTAATGAGAGAG----------------------------------------------------------------
--------------------------------------------------------------------------------
--------------------------------------------------------------------------------
--------------------------------------------------------------------------------
--------------------------------------------------------------------------------
----------------------------------------------------------CTTA---TAT-CAATGCTGCCC
TTAGACATGTT-TTTGATTTGT---GTTTAATCAAAGACTTATCCTCA--------------------------------
-------------------------------------------------------------------------TTTTTTT
TTTTCTGACAG-TTTCTTAATTTAAAGAAATTACATGCATGTGTTTTAGTGTAC-TTTCCTCTTCCTCAATAA-CTTCCA
TGTAAGATCACCATAGCCACTCAGTTTAGGGAAAGAGTTTTTAACTAAGAAAGCTCTAACGTCATTTTCTTTAGTTAA--
-------GCTGTATGTGAAAGCCATTTGCTACCGCAAGCAAGAAAGTGCCCATCTAGTTGTTTGGTCTGAAT-ATACCCA
TACAGTTGCTGGTTCAGCTCGGGTGGAAAGTTCTTTGTACCTTTTTGTTTTGGCTTCAGCTTTAGTTTCACTTTGTTGAA
AC-TTTAAAATCAATGCTTCAGTCACTGTTAATTCTTTGGGTTTTTGTCATAG
>Asi_533246941
TCTGGAGGACATTCGGGAGGAGCTTGCCATGGTTATACTG---GTAGGCATGAATGCCATTTA-----GAGTCCTGCTAG
GAAACATGATATCTAGCCAGAGGCAAAATCGCATGCTTTAG-AATCTGAACCTGGTGTTCC---GTTCTGAAGTTTTACT
TC--AAATTGTCTGAAATCTT-TTTTGGTTGGTCTTTTTGCTGCATCTTATTTAAAAATCCTTGTATTTTTGTGAATTAA
GGTAGACAGCTAA--TTTTGTCTTTGATTT-------------------------ATGTCTTCCTTTTCAGTGACAAGGT
GTATTTAATGAGAGAG----------------------------------------------------------------
--------------------------------------------------------------------------------
--------------------------------------------------------------------------------
--------------------------------------------------------------------------------
--------------------------------------------------------------------------------
----------------------------------------------------------CTTA---TAT-CAATGCTGCCC
TTAGACATGTC-TTTGATCTGT---GTTTAATCAAAGACCTATCCTCA--------------------------------
-----------------------------------------------------------------------------TTT
TTTTCTGACAG-TTTCCTAATTTAAAGAAATTACATGCATGTGTTTTAGTGTAC-TTTCCTCTTCCTCAACAA-CCTTCA
TGTAAGCTCACCATAGACACTCAGTTTAGGGAAA----------------------------------------------
--------------------------------------------------------------------------------
--------------------------------------------------------------------------------
-----------------------------------------------------
>Ami_scaffold-12431
TCTGGAGGACATTCGGGAGGAGCTTGCCATGGTTATACTG---GTAGGCATGAATACCATTTA-----GAGTCCTGCTAG
GAAACATGATATCTAGCCAGAGGCAAAATCACATGCTTTAG-AATCTGAACCTGGTGTTCT---GTTCTGAAGTTTTACT
TC--AAATTGTCTGAAATCTT-TTTTGGTTGGTCTTTCTGCTGCACCTTATTTAAAAATCCTTGTATTTTTGTGAATTAA
GGTAGACAGCTAA--TTTTGTCTTTGATTT-------------------------ATGTCTTCCTTTTCAGTGACAAGAT
GTATTTAACAAGAGAG----------------------------------------------------------------
--------------------------------------------------------------------------------
--------------------------------------------------------------------------------
--------------------------------------------------------------------------------
--------------------------------------------------------------------------------
```

```
----------------------------------------------------------CTTA---TAT-CAATGCTGCCC
TTAGACATGTC-TTTGATCTGT---GTTTAATCAAAGACCTATCCTCA--------------------------------
-----------------------------------------------------------------------------CTT
TTTTCTGACAG-TTTCCTAATTTAAAGAAATTACATGCATGTGTTTTAGTGTAC-TTTCCTCTTCCTCAACAA-CCTTCA
TGTAAGCTCACCATAGACACTCAGTTTAGGGAA--AGTTTTTAATTAAGAAAGCTCTACTCTCATTTTCTTCAGTTAA--
-------GCTGTATGTGAAAGCCATTTGCTACCACAAGCAAGAAAGTGCCCATCTAGTTGTTTGGTCTGAAT-ATACCCA
TACAGTTGCTGGTTCAGCTCAGGTGGAAAGTTCTTTGTACCTTTTTGTTTTGGCTTCAGCTTTAGTTGCACCTTGTTGAA
AC-TTAAAGATCAATGCTTCAGTCACTGTTAATTGTTTGGGTTTTTATCATAG
>Gallus_GB
CCTGGAGGATGTTCGGGAGGAGCTGACCATGGCAATATTG---GTATGTGTTAATGTTACTTTTGAAGGGGTTTTTTTGC
A---CTTACCTCTATGCAGGAAATAAAACTATTTATGTAAG----TTTGTTTGTATTTATTTAATTTATAATGTGTTGCT
CATGAGA-----------------------------TCTAGCATGCTACAAACTAGGAATATTGCAACATCTTGATTTAA
AATAGACTTTTATAATTTGCTTTCTGGTTTGTTACAAGTCTGGTGCATATATTAAATACTTCATTTTTTGTATTCAAGAT
TTGATTTTTTGGTATGTTCATTATTAC-----------------------------------------------------
----------------------------------------------------------TGCATCTTAAGTATTCTAAAAG
TT------------------------TTCTACTGATAACCTTAGTTAGACAGTTGCTATATGGGGGGGAAAAAAA-----
---------------------------CAAAAACACTGAGACTGCTC---------------------------------
--------------------------------AATAGCCTTGCTGCTGGTCATAA-------------------------
-----------------------------------------------------------------CATAGAATCATTGAG
GCTGGAAAAGACTCTAGGATCACGTAGTCCAACTGTTGGCCCATCCCCA-------------------------------
--------------------------------------------------------------------------------
--CCATGACTGCTATCTCA--------------------TGTCCCTCAGTGCCCCATCTCCATGGCTTGTGAACACCTCC
AGGGACTTCACCA---------CCTTCCTGGGCAGCCTGT------GCCAGTGCCTCACCACTCTTGTCTCTGAATAATG
ATGCATTTCTCAATTTGGGGGGTATTCATCAATGCAAGATAATAAGATGCCAGTTGACTGGCTGAGTTTATTCATT----
-----TAACCTGTAAAGAGTTAGAATAAAGTACTTGGTTATAAATGAATTTTCTTCCAGCTGTAACTGCAACAT------
------------TGGCTTTTTGTCACCGATAACCATGATAATTTATTTCATAG
```

CP14

```
>CR1-7B
--------------------------------------------------------------------------------
--------------------------------------------------------------------------------
--------------------------------------------------------------------------------
--------------------------------------------------------------------------------
--------------------------------------------------------------------------------
--------------------------------------------------------------------------------
--------------------------------------------------------------------------------
--------------------------------------------------------------------------------
-------------------------------------------------------TAGTTT---CATAGT---TTCATAG
TTTCTAGGGTCGGAAGGGACCTGAGCAGATCATCTAGTCCGACCCCCTGCCATGGCAGGAAAGAGTACTGGGGTCAAACG
ACCCCAGCAAGGTGTTCATCTA--------------------------------ACCTCCTCTTAAAAACCCCCAGGGTA
GGAGCCTGCACCACCTCCCTTGGAAGTTGGTTCCAGACCCTAGCCGCCC-------------------------------
-----------------------------------------------------------
>Exon
AGATCTTGCCAGGGACTTGGCAAATGACATCATGACTCTG----------------------------------------
--------------------------------------------------------------------------------
--------------------------------------------------------------------------------
--------------------------------------------------------------------------------
--------------------------------------------------------------------------------
--------------------------------------------------------------------------------
--------------------------------------------------------------------------------
--------------------------------------------------------------------------------
--------------------------------------------------------------------------------
--------------------------------------------------------------------------------
--------------------------------------------------------------------------------
--------------------------------------------------------------------------------
-----------------------------------------------------------
>Cpo_3930_116650-118200
AGATCTTGCCAGGGACTTGGCAAATGACATCATGACTCTG---GTGAGT---TGAGAC--AAACTAGCTCCCT-------
-ATTTCTTGAGGCCTAGACTGTGTTTTAAGCTG-TTTTATAAATTTACTGTTCCCA-----ATAGAATGTAGGACTACAA
TGCCTGAAACAGTCGTAATTTTCATTTTAATACTTCTGATGTTTGAGAGCTGTACTGCTATAATTTACATAAATCTCTGG
CAGTCCAAGTCCCAAATCAACAAACTAATTGTGCTTTCTGAGTTCAGTTTAAAACATTTTCAAAATGGGACTACTTCCCA
TCTGAATCAAGTACACGACGTAA-TTTGTTAAGCATGAAATGTGCTGACATTACTATGTTGGACCTGGTAATATGGCTTT
CCACATCACTTCTGTCACCTTACAATGATCAGATTAGTTTTTCAGGAGGAAAACAGTCTTGAATCTAACAGATGATGTTT
CTGCTCCAGGATGTGTTCTCTGACATAAATGGAGATTTTT--T-GGTACTTTTCTCCTCTTATAGTGTGTGCCGTTTGAA
GTCTTGTAGGCACTTAGATGATTTAGATGGGTGTGCAGAACCAAGATGCAATTATGTTCTGACCCAAACTAGTCATATAG
GGTAAAGTAAAACATATAAGTGAATTTTGGCTTTTATACGTCTTTTAAATATTATCAACTT---AATAGT---TTCATAG
TTTGTAGGGTCGGAAGGGACCTGAGCAGATCATCTAGTCCGATCCCCTGCCATGGCAGGAAAGAATACTGGGGTCAAACG
ACCCCCGCAAGATGTTCATCTATGTTGTG---AATGGT---GAAGATGTTATCCATGTATTTAATGAAGATGTCGATTTA
ACCAGATTTTTCTCTGGGTTATTTTATTCCCCTGTTCTCAGTCATTTCACTGCGGTCACTAATTGATGCAGTGTGCTTGA
CTGAAAGATAGTAATTGAAGTC-TTCATATTCTACAAGGTA--AATGACAACCTAGAGG
>Cno
AGATCTTGCCAGGGACTTGGCAAATGACATCATGACTCTG---GTGAGT---TGAGAC--AAACTAGCTCCCT-------
-ATTTCTTGAGGCCTAGACTGTGTTTTAAGCTG-TTTTATAAATTTACTGTTCNCA-----ATAGAATGTAGGACTACAA
TGCCTGAAACAGTCGTAATTTTCATTTTAATACTTCTGATGTTTGAGAGCTGTACTGCTATAATTTACATAAATCTCTGG
CAGTCCAAGTCCCAAATCAACAAACTAATTGTGCTTTCTGAGTTCAGTTTAAAACATTTTCAAAATGGGACTACTTCCCA
TCTGAATCAAGTACATGACGTAA-TTTGTTAAGCATGAAATGTGCTGACATTACTATGTTGGACCTGGTAATACGGCTTT
CCACATCACTTCTGTCACCTTACAATGATCAGATTAGTTTTTCAGGAGGAAAACAGTCTTGAATCTAACAGATGATGTTT
CTGCTCCAGGATGTGTTCTCTGACATAAATGGAGATTTTT--T-GGTACTTTTCTCCTTTTATAGTGTGTGCCATTTGAA
```

```
GTCTTGTAGGCACTTAGATGATTTAGATGGGTGTGCAGAACCAAGATGCAATTATGTTCTGACCCAAACTAGTCATATAG
GGTAAAGTAAAACATATAAGTGAATTTTGGCTTTTATACGTCTTTTAAATATTATCAACTT---AATAGT---TTCATAG
TTTGTAGGGTCGGAAGGGACCTGAGCAGATCATCTAGTCCGATCCCCTGCCATGGCAGGAAAGAATACTGGGGTCAAATG
ACCCCAGCAAGATGTTCATCTATGTTGTG---AATGGT---GAAGATGTTATCCATGTATTTAATGAAGATGTCGATTTA
ACCAGATTTTTCTCTGGGTTATTTTATTCCCCTGTTCTCAGTCATTTCACTGCGGTCACTAATTGATGCAGTGTGCTTGA
CTGAAAGATAGTAATTGAAGTC-TTCATATTCTACAAGGTA--AATGACAACCTAGAGG
>Ote
--------------------------ACATCATGACTCTG---GTGAGT---TGAGACAGAAACTAGCTCCCT-------
-ATTTCTTGAGGCCTAGACTGTGTTTTAAGCTG-TTTTATAAATTTACTGTTCCCA-----ATAGAATGTAGGACTACAA
TGCCTGAAACAGTCGAAATTTTCATTTTAATACTTCTGGTGTTTGAGAGCTGTACTGCTATAATTTACATAAATCTCTGG
CAGTCCAAGTCCCAAATCAACAAACTAATTGTGCTTTCTGAGTTCAGTTTAAAACATTTTCAAAATGGGACTACTTCCCA
TCTGAATCAAGTACATGACGTAA-TTTTTTAAGCATGAAATGTGCTGACATTACTATGTTGGACCTGGTAATACGGCTTT
CCACATCACTTCTGTCACCTTACAATGATCAGGTAAGTTTTTCAGGAGAAAAACATCTTTGATTCTAACAAATAATGTTC
CTGCTCTAGGAGGGGTCCTCTAACTTAAAGGAAAATTTTTTTTGGGTCCTTTCCCCCTTTAATGGGGGGGGCGGTTGAAA
GTCTTGTAGGCACTTAGATGATTTAGATGGGTGTGCAGAACCAAGATGCAATTATATTCTGACCCAAACCAGTCATATAG
GGTAAAGTAAAACATATAAATGAATTTTGGCTTTTATACGTCTTTTAAATATTATCAACTT---AATAGT---TTCATAG
TTTGTAGGGTTGGAAGGGACCTGAGCAGATCATCTAGTCCGACCCTCTGCCATGGCAGGACCGAATACCCGGGTCAAATG
ACCCCAGCAAGATGTTCATCTATCTTGTG---AATGGT---GAAGATGTTATCCATGTATTTAATGAAGATGTCGCTTTA
ACCAGATTTTTCTCTGGGATCTTTTATTCCCCTGTTCTCAGTCATTTCACTGCCGTCACTAACTGATGCAGTGTGCTTGA
CTGAACAGAT-------------------------------------------------
>Mca
AGATCTTGCCAGGGACTTGGCAAATGACATCATGACTCTG---GTGAGT---TGAGACAGAAACTAGCTCCCT-------
-ATTTCTTGAGGCCTAGACTGTGTTTTAAGCTG-TTTTATAAATTTACTGTTCCCA-----ATAGAATGTAGGACTACAA
TGCATGAAACAGTTGTAATTTTCATTTTAATACTTCTGGTGTTTGAGAGCTGTACTGCTATAATTTACATAAATCTCTGG
CAGTCCGAGTCCCAAATCAACAAACTAATTGTGCTTTCTGAGTTCAGTTTAAAACATTTTCAAAATGGGACTACTTCCCA
TCTGAATCAAGTACCCGACGTAA-TTTTTTAAGCATGAAATGTGCTGACATTACTATGTTGGACCTGGTAATACAGCTTT
CCACATCACTTTTGTCACCTTACAATGATCAGATTAGTTTTTCAGGAGGAAAACAGTCTTGAATCTAACAGATGATGTTT
CTGCTCTAGGATGTGTTCTCTGACATAAATGGAGATTTTTT--TGGGTACTTTTCTCCTTTTATTGTGTGTGCTGTTTGAA
GTCTTGTAGGCACTTAGATGATTTAGATGGGTGTGCAGAACCAAGATGCAATTATATTCTGACCCAAACCAGTCATATAG
GGTAAAGTAAAACATATAAATGAATTTTAGCTTTTATACGTCTTTTAAATATTATCAACTT---AATAGT---TTCATAG
TTTGTAGGGTTGGAAGGGACCTGAGCAGATCATCTAGTCCGACCCCCTGCCATGGCAGGAAAGAATACTGGGGTCAAATG
ACCCCAGCAAGATGTTCATCTATGTTGTG---AATGGT---GAAGATGTTATCCATGTATTTAATGAAGATGTCGATTTA
ACCAGATTTTTCTCTGGGATATTTTATTCCCCTGTTCTCAGTCATTTCACTGCGGTCACTAATTGATGCAGTGTGCTTGA
CTGAAAGATAGTAATTGAAGTC-TTCATATTCTACAAGGTA--AATGACNACCTAGAGG
>Tsc
AGATCTTGCCAGGGACTTGGCAAATGACATCATGACTCTG---GTGAGT---TGAGACAGAAACTAGCTCCCTATTTCTG
GATTTCTTGGGGCCTAGACTGTGTTTTAAGCTG-TTTTATAAATTTACTGTTCTCA-----ATAGAATGTAGGACTACTA
TGTCTGAAACGGTCGTAATTTTCATTTTAATACTTCTGGTGTTTGAGAGCTGTACTGCTATAATTTACATAAATCTCTGG
CAGTCCAAGTCCCAAATCAACAAACTAATTGTGCTCCCTGAGTTCAGTTTAAAACATTTTCAAAATGGGACTATTTCCCA
TCTGAATCAAGTACATGATGCAATTTTTTTAAGCATGAATTGTGCTGACATGACTATGTTGGACCTGGTAATACGGCTTT
CCACATCACTTCTGTCACCTTACAATGATCAGATTAGTTTTTCAGGAGGAAAACAGTCTTGAATCTAACGGATGATGTTT
CTGCTTCAGGATGTGTTCTCTGACATAAATGGGGATTTTT--TTGGTACTTTTCTCCTTTTACTGTGTGTGCCCCTTTGAA
GTCTTGTCGGCACTTAGATGATTTAGATGGGTTTGCAGAACCAAGATGCAATTATATTCTGACCCAAACCAGTCATATAG
GGTAAAGTAATACATATAAATGAATTCTGGCTTTTATACATCTTTTAAATATTATCAACAT-------------------
--------------------------------------------------------------------------------
-------------------------------AATGGT---GAAGATGTTATCCATGTATTTAATGAAAATGTCGATTTA
ACCAGATTTTTCTCTGGGATATTTTATTCCCCTGTTCTCAGTCATTTCACTGCGGTCACTAATTGATGCAGTGTGCTTGA
CTGAAAGATAGTAATTGAAGTCTTTCATATTCTACAAGGTG--AATGACAGCCTANAGG
>Gga_scaffold8751
AGATCTTGCCAGGGACTTGGCAAATGACATCATGACTCTG---GTGAGT---TGAGACAGAAACTAGCTCCCTATTTCTG
GATTTCTTGGGGCCTAGACTGTGTTTTACACTG-TTTTATAAATTTACTGTTCCCA-----ATAGAATGTAGGACTACAA
TGCCTGAAACGGTCGTAATTTTCATTTTAATACTTCTGGGGTTTGAGAGCTGTACTGCTATAATTTACATAAATCTCTGG
CAGTCCAAGTCCCAAATCAACAAACTAATTGTGCTCCCTGAGTTCAGTTTAAAACATTTTCAAAATGGGACTATTTCCCA
TGTGAATCAAGTACATGATGCAATTTTTTTAAGCATGAATTGTGCTGACATTACTATGTTGGACCTGGTAATACGGCTTT
CCACATCACTTCTGTCACCTTACAATGATCAGATTAGTTTTTCAGGAGGAAAACAGTCTTGAATCTAACGGATGATGTTT
CTGCTTCAGGATGTGTTCTCTGACATAAATGGGGATTTTT--TTGGTACTTTTCTCCTTTTACTGTGTGTGCCCCTTTGAA
GTCTTGTCGGCACTTAGATGATTTAGATGGGTTTGCAGAACCAAGATGCAATTATATTCTGACCCAAACCAGTCATATAG
GGTAAAGTAATACATATAAATGAATTCTGGCTTTTATACATCTTTTAAATATTATCAACAT-------------------
--------------------------------------------------------------------------------
-------------------------------AATGGT---GAAGATGTTATCCATGTATTTAATGAAAATGTCAATTTA
ACCAGATTTTTCTCTGGGATATTTTATTCCCCTGTTCTCAGTCATTTCACTGCGGTCACTAATTGATGCAGTGTGCTTGA
CTGAAAGATAGTAATTGAAGTC-TTCGTATTCTACAAGGTG--AATGACAGCCTAGAGG
>Cya
AGATCTTGCCAGGGACTTGGCAAATGACATCATGACTCTG---GTGAGT---TGAGACAGAAGCTAGCTCCCTATTTCTG
GATTTCTTGGGGGCTAGATTATGTTTTAAGCTGTTTTTATAAATTTACTGTTCCCA-----ATAGTATGTAGGACTACAA
TGCCTGAAACAGTTGTAATTTTCATTTTAATACTTCTGGTGTTTGAGAACTGTACTGCTATAATTTGCATTAATCTCTGG
CAGTCCAAGTCCCAAATCAGCAAACTAATTGTGCTCCCTGTGTTCAGTTTAAAACATTTTGAAAATGGGACTACTTTCCA
TCTGAATCAAGTACACGATGCAA-TTTTTTAAGCATGAATT---------------TCTTGGACCTGGTAATACAGCTTT
CCACGTCACTTCTGTCACCTTACAATTATCAGATTGTTTTCCTCCTGAAAAACTAATCTTGAACCTAACAGATGTTGTTT
CTGCTCCAGGATGTGTTCTCTGACACACGTGGGGATTTTT--T-GGTACTTTCCTCCTTTTATTTTGTGTGCCCTTTGAA
GTCTTGTCAGGACTTAGATGATTTAGATGGGTTTGCAGAACCAAGGTGCAATTATATTCTGACCCAAACCAGTCGTTTAG
GGTAAAGTAATACATATAAATGAACTCTGGCTTTTATACATCTTTAAATTATTACCAACAT-------------------
--------------------------------------------------------------------------------
-------------------------------AATGGT---GAAGAAGTTATCCATGTGTTTAATGAAGATGTCAGTTTA
ACCAGATTTTTCTCTGGGATATTTTATTCCCCTGTTCTCAGTCATTTCACTGCAGTCACTAATTGATGCAGCGTGCTTGA
CTGAAAGATAGTAATTGAAGTCTTTCATATTCTACAAGGTGGAAATGGNAATCTAGAGG
>Asi_533311147
AGATCTTGCCAGGGACTTGGCAAATGACATCATGACTCTG---GTGAGT---TGAGACAGAAACTAGCTCCCTATTTCTG
```

GATTTCTTGGGGGCTAGACTGTGTTTTAAGCTGTTTTTATAAATGTACTGTTCCCA-----ATAGTATGTAGGACTACAA
TGCCTGAAACAGTTGTAATTTTCATTTTAATACTTCTGGTGTTTGAGAGCTGTACTGCTATAATTTGCATTAATCTCTGG
CAGTCCAAGTCCCAAATCAGCAAACTAATTGTGCTCCCTGTGTTCAGTTTAAAACATTTTGGAAATGGGACTACTTTCCA
TCTGAATCAAGTACATGATGCAA-TTTTTTAAGCATGAATT---------------GCTTGGACCTGGTAATACAGCTTT
CCACGTCACTTCTGTCACCTTACAATGATCAGATTAGTTTTTCAGGAGGAAAACAATCTTGAACCTAACAGATGATGTTT
CTGCTCCAGGATGTGTTCTCTGACACAAGTGGGGATTTTT--T-GGTACTTTCCTCCTTTTATTTTGTGCGCCCTTTGAA
GTCTTGTCGGCACTTAGATGATTTAGATGGGTTTGCAGAACCAAGATGCAATTATATTCTGACCCAAACCAGTCGTATAG
GGTAAAGTAATACATATAAATGAACTCTGGCTTTTATACATCTTTAAAATATTATCAACAT-------------------
----
--------------------------------AATGGT---GAAGAAGTTATCCATGTGTTTAATGAAGATGTCGATTTA
ACCAGATTTTTCTCTAGAATATTTTATTCCCCTGTTCTCAGTCATTTCACTGCGGTCACTAATTGATGCAGCGTGCTTGA
CTGAAAGATAGTAATTGAAGTC-TTCGTATTCTACAAGGTG--AATGGCAAGCTAGAGG
>Ami_scaffold-772
AGATCTTGCCAGGGACTTGGCAAATGACATCATGACTCTG---GTGAGT---TGAGACAGATACTAGCTCCCTATTTCTG
GATTTCTTGGGGGCTAGAATGTGTTTTAAGCTGTTTTTATAAATTTACTGTTCCCA-----ATAGTATGTAGGACTACAA
TGCCTGAAACAGTTGTAATTTTCATTTTAATACTTCTGGTGTTTGAGAGCTGTGCTGCTATAATTTGCATTAATCTCTGG
CAGTCCAAGTCCCAAATCAGCAAACTAATTGTGCTCCCTGCGTTCAGTTTAAAACATTTTGAAAATGGGACTACTTCCCA
TCTGAATCAAGTACATGATGCAG-TTTTTTAAGCATGAATT---------------GCTTGGACCTGGTAATCCAGCTTT
CCACATCACTTCTGTCACCTTACAATGATCAGATTGTTTTCCTCCTAAAAAACTAATCTTGAACCTAACAGATAATGTTT
CTGCTCCAGGATGTGTTCTCTGACACAAGTGGGGATTTTT--T-GGTACTTTCCTCCTTTTATTTTGTGTGCCCTTTGAA
GTCTTGTCTGCACTTAGATGATTTAGATGGGTTTGCAGAACCAAGATGCAATTATATTCTGACCCAAACCAGTCGTATAG
GGTAAAGTAATACATATAAATGAACTCTGGCTTTTATACATCTTTAAAATATTATCAGCAT-------------------
----
--------------------------------AATGGT---GAAGAAGTTATCCATGTGTTTAATGAAGATGTCGATTTA
ACCAGATTTTTCTCTAGAATATTTTATTCCCCTGTTCTCAGTCATTTCACTGCGGTCACTAATTGATGCAGCATGCTTGA
CTGAAAGATAGTAATTGAAGTC-TTCATATTCTACAAGGTG--AATGGCAAGCTAGAGG
>Gallus_GB
AGATCTTGCCAGGGACTTGGCAAACGACATCATGACCTTG---GTGAGTCAGTGAGACTGAAATAACTTC----------
---CTTCCTGTAGTCAAAGAACGT----------
--TAAATTGTTCAAATCTG---

-------------TGTGATTTAGAACTGCTATATGTGAAAAGTGA-----------------------------------
----
--TCCTTCTTCCTTGTGAGCCCTTTGGG
G-------AGCCCTTCCAGGCTTGAGTTAGTTCTGGAGGT--

--TTCCGTTGGTTGTTTTTTAGTGGAGGTGATAGATGC
CAGCCCAG---

CP15

>CR1-8

-------------------------------AG---TTTCATAGTTTCATAGATGTTAGGGTCGGAAGGGACCTAT-TAG
ATCATCGAGTCCGACCCCCTGCCCTGGGCAGGAATGAGTGCTG---GGGTCAG-ATGACCCCAGCCAGGTGTCTGTCC--
----

>Exons
ACTTGTTACG--

>Cpo_4909_56950-58500
ACTTGTTACG---GTAACTTTTTCTAACAAAAAACTCCTTCA----------AGGGTCATATGGCGTGCTATA----GAA
TCTAG--GAGTGCTGACTGCTTGCAGGGGGATGAGGG---
-GGAGGTATTGATTTGTATGTCATCTACTGAGCTCCTTCCTAGT--AAAGTGTGACTGCTTCCCAAAAATGTCAAAAGCT
TTAATTTCATCTTGTTCCTTGAATGGTGGAAGTTAGCTTGAGACGTTTTCAGAACT------------------------
----------------TTACTTTATAGATGAGTGCAAAGTGGAAGCCTTTTTGCTGTTTGAATTGATAGTGGAAACTGCT

```
ATTTTTGAAATCAGGA--ACAG-AGTCAGCCAAGGCAGACTTTGTAGCCTTTCTACCTTTTTGAGGTTCTTAACAACCAC
ATGGGCATTGTTTCCCTTAAGAAAAACTTGGATTGTTTT------------------GCAGATTTCAGATCCTTCAGTGA
AAATTGAAGTCTCACAAGCCTGCTGTAATAAGGAGTTTTTATACCTACTATATA-GTGTACAATCTGTACAAGATCAACT
TGCCCAGATCCACACAGTGAGGCATTGACAAAG---TTTCATAGTTTCATAGTAGCTAGAGTCGGAAGGGACCTAGGTAG
ATCATCTAGTCCGACCCCCTGCCTTGG-CAGGAAAGAGCACCG---GATTTAGTGGGACCCGAATCTTGTCTTCTGTCCC
TCAAA-CCAGGATAACTAGGAAAAGCTGATTTAGAAAGAAAACACCGTTGTACATAAAAAGCAACTTGGATCTCTATTTT
TAATACAC---CCAGTGTCAGCCAG--TTTTTTCTTGATCTTTGACTTGAATCACTTTGTCAAGTAACTGAACTTGTTTT
TAATTCATCTTCCCTTCTAAGCAACACTTCATTTCCGAGGTAGAAAGACCTATAAATG-AC-------------------
---TGCGGTAAAGCGGTGTACTAATGCTTCTCCTTCCTGTTGTGGATGTTTCA
>Cpa
---------------------------------------------------------TTGTTGGCGTGCTACA----GAA
TCTAG--GAGTGCTGACTGCTTGCGGGGGGATGAGGG-------------------------------------------
-GGAGGTATTAATTTGTATGTCATCTACTGAGCTCCTTCCTAGT--AAAGTGTGACTGCTTCCCAAAAATGTCAAAAGCT
TTAATTTCATCTTGTTCCTTGAATGCTGGAAGTTAGCTTGAGACGTTTTCAGAACT------------------------
----------------TTACTTTATAGATGAGTGCGAAGTGGAAGCCTTTTTGCTGTTTGAATTGGTAGTGGAAACTGCT
ATTTTTGAAATCAGGA--ACAG-AGTCAGCCAAGGCAGACTTTGTAGCCTTTCTGCCTTTTTGAGGTTCTTAACAACCAC
ATGGGCATTGTTTCCCTTAAGAAAAACTTGGATTGTTTT------------------GCAGATTTCAGATCCTTCAGTGA
AAATTGAAGTCTCACAAGCCTGCTGTAATAAGGAGTTTTTATAGCTACTATATA-GTGTACAATCTGTACAAGATCAACT
TGCCCAGATCCACACAGTGAGGCATTGACAAAG-----------------------------------------------
----------------------------------------------GATTTAGTAGGACCCGAATCTTGTCTTCTGTCCC
TCAAA-CCAGGATAACTAGGAAAAGCTGATTTAGAAAGAAAACACCATTGTACATAAAAAGCAACTTGGATCTCTATTTT
TAATACAC---CCAGTGTCAGCCAG--TTTTTTCTTGATCTTTGACTTGAATCACTTTGTCAAGTAACTGAACTTGTTTT
TAATTCATCTTCCCTTCTAAGCAACACTTCATTTCCGAGGTAGAAAGACCTATAAATG-AC-------------------
---TGCGGTAAAGCGGTGTACTAATGCTTCTCCTTCCTGTTGTGGATGTTTCA
>Cmi
----------------------------------CTCCTTCA----------AGGGTCGGATGGCGTGCTACA----GAA
TCTAG--GAGTGCTGACTGCTTGCAGGGGGATGAGGG-------------------------------------------
-GGAGGTATTGATTTGTATGTCATCTACTGAGCTCCTTCCTAGT-AAAAGTGTGGCTGCTTGGCAAAAATGTCAAAAGCT
TTAATTTCATCTTGTTCCTTGAATGGTGGAAGTTAGCTTGAGACGTTTTCAGAACT------------------------
----------------TTACGGTATAGATGAGTGCAAAGTGGAAGCCTTTTGGCTGGTTGAAGTGATAGGGGAAACTGCT
ATTTTTGAGGTCAGGA--ACAG-ACTCAGCCAAGGCAGCATTTGTAGCCTTTCTACCTTTTTGAGGTTCTTAACAACCAC
ATGGGCATTGTTTCCCTTAAGAAAAACTTGGATTGTTTT------------------GCAGATTGCAGATCCTTCAGGGA
AAAGTGAAGTGTCACAAGCCTGCTGTAGGAGGGAGTTGTTATACCTAGTATATG-GTGTACGAGCTGTACAAGATCAACT
TGCACAGATCCACACAGTGAGGCATTGACAAAG-----------------------------------------------
----------------------------------------------GATTTAGTGGGACCCGAATCTTGTCTTCTGTCCC
TCAAA-CCAGGATAACTAGGAAAAGCTGATTTAGAAAGAAAACACCATTGTACATAAAAAGCAACTTGGATCTCTATTTT
TAATACAC---CCAGTGTCAGCCAG--TTTTTTCTTGATCTTTGACTTGAATCACTTTGTCAAGTAACTGAACTTGTTTT
TAATTCATCTTCCCTTCTAAGCAACACTTCATTTCCGAGGTAGAAAGACCTATAAATG-AC-------------------
---TGCGGTAAAGCGGTGTACTAATGCTTCTCCTTCCTGTTGTGGATGTTTCA
>Gga_scaffold15258
ACTTGTTACG---GTAACTTTTTCTAACAAAAAACTCCTTCA----------AGGGTTGTATGGCATGCTACA----GCA
TCTAGGAGAGTGCTGACTGCTTGCTGGGGGGTGAGGG-------------------------------------------
-GGAGGTATTGATTTGTATGTCATCTACTGAGCTCCTTCCTAGT--AAAGTGTGACTTCTTCCCAAAAATGTCAAAAGCA
TTAATTTCATCTTGTTCCTTGAATGCTGGAAGTTAGCTTGAGATGTTTTCAGAACT------------------------
----------------TTACTTTATAGATGAGTGCAAAGTGGAAGCCTTTTTGCTGTTTGAATTGATAGTGGAAACTGCT
ATTTTTGAAATCAGGAACACAG-AGTCAGCCAAGGCAGACTTTGTAGCCTTTCTACC-TTTTGAGGCTCTTAACAACCAT
ATGGGCATTGTTTCCCTTAAGAAAAACTTGGATTGTTTT------------------GCAGATTTCAGATCCTTCAGTGA
AAATTGAAGTCTCACAAGCCTGCTGTAATGAGG---TTTTATACCTCCTATATAGGTGTACAATCTGTACAAGATCAACT
TGCCCAGATCCTCACAGTGAGGCATCGACAAAG-----------------------------------------------
----------------------------------------------GATTTAGTAGGACCCGAGTCTTGTCTTCTGTCCC
TCAAA-CCAGGATAACTAGGAAAAGCTGATTTAGAAAGAGAACACCCATGTACACAAAAAGCAACTTAAATCTCTATTTT
TAATACAC---CCAGTGTCAGCCAG--TTTTTTCTTGATCTTTGACTTGAATCACTTTGTCAAGTAACTGAACTTGTTTT
TAATTCATCTTCCCTTCTAAGCAACACTTCATTTCCGAGCTAGAAAGACCTATAAGTG-AC-------------------
---TGCGGTAAAGCGGTGTACTAATACTTCTCCTTCCTGTTGTGGACGTTTCA
>Asi_533334148
ACTTGTTACG---GTAACTTTTTCTAACGAAAAACTCCTTCA----------AGGGTCATATGGCGTGCTACA----GCA
TCTAGGAGAGTGCCGGCTGCTTGCTGGGGGATGAGGG-------------------------------------------
-GGAGGTATTGATTTGTATGTCATCTACTGAGCTCCTTCCTAGT--AAAGTGTGACTGCTTCCTAAAAATGTCAAAAGCT
TTAATTTCATCTTGTTCCTTGAATGCTGGAAGTTAGCTTGAGACATTTTCAGGACT------------------------
----------------TTACTTTATAGATGAGTGCAAAGTGGAAGCCTTTTTGTTGTTTGAATTGATAGTGGAAACTGCT
ACTTTTGAAATCAGGAACACAG-AGTCAGCCAAGGCAGACTTTATAGCCTTTCTACCC-TTTGAGGCTCTTAAAAACCAT
ATGGGCATTGTTTTCCTTAAGAAGAACTTGGATTGTTTTGCCTTTGCTTTTTATAAAGCAGATTTCAGATCCTTCAGTGA
AAATTGATGTCTCACAAGCCTGCTGTAAAATGGAGCTTTTACACCTACTATATAGGTGTACAATCTGTAGAAGATCAGCT
TGCCCAGATCCACACAGTGAGGCATTGACAAAG-----------------------------------------------
----------------------------------------------GATTTAG---GACCCGAGTCTTGTCTTCTGTCCC
TCAAG-CCAGGATAACTAGGAAAAGCTGATTTAGAAAGAGAACACCCTTGCACATAAAAAGCAACTTAAAGATCTATTTT
TAATGCAC---CCAGTGTCAGCCAGTTTTTTTTCTTGATCTTTGACTTGAATTGCTTTGCTAAGTAACTGAACTTGGTTG
TAATTCAGCTGCCCTTCCAAGCAACACTTCGTTTCCAAGCTAGAAAGACCTATAAATG-AC-------------------
---CGCAGTAAAGCAGTGTACTAATGCTTCTCCTTCCTGTTGTGGACGTTTCA
>Ami_scaffold-663
ACTTGTTACG---GTAACTTTTTCTAACGAAAAACTCCTTCA----------AGGGTCATATGGCGTGCTACA----GCA
TTTAGGAGAGTGCCGGCTGCTTGCTGGGGGATGAGGG-------------------------------------------
-GAAGGTATTGATTTGTATGTCATCTACTGAGCTCCTTCCTAGT--AAAGTGTGACTGCTTCCTAAAAATGTCAAAAGCT
TTAATTTCATCTTGTTCCTTGAATGCTGGAAGTTAGCTTGAGACATTTTTAGGACT------------------------
----------------TTACTTTATAGATGAGTGCAAAGTGGAAGCCTTTTTGTTGTTTGAATTGATAGTGGAAACTGCT
ACTTTTGAAATCAGGAACACAG-AGTCAGCCAAAGCAGACTTTATAGCCTTTCTACC-CTTTGAGGCTCTTAAAAACCAT
ATGGGCATTGTTTTCCTTAAGAAGAACTTGGATTGTTTTGCCTTTGCTTTTTATAAAGCAGATTTCAGATCCTTCAGTGA
AAATTGAAGTCTCACAAGCCTGCTGTAAAATGGAGCTTTTACACCTACTATATAGGTGTACAATCTGTAGAAGATCAACT
```

TGCCCAGATCCACGCAGTGAGGCATTGACAAAG---
--GATT---TAGGACCCGAGTCTTGTCTTCTGTCCC
TCAAA-CCAGGATAACTAGGAAAAGCTGATTTAGAAAGAGAACACCCTTGCACATGAAAAGCAACTTAGAGATCTATTTT
TAATGCAC---CCAGTGTCAGCCAGTTTTTTTTCTTGATCTTTGACTTGAATCGCTTTGCTAAGTAACTGAACTTGGTTG
TAATTCAGCTGCCCTTCTAAGCAACACTTCATTTCCAAGCTAGAAAGACCTATAAATG-AC-------------------
---TGCAGTAAAGCAGTGTACTAATGCTTCTCCTTCCTGTTGTGGACGTTTCA
>Gallus_GB
ACTTGTTACG---GTAAACACCTTTAAGATGAGAGCTTTCTTGTGAGACTTGCACAGCAGGGAACGTGGTCCATCTGGCA
TCTCC--CAGTGCCAGCTTCCTGGAGGAAACGCAGAGCTTGTGAAACATGCTGCTTACATTCTAAAAGAGTGTGTTCTCT
TAGCAGTCTTGCTTGGCATGTT--------CAGTCGTTGCTAGTGAAAAGCGTAACTGCCCACTGAAA------------
-------------CTCCATTAAACGATGACAGCTACATTGTCGTCGTTTCAGAGCTGTGGAATATTGTCGATGGTTTGGA
ACATCTCAGCTCCTGCATACATTGCAGAGCAGGACTGAGTGGGAGCATGGCTGCTGGAGCTGCAGCTGACG--AACTGGC
AAAGCGCCAGCCAGGAAAAGAGCAGTCACCTCATACAAAC-TCACAGCCTTTTCACCTGCTGGAAATCCT----------
---GTTGGT-----------------

---GTATTTCTTTCTGTCCT
TCTAA-CTGGTGTTATTACTACTA------TTCCAAAGACAGCATTTCAGCACAAAAGCATACAATGA----CCTATTTT
AGACATACTTTCTAATGTAAATCAG--TTTGATCTCGTTCTTTGACTTGAAGCGTTCTGTT--GTGCTTCATCTGTTCTT
GCAACTGTTGTTCTTTTTAAACA------------------GGAAAGACCTCTTAGTGAATACACACAGCTTGTTGATGA
GTATGCAGCAAA----CGTTCTAATGTTGAATCTT--TGTTCCGCATGTTTCA

CP18

>CR1-2C

---------------------------------------TTTC---ATAGATTTCATAGACATTAGGG---CTGGAAGGG
A-CCTCGGAAGATCATCGAGT-CCAGCCCCCCGCCCGAAGGGCAGGAAGTCAGCTGGGGTCATAGGATCCCAGCAAGATA
AGCATCCAATTTGCTCTTGAAGGTGTTCAATGTAGGTGCTTGAACCACCTCCGATGGCAGGCTGTTCCAGACCTTGGGGG
CTCGGACAGTAAAGAAATTCTTCCTTATGTCCAGCCTGAAA-CGGTCTTGCAGTAGTTTATGACCGTTCGACCTCGTCAT
CCCTTGGGGCGCTCTGGTGAACAAACGTTCCCCCAGATCCTGGTGGTCACCCCTG-ATAAACTTATAGGTGGCCATCAGA
TCACCCCTGAGCCTGCGCTTTTCCAGGCTAAAGAGCCCCATGGCTCTCAGCCTGTCATCGTAAGGTCTGTTTTCCTGACC
TCTGATCATGCGCGTGGCTCTTCTCTGGACTCTCTCAAGCTTCTCCACATCCTTTTTGAATTGTGGAGCCCAAAACTGGA
CGCAGTACTCCAGCTGCGGCCTCACCAAGGCCGAGTACAAGGGGAGAATGACGTCCCGGGATTTGCTTGAGAAGCATCTA
TGGATGCAAGCCAGCGTTTTGGTCGCTTTACTAGCCGCA---------------GCATCGCATTGCAGGCTCATGTTCAT
CTTGTGGTCAATGATGACCCCCAAGTCTCTTTCTTCCGTAGTGCTAGCCAGCGTAGCACTGCCGAGCCTAT---------
----

>Exons
CAAAG---

--GCAGGG
TGGCCAAGATG
>Cpo_7874_34550-36100
CAAAG---GTAGAGCCAAGGCTGCAGAGGGATGAACCTGTGAAAGCTTTCAGAGGTTGAGGGGGGGTAGAAGGCATGATT
CTTTGGGCTGAGCTGAGGGAAGAGGCACAACTCCCTCTCTGCAGCCAGTTCAAATCCAGCCCAGGATTGGGAGCTACCAC
ATGATTGCTGTCTGGGTGGTGGCCTTGGATGAATGACTTTGGGCTTTGGCTCCTCTTGNNNNNNNNNNNNNNNNNNNNNN
NNNNNNNNNNNNNNNNNNNNNNNNNNNNNNNNNNNNNNNNNNNNNNNNNNNNNNNNNNNNNNNNNNNNNNNNNNNNNNNN
NNNNNNNNNNNNNNNNNNNNNNNNNNNNNNNNNNNNNNNNNNNNNNNNNNNNNNNNNNNNNNNNNNNNNNNNNNNNNNNN
NNNNNNNNNNNNNNNNNNNNNNNNNNNNNNNNNNNNNNNNNNNNNNNNNNNNNNNNNNNNNNNNNNNNNNNNNNNNNNNN

NNNNNNNNNNNNNNNNNNNNNNNNNNNNNNNNNNNNNNNNNNNNNNNNNNNNNNNNNNNNNNNNNNNNNNNNNNNNNNNN
NNNNNNNNNNNNNNNNNNNNNNNNNNNNNNNNNNNNNNNNNNNNNNNNNNNNNNNNNNNNNNNNNNNNNNNNNNNNNNNN
NNNNNNNNNNNNNNNNNNNNNNNNNNNNNNNNNNNNNNNNNNNNNNNNNNNNNNNNNNNNNNNNNNNNCGGCCTCACCAA
GGCCGAGTACAAGGGGAGAATGACGTC---CCGGGATTTGCTCGAGAAGCATCTATGGATGCAA-GCCAGCGTTTTGGTC
GCTTTATAGCCAGC---------------------G--

--TTTT---GGACTGAGGAAAGCTGATGGGGCATTCCAGG
TTGGAGAGTTTGCTCTCTTACATTTGACATTAG------------------------------------AGGGGTTCTGG
CCTAGAAGA-GGGGGACTCTGCCCCATGCAAGGGAAAGATAG--TGGGAGGAGGTAGCTTGGACCTTCATGGGTATGATG
GAGCTGGCTGTCTTGTGACTAAACACCTTACAGCTGGC-CCTAAGTGATGTATTTCCCTTTTC-CATGCAG---GCAGGG
TGGCCAAGATG
>Cno
---CTTTCAGAGGTTGAGGGGGGGTAGAAGGCATGATT
CTTTGGGCTGAGCTGAGGGAAGAGGCACAACTCCCTCTCTGCAACCAGTTCAAATCCAGCCCAGGATTGGGAGCTACCAC
ATGATTGCTGTCTGGGTGGTGGCCTTGGATGAATGACTTTGGGCTTTGGCTCCTCTT-----------------------
----

--------GGAGATGAGCTTCTGCAAC---TGCATGTCTGGCTGGCCTCTCTGCAGGGGCACAG-AGGGCTGTAGAGGCT
GAGCTTTTCCCTGCATTCCTGGAGCATGTCCTTCAG---TTTC---ATAGATTTCATAGACATCAGGG---CTGGAAGGG
A-CCTCGAGAGATCATCAAGT-CCAGCCCCCTGCCCAAAGGGCAGGAAGTCAGCTGGGGTCATAGGATCCCAGCAAGATA
AGCATCCAGTTTACTCCTGAAGGTGTTCAATGTAGGTGCTTGAACCACCTCCGCTGGCAGACTGTTCCAGACCTTGGGGG
CTCGGACAGTAAAGAAATTCTTCCTTATGTCCAGCCTGAAG-CGGTCTTGCAGGAGTTTGTGACCGTTCGACCTAGTCAT
CCCTTGGGGCGCTCTGGTGAACAAACATTCCCCCAGATCCTGGTGGTCACCCCTG-AGAAACTTATAGGTGGCCATCAGA
TCACTCCTGAGCCTGCGCTTTTCCAGGCTAAAGAGTCCCATGTCTCTCAGCCTATCATCGTAA-----------GATCTG
TTTTCCTGACCTCTGATCATGCATGTGAATCTCTCAAGCTTCTCCACATCCTTTTTGAATTGTGGAGCCCAAAATTGGAC
GCA-GTACTCCAGCTGCGGTCTCACCAAGGCTGAGTACAAGGGGAGAATGACGTCCCGGGATTTGCTCGAGAAGCATCTA
TGGATGCAAGCCAGCGTTTTGGTCGCTTTATAGCCAGCG---TTTT---GGACTGAGGAAAGCTGATGGGGCATTCCAGG
TTGGAGAGTTTGCTCTCTTACATGTGACATTA------------------------------------GAGGGGTTCTGG
CCTAGAAGA-GGGGGACTCTGCCCCATGCAAGGGAAAGATAGTG--GGAGGAGGTAGCTTGGACCTTCATGGGTATGATG
GAGCTGGCTGTCTTGTGACTAAACACCTTACAGCTGGC-CGTAAGTGATG------------------------------
----
>Ote
---CTTTCAGAGGTTGAGGGGGGGTAGAAGGCATGATT
CTTTGGGCTGAGCTGAGGGAAGAGGCACAACTCCCTCTCTGCAGCCAGTTCAAATCCAGCCCAGGATTGGGAGCTACCAC
ATGATTGCTGTCTGGGTGGTGGCCTTGGATGAATGACTTTGGGCTTTGGCTCCTCTT-----------------------
----

--------GAAGATGAGCTTCTGCAAC---TGCATGTCTGGCTGGCCTCTCTGCAGGGGCACAG-AGGGCTGTAGAGGCT
GAGCTTTTCTCTGCATTCCTGGAGCATGTCCTTTAG---TTTC---ATAGATTTCATAGACATCAGGG---CTGGAAGGG
A-CCTCGAGAGATCATCGAGT-CCAGTCCCCTGCCCAAAGGGCAGGAAGTCAGCTGGGGTCATAGGATCCCAGCAAGATA
AGCATCCAGTTTACTCCTGAAGGTGTTCAATGTAGGTGCTTGAACCACCTCCGCTGGCAGACTGTTCCAGACCTTGGGGG
CTCGGACAGTAAAGAAATTCTTCCTTATGTCCAGCCTGAAG-CAGTCTTGCAGGAATTTGTGACCGTTTGACCTAGTCAT
CCCTTGGGGCGCTCTGGTGAACAAACGTTCCCCCAGATCCTGGTGGTCACCCCTG-ATAAACTTACAGGTGGCCATCAGA
TCACCCCTGAGCCTGCGCTTTTCCAGGCTAAAGAGTCCCATGTCTCTCAGCCTATCATCGTAA-----------GATCTA
TTTTCCTGACCTCTGATCATGCATGTGAATCTCTCNTGGACTCTCTCATCCTTTTTGAATTGGGTAGCCCAAAACTGGAG
CCA-GTACTCCAGCTGCGGCCTCACCAAGGCCGAGTACAAGGGGAGAATGACGTCCCGGGATTTGCTCGAGAAGCATCTA
TGGATGCAAGCCAGCATTTTGGTCGCTTTATAGCCAGCA---TTTT---GGACTGAGGAAAGCTGATGGGGCATTCCAGG
TTGGAGAGTTTGCTCTCTTACATGTGACATTA------------------------------------GAGGGGTTCTGG
CTTAGAAGA-GGGGGACTCTGCCCCATGCAAGGGAAAGATAGTG--GGAGGAGGTAGCTTGGACCTTCATGGGTATGATG
GAGCTGGCTGTCTTGTGACTAAACACCTTACAGCTGGC-CCCAAGTGATG------------------------------
----
>Mca
---GCTTTCAGGAGGTTGAGGGGGGGTAGAAGGCATGATT
CTTTGGGCTGAGCTGAGGGAAGAGGCACAACTCCCTCTTTGCAGCCAGTTCAAATCCAGCCCAGGATTGGGAGCTACCAC
ATGATTGCTGTCTGGGTGGTGGCCTTGGATGAATGACTTTGGGCTTTGGCTCCTCTT-----------------------
----

--------GGAGATGAGCTTCTGCAAC---TGCATGTCTGGCTGGCCTCTCTGCAGGGGCACAG-AGGGCTGTAGAGGCT
GAGCTTTTCCTTGCATTCCTGGAGCATGTCCTTCAG---TTTC---ATAGATTTCATAGACATCAGGG---CTGGAAGGG
A-CCTCGAGAGATCATCGAGT-CCAGTCCCCTGCCCAAAGGGCAGGAAGTCAGCTGGGGTCATAGGATCCCAGCAAGATA

```
AGCATCCAGTTTACTCCTGAAGGTGTTCAATGTAGGTGCTTGAACCACCTCCGCTGGCAGACTGTTCCAGACCTTGGGGG
CTCGGACAGTAAAGAAATTCTTCCTTATGTCCAGCCTGAAG-CAGTCTTGCAGGAGTTTGTGACCGTTCGACCTAGTCAT
CCCTTGGGGCGCTCTGGTGAACAAACGTTCCCCCAGATCCTGGTGGTCACCCCGATAAACTTTTATAGGTGGCCATCAGA
TCACCCCTGAGCCTGCGCTTTTCCAGGCTAAAGAGTCTCATGTCTCTCAGCCTATCATCATAA-----------GATCTG
TTTTCCTGACCTCTGATCATGCATGTGAATCTCTCAAGCTTCTCCACATCCTTTTTGAATTGTGGAGCCCAAAACTGGAC
GCA-GTACTCCAGCTGCGGCCTCACCAAGGCCGAGTACAAGGGGAGAATGACGTCCCGGGATTTGCTCGAGAAGCATCTA
TGGATGCAAGCCAGCGTTTTGGTCGCTTTATAGCCAGCG---TTTT---GGACTGAGGAAAGCTGATGGGGCATTCCAGG
TTGGAGAGTTTGCTCTCTTACATGTGACATTA------------------------------------GAGGGGTTCTGG
CCTAGAAGA-GGGGGACTCTGCCCCATGCAAGGGAAAGATAGTG--GGAGGAGGTAGCTTGGACCTTCATGGGTATGATG
GAGCTGGCTGTCTTGGGGCCA-----------------------------------------------------------
-----------
>Tsc
CAAAG---GTAGAGCCAAGGCTGCAGAGGGATGAACCTGTGAAAGCTTCCAGAGGTC--CGGGGGGTAGAAGGCATGATT
CTTTGGGCTGAGCTGAGGGAAGAGGCACAACTCCCTCTCTGCAGCCAGTTCAAATCCAGCCTGGGATTGGGAGCTACCAC
ACGATTGCTGTCTGGGTGGTGGCCTTGGATGAATGACTTTGGGCTTTGGCTCCTCTT-----------------------
--------------------------------------------------------------------------------
--------------------------------------------------------------------------------
--------------------------------------------------------------------------------
--------------------------------------------------------------------------------
--------------------------------------------------------------------------------
--------------------------------------------------------------------------------
--------GGAGATGAGTTTCTGCAAC---TGGATGTCTAGCTGGCCTCTCTGCAGGGGCACGG-AGGGCTGTAGAGGCT
GAGATTTTCCCTGCATTCCTGGAGCATGTCCTTCAG--------------------------------------------
--------------------------------------------------------------------------------
--------------------------------------------------------------------------------
--------------------------------------------------------------------------------
--------------------------------------------------------------------------------
--------------------------------------------------------------------------------
--------------------------------------------------------------------------------
--------------------------------------------------------------------------------
------------------------------------------TTTG---GGACTGAAGAAAGCTGATGGGGCATTCCAGG
TTGGAGAGTTTGCTCTCTTACATGTGACATTA------------------------------------GAGGGGTTCTGG
CCTAGGAGAGGGGGGACTCTGCCCCATGCAAGAGAAAGATGGTG--GGAGGAGGTACCTTGGACCTTCATGGGTATGATG
GGGCTGGTTGTCTTGTGACTAAACACCTTACAGCTGGACCCCAAGTGATGTATTTCCCTTTTC-CACGCAG---GCAGGG
TGGCCAAGATG
>Gga_scaffold27566
CAAAG---GTAGAGCCAAGGCTGCAGAGGGGTGAACCTGTGAAAGCTTCCAGAGGTC--GGGGGGGTAGAAGGCATGATT
CTTTGGGCTGAGCTGAGGGAAGAGGCACAACTCTCTCTCTGCAGCCAGTTCAAATCCAGCCCGGGACTGGGAGCTACCAC
ATGATTGCTGTCTGGGTGGTGGCCTTGGATGAATGACTTTGGGCTTTGGCTCCTCTTGG---------------------
--------------------------------------------------------------------------------
--------------------------------------------------------------------------------
--------------------------------------------------------------------------------
--------------------------------------------------------------------------------
--------------------------------------------------------------------------------
--------------------------------------------------------------------------------
----------AGATGAGTTTCTGCAAC---TGGATGTCTAGCTGGCCTCTCTGCATGGGCACAG-AGGGCTGTAGAGGCT
GAGCTTTTCCCTGCATTCCTGGAGCATGTCCTTCAG--------------------------------------------
--------------------------------------------------------------------------------
--------------------------------------------------------------------------------
--------------------------------------------------------------------------------
--------------------------------------------------------------------------------
--------------------------------------------------------------------------------
--------------------------------------------------------------------------------
--------------------------------------------------------------------------------
------------------------------------------TTTG---GGACTGAAGAAAGCTGATGGGGCATTCCAGG
TTGGAGAGTTTGCTCTCTTACATGTGACATTAG------------------------------------AGGGGTTCTGG
CCTAGGAGA-GGGGGACTCTGCCCTATGCAAGAGAAAGATGG--TGGGAGGAGGTACCTTGGACCTTCATGGGTATGATG
GGGCTGGTTGTCTTGTGACTAAACACCTTACAGCTGGA-CCCAAGTGATGTATTTCCCTTTTC-CACGCAG---GCAGGG
TGGCCAAGATG
>Cya
CAAAG---GTAGAGCTGAGGCTGCAGAGGGACGAAGCTGTGAAAGCTTCCAGGAGTC---GGGGGGTGGAAGACATGATT
CTTTGGACTGAGCTGAGGGAAGAGGCACAACTCCTTCTCTGCAGCCAGTTCAAATCCAGCCTGGGATTGGGAGCTACCAC
ATGATTGCTGTCTGGGTGGTGGCCTTGGATGAATGACTTTGGGCTTTGGCTCCTT-------------------------
--------------------------------------------------------------------------------
--------------------------------------------------------------------------------
--------------------------------------------------------------------------------
--------------------------------------------------------------------------------
--------------------------------------------------------------------------------
--------------------------------------------------------------------------------
--------GGAGATGAGTTTCTGCAACTCATAGGTGTCTGGCTGGGCTCTCTGCAGGGGCACAG-AGAGCTGTAGAGGCT
GAGCTTTATTCTGCATTCCTGGAGCATGTCCTCCAG--------------------------------------------
--------------------------------------------------------------------------------
--------------------------------------------------------------------------------
--------------------------------------------------------------------------------
--------------------------------------------------------------------------------
--------------------------------------------------------------------------------
--------------------------------------------------------------------------------
--------------------------------------------------------------------------------
```

--TTTG---GGACTGAAGAAAGCTGATGGGGCATTGCAGG
TTGGAGAGTTTGTGCTCTAACATGTGACACTA------------------------------------AAGGGGTTCTGG
CCTAGAAGA-GGGGGACTCTGCCCCATGCAAGGAAAAGATGGTT--AGAGGATGTAGCTTGGACCTTTGTGGGTATGACG
GGGTTGGCTGTCTTGTGACTAAACACCTTACAGCTGGG-CCCAAATGATGCATTTCCCTTC-------------------
----
>Asi_533413340
CAAAG---GTAGAGCCGAGGCTGCAGAGGGACGAAGCTGTGAAAGCTTCCAGGGGTC---GGGGGGTGAAAGGCATGATT
CTCTGGAGTGAGCTGAGGGAAGAGGCGCAACTCCCTCTCTGCAGCCAGTTCAAGTCCAGCCTGGGA-TGGGAGCTACCAC
GTGATTGCTGTCTGGGTGGTGGCCTTGGATGAATGACTTTGGGCTTTGGCTCCTCTTGG---------------------
----

----------AGATGAGTTTCTGCAACTCATAAGTGTCTGGCTGGGCTCTCTGCAGGGGCACAG-AGAGCTGTAGAGGCT
GAGCTTTACTCTGCATTCCTGGAGCATGTCCTTCAG--

--TTTG---GGACTGAAGAAAGCTGATGGGGCATTGCAGG
TTGGAGAGTTTGCGCTCTAACATGTGACACTAG------------------------------------AGGGGTTCTGG
CCTAGAAGA-GGGGGACTCTGCCCCGTGCAAGGGAAAGATGG--TGGGAGGATGTAGCTTGGACCTTTGTGGGTATGACG
TGGCTGGCTGTCTTGTGACTAAACACCTTACAGCTGGG-CCCAAATGATGTATTTCCCTTTTC-CCTGCAG---GCAGGG
TGGCCAAGATG
>Ami_scaffold-6101
CAAAG---GTAGAGCCGAGGCTGCAGAGGGACAAAGCTGTGAAAGCTTCCAGGGGCC---GGGGGGTGGAAGGCATGATT
CTTTGGGGTGAGCTGAGGGAAGAGGCGCAACTCCCTCCCTGCAGCCAGTTCAAGTCCAGCCTGGGA-TGGGAGCTACCAC
GTGATTGCTGTCTGGGTGGTGGCCTTGGATGAATGACTTTGGGCTTTGGCTCCTCTTGG---------------------
----

----------AGATGAGTTTCTGCAACTCATAAGTGTCTGGCTGGGCTCTCTGCAGGGGCACAG-AGAGCTGTAGAGGCT
GAGCTTTACTCTGTATTCCTGGAGCATGTCCTTCAG--

--TTTG---GGACTGAAGAAAGCTGATGGGGCATTGCAGG
TTGGAGAGTTTGTGCTCTAACATGTGACACTAG------------------------------------AGGGGTTCTGG
CCTAGAAGA-GGGGGACTCTGCCCCATGCAAGGGAAAGATGG--TGGGAGGATGTAGCTTGGACCTTCGTGGGTATGATG
GGGCTGGCTGTCTTGTGACTAAACACCTTACAGCTGGG-CCCAAATGATGCATTTCCCTTTTC-CCTGCAG---GCAGGG
TGGCCAAGATG
>Gallus_GB
TAAAG---GTA-----------ATGAAGGGCTGAGTGACTGAGGAGCCAGACAGGTCTGGGAAGGCTG------------
CCTGGGGGAAGTCTGAGAAAGG----------TCTTTAGGAGTGGTTGGTTTGAATCCAATGCAATG-TAAGAGTTGCTC
AACTCTGTTGCTT--GTGGTAGCACTCACTTAACAGCTTAGCAGTCTGT-------------------------------
----

----------AGATCACTT-----------CCGTAGTGTTGTGGAGCAGTATTTTACAGATGGA-ATAGCTGTGGAGGCA
GCCTTT-----------------GTGACTTCTATAT--

--GCTA---GGATTGAAGAATACCAGGG--------TGAG
GTGAAACATCCTTCCTTTTGTTGGTGAATATAGTGAGAACTACTTATCACAGCCAATCCTGTGACTGCAGGAGGTTCAGA
CCAAAGTGA-GTAGGCTGTCTCCTGAGCTTTGGAACAGATGGACTGCAGAAAGGTGTCTGGAACTTGTTGCAATAAGACA
AA-------------TACCCAAAAGCTTCCTTTTTGGA-TCTAAGCACTGTTTCCCTCTGGCCACCTACAG---GTAGAG
TGGCCAAAATG

CP19

```
>CR1-7B_Gav
--------------------------------------------------------------------------------
------------------TAG------TTTCATAGTTTCATAGTTTCTAGGGTCGGAAGGGACCTGAGCAGATCATCTAG
TCCGACCCCCTGCCATGGCAGGAAAGAGTACTGGGGTCAAACGACCCCAGCAAGGTGTTCATCTAACCTCCTCTT-AAAA
ACCCCCAGGGTAGGAGCCTGCACCACCTCCCTTGGAAGTTGGTTCCAGACCCTAGCCGCCCTGACCGTGAAATAGCGCCT
CCTGATGTCTAACCTAAACCTACCCTCTGCCAGCTTGTGACCGTTGTTCCTGGTCACTCCTGGCTGTGCTCGGGGGAACA
GTGACTCCCCCAATGCCTGCTGAT---CCCCCCTGACCAGTTTGTAACAGGCCACAAGATCCCCCCTCAGCCTTCTCTTT
TGGAGGCTGAACAGGTTCAGGTCCCGTAGCCTCTCCTCATAGGGCCTGCCCTGCAGTCCCCTGATCATGCGAGTGGCCCT
CCTCTGGACCCTCTCCATGCTGTCCACA---TCCCTCTTGAA------------------GTGCGGCGCC------CAGA
ACTGGACGCAGTACTCCAACTGCGGCCTGACCAGTGCCGCATAGAGGGGGAGGATCACCTCTCTGGACCTGCTCGAGATG
CATCTGTGGATGCATGACAAGGTACG------------------------------------------------------
--------------------------------------------------------------------------------
--------------------------------------------------------------------------------
--------------------------------------------------------------------------------
--------------------------------------------------
>Exons
--------------------------------------------------------------------------------
--------------------------------------------------------------------------------
--------------------------------------------------------------------------------
--------------------------------------------------------------------------------
--------------------------------------------------------------------------------
--------------------------------------------------------------------------------
--------------------------------------------------------------------------------
--------------------------------------------------------------------------------
--------------------------------------------------------------------------------
--------------------------------------------------------------------------------
--------------------------------------------------------------------------------
--------------------------------------------------------------------------------
--------------------------------------------------------------------------------
-------------------GTTGATGAAATCATGATGACTCTAAAACAGG
>Cpo_9705_38350-39900
CGACATAAATGAAGAGAGCAATCTTGTACCTTGCTGAGAGGTTTCTTTGGCTTAGTG--TTTTCAATTCCCATTGCCCTG
GAACTGCACCCAGAA---TGTAAT---TGTCATAGTTTCATAGTTTCTAGGGTCAGAAGGGACCTGAGTAGATCATCTAG
CCCGACCCCCTGCCGTGGCAGGAAATAATACTGGGGTCAAACAACCCCAGCAAGATGTTCATCCAACCTCCTCTT-AAAA
ACCCCCAGGGTAGGAGCCAGCACCACTTCTCTTGGAAGTTGGTTCCAGACTCTAGCCGCCCTGTC--TGAAGTAGCGCTT
CCTGATATCTAGCCTAAACATGCCCTCTGCCAGCTTGTGACCGTTATTCCTGGTCACTCCTGGCAGTGCATGGGGGAATA
GTGACTCCCCTAATGCCTGCTGAT---CCTCCCCGAGTAGTTTATAACAGGCCACTAGATCCCCCCTCAGCCTTCTCTTG
TGGAGGCTGAACATGTTCAGGTCCCGTAACCTCTTCTCGTAGGGTCTACCCTGCTGCCCCCTGATCATGTAAGTGGCCCT
CCTCTGGACCCTCTCGATGCTGTCCACAAGATTGCAGTGTAATGTAA---TGTAA---TGTCCTTAAGATTGTAATGTAA
AAAAAAA---GTTAATATTTGTTTAAGGCAGAAATAAGAATATTAAAACCAATGTAATCATTTCCCTTGATATTTTGGGT
GACCATGGAAAACTTCATGCTTAACAAAG---TATTGAATCAGATTATATGCAATAGAATTCCCATCAAAATGAATGGGG
AGTCCTTAAAACTGACACCAGTGTTGATTTTTAAAGTCAAAGCTCCCTGCTTCACTAGTAATCGGGTCATTGTCTCATTG
A---CATCCTCTGCTCTGGAGCAT-AAATA------------TTAACCTAAGATGCCATTTCAAAGAAAAAGCACACCAA
CATAA-CTTTAAC-----ATAAGTTTTGTTCAT------------------------------TCACCTTGGTTTCT-GT
TGTTA-TTCTCTTTAG---GTTGATGAAATCATGATGACTCTAAAACAGG
>Cno
-------AATGAAGAAGCAATCCTTGTACCTTGCTGAGAGGTTTCTTTGGCTTAGTG--TTTTCAATTCCCATTGCCCTG
GAACTGCACCCAGAA---TGTAAT---TGTCATAGTTTCATAGTTTCTAGGGTCGGAAGGGACCTGAGTAGATCATCTAG
TCCGACCCCCCGCCGTGGCAGGAAATAATACTGGGGTCAAACAACCCCAGCAAGATGTTCATCCAACCTCCTCTT-AAAA
ACCCCCAGGGTAGGAGCCAGCACCACTTCTCTTGGAAGTTGGTTCCAGACTCTAGCCGCCCTGTC--TGAAGTAGCGCTT
CCTGATATCTAGCCTAAACATGCCCTCTGCCAGCTTGTGACCGTTATTCCTGGTCACTCCTGGCAGTGCATGGGGGAATA
GTGACTCCCCTAATGCCTGCTGAT---CCTCCCCGACTAGTTTATAACAGGCCACTAGATCCCCCCTCAGCCTTCTCTTG
TGGAGGCTGAACATGTTCAGGTCCCGTAACCTCTCCTCGTAGGGTCTACCCTGCTGCCCCCTGATCATGTAAGTGGCCCT
CCTCTGGACCCTCTCGATGCTGTCCACAAGATTGCAGTGTAATGTAA---TGTAA---TGTCCTTAAGATTGTAATGT-A
AAAAAAA---GTTAATATTTGTTTAAGGCAGAAATAAGAATATTAAAACCAATGTAATCATTTCCCTTGATATTTTGGGT
GACCATGGAAAACTTCATGCTTAACAAAG---TATTGAATCAGATTATATGCAATAGAATTCCCATCAAAATGAATGGGG
AGTCCTTAAAACTGACACCAGTGTTGATTTTTAAAGTCAAAGCTCCCTGCTTCACTAGTAATCAGGTCATTGTCTCATTG
A---CATCCTCTGCTCTGGAGCAT-AAATA------------TTAACCTAAGATGCCATTTCAAAGAAAAAGCACACCAA
CATAA-TTTTAAC-----ATAAGTTTTGTTCAT------------------------------TCACCTTGGTTTCT-GT
TGTTA-TTCTCTTTAG---GTTGATGAAATCATGATGACTCTAAAACAGG
>Ote
CGACATAAATGAAGAGAGCAATCTNGTACCTTGCTGAGAGGTTTCTTTGGCTTAGTGTTTTTTCAATTCCCATTGCCCTG
GAACTGCACCCAGAA---TGTAAT---TGTCATAGTTTCATAGTTTCTAGGGTCGGAAGGGACCTGAGTAGATCATCTAG
TCCGACCCCCCACCATGGCAGGAAATAATACTGGGGTCAAACGACCCCAGCAAGATGTTCATCCAACCTCCTCTT-AAAA
ACCCCCAGGGTAGGAGCCAGCACCACTTCTCTTGGAAGTTGGTTCCAGACCCTAGCCGCCCTGTCCGTGAAGTAGCGCTT
CCTGATATCTAGCCTAAACATGCCCTCTGCCAGCTTGTGACCGTTATTCCTGGTCACTCCTGGCAGTGCACGGGGGAATA
GTGACTCCCCTAATGCCTGCTGAT---CCCCCTCGACTAGTTTATAACAGGCCACTAGATCCCCCCTCAGCCTTCTCTTG
TGGAGGCTGAACATGTTCAGGTCCCGTAGCCTCTCCTCATAGGGTCTACCCTGCTGCCCCCTGATCATGTAAGTGGCCCT
CCTCTGGACCCTCTCGATGCTGTCCACAAGATTGCAG-----TGTAA---TGTAA---TGTCCTTAAGATTGTAATGT-A
AAAAAAA---GTTAATGTTTGTTTAAGGCAGAAATAAGAATATTAAAACCAATGTAATCATTTCCCTTGATATTTTGGGT
GACCATGGAACACTTCATGCTTAAAAAAG---TATTGAATCAGATTATATGCAATAGAATTCCCATCAAAATGAATGGGG
AGTCCTTAAAACTGACAGCAGTGTTGATTTTTAAAGTCAAAGCTCCCTGCTTCACTAGTAATCAGGTCATTGTCTCATTG
A---TATGCTCTGCTCTGGAGCAT-AAATA------------TTAACATAAGATGCCATTTCAAAGAAAAAGCACACCAA
CATAA-CTTGAAC-----ATAAGTTTTATTCAT------------------------------TCACCTTGGTTTCT-GT
TGTTA-TTCTCTTTAG---GTTGATGAAATCATGATGACTCTAAAACAGG
>Mca
-------------------------GTACCTTGCTGAGAGGTTTCTTTGGCTTAGTG--TTTTCAATTCCCATTGCCCTG
```

```
GAACTGCACCCAGAA---TGTAAT-----------TGTCATAGTTTCTAGGGTCGGAAGGGACCTGAGTAGATCATCTAG
TCCGACCCCCCACCATGGCAGGAGATAATACTGGGGTCAAACGACCCCAGCAAGATGTTCATCCAACCTCCTCTT-AAAA
ACCCCCAGGGTAGGAGCCAGCACCACTTCTCTTGGAAGTTGGTTCCAGACCCTAGCCGCCCTGTCCGTGAAGTAGCGCTT
CCTGATATCTAGCCTAAACATGCCCTCTGCCAGCTTGTGACCGTTATTCCTGGTCACTCCTGGCCGTGCACAGGGGAATA
GTGACTCCCCTAATGCCTGCTGATCCCCCCCCCGACTAGTTTATAACAGGCCACTAGATCCCCCCTCAGCCTTCTCTTG
TGGAGGCTGAACATGTTCAGGTCCCGTAGCCTCTCCTCATAGGGTCTACCCTGCTGCCCCCTGATCATGTAAGTGGCCCT
CCTCTGGACCCTCTCGATGCTGTCCACAAGATTGCAG-----TGTAA---TGTAA---TGTCCTTAAGATTGTAATGT-A
AAAAAAA---GTTAATGTTTGTTTAAAGCAGAAATAAGAATATTAAAACCAATGTAATCATTTCCCTTGATATTTTGGGT
GACCATGGAACACTTCATGCTTAACAAAG---TATTGAATCAGATTATATGCAATAGAATTCCCATCAAAATGAATGGGG
AGTCCTTAAAACTGACAGCAGTGTTGATTTTTAAAGTCAAGCTCCCCTGCTTCACTAGTAATCAGGTCATTGTCTCATTG
A---TATGCTCTGCTCTGGAGCAT-AAATA------------TTAACCTAAGATGCCATTTCAAAGAAAAAGCACACCAA
CATAA-CTTTAAC-----ATAAGTTTTGTTCAT------------------------------TCACCTTGGTTTCT-GT
TGTTA-TTCTCTTTAG---GTTGAT-------------------------
>Tsc
CGACATAAATGAAGAGAGCAATCTTGTACCTTGCTGAGAGGTTTCTTTGGCTTAGTG--TTTTCAGTTCCCATTGCCCTG
GAACTGTACCCAAAA---TGTAAT--------------------------------------------------------
--------------------------------------------------------------------------------
--------------------------------------------------------------------------------
--------------------------------------------------------------------------------
--------------------------------------------------------------------------------
--------------------------------------------------------------------------------
----------------------------------------------------------TGTCCTTAAGATTGCAATGT-A
AAAAAAA---GTTAATGTTTGTTTAAGGCAGAAATAAGAATATTAAAACCAATGTAATCATTTTCCTTGATATTTCGGGT
GACCATGGAACACTTCATGCTTAACAAAG---TATTGAATCAGATTATATGCAATAGAATTCCCATCAAAATGAATGGGG
AGTCCTTAAAACTGACAGCAGTGTTGATTTTTAAAGTCAAAGCTCCCTGCTCCACTAGTAGTCAGGTCATTGTCACATTG
A---TACGCTCTGCTCTGGAGCAT-AAATA------------TTAACCTAAGATACCATTTCAAAGAAAAAGCACACCAA
CATAA-CTTTAAC-----ATAAGTTTTGTTCAT------------------------------TCACCTTGGTTTCT-GT
TGTTA-TTCTCTTTAG---GTTGATGAAATCATGATGACTCTAAAACAGG
>Gga_scaffold15335
CGGCATAAATGAAGAGAGCAATCTTGTACCTTGCTGAGAGGTTTCTTTGGCTTAGTG--TTTTCAATTCCCATTGCCCTG
GAACTGTACCCAAAA---TGTAAT--------------------------------------------------------
--------------------------------------------------------------------------------
--------------------------------------------------------------------------------
--------------------------------------------------------------------------------
--------------------------------------------------------------------------------
--------------------------------------------------------------------------------
----------------------------------------------------------TGTCCTTAAGATTGCAATGT-A
AAAAAAA---GTTAATGTTTGTTTAAGGCAGAAATAAGAATATTAAAACCAATGTAATCATTTTCCTTGATATTTCGGGT
GACCATGGAACACTTCATGCTTAACAAAG---TATTGAATCAGATTATATGCAATAGAATTCCCATCAGAATGAATGGGG
AGTCCTTAAAACTGACAGTAGTGTTGATTTTTAAAGTCAAAGCTCCCTGCTCCACTAGTAGTCAGGTCATTGTCTCATTG
A---TACGCTCTGCTCTGGAGCAT-AAATA------------TTAACCTAAGATACCATTTCAAAGAAAAAGCACACCAA
CATAA-GTTTAAC-----ATAAGTTTTGTTCAT------------------------------TCACCTTGGTTTCT-GT
TGTTA-TTCTCTTTAG---GTTGATGAAATCATGATGACTCTAAAACAGG
>Cya
CAGTATAAATTAAGAGAACAATCTTGTACCTTGCTGAGAGGTTTCTTTGACTTAGTG--CCTTCAGTTTCCATTGCCCTG
GAACTGTACCCAAAA---TGTAAT--------------------------------------------------------
--------------------------------------------------------------------------------
--------------------------------------------------------------------------------
--------------------------------------------------------------------------------
--------------------------------------------------------------------------------
--------------------------------------------------------------------------------
----------------------------------------------------------TGTCCTTAAGACTGCAATGT-A
AAAAAAAAATGTTAATGTTTGTTTGAAGCAGAAATAAGAATATTAAAACCAATGTAATCATTTTCCTTGGTATTTTGGGT
GACCATGGAACACTTCATGCTTAACAAAGAATTATTGAGTCAGGTTATATGCAATAGAATTCCCATCAAAATGAATGGGG
AGTCCTTAAAACTGACAGCAGTGTTGATTTTTAAAGTCAAAACTCCCAGCTCCACTATTAGTCATGTCATTGTCTTATTG
A---TATGCTCTGCTCTGGAGCAT-AAATA------------TTAACCTAAGATAACATTTCAAAGAAAAAGCACACCAA
CATAA-CTTTAACATAGTATAAGTTTTGTTCAT------------------------------TCACCTTGGTTTCT-GT
TGTTA-TTCTCTTTAG---GTTGATGAAATTATGATGACTCTAAAACAGG
>Asi_533317515
CAATATAAATGAAGAGAACAATCTTGTACCTTGCTGAGAGGTTTCTTTGACTTAGTG--CCTTCAGTTCCCATTGCCCTG
GAACTGTACCCAAAA---TGTAAT--------------------------------------------------------
--------------------------------------------------------------------------------
--------------------------------------------------------------------------------
--------------------------------------------------------------------------------
--------------------------------------------------------------------------------
--------------------------------------------------------------------------------
----------------------------------------------------------TGTCCTTAAGATTGCAATGT-A
AAAAAAA-ATGTTAATGTTTGTTTGAGGCAGAAATAAGAATATTAAAACCAATGTAATCATTTTCCTTGATATTTTGGGT
GACCATGGAACACTTCATTCTTAACAAAG---TATTGAGTCAGATTATATGCAATAGAATTCCCATCAAAATGAATGGGG
AGTCCTTAAAACTGACAGCAGTGTTGATTTTTAAAGTCAAAACTCCCTGTTCCACTATTAGTCATGTCATTGTCTCATTG
A---TATGCTCTGCTCTGGAGCAT-AAATA------------TTAACCTAAGATACCATTTCAAAGAAAAAGCACACCAA
CATAA-CTTTAACATAGTATAAGTTTTGTTCAT------------------------------TCACCTTGGTTTCT-GT
TGTTA-TTCTCTTTAG---GTTGATGAAATCATGATGACTCTAAAACAGG
>Ami_scaffold-13922
CAATATAAATGAAGAGAACAATCTTGTACCTTGCTGAGAGGTTTCTTTGACTTAGTG--CCTTCAGTTCCCATTGCCCTG
GAACTGTACCCAAAA---TATAAT--------------------------------------------------------
--------------------------------------------------------------------------------
--------------------------------------------------------------------------------
```


--TGTCCTTAAGATTGCAATGT-A
AAAAAAA-ATGTTAATGTTTGTTTGAGGCAGAAATAAGAATATTAAAACCAATGTAATCATTTTCCTTGATATTTTGGGT
GACCATGGAACACTTCATTCTTAACAAAG---TATTGAGTCAGATTATATGCAATAGAATTCCCATCAAAATGAATGGGG
AGTCCTTAAAACTGACAGCAGTGTTGATTTTTAAAGTCAAAACTCCCTGTTCCACTATTAGTCATGTCATTGTCTCATTG
A---TATGCTCTGCTCTGGAGCAT-AAATA------------TTAACCTAAGATACCATTTCAAAGAAAAAGCACACCAA
CATAA-CTTTAACATAGTATAAGTTTTGTTCAT------------------------------TCACCTTGGTTTCT-GT
TGTTA-TTCTCTTTAG---GTTGATGAAATCATGATGACTCTAAAACAGG
>Gallus_GB
TGAAAG---------AAGAGACCTATTGTCATTTTGTGTTCTGTATTTTGCTGGATATCTTCACAACTGTGGGGCTGTGC
TACGGGTCAGTGCAG---TGCCTC--

--ACTGCTGTAG------TAGCCTTGAGTTTTTGGTGT--
---------TTTTTTTTTTTCTTTTAATCGTATACAAGAAAACATTCCCAGATAGAAGCAGTGTCTATGAC---------
---TATACCACAAAA-----ATAAAAATGATT----
AGCTGGAAAAAGTGACTTCAATAATTTTTTT--------------------------------------TTGTCCTTATG
AGTTCTTGTGCTGTTTCTGCACAC-AAACAACTCCTCTTTGCTTAACAT-ATGCACCTTCCCAA---------ACAGCAG
TGTCATTCTGAAT-----GAAGGTTGTGTTTGTGTTAGGGAAAGGAGAAGGTCTGTGGAGAAGTGAAATTGTTCTTTTGC
TCTCTGTTCTTTTTAG---GTTGATGAAATCATGATGACATTGAAACAGG

GC01

>CR1-2

--GAGGAG
AGGCTACGGGACCTGAACCTGTTCAGCCTCCACAAGAGAAGGCTGAGGGGGGATCTAGTGGCCTGTTACAAACTAGTCAG
GGGGGACCAGCAGGCACTGGGGGAGTCCCTGTTCC---------CCCGAGCACTGCCAGGAGTGACCAGGAAATAA---C
GGTCACAAGCTGGCAGAGGGTAGATTCAGATTAGACATCAGGAGGCGCTACTTCACTGTCAGGGCGGCTAGGATCTGGAA
CCAACTTCCAAGCGAAGTGGTGCTGGCTCCTACCCTGGGGGTCTTTAAGAGGAGGCTTGACGAWCACCTTGCTGGGGTCG
TTTGACCCCAGTACTCTTTCCTGCCATGGCAGGGGGTCGGACTTGATGATCTGCTCAGGTCCCTTCCGACCCTACTAACT
ATGAAAC--TATGAAACTATG---

>Exons_rc
GCTGCTCCTGGCAGTAG---

>Cpo_1895_155750-157300
GCTGCTCCTGGCAGTAG---CTGTGCAACAGAGGCACCCACGGGATGAAGTGT------------GAGGGGCGAGGGTGC
GACTC--CGAGATGGGCACA
GCCCTCGTGGAGACTGCGCGGCAGC----------------GCGAGTCT--GGGGCAGGGAGCCAGGGTCCTGCCGTGTC
AACCTTGGCTCTGACATGGGG-----CTCAGCTCCCTTATGGGGCTACTGCCACCGCAGCTTTGCTGCCAGAGTCCCCTA
GTTTCAAGCAGCAGGCGCCAGAAACCTTCTTTGTCAGCCCAGAAGGGCTTTTACCAGTGCTTCAGCAAAAGGATGTGCTA
TTTCCACC--------------CCTGCCAATGCTGGCAAAATAGCATAAGGGA---------------------------
------------------GATAA---------ATGC------AACCCGAGCACTGCCAGGAGTGACCAGGAA-TAG---C
CGGCACAAGCTGGCAGAGGGTAGATTTAGGCTAGACATCAGGAGGCGCGACTTCACGGTCAGGGCGGCTAGGGTCTGGAC
CCAATTTCCAAGCAAAGTGGTGCTGGCTCCTACCCTGGGGGGCTTTAAGAGGAGGCTGGATGAACACCTTACTGGGGTCC
TTTGACCC-AGTACTCTTTCCTGCCATGGCAGGGGGCTGGACTAGATGATCTGCTCAGGTCCCTCC-GACCCGACCAACT
ACGAAACTATATGAAACCTAG---CCCTTGAGAGGCAGGGCAAGGAAGACGGCA-GCGCACAGATGTCATGCTCCGGCCA
CAGTAGCCAGGGGCCAGGGCACTAGATCAGCCATGACCGACCAGAGCCAGAGCTGGCACGG-------------------
-CTCAGACCCCGCTGTGCCCAGGGCTGGGAGGAGGGAGACGGCGACTGAACCCAGCGGGAAACT
>Ote

---GGGGCGGGAAGCCAAGGTCCTTCCATGTT
AACCTTGGGTTTGACCAGGGG-----GTTAGCTCCCTTTAGGGGGTAATTCCAACGGAGCTTTGNNGCCAGAATTCCCTA
ATTTCAAACAGCCGGGCCCAGAAACCTTCTTTGTCAGCCCCGGAGGGGTTTTTCCCAGGCTTTAACCAAAAGAATTGCCA
TTTTCCCC--------------CCTGCCGAAGCTGGGAAAATAACATAAAGGA---------------------------
------------------GATAA---------ATGC------AACCCGAGCACCGCCAGGAGTGACCAGGAA-TAG---C
CGGCACAAGCTGGCAGAGGGTAGATTTAGGCTAGACATCAGGAGATGCTACTTCACGGTCAGGGCGGCTAGGATCTGGAC

```
CCAATTTCCAAGCAAAGTGGTGCTGGCTCCTACCCTGGGGGGCTTTAAGAGGAGGCTGGATGAACACCTTGCCGGGGTCC
TTTGACCC-AGTACTCTTTCCTGCCATGGCAGGGGGCCGGACTAGATGATCTGCTCAGGTCCCTTCGGACCCGACCAATT
--------------------------------------------------------------------------------
--------------------------------------------------------------------------------
----------------------------------------------------------------
>Mca
GCTGCTCCTGGCAGTAG---CTGTGCAACAGAGGCACCCGCGGGATGAAGTGT------------GAGGGGCGAGGGTGC
GACTC--------------------------------------------------------------CGAGATGGGCACA
GCCCTCGTGGAGACTGCGCGGCCGT----------------GCGAGTCT--GGGGCAGGGAGCCAGGGTCCTGCCGTGTC
AACCTTGGCTCTGACATGGGG-----CTCAGCTCCCTTACGGGGCTACTGCCACCGCAGCTTTGCTGCCGGAGTCCCCTA
GTTTCAAGCAGCAGGCGCCAGAAACCTCCTTTGTCAGCCCAGAAGGGCTTTTACCAGTGCTTCAGCAAAAGGATGTGCTA
TTTCCACC--------------CCTGCCGATGCTGGCAAAATAGCATAAGGGA---------------------------
------------------GATAA---------ATGC------AACCCGAGCACTGCCAGGAGTGACCAGGAA-TAG---C
CGGCACAAGCTGGCAGAGGGTAGATTTAGGCTAGACATCAGGAGATGCTACTTCACGGTCAGGGCGGCTAGGATCTGGAC
CCAATTTCCAAGCAAAGTGGTGCTGGCTCCTACCCTGGGGGGCTTACATCAGGGCTTGCTTCTCCACTGATTACCTCTAT
TCTAGGT--AGTCTTTGGTTAGCCTGCAAGGTACAAAAGTACCCTGCCTTGCATTCTAGTAGT-----------------
--------------AATTTAC---TTTTTATGTGGATATACAGAGATAGTTTTA-AAGCAGCTAGCATGTGTACTGCTAG
CACTGCGC---------------------------CCTCATCAGCACAGAACTCAGCATAGACTTACTGGCCATGTATTT
ATTCAGCTTCCCCAGCAACTTCTGCAGCCGGCACTGAACTCCTTGATGCAGCAGCTACACAGCC
>Tsc
GCTGCTTCTGGCAGTAG---CTGTGCAACAGAGGCACCCACAGGATGAAGTGT------------GAGGGGTGAGGGTGC
GACTC--------------------------------------------------------------CGAGATGGGCACA
GCCCCCGTGGAGACTGCGCGGCAGT----------------GCGAGTCT--GGGGCAGGGAGCCAGGGTCCTGCCGTGTC
AACCTTGGCTCTGACATGGGG-----CTCGGCTCCCTTATGGGGCTACTGCCACCGCAGCTTTGCTGCCAGAGTCCCCTA
GTTTCAAGCAGCAGGCACCAGAAACCTCCTTTGCCAGCCCAGAAGGGCTTTTACCAGTGCTTCAGCAAAAGGATGTGCTA
TTTCCACC--------------CCTGCCGATGCTGGCAAAATAGCATAAGGGA---------------------------
------------------GATAAGCCACTGCCATGC------AACCCGAGCACTGCCAGGAGTGACCAGGAA-TAA----
--------------------------------------------------------------------------------
--------------------------------------------------------------------------------
-------------------------------------TGGACTTGATGATCTGCTCAGGTCCCTCT-GACCCGACCAACT
ATGAAACTATATGAAACCTAG---CCCTTGAGAGGCAGGGCAAGGGAGACCGCA-ATGCATAGATGTCACGCTCCGGCCA
CACTAGCCAGGGGCCAGGGCACTAGATCAGCCATAACTGACCAGAGCTAGAGCTGGCATGG-------------------
-CTCAGACCCCACGGTGCCCAGGGCTGGGAGGAGGGAGACGGCGACTGAACCCGGTGGGAAACT
>Gga_scaffold21810
GCTGCTTCTGGCAGTAG---CTGTGCAACAGAGGCACCCACAGGATGAAGTGT------------GAGGGGTGAGGGTGC
GACTC--------------------------------------------------------------CGAGATGGGCACA
GCCCCCGTGGAGCCTGCGCGGCAGT----------------GCGAGTCT--GGGGCAGGGAGCCAGGGTCCTGCCGTGTC
AACCTTGGCTCTGACTTGGGG-----CTCGGCTCCCTTATGGGGCTACTGCCACCGCAGCTTTGCTGCCAGAGTCCCCTA
GTTTCAAGCAGCAGGCACCAGAAACCTCCTTTGTCAGCCCAGAAGGGCTTTTACCAGTGCTTCAGCAAAAGGATGTGCTA
TTTCCACC--------------CCTGCTGATGCTGGCAAAATAGCATAAGGGA---------------------------
------------------GATAAGCCACTGCCATGC------AACCCGAGCACTGCCAGGAGTGACCAGGAA-TAA----
--------------------------------------------------------------------------------
--------------------------------------------------------------------------------
-------------------------------------TGGACTTGATGATCTGCTCAGGTCCCTCT-GACCCGACCAACT
ATGAAACTATATGAAACCTAG---CCCTTGAGAGGCAGGGCAAGGAAGACAGCA-ATGCACAGATGTCACGCTCCGGCCA
CACTAGCCGGGGGCCAGGGCACTAGATCAGCCATAACTGACCAGAGCTAGAGCTGGCATGG-------------------
-CTCAGACCCCACGGTGCCCAGGGCTGGGAGGAGGGAGACAGCGACTGAACCTGGCGGGAAACT
>Cya
GCTGCTCCTGGCAGTGG---CTGTGCAACAGAGGCACCCACAGGATGAAGTCT------------GAGGGGTGAGGGCGC
GACTC--------------------------------------------------------------CGGGATGGGCACA
GCCCTCGTGGAGACCGCAGGGCAATCAGAGAGCGGGGCCGGGTGAGTCT--GGGGCAGGGAGCCAGGGTCCTGCCAGGTC
AAC-----------------------CTTGGCTCCCTTATGGGGCTACTGCCACCGCAGCTTTGCTGCCAGGGTCCCCTA
GTGCCAAGCAGCAGGCACCAGAAACCTCCTTTGTCATCCCAGAAGGGGGTTTACCAGTGCTTCAGCAAAAGGATGTACTG
TTTCCACCCTCACCACCCCTGACCTACCGATGCTGGCAAAATAGCATAGGGGA---------------------------
------------------GATAAGCCACGGCCATGC--------------------------------------------
--------------------------------------------------------------------------------
--------------------------------------------------------------------------------
--------------------------------------------------------------------------------
--------------AACCTAG---CCCTTGAGAGGCAGGGCAAGGATGACAGCA-ATGCATAGATGTCATGCGCTGGCCA
CACTATCCAGGGGCCCGGGACCTAGATCGGCCATCACTGACCAGAGCCAGAGCTGGCATGG-------------------
-CTCGGACCCCACTGTGCCCAGTGCCGGCAGGAGGGAGACAGCGATTGAACTTGGTGGGAAACT
>Asi_533486932
GCTGCTCCTGGCAGTAG---CTGTGCAACAGAGGCACCCACAGGATGAAGTCT------------GAGGGGTGAGGGCGT
GACTC--------------------------------------------------------------CGAGGTGGGCACA
GCCCTCATGGAGACCGCAGGGCAATCAGAGAGCGGGGCACTGTGAGTCT--GGGGCAGGGAGCCAGGGTCCTGCCATGTC
AACCTTGGCAGGACCCTGACATGGGGCTCAGCCCTCTTATGGGGCTACTGCCACCGCAGCTTTGCTGCCAGAGGTCCCTA
GTGCCAAGCAGCAGGCACCAGAAACCTCCTTTGTCAGCCCAGAAGGGGTTTTATCAGTGCTTCAGCAAAAGGATGTACTG
TTTCCACCCCTGCCACACCTGACCTACCGATGCTGGCAAAATAGCATAGGGGA---------------------------
------------------GATAAGCCACTGCCATGC------AACCTAGCCCTTGAGAGGCAGGGCAAGGAT-GAC----
--------------------------------------------------------------------------------
--------------------------------------------------------------------------------
--------------------------------------------------------------------------------
--------------------------------------------------AGCA-ATGCATAGATGTCATGCTCTGGCCA
CGCTATCCATGGGCCAGGGAACTAGATCAGCCATGACTGACCAGAGCCAGAGCTGGCATGG-------------------
-CTCGGACCCCACCGTGCCCAGTGCTGGCAGGAGGGAGACAGCGACTGAATCTGGTGGGAAACT
>Ami_scaffold-11218
GCTGCTCCTGGCAGTAG---CTGTGCAACAGAGGCACCCCCAGGATGAAGTCT------------GAGGGGTGAGGGCGC
GACTC--------------------------------------------------------------CGAGATGGGCACA
```

```
GCCCTCGTGGAGACCGCAGGGCAATCAGAGAGTGGGGCAGTGTGAGTCT--GGGGCAGGGAGCCAGGGTCCTGCCATGTC
AACCTTGGCTCTGACATGGGG-----CTTGGCTCCCTTATGGGGCTGCTGCCACCGCAGCTTTGCTGCCAGAGGCCCCTA
GTGCCAAGCAGCAGGCACCAGAAACCTCCTTTGTCAGCCCAGAAGGGGTTTACCAGTGCTTCAGTAAAAGGATGTACTA
TTTCCACCCCCGCCACACCTGACCTACCAATGCTGACAAAATAGCATAGGGGA---------------------------
------------------GATAAGCCACTGCCATGC--------------------------------------------
--------------------------------------------------------------------------------
--------------------------------------------------------------------------------
--------------------------------------------------------------------------------
--------------AACCTAG---CCCTTGAGAGGCAGGGCAAGGATGACAGCA-ATGCATAGATGTCATGCTCTGGCCA
CACTACCCAGGGGCCAGGGAACTAGATCAGCCATGACCGACCAGAGCCAGAGCTGGCATGG-------------------
-CTCGGACCCCACTGTGCCCAGTGCTGGCAGGAGGGAGACAGCGATTGAACTCGGTGGGAAACT
>Anolis_GB
ACTGCTTCGTGCAGTAG---CTGGAATGTAAATAAATCAAATGGTGAAAGAGCTTTTTAAACACAGAAGAGTAAAAGTAT
GATCTAAATACCCAACAGTCATCAGAAAAATCTTAAATTTGGAAAGACACAGAAAAATTGCCAGTGGAAAGAAGTGGAGA
GTATACATGCAGACAAAGGGAAAA-----------------ATCTATTT--GTATTCTGTATTTAAAGTTC---------
--ACTTGGTATTCAACCATTG-----CTTAGTTCACTTG-GCCCCTGTTGGCCACACAGTACATATTCCCAAACAGCAGT
TTTTCACATTATGTTGCATAACAATCTGCTCACTTATATAAAATTAGCTATAAACTATGAGT-----------------G
ATTCCACAT-------------CACACTGGATGTAGCAAAGTATTAAAGGTTAATTGTGGCTGACATTTGCATTTTTCCC
TTGTGAAGCATTTTCCTTAACAAACATCCTTTATAC------AGTATGGCATGTGATCAAAGCAATGATGAC-TGA----
--------------------------------------------------------------------------------
--------------------------------------------------------------------------------
--------------------------------------------------------------------------------
ATAGATTGGCAGAAAACTGATGAATGAATGACAAAAACAGATGGAAAAACAACACAAACATAAATGAGAAAGACAGGCAG
TAGTGGTGAACAGTGTGAACACTTGAACATTCATGCATGGCAAGAGATAGAGAAGACTCAA-------------------
------------------------------------------------------------AACT
```

GC03

```
>CR1-2_Crp
--------------------------------------------------------------------------------
------------------------------------------------------------CATAGTTTCATAGTTTCATA
GTTAGTAGGGTCGGAAGGGACCTGAG-CAGATCATCAAGTCCGACCCCCTGCCATG----------------GCAGGAAA
GAGTACTGGGGTCAAACGACCCCAGCAAGGTGSTCGTCAAGCCTCCTCTTAAAGACCCCCAGGGTAGGAGCCAGCACCAC
TTCGCTTGGAAGTTGGTTCCAGATCCTAGCCGCCCTGACAGTGAA-----------------------------------
--------------------------------------------------------------------------------
--------------------------------------------------------------------------------
--------------------------------------------------------------------------------
-----------------------------------------------
>Exons
G-------------------------------------------------------------------------------
--------------------------------------------------------------------------------
--------------------------------------------------------------------------------
--------------------------------------------------------------------------------
--------------------------------------------------------------------------------
--------------------------------------------------------------------------------
--------------------------------------------------------------------------------
--------------------------------------------------------------------------------
------------------------AATCGATACAAAATGGTGGTTTT
>Cpo_415_216550-218100
A---GTGAGTGCCGACACCACTTGCTCTAAAACCAAACCATTTTGCCTGGTTATTTATTATAGTGTCGCCATGTAGCTAT
GTCAAGTACGTACAGTTAACAGGGTCTTTCAGACCTAAAGAAAATTATATTGGCCTA---CAT-GTTTCATAGTTTCATA
GTTAGTAGGGTCAGAAGGGACCTGAG-CAGATCATCAAGTCCGACCCCCTGCCATG---TAATATTAATGTTGTGTTTCA
GACCATTTGCTTGTAAAGAA--------ATAGTTTCATAGTTGAGCATGCAGATCTGTACATTACTGATGATGTGAAATT
AGCATATATCATTAGCTGTTAAACACCTTAATGCTTTCAGATAATGTGCTTTAAGGAAGGCATTTTGGATCTTTCTGTCT
TTGTCCCTGTCATGGGACCAGTGCCTTAGTCTAGATCTTTCTATGGGCCTTGAGAGGGCTACGTCAGACCAGGCAGTTCT
CCCATCTCCAGTAAGATGCCTGGC-TCATTTCATGCTGAGATGTATAATTAAACAGACATGCTGGAATCCCCTAATTCTG
GCCATTGAATTCCACCACTGGAC-CAAGAAGACTTTATGGTGATTCTGGATTCATGCTGTTGTGTTTATT----------
---------TTCCCATGCCAG---AATCGATACAAAATGGTGGTTTT
>Ote
A---GTGAGTGCCGACACCACTTGCTCTAAAACCAAACCATTTTGCCTGGTTATTTATTATAGTGTCGCCATGTAGCTAT
GTCAAGTACGTACAGTTAACAGGGTCTTTCAGACCTAAAGAAAATTACATTGGCCTA---CAT-GTTTCATAGTTTCATA
GTTAGTAGGGTCAGAAGGGACCTGAG-CAGATCATCAAGTCCGACCCCCTGCCATG---TAATATTAATGTTGTGTTTCA
GAGCATTTGCTTGTAGAGAA--------ATAGTTTCATAGTTGAGCATGCAGATCTGTACATTACTAATGATATGAAATT
AGCATATATCATTAGCTGTTAAACACCTTAATGCTTTCAGATAATGTGCTTTAAGGAAGGCATTTTGGATCTTTCTGTCT
TTGTCCCTGCCATGGGACCAGTGCCTTAGTCTAGATCTTTCTATGGGCCTTGAGAGGGCTACGTCAGACCAGGCAGTTCT
CCCATCTCCAGTAAGATGCCTGGC-TCATTTCATGCTGAGATGTATAATTAAACAGACATGCTGGGATCCCCTCATTCTG
GCCATTGAATTCCAACACTGGAC-CAAGAAGGCTTTATGGTGATTCTGGATTCATGCTGTTGTGTTTATT----------
---------TTCCCATGCCAG---AATCGATACAAAATGGTGGTTTT
>Mca
-------------------------------------CCATTTTGCCTGGTTATTTATTATAGTGTCGCCATGTAGCTAT
GTCAAGTACGTACAGTTAACAGGGTCTTTCAGACCTAAAGAAAATTACATTGGCCTA---CAT-GTTTCATAGTTTCATA
GTTAGTAGGGTCAGAAGGGACCTGAG-CAGATCATCAAGTCCGACCCCCTGCCATG---TAATATTAATGTTGTGTTTCA
GACCATTTGCTTGTAGAGAA--------ATAGTTTCATAGTTGAGCATGCAGATCTGTACATTACTAATGATGTGAAATT
AGCATATATCATTAGCTGTTAAACACCTTAATGCTTTCAGATAATGTGCTTTAAGGAAGGCATTTTGGATCTTTCTGTCT
TTGTCCCTGCCATGCGACCAGTGCCTTAGTCTAGATCTTTCTATGGGCCCTGAGAGGGCTACGTCAGACCAGGCAGTTCT
CCCATCTCCAGTAAGATGCCTGGC-TCATTTCATGCTGAGATGTATAATTAAACAGACATGCTGGAATCCCCTCATTCTG
GCCATTGAATTCCACCACTGGAC-CAAGAAGGCTTTATGGTGATTCTGGATTCATGCTGTTGTGTTTATT----------
---------TTCCCATGCCAG---AATCGATACAAAATGGTGGTTTT
>Tsc
```

```
G---GTGAGTGCTGACACCACCTGCTCTAAAACCAAACCGTTTTGCCTTGTTATTTATTATAGTGTTGCCATGTAGCTAC
GTCAACTACGTACAGTTAACAGGGTCTTTCAGACCTAAAGAAAATTACATTCGCCTA---CAT-GTTTCATAGTTTCATA
GTTAGTAGGGTCGGAAGGGACCTGAG-CAGATCATCAAGTCCGACCCTCTGCCATG---TAATATTAATGTTGTGTTTCA
GACCATTTGCTTGTAGAGAA--------ATAGTTTCATAGTTGAGCATGCAGATCTGTACATTACTAATGATGTGAAATT
AGCATATATTGTTAGCTGTTAAACATCTTAATGCTTTCAGATAATGTGCTTTAAGGAAGGCATTTTGGATCTTTCTGTCT
TTGTCCCTGCCATGGGACCAGTGCCTTAGTCTGGATCTTTCTATGGGCCTT---------------GACAAGGCAGTTCT
CCCATCACCAGTAAGATGCCTGGC-TCATTTCATGCTGAGATGTACAATTAAACAGACATGCTGGGATCCCCTAATTCTG
GCCATTGAATTCCACCACTGGAC-CAAGAAGGCTTTATGGTGATTCTGGATTCATGCTGTTGTGTTTATT----------
---------TTCCCGTGCCAG---AATCGATACAAAATGGTGGTTTT
>Gga_scaffold20959
G---GTGAGTGCTGACACCACCTGCTCTAAAACCAAGCCGTTTTGCCTGGTTATTTATTATAGTGTCGCCACGTAGCTAT
GTCAACTACGTACAGTTAACAGGGTCTTTCAGACCTAAAGAAAATTACATTCGCCTA---CAT-GTTTCATAGTTTCATA
GTTAGTAGGGTCGGAAGGGACCTGAGCCAGATCATCAAGTCCGACCCCCTGCCATG---TAATATTAATGTTGTGTTTCA
GACCATTTGCTTGTAGAGAA--------ATAGTTTCATAGTTGAGCATGCAGATCTGTACATTACTAATGATGTGAAATT
AGCATATATTGTTGGCTGTTAAACATCTTAATGCTTTCAGACAATGTGCTTTAAGGAAGGCATTTTGGATCTTTCTGTCT
TTGTCCCTGCCATGGGACCAGTGCCTTAGTCTGGATCTTTCTATGGGCCTTGACAGGGCTACGTCAGACAAGGCAGTTCT
CCCATCACCAGTAAGATGCCTGGC-TCATTTCATGCTGAGATGTACAATTAAACAGACATGCTGGGATCCCCTAATTCTG
GCCATTGAATTCCACCACTGGAC-CAAGAAGGCTTTATGGTGATTCTGGATTCATGCTGTTGTGTTTATT----------
---------TTCCCGTGCCAG---AATCGATACAAAATGGTGGTTTT
>Cya
G---GTGAGTGCTGACACCACCTGCTCTAAAACCAACCCGTTTTGCCTGGTTATTTATTATAGAGTCGCCATGTAGCTAT
GTCAACCACATACAGGTAACAGGATCTTTCAGACCTAGAGAAAATTACATTGGCCTA-----------------------
---------------------------------------------------CATG---TAATATTAATGTTGTGTTTCA
GACCATTTGCTTGTAGAGAA--------ATAGTTTCATAGCTGAGCATGCAGATCTGTACATTACTACTGCTGTGAAATT
AGCATGTATCATTAGCTGTTAAACACCTTAATGCTTTCAGGTAATGTGCTTTAAGGAAGGCATTTTGGATCTTTCTGTCT
TCGTCCCTGCCATGGGACCAGTGCCTTAGTCTGGATCTTTCTATGGGCCTTGACAGGGCTGTGTCAGACCAGGCAGTTCC
CCCATCTCCAGTAAGATGCCTGGC-TCCTTTCATGCTGAGATGTACAATTAAACAGACATGCTGGGATCCCCTCATTCTG
GCCATTGAATTTCACCGCTGGAC-CAAGGGGGCTTTATGGTGATTCTGGATTCATGGTGTTTTGTTTATT----------
---------TTCCCATGTTAG---CATCGAGACAAAATGGTGGTTTT
>Asi_533401603
G---GTGAGTGCTGACACCACCTACTCTAAAACCAAACCGTTTTGCCTGGTTATTTATTGTAGTGTCGCCGTGTAGCTAT
GTCAACCACATACAGTTAACAGGGTCTTTCAGACCTAAAGAAAATTATATTGGCCTA-----------------------
---------------------------------------------------CATG---TAATATTACTGTTGTGTTTCA
GACCATTTGCTTGTAGAGAAATAGTTTCATAGTT--------GAGCATGCAGATCTGTATATTACTAATGATGTGAAATT
AGCATATATCATTAGCTGTTAAACACCTTAATGCTTTCAGATAATGTGCTTTAAGGAAAGCAGTTTGGATCTTTTTGTCT
TTGTCCCTGCCATGGGACCAGTGCCTTAGTTTGGATCTTTCTATGGGCCTTGACAGGGCTATGTCAGACCAGGCAGTTCC
CCCATCTCCAGTAAGATGCCTGGC-TCCTTTCATGCTGAGATGTACAATTAAACAGACATGCTGGGATCCCCTAATTCTG
GCCATTGAATATCACCGCTGGAC-CAAGACGGCTTTATGGTGATTCTGGATTCATGGTGTTGTGTTTATT----------
---------TTCCCATGCCAG---AATCGATACAAAATGGTGGTTTT
>Ami_scaffold-11084
G---GTGAGTGCTGACACCACCTACTCTAAAACCAAACCATTTTGCCTGGTTATTTATTATAGAGTCGCCATGTAGCTAT
GTCAGCCACATACAGTTAACAGGGTCTTTCAGACCTAAAGAAAATTACATTGGCCTA-----------------------
---------------------------------------------------CATG---TAATATTAATGTTGTGTTTCA
GACCATTTGCTTGTAGAGAAATAGTTTCATAGTTTCATAGTTGAGCATGCAGATCTGTACATTACTAATGATGTGAAATT
AGCATATATCATTAGCTGTTAAATGCCTTAATGCTTTCAGATAATGTGCTTCAAGGAAGGCATTTTGAATCTTTCTGTCT
TTGTCCCTGCCATGGGACCAGTGCCTTAGTCTGGATCTTTCTATGGGTCTTGACAAAGCTATGTCAGACCAGGCAGTTCC
CCCATCTCCAGTAAGATGCCTGGC-TCCTTTCATGCTGAGATGTACAATTAAACAGACATGCTGGGATCCCCTAATTCTG
GCCATTGAATTTCACCGCTGGAC-CAAGACGGCTTTATGGTGATTCTGGATTCATGGTGTTGTGTTTATT----------
---------TTCCCATGCCAG---AATCGATACAAAATGGTGGTTTT
>Gallus_GB
A---GTGAGTACT---------------------------TTTTACTA--------------------------------
------------------------------AAAGTTAAAGACAGCAACAGT-----------------------------
---------------------------------------------------TTTT---GCACCACCTTGAGGTGGTTTT
GATCCTTTGGTTGTCCTAAG---------------------------------------------------CTGAAGTT
GTCCTAAGCTGCT-------------------------------------------------------------------
-------------------------------TTGCTTTTCTCATGAAGCCTAACAGAATTA-------------------
---ACTTCCAGTGGGATGTTTAATGTTTTTTCATACTGACAGCCTTAACTGAATAAACCCTTAGGGATAGCCTAATTGCA
GCCACTGAACTCCAACCCTGAA--AAAGAAAATCCTCTGGTGAGTTCACATTTCTGATGGTACATTCGTTGTTGATGGTG
GGTTTTCTCCCTTCTTCATAG---AATCGGTACAAAATGGTGGTTTT
```

GC04

```
>CR1-7B
--------------------------------------------------------------------------------
--------------------------------------------------------------------------------
--------------------------------------------------------------------------------
--------------------------------------------------------------------------------
------------------TA----GTTTCATAGTTTCATAGTTTCTAGGGTCGGAAGGGACCTGAGCAGATCATCTAGTC
CGACCCCCTGCCATGGCAGGAAAGAGTACTGGGGTCAAACGACCCCAGCAAGGTGTTCATCTAAC---------------
-----------------------------------------------------------------------CTCCTCTT
AAAAACCCCCAGGGTAGGAGCCTGCACCACCTCCCTTGGAAGTTGGTTCCAGACCCTAGCCGCCCTGACCGTGAAATAGC
GCCTCCTGATGTCTAACCTAAACCTACCCTCTGCCAGCTTGTGACCGTTGTTCCTGGTCACTCCTGGCTGTGCTCGGGGG
AACAGTGACTCCCCCAATGCCTGCTGATCCCCCCTGACCAGTTTGTAACAGGCCACAAGATCCCCCCTCAGCCTTCTCTT
TTGGAGGCTGAACAGGTTCAGGTCCCGTAGCCTCTCCT-----------------------------CATAGGGCCTGC
CCTGCAGTCCCCTGATCATGCGAGTGGCCCTCCTCTGGACCCTCTCCATGCTGTCCACATCCCTCTTGAAGTGCGGCGCC
CAGAACTGGACGCAGTACTCCAACTGCGGCCTGACCAGTGCCGCATAGAGGGGGAGG-----------------------
--------------------------------------------------------------------------------
--------------------------------------------------------------------------------
--------------------------------------------------------------------------------
```

```
--------------------------------------------------------------------------------
--------------------------------------
>Exons_proposal
AATTCTATCAGATGCATGGTTCCACTTTGCATT-----------------------------------------------
--------------------------------------------------------------------------------
--------------------------------------------------------------------------------
--------------------------------------------------------------------------------
--------------------------------------------------------------------------------
--------------------------------------------------------------------------------
--------------------------------------------------------------------------------
--------------------------------------------------------------------------------
--------------------------------------------------------------------------------
--------------------------------------------------------------------------------
--------------------------------------------------------------------------------
--------------------------------------------------------------------------------
--------------------------------------------------------------------------------
--------------------------------------------------------------------------------
--------------------------------------------------------------------------------
--------------------------------------------------------------------------------
--------------------------------------------------------------------------------
----------------------TTTTATACAAGCTGTG
>Cpo_415_357400-358950
AATTCTATCAGATGCATGGTTCCACTTTGCATT---GTAAGCCTAACAT-GTAAATAATTGTTGCTTCTGAAATAACTTT
CATTCTGTTAGTTATATATCTTTGATTGGCTGAAAATAGAATCCTTTCACTGTCAATCACATTTCTTTA------CATTT
ATTTGAGAAAATATTATAATTTTAGATGTAACTTTAGTGTAAGACACTGCTCATTGTCTGGCACCATGTTTAGTTAAACT
GATCAGTAATTATTCT--GAATTAATAGATGCAAACTTCTGGCCCCAGGAGTATTTGTTCAATATATGTAGCTGTCTCTA
---ATTT-CTTGAGAGACTTT---ATTTCATAGTTTCATAGTTTCTAGGGTTGGAAGGGACCTGAGCAGATCATCGAGTC
CGACCCCCTGCCATGGCAGGAAAGAGTACTGGAGTGAAACAACCCCAGCAAGGTGTACATCTAGC---------------
------------------------------------------------------------------------CTCCTCTT
AAAGCCCCCCAGGGTAGGAGCCTGCACCACTTCTCTTGGCAGTTGGTTCCGGATCCTAGCTGCCCTGACCGTGAAGTCGC
GCCTCCTGATGTTGAGCCTGAATCTCCCCTCTGCCAGCTTATGACCGTTATTCCTGCTCACTCCTGGCGGTGCTCGGGGG
AACAGGGACTCCCCCAGTGCCTGCTGCTCCCCTCTGACTAGTTTGTAACAGGCCACGAGATCCCCCCTCAGCCTTCTCTT
GTGGAGGCTGAACAGGTTCAGGTCCCGTCGCCTCTCCT---ATTTCTGAAAGTGCTGT--AGGATAGAGGGCTATATTTT
GTTTAAAAAGAAAT--GTCATT-GGGGCCGGATCCTGACCTTCATCATCTTTGTGTTGCTTTGAGGAAAAAGCTAACAAA
GCTGCCCTTACTGGCT-GGTTGGGTCTTTTATATATGGGAATCCTGAAGTGGCTTGAAGTCAGCACT-GGGATTCCTATT
CTATCCTCTCCTGGTC--------------------------------------TTGGCAAAGGAAACATGACTG-----
---------GGGGATCAGTCAAGAT-------------------CCTTATA-----------CTATTCCTAGACAATCCT
TTCCTACTGTTAAATTAT---AATAATACACATAGGAAAAAGCAGGATGGGAAAGTGAAATTAGATGTTAAACATAAAGA
AATAAGGAAGTTCAATATTACTGTTCTCCATT-ATCTATTTTAAGTTTGTGTGTTGTGTAAGAATATT---------TAC
ACGATTTTTTTTA--ACAG---TTTTATACAAGCTGTG
>Ote
------------------------------------GTAAGCCTAACATGGTAAATAATTGTTGCTTCTGAAATAACTTT
CATTCTGTTAGTTATATATCTTTGATTGGCTGAAAATAGAATCCTTTCACTGTCAATCACATTTCTTTA------CATTT
ATTTGAGGCAATATTATAATTTTAGATGTAACTTTAGTGTAAGACACTGCTCATTATCTGGCACCATGTTTAGTTAAACT
GATCAGTAATTATTCT--GAATTAATAGATGCAAACTTCTGGCCCCAGGAGTATTTGTTCAATATATGTAGCTGTCTCTA
---ATTT-CTTGAGAGACTTG---ATTTCATAGTTTCATAGTTTCTAGGGTTGGAAGGGACCTGAGCAGATCATCGAGTC
CAACCCCCTGCCATGGCAGGAAAGAGTACTGGAGTGAAACAACCCCAGCAAGGTGTACATCTAGC---------------
------------------------------------------------------------------------CTCCTCTT
AAAGCCCCCCAGGGTAGGAGCCAGCACCACTTCTCTTGGAAGTTGGTTCCGGATCCTAGCCGCCCTGACCGTGAAGTCGC
GCCTCCTGATGTTGAGCCTGAATCTCCCCTCTGCCAGCTTGTGACCGGTATTCCTGCTCACTCCTGGCAGTGCTCGGGGG
AACAGGGATTCCCCCAGTGCCTGCTGCTCCCCTCTGACTAGTTTGTAACAGGCCACTAGATCCCCCCTCAGCCTTCTCTT
GTGGAGGCTGAACAGGTTCAGGTCCCGTAGCCTCTCCT---ATTTCTAAAAGTGCTAT--AGGATAGAGGGCTATATTTT
GTTTAAAAAGAAAT--GTCATT-GGGGCCGGATCCTGACCTTCATCATCTTTGTGTTGCTTTGAGGAAAAAGCTAACAAA
GCTGCCCTTACTGGCT-GGTTGGGGCTTTTATATATGGGAATCCTGAAGTGGCTTGAAGTCAGCATT-GGGGTTCCTATT
CTATCCTCTCCTGGTC--------------------------------------TTGGCAAAGGAAACATGACTG-----
---------GGGGATCAGTC-AGAT-------------------CCTTATA-----------CTATTCCTAGACAATCCT
TTCCTACTGTTAAATTAT---AATAATACACATAGGAAAAAGCAGGATGGGAAAGTGAAATTAGATGTTAAACATAAAGA
AATAAGGAAGTTCAATATTACTGTTCTCCATT-ATCTATTTTAAGTTTCTGTGTTGTGTAAGAATATT---------TAC
A-------------------------------------
>Mca
-----------------------------------------CCTAACAT-GTAAATAATTGTTGCTTCTGAAATAACTTT
CATTCTGTTAGTTATATATCTTTGATTGGCTGAAAATAGAATCCTTTCACTGTCAATCACATTTCTTTA------CATTT
ATTTGAGGCAATATTATAATTTTAGATGTAACTTTAGTGTAAGACACTGCTCATTATCTGGCACCATGTTTAGTTAAACT
GATCAGTAATTATTCT--GAATTAATAGATGCAAACTTCTGGCCCCAGGAGTATTTGTTCAATATATGTAGCTGTCTCTA
---ATTT-CTTGAGAGACTTT---ATTTCATAGTTTCATAGTTTCTAGGGTTGGAAGGGACCTGAGCAGATCACCTAGTC
CGACCCCCTGCCATGGCAGGAAAGAGTACTGGAGTGAAACAACCCCAGCAAGGTGTACATCTAGC---------------
------------------------------------------------------------------------CTCCTCTA
AAAGCCCCCCAGGGTAGGAGCCAGCACCACTTCTCTTGGAAGTTGGTTCCGGATCCTAGCCGCCCTGACCGTGAAGTCGC
GCCTCCTGATGTTGAGCCTGAATCTCCCCTCTGCCAGCTTGTGACCGGTATTCCTGGTCACTCCTGGCGGTGCTCGGGGG
AACAGGGACTCCCCCAATGCCTGCTGGTCCCCTCTGACTAGTTTGTAACAGGCCACTAGATCCCCCCTCAGCCTTCTCTC
GTGGAGGCTGAACAGGTTCAGGTCCCGTAGCCTCTCCT---ATTTCTAAAAGTGCTGT--AGGATAAAGGGCTATATTTT
GTTTAAAAAGAAAT--GTCATT-GGGGCCGGATCCTGACCTTCATCATCTTTGTGTTGCTTTGAGGAAAAAGCTAACAAA
GCTGCCCTTACTGGCT-GGTTGGGTCTTTTATATATGGGAATCCTGAAGTGGCTTGAAGTCAGCATT-GGGATTCCTATT
CTATCCTCTCCTGGTC--------------------------------------TTGGCAAAGGAAACATGACTG-----
---------GGGGATCAGTCAAGAT-------------------CCTTATA-----------CTATTCCTAGACAATCCT
TTCCTACTGTTAAATTAT---AATAATACACATAGGAAAAAGCAGGATGGGAAAGTGAAATTAGATGTTAAACATAAAGA
AATAAGGAAGTTCAATATTACTGTTCTCCATT-ATCTATTTTAAGTTTGTGTGTTGTGTAAGAATATT---------TAC
ACAA----------------------------------
```

>Tsc
--CTAACAT-GTAAATAG--GTTGCTTCTGAAATAACTTT
CATTCTGTTAGTTATATATCTTTGATTGGCTGAAAATAGAATCCTTTCACTGTCAATCACATTTCTTTA------CATTT
ATTTGAGGCAATATTATAATTTTAGATGTAACTTTAGTGTAAGACACTGCTCATTATCTGGCACCATGTTTAGTTAAACT
GATCAGTAATTATTCT--GAATTAATAGGTGCAAACTTCTGCCCCCAGGAGTATTTGTTCAATATATGTAGCTATCTCTA
---ATTT-CTTGAGAGACTTT---ATTTCATAGTTTCATAGTTTCTAGGGTTGGAAGAGACCTGAGCAGATCATCGAGTC
CGACCCCCTGCCATGGCAGGAAAGAGTACTGGGGTCAAATGACCCCGGCAAGGTGATTGTCTAGC---------------
--CTCCTCTT
AAAGATCCCCAGGGTAGGAGCCAGCACCACTTTGCTTGGAAGTTGGTTCCAGATCCTAGCCGCCCTGACAGTGAAGTCAC
GCCTCCTGATGTCTAGTCTGAATCTCCCCTCTGCCAGCTTGTGACCGTTATTCCTGGTCACTCCTGGTAGTGCTCAGGGG
AACAGGGACTCCCCCAATGCCTGCTGCTCCCCTCTGACTAGTTTGTAACAGGCCACTAGATCCCCCCTCAGCCTTCTCTT
GTGGAGGCTGAACAGGTTCAGGTCCCGTAGCCTCTCCT---ATTTCTAAAAGTGCTGT--AGGATAGAGGGCTATATTTT
GTTTAAAAAGAAATAAGTCACT-GGGGCCGGATCCTGACCTTCATCATCTTTGGCTTGCTTTGAGGAAAAAGCTAACAAA
GCTGCCCTTACTGGCT-GGTTGGGGCTTTTATATATGGGAATCGTGAAGTGGCTTGAAGTCAGCATT-GGGATTCCTATT
CTATCCTCTCCTGGTC--------------------------------------TTGGCAAAGGAAATATGACTG-----
---------GGGGATCAGCCAAGAT-------------------CCTTATA-----------CCATTCCTAGACAATCCT
TTCCTACTGTTAAATTAT---AATAATACACACAGGAAAAAGCAGGATGGGAAAGTGAAATTAGATGTTAAACATAAAGA
AAAAAGGAAGTTCAATATTACTGTTCTCCATT-ATCTATTTTAAGTTTGTGTGTTGTGTAAGAATATT------------
----
>Gga_scaffold18589
AATTCTATCAGATGCATGGTTCCACTTTGCATT---GTAAGCCTAACAT-GTAAATAACTGTTGCTTCTGAAATAACTTT
CATTCTGTTAGTTATATATCTTTGACTGGCTGAAAATAGAATCCTTTCACTGTCAATCACATTTCTTTA------CATTT
ATTTGAGGCAATATTATAATTTTAGATGTAACTTTAGTGTAAGACACTGCTCATTATCTGGCACCATGTTTAGTTAAACT
GATCAGTAATTGTTCT--GAATTAATAGATGCAAACTTCTGCCCCCAGGAGTATTTGTTCAATATATGTAGCTATCTCTA
---ATTT-CTTGAGAGACTTT---ATTTCATAGTTTCATAGTTTCTAGGGTTGGAAGGGACCTGAGCAGATCATCAAGTC
CGACCCCCTGCCATGGCAGGAAAGAGTACTGGGGTCAAACGACCCCAGCAAGGTGTACATCTAGCNNNNNNNNNNNNNNN
NNNNNNNNNNNNNNNNNNNNNNNNNNNNNNNNNNNNNNNNNNNNNNNNNNNNNNNNNNNNNNNNNNNNNNNNNNNNNNNN
NNNNNCCCCCAGGGTAGGAGCCAGCACCACTTCTCTTGGAAGTTGGTTCCAGATCCTAGCCGCCCTGACCGTGAAGTCAC
GCCTCCTGATGTCTAGTCTGAATCTCCCCTCTGCCAGCTTGTGACCGTTATTCCTGGTCACTCCTGGTAGCACTCGGTGG
AACAGGGACTCCCCCAATGCCTGCTGCTCCCCTCTGACTAGTTTGTAACAGGCCACTAGATCCCCCCTCAGCCTTCTCTT
GTGGAGGCTGAACAGGTTCAGGTCCCGTAGCCTCTCCT---ATTTCTAAAAGTGCTGT--AGGATAGAGGGCTATATTTT
GTTTAAAAAGAAATAAGTCACT-GGGGCTGGATCCTGACCTTCATCATCTTTGTGTTGCTTTGAGGAAAAAGCTAACAAA
GCTGCCCTTACTGGCT-GGTTGGGGCTTTTATATATGGGAATCCTGAAGTGGCTTGAAGTCAGCATT-GGGATTCCTATT
CTATCCTCTCCTGGTC--------------------------------------TTGGCAAAGGAAATATGACTG-----
---------GGGGATCAGTCAAGAT-------------------CCTTATA-----------CTATTCCTAGACAATCCT
TTCCTACTGTTAAATTAT---AATAATACACACAGGAAAAAGCAGGATGGGAAAGTGAAATTAGATGTTAAACATAAAGA
AAAAAGGAAGTTCAATATTACTGTTCTCCATT-ATCTATTTTAAGTTTGTGTGTTGTGTAAGAATATT---------TAC
AAGATTTTTTTTTA-ACAG---TTTTATACAAGCTGTG
>Cya
AATTCTATCAGATGCATGGTTCCACTTTGCATT---GTAAGCCTAACAT-GTAAATAATTGTTGCTTCTGAAATAACATT
CATTCTGTTAGCTATATATCTTTAGGCTGCTGAAAATATAAGACTTTTGCTGTCCATCACATTTCTTTA------CACCT
ATTTGAGGCAATATTATCATTTTAGATGTAACTTCAGTGTAAGACACTGCTCATTAT-TGGCACCATGTTTAGTTAATCT
GATCAGGGATTATTCT--GAATTACTAGATGCAAACTTCTGCCCCACACAGTATTTGTTCAATATATGTGGCTATCTCTA
---ATTT-CTTGCGAGACTTT---

---ATTTCTAAAAGTGCTGT--AGGATAGAGGGCTATATTTT
GTTTAAAAAGAAATAAGTCATT--GGGCCAGAGCCTGACCTTCATCATCTTTGTGTTGCTTTGA-GAAAAAGCTAATAAA
GCTGGCCTTACTGGCT-GGTTGGGGCTTTTATACATGGGAATCCTCAAGTGGCTTGAAGTCAGCTTT-CGGGTGCCTATT
CTGTCCTCTCCTGGTC--------------------------------------TTGGCAAAGGAAACATGACTG-----
---------GGGGATCAGTCAAGAT-------------------CCTTATA-----------CTATTCCTAGATAATCCT
TTCCTACTGTTAAATTAT---ACTAATGCACACAGGAAAAAGCAGGATGGGAAAGTGAAGGTAGACATTAAACATAAAGA
AATAAGGAAGTTCAATATTACTGTTCTCCATT-ATCTATTTTAAGTGTGAGTCTTGTGTAAGAATATT---------TAC
AAGATTTTTTTTTTAACAG---TTTTATACAAGCTGTG
>Asi_533401628
AATTCTATCAGATGCCTGGTTCCACTTTGCATT---GTAAGCCTAACAT-GTAAATAATTGTTGCTTCTGAAATAACGTT
CATTCTGTTAGCTATATATCTTTGAGTTGCTGAAAATATAAGACTTTTGCTGTCGATCACATTTCTTTA------CACTT
ATTTGCGGCAATATTATCATTTTAGATGTAACTTTAGTGTAAGACACTGCTCATTATCTGGCACCATGTTTAGTTAAACT
GATCAGTAATTATTCT--GAATTAATAGATGCAAACTTCTGCCCCCCAGAGTATTTGTTCAATATATGTGGCTATCTCTA
---ATTT-CTTGCGAGACTTT---

---ATTTCTAAAAGTGCTGT--AGGATAGAGGGCTATATTTT
GTTTAAAAAGAAATAAGTCATT--GGGCCAGATCCTGACCTTCATCATCTTTGTATTGCTTTGA-GAAAAAGCTAATAAA
GCTGGCCTTACTGGCT-GGTTGGGGCTTTTATATATGGGAATCCTCAAGTGGCTTGAAGTCAGCATT-GGGATTCCTGTT
CTATCCTCTCCTGGTC--------------------------------------TTGGCAAAG-AAACATGACTG-----
---------GGGGATCAGTCAAGAT-------------------CCTTATA-----------CTATTCCTAGATAATCCT
TTCCTATTGTTAAATTAT---ACTAATACACACAGGAAAAAGCAGGATGGGAAAGTGAAATTAGACGTTAAACTTAAGGA
AATAAGGAAGTTCAATATTACTGTTCTCCATT-ATCTATTTTAAGTGTGTGTGTTGTGGAAGAATATT---------TAC
AAGATTTTGTTTTTAACAG---TTTTATACAAGCCGTG
>Ami_scaffold-11084
AATTCTATCAGATGCATGGTTCCACTTTGCATT---GTAAGCCTAACAT-GTAAATAGTTGTTGCTTCTGAAATAACATT

CATTCTGTTAGCTATATATCTTTGAGTTGCTGAAAATATAAGACTTTTGCTGTCGATCACATTTCTTTA------CACTT
ATTTGAGGCAATATTATCATTTTAGATGTAACTTTAGTGTAAGACACTGCTCATTATCTGGCACCATGTTTAGTTAAACT
GATCAGTAATTATTCT--GAATTAATAGATGCAAACTTCTGCCCCCCGGAGTATTTGTTCAATATATGTGGCTATCTATA
---ATTT-CTTGCGAGACTTT---

---ATTTCTAAAAGTGCTGT--AGGATAGAGGGCTATATTTT
GTTTAAAAAGAAATAAGTCATT--GGGCCAGATCCTGACCTTCATCATCTTTTTGTTGCTTTGA-GAAAAAGCTAATAAA
GCTGGCCTTACTGGCT-GGTTGAGGCTTTTATATATGGGAATCCTCAAGTGGCTTGAAGTCAGCATT-GGGATTCCTATT
CTATCCTCTCCTGGTC--------------------------------------TTGGCAAAGGAAACATGACTG-----
---------GGGGATCAGTCAAGAT-------------------CCTTATA-----------CTATTCCTAGATAATCCT
TTCCTATTGTTAAATTAT---ACTAATACACACAGGAAAAAGCAGGATGGGAAAGTGAAATTAGACGTTAAACATAAAGA
AATAAGGAAGTTCAATATTACTGTTCTCCATT-ATCTATTTTAAGTGTGTGTGTTGTGTAAGAATATT---------TAC
AAGATTTTGTTTTTAACAG---TTTTATACAAGCTGTG
>Gallus_GB
AATTCTTTCTGATGCATGGTTCCACTTTGCATT---GTAAGTCTGTCAT-GCATATATTTTATACAGTATATATCATACA
TATCTAATCCATGAT-------------TACAGAGAATGACTCCTTCAGTAGATGCTCAATTTTCTTTAGATTATCAGTT
TTCAGAGAGATGGATGTAATCTTCCATGCAGCTTTTCCTTAGCACATGCT------------------GATGCTTTCACT
GGTCAATAATAGTACTGGGGATTGATGTGTACAGGCTGT--------------TTTTCACAAGGTGCAGTACTGTCTTTT
---ATTTCCTTCACATGCTGC---ATCTCTTCATGCTGTA--
----------------------TATAGACAAATACACGTAGGAAACCTGGTTAATAAACTAAAT----------------
----
--------------------------CTAGCTTTACTCCTCCTGGAAG--------------------------------
--AAAACA
CGGTGTAAAGCTGGTTCAGCTGGCTAGTGGCAGTATCCCATCAGCTGGTCTTCATAAAGCACAGGGGATTTCCTCTCCTT
TCT--AGAATAAGATCAT
ATATCAATAAAAACAGGTAGCTTAACTCTACAGCCTTTCTAATATCAATCTTGTGCTGCCAG------------------
-----------------AATCTGGTCTATATCCTATGAGAAT--------------------------GCACTGCATGTT
CTGTGTTCTCCTTGCTGAGAGGTTCATGGAAACTATTGATTTCAAATATCTCTGTTAGCAAAATCAGTTGAACTGCTGTT
CAGGGGAGAAGAGTTCATTTAAAGTAAGTTTTAATAAGGATAGCCATTATATGTTAACGTACCTAGACTTCAACTCATTT
ACTTTATTGTTAAATTATTTTGGTTATGTTCATAGGGGAGCATGGAA-AGCAATATGAAAGTAGAGTTGAAGCCTAAGAA
GCAGTTGTTTTCATATATTTCCATTATGCTAT-----------------TGGTTTTGTGCAGTAATTTCCCTTTTACAAC
GTGTGTTCTTTTTTAACAG---TTTTATACAAGCTATG

GC08

>CR1-2

--CTGTTACAAA
CTAGTCAGGGGGGACCAGCAGGCACTGGGGGAGTCCCTGTTCCCCCGAGCACTGCCAGGAGTGACCAGGAAATAACGGTC
ACAAGCTGGCAGAGGGTAGATTCAGATTAGACATCAGGAGGCGC--------TACTTCACTGTCAGGGCGGCTAGGATCT
GGAACCAACTTCCAAGCGAAGTGGTGCTGGCTCCTACCCTGGGG--------------GTCTTTAAGAGGAGGCTTGACG
AWCACCTTGCTGGGGTCGTTTGACCCCAGTACTCTTTCCTGCCATGGCAGGGGGTCGGACTTGATGATCTGCTCAGGTCC
CTTCCGACCCTACTAACTATGAAACTATGAAA---CTATG--

>Exons_rc
TGTTAATGACAACACCACGGGCAGTGC---

CTTCCACACA
>Cpo_719_96700-98250
TGTTAATGACAACACCACGGGCAGTGC---CTGGAGGACAGAGA-------------------------CAAGCTGCAAT
ACAGTACTTGCTGAGGCCAGACTCAAGAAAACTTTCAGCT----CCAGCAGGCTGGAAGAAGCCATTCCAA---------
-------GTCTCTATATGGGTGTTTGACTGTGGAGTGAGAAGTGTGAGGCCATCAGACCACAATGCCCCTTGTTGTTCCT
ATCTACATCAAGGGC---------TCTACCTATGGATTGCTAACC-----TGTACTTTAGTGTCAGGGTGCCTAGGATCT
GGAATCAACTTCCAAGAGAAGTGGTGCTGGCTCTTACCCTGGGG--------------GTCTTTAAGAAGAGACTAGATG
AACACCTTGCTGGGGTTGTTTGACCTCCGTACTCTTTCCTCCCATGGCAGGGGGTTGGACTTGGTGATCTGCTCACGTCC
CTTCTGACCCTAGCAACTACGAAACTATAACC---CAGCCCCAGTGAAGGGATTTCTACAGGGGTAGGGCTGCCTATCAC
CAGGGATCCCATTTTTCCCGTTTAAAA------AGAAAAATGACTTCTATAAGTAGGAGTTCAGATACCAG--------A
CACTTTCTGACAGCATCATATCTGCTCGAGGCTTTCTTTAATCC-AAATTAAAGGAACAGA------------------A
GTACAGCGTTTAGGAGAAATA-AGAAAGATACTAAAGCAGCTTTAAACTTTCCCTACTAAATTACTGCATAATAT--GAC
TTTTTACTAGATACAGCCT-TTCTAGGAGCTAATATTCTATTCATGTTTGGGTGTGGAGCAGAGAACCACATACATGTTT

ACTGTTGCTATTCAAGTTTCCTGATTAAGTGTTCTAATTCCCAGTGTTACATCTGAGAGAATGTGAACATCACATAC---
CTTCCACACA
>Ote
TGTTAATGACAACACCACGGGCAGTGC---CTGGAGGAGAGAGA-------------------------CAAGCTGCAAT
ACAGTAC-TGCTGAGGCCAGACTCAAGAGAACTTTCAGCT----CCAGCAGGCTGGAAGAAGCCATTCCAA---------
-------GTCTCTATATGGGTGTTTGACTGAGGAGTGAGAAGTGTGAGGCCATCAGACCACAATGCCCCTTGTTGTTCCT
ATCTACATCAAGGGC---------TCTACCTATGGATTGCTAACC-----TGTACTTTAGTGTCAGGGTGCCTAGGATCT
GGAATCAACTTCCAAGAGAAGTGGTGC-AGCTCTTACCCTGGGG--------------ATCTTTAAGAAGAGACTAGATG
AACACCTTGCTGGGGTTGTTTGACCTCCGTACTCTTTCCTCCCATGGCAGGGGGTTGGACTTGGTGATCTGCTCACGTCC
CTTCTCACCCTACCAATTACGAAACTATAACC---CAGCCCCAGTGAAGGGATTTCTACAGGGGTAGGGCCGCCTATCAC
CTGGGATCCCATTTTTCCCGTTTAAAA------AGAAAAATGACTTCTATAAGTAGGAGTTCAGATACCAG--------A
CACTTTCTGACAGCATCATATCTGCTCGTGGCTTTCTTTAATCC-AAATTAAAGGAACAGA------------------A
GTACAGCGTTTAGGAGAAATA-AGAAAAATACCAAAGCAGCTTTAAACTTTCCCTACTAAATTACTGCATAGTAT--GAC
TTTTTACTAGATACAGCCT-TTCTAGGAGCTAATATTCTATTCATGTTTGGGTGTGGAGCAGAGAACCACATATATGTTT
ACTGTTGCTATTCAAGTTTCCTGATTAAGTGTTCTAATTCCCAGTGTTACATCTGAGAGAATGTGAACATCACATAC---
CTTCCACACA
>Mca
------------------------------------GAGAGAGA-------------------------CAAGCTGCAAT
ACAGTACTTGCTGAGGCCAGACTCAAGAGAACTTTCAGCT----CCAGCAGGCTGGAAGAAGCCATTCCAA---------
-------GTCTCTATATGGATGTTGGACTGAGGAGTGAGAAGTGTGAGGCCATCAGACCACAATGCCCCTTGTTGTTCCT
ATCTACATCAAGGGC---------TCTACCTATGGATTGCTAACC-----TGTACTTTAGTGTCAGGGTGCCTAGGATCT
GGAATCAACTTCCAAGAGAAGTGGTGCTGGCTCTTACCCTGGGG--------------GTCTTTAAGAAGAGACTAGATG
AACACCTTGCTGGGGTTGTTTGACCTCCATACTCTTTCCTCCCATGGCAGGGGGTTGGACTTGGTGATCTGCTCACGTCC
CTTCTGACCCTACCAATTACGAAACTATAACC---CAGCCCCAGTGAAGGGATTTCTACAGGGGTAGGGCCGCCTATCAC
CAGGGATCCCATTTTTCCCCTTTAAAA------AGAAAAATGACTTCTATAAGTAGGAGTTCAGATACCAG--------A
CACTTTCTGACAGCATCATATCTGCTCGTGGCTTTCTTTAATCC-AAATTAAAGGAACAGA------------------A
GTACAGTGTTTAGGAGAAATA-AGAAAGATACCAAAGCAGCTTTAAACTTTCCCTACTAAATTACTGCATAGTAT--GAC
TTTTTACTAGATACAGCCT-TTCTAGGAGCTAATATTCTATTCATGTTTGGGTGTGGAGCAGAGAACCACATACATGTTT
ACTGTTGCTATTCAAGTTTCCTGATTAAGTGTTCTAATTCCCAGTGTTACATCTGAGAGAA-------------------
----
>Tsc
TGTTAATGACAACACCACGGGCAGTGC---CTGGAGGAGAGAGA-------------------------CAAGCTGCAAT
ACAGTACTTGCTGAGGCCAGACTCAAGAGAACTTTCAGCT----CCAGCAGGCTGGAAGAAGCCATTCCAA---------
-------GTCTCTGTATGGGTGTTTGACTGTGGAGTGAGAAGTGTGAGGCCATCAGACCACAATGCCCCTTTTTGTCCCT
ATCCACATCAAGGGC---------TCTACCTATGGATTGCTAACC-----TGTACTTCAGTATCAGGGTGCCTAGGATCT
GGAATCAACTTCCAAGAGAAGTGGTGCTGGCTCTTACCCTGGGG--------------GTCTTTAAGAAGAGACTAGATG
AACACCTTGCCGGGGCTGTTTGACCTCCATACTCTTTCCTCCCACGGCAGGGGGTCGGACTTGATGATCTGCTCACGTCC
CTTATGACCCTACCAACTACGAAACTATAACC---CAGCCCCAGTGAAGGGATTTCTACAGGGGTAGGGCTGCCCATCAC
CAGGGATCCTATTTTTCCTGTTTAAAA------AGAAAAATGACTTCCATAAGTAGGACTTCAGATCCCAG--------A
CACTTTCTGACAGCCACGAGCAGATATGATGCTTTCTTTAATCC-AAATTAAAGGAACAGA------------------A
GTACATCATTTAGGAGAAATA-AGAAAGATACCAGAGCGGCTTTAAACGTTCCCTACTAAATTACTGCATAGTAT--GAC
TTTTTACTAGATACAGCCT-TTCTAGGAGCTAATATCCTATTCTTGTTTGGGTGTGGAGCAGAGAACCACATACATGTTT
ACTGTTGCTATTCAAGTTTCCTGATTAAGTGTTCTAATTCCCAGTGTTACATTTGAGAGAATGTGAACATCACATAC---
CTTCCACACA
>Gga_scaffold14435
TGTTAATGACAACACCACGGGCAGTGC---CTGGAGGAGAGAGA-------------------------CAAGCTGCAAT
ACAGTACTTGCTGAGGCCAGACTCAAGAGAACTTTCAGCT----CCAGCAGGCTGGAAGAATCCATTCCAA---------
-------GTCTCTGTATGGGTGTTTGACTGTGGAGTGAGAAGTGTGAGGCCATCAGACCACAATTCCCGTTTTTGTCCCT
ATCCACATCAAGGGC---------TCTACCTATGGATTGCTAACC-----TGTACTTCAGTGTCAGGGTGCCTAGGATCT
GGAATCAACTTCCAAGAGAAGTGGTGCTGGCTCTTACCCTGGGG--------------GTCTTTAAGAAGAGACTAGATG
AACACCTTGCCGGGGTTGTTTGACCTCCATACTCTTTCCTCCCACGGCAGGGGGTCGGACTTGATGATCTGCTCACGTCC
CTTATGACCCTACCAACTACGAAACTATAACC---CAGCCCCAGTGAAGGGATTTCTACAGGGGTAGGGCTGTCCATCAC
CAGGGATCCTATTTTTCCTGTTTAAAA------AGAAAAATGACTTCCATAAGTAGGACTTCAGATCCCAG--------A
CACTTTCTGACAGCCACGAGCAGATATGATGCTTTCTTTAATCC-AAATTAAAGGAACAGA------------------A
GTACATCATTTAGGAGAAATA-AGAAAGATACCAGAGCAGCTTTAAACTTTCCCTACTAAATTACTGCATAGTAT--GAC
TTTTTACTAGATACAGCCT-TTCTAGGAGCTAATATCCTACTCTTGTTTGGGTGTGGAGCAGAGAACCACATACATGTTT
ACTGTTGCTATTCAAGTTTCCTGATTAAGTGTTCTAATTCCCAGTGTTACATTTGAGAGAATGTGAACATCACATAC---
CTTCCACACA
>Cya
TGTTAATGACAACACCACGGGCAGTGC---CTGGAGGAGAGAGA-------------------------CAAGCTGCAAT
ACAGTACTTGCTGAGGCCAGACTCAAGAGAACTGCCAGCT----CCAGCAGG----------------------------
-----------CTGTGTGTGTGTTTGACTGTGGAGTGAGAAGTGTGAGGCTGTCAGACCACAATGCCCCTTTTTGTCCCT
GTCCACATCAAGGGC---------TCTGCCTATGGATTGCTAACC-----------------------------------
----

-----------------------------------CAGCCCCAGTGA------------AGGGATAGGGTTGCCTACCAC
CAGGGATCCTATTTTTCCTGTTTAAAA------AGAAAAATGACTTCCATAAGTAGGACTCCAGATCCCAG--------A
CACTTTCTGACAGCCATGAACAGATAGGATGCTTTCTTTCATTCGAAATTAAGAGAACAGA------------------A
GTACAGCATTTAGAAGAAACA-AGAAAGATACCAAAGCAGCTTTAAACTTTCCCTACTAAATTACTGTGTAGTAT--GA-
TTTTTACTAGATACAGCCT-TTCTAGGAGCTAATATTCTATTCCTGTTTGGGTGTTGAGCAGAGAACCACATACATGTTT
ACTGTCGCTATTCAAGTTTCCTGATTAAGTGTTCTAACCCCCAGTGTCACACTTGAGAAACGGTGAACATCACATAC---
CTTCCACACA
>Asi_533262059
TGTTAATGACAACACCACGGGCAGTGC---CTGGAGGAGAGAGA-------------------------CAAGCTGCAAT
ACAGTACTTGCTGAGGCCAGACTC---AGAACTTTCAGCT----CCAGCAGGCTGGAAGGAGCCGTTCCAA---------
-------GCCTCTGTATGTGTGTTTGACTGTGGAGTGAGAAGTGTGAGGCTATCAGACCACAATGCCCTTTTTTGTCCCT
GTCCACATCAAGGGC---------TCTGCCTATGGATTGCTAACC-----------------------------------
----

```
--------------------------------------------------------------------------------
-----------------------------------CAGCCCCAGTGA------------AGGGATAGGGTTGCCTATCAC
CAGGGATCCTATTTTTCCTGTTTAAAA------AGAAAAATGACTTCCATAAGTAGGAGTCCAGATCCCAG--------A
CACTTTCTGACAGCCATGAGCAGATAGGATGCTTTCTTTCATTC-AAATTAAGGGAACAGA------------------A
GTACAGCATTTAGGAGAAACA-AGAAANNNNNNNNNNNNNNNNNNNNNNNTCCCTACTAAATTACTACGTAGTAT--GAC
TTTTTACTAGATACAGCCT-TTCTAGGAGCTAATATCCTATTCTTGTTTGGGTGTGGAGCAGAGAACCACATACATGTTT
ACTGTTGCTATTTAAGTTTCCTGATTAAGTGTTCTAATCCCCAGTGTTACATTTGAGAAACTGTGAACATCACATAC---
CTTCCACACA
>Ami_scaffold-2098
TGTTAATGACAACACCACGGGCAGTGC---CTGGAGGAGAGAGA-------------------------CAAGCTGCAAT
AAAGTACTTGCTGAGGCCAGACTC---AGAACTTTCAGCT----CCAGCAGGCTGGAAGGAGCCGTTCCAA---------
-------GCCTCTGTATGTGTGTTTGACTGTGGAGTGAGAAGTGTGAGGCTATCAGACCACAATTCCCTTTTTTGTCCCT
GTCCACATCAAGGGC---------TCTGCCTATGGATTGCTAACC-----------------------------------
--------------------------------------------------------------------------------
--------------------------------------------------------------------------------
-----------------------------------CAGCCCCAGTGA------------AGGGATAGGGTTGCCTATCGC
CAGGGATCCTATTTTTCCTGTTTAAAA------AGAAAAATGACTTCCATAAGTAGGACTCCAGATCCCAG--------A
CACTTTCTGACAGCCATGAGCAGATAGGATGCTTTCTTTCATTC-AAATTAAGGGAACAGA------------------A
GTACAGCATTTAGGAGAAACA-AGAAAGATACCGAAGCAGCTTTAAACTTTCCCTACTAAATTACTACGTAGTAT--GAC
TTTTTACTAGATACAGCCT-TTCTAGGAGCTAATATCCTATTCTTGTTTGGGTGTGGAGCAGAGAACCACCTACATGTTT
ACTGTTGCTATTCAAGTTTCCTGATTAAGTGTTCTAATCCCCAGTGTTACATTTGAGAAACTGTGAACATGACATAC---
CTTCCACACA
>Gallus_GB
TGCTAATAACAATGCCACGGGCAGTGC---CTAGAAGAGAAAGAGGCTTTAGAAAAGAAGAACATCCCAGAAGACACAAA
ACTGAGCTCGTTTAGTCTG--CCAAGCAGCTTTGTAAGTTTGGCCCAATATAATGCAGGTAGACATCCCAATGGGCCAAG
GTCTCCCACCACAGCTTTTATGCTCTCCAACAGGATGGAGGTATCTGGAGATGAGGGCTACACAGTCTTCCTGTACAACT
AGCCATACTGCATGCCCTTTCCCTTCTCCCAGAGAATCAAGAATCCA------------------------CTTGAATCA
AGTGCCAGCTGCATAAAAAATTAAAGAAGGAAGCAGTCTTCAAGTAGACTAAGCCTCTGCTTTAAAGGAGGAAAGAGG--
----------------------------ACACTTTCTCTTCCCAAGGCATGGAAAAAAATGCTCTGAGCTGTACATAA--
--------------------------------------------------------------------------------
-AGAGGACCTACTGTTGCAGTTCCACATATGGCAGAAGTTAGCTGGAGATATCTGAAACTTCTGGTTCCTGCAGCAGCTA
CATCATTTGCTAAACCTCTGCTTAGCCCTCTCATTATGGTATCTGCAATAAAGCACCCATGCCTGAAGACACATCACACA
GCTCCTCTTCTGGGAGAAATATGGTAAGACAATGCTGTGGAGTCATCTATGCCC---------ACTGTACAGTACTGGGG
TTGGAATGAGCAAAAGCCTGTGCTGGAAGCTACAAGCCTGCTGTTGTAGGAGTGGGTTACAAACAAATATGT---TGTGT
GTATTTGGCATTTGCATTGCTTTCCCACAGAGTTAATTCCCTCATGTTAATACTTGAACACCAAGACAGACACGTAC---
CTTCCACACA
```

GC09

```
>CR1-20
GTACGTCAGGGGTGAGCACCAAGATCTAGGGGAGCAACTCTTCAGGAAGGCACCTCAAGGGAGGACGAGGACCAATGGGC
ATAAGCTAATAGAGGGTAAATTCAGGCTGGACATAAGGAAAAACTTCT-----------------------------TCT
CTGTAAGGGTC-ACCAGAATCTGGAACACACTCCCAGCAGAGGTGGTGCAGTCACCCTCCTTGGAGGTGTTCAAGATGAG
GCTGGACAAGCACCTTGTTGAGCTCGTCTGAGCCCAATAA-CTTCCTGCCCATGGCAGGGGACTGGACTTGATGATCT-T
ACAGGTCCCTTCC------GGTCCTATGATTCTAT---------------------------------------------
------------------------
>Cpo_877_125850-127400_CR1_2.cl2_Am.Cp
TAGCTAATCACTTTTTAGTAGAGTCTGTTCTC------------------TGGAATAA----CTTCTTAAACATAGAAAT
TACCATTTCTGTT--GCAGTGTGCAATTATGGCTGTAATAAAACCTGCTTATCTTCATCTCCCTCTTTTTG------TCT
CTGTAGTTATTGGTAAATAACTGGAACCTACTTCCAAGAGAAGTGGTGCTGGCTCCTACCCTTGGGGTCTTTAAGAGGAG
GCTAGATGTACACCTTGCTGGGGTCGTTTGACCCCAGTACTCCTTCCTGCCATGGCAGGGGGTTGGTCTAGATGATCTAC
TCAGTTCCCTTCC-------GACCCTAGAAACTATGAAA-CCA--------AATAAAAACAGACATATATTGCTTCATTT
--TGTTTTTTCCTTAGATATCTTT
>Ote
GAGATATTCTCTTTTTAGAGGAGTGTGTTTTC------------------TGGGATATATTTTTTT----ACACAGAAAT
TACCATTTCTGTT--GCAGTGTGCAATTATGGATGTAATAAAACCTGCTTATCTTCGTCTCCCTCTTTTTG------TCT
CTGTAGTTATTTGTAAATAACTGGAACTTACTTCCAAGAGAAGTGGTGCTGGCTCCTACCCTCGGGGTCTTTAAGAGGAG
GCTAGACGTACACCTTGCTGGGGTCGTGTGACCCCAGTACTCCTTCCTGCCATGGCAGGGGGTTGGTCTAGATGATCTAC
TCAGTTCCTTTCC------TGACCCTAGAAACTATGAAA-CCA--------AATAAAAACAGACATATATGCT-------
------------------------
>Mca
TAGCTAATCACTTTTTAGTAGAGCCTGTTCTC------------------TGGAATAA----CTTCTTAAACATAGAAAT
TACCATTTCTGTT--GCAGTGTGCAATTATGGATGTAATAAACCCTGCTTATCTTCATCTCCCTCTTTTTG------TCT
CTGTAGTTATTCGTAAATAACTGGAACTTACTTCCAAGAGAAGTGGTGCTGGCTCCTACCCTCGGGGTCTTTAAGAGGAG
GCTAGATGTACACCTTGCTGGGGTCGTGTGACCCCAGTACTCCTTCCTGCCATGGCAGGGGGTTGGTCTAGATGATCTAC
TCAGTTCCTTTCC-------GACCCTAGAAACTATGAAA-CCA--------AATAAAAACAGACATATATTGCTTCATTT
--TGTTTTTTCCTTAGATATCTTT
>Tsc
TAGCTAATCACTTTTTAGTAGAGTTTGTTCTC------------------TGGAATAACTTCCTTCTTAAACATAGAAAT
TACCATTTCTATT--ACAGTGTGCAATTATGGATGTAATAAAACCTGCTTATCTTCATCTCCCTCTTTTTG------TCT
CTTTAGTTATTGGTAAATAACTGGAACCTACTTCCAAGAGAAGTGGTGCTGGCTCCTACCCTCGGGGTCTTTAAGAGGAG
GCTAGATGTACACCTTGCTGGGGTTGTTTGACCCCAGTACTCTTTCCTGCCATGGCAGGGGGTCAGTCTAGATGATCTAC
TCGGGTCCCTTCC-------GACTGTAGAAACTATGAAA-CCATGAAACCAAATCAAAACAAAGCAATATATGTCTGTTT
--T-TTTTTTCCTTAGATATCTTT
>Gga_scaffold7517 orig_len=166215
TAGCTAATCACTTTTTAGTAGAGTCTGTTCTC------------------TGGAATAACTTCCTTCTTAAACATAGAAAT
TACCATTTCTATT--ACAGTGTGCAATTATGGATGTAATAAAACCTGCTTATCTTCATCTCCCTCTTTTTG------TCT
CTTTAGTTATTGGTAAATAACTGGAACCTACTTCCAAGAGAAGTGGTGCTGGCTCCTACCCTTGGGGTCTTTAAGAGGAG
GCTAGATGTACACCTTGCTGGGGTCGTTTGACCCCAGTACTCCTTCCTGCCATGGCAGGGGGTCAGTCTAGATGATCTAC
```

```
TTGGGTCCCTTCT-------GACTGTAGAAACTATGAAA-CCATGAAACCAAATCAAAACAAAGCAATATATGTCTGTTT
--T-TTTTTTCCTTAGATATCTTT
>Cya
---------------------AGTCGGTTCTC------------------TGGAATAACTTCCTTCTTAAGCATAGAAAT
TACCATTTCAGTTCGGTAGTGTGCAATTATGGATGTAATGAAACCTGCTTATCTTTGTCTCCCTCTTTCTG---------
--------------------------------------------------------------------------------
--------------------------------------------------------------------------------
----------TCT------CTTTAGTTAAATAAAATAAATTCA--------AATAAAAACAGGCATATATTGCTTTGTT-
----TTTTTCCCTTAGATATCTTT
>Asi_533262060
TAGCTAATCCCTTTTTAGTAGAGTCTGTTCTC------------------TGGAATAACTTCCTTCTTAAGCATAGAAAT
TACCATTTCAGTTTGGCAGCGTGCAATTATGGATGTAATGAAACCTGCTTATCTTCGTCTCCCTCTTTCTG---------
--------------------------------------------------------------------------------
--------------------------------------------------------------------------------
-------------------CCTCTTTAGTTATTGGTAAA-TCA--------AATAAAACAGACATATTGCTTTGTTTTT-
------TTTTCTGTAGATATCTTT
>Ami_scaffold-3783 orig_len=1520965
TAGCTAATCCCTTTTTAGTAGAGTCTGTTCTC------------------TGGAATAACTTCCTTCTTAAGCATAGAAAT
TACCATTTCAGTTCGGCAGTGTGCAATTATGGATGTAATGAAACCTGCTTATCTTTGTCTCCCTCTTTCTG---------
--------------------------------------------------------------------------------
--------------------------------------------------------------------------------
-------------------TGTCTTTAGTTATTGGTAAA-TCA--------AATAAAAANAGACATGTATTGCTTTGTT-
----TTTTTTCCTTAGATATCTTT
>Gallus_GB
AAAACTGATGAAATTTACAGTAGTGTGCTTCTAAGGCACAGCTGTGTTGGTTGAGCAGCTTCACTTCTGAGCA-------
-AGTGCTTCACTTCAGCTTTGCTTGAACACTGCTGAATTAAACTGGGTTTACATTGAATTTCTCAACTAAT---------
--------------------------------------------------------------------------------
----------------------------------------------------------TCACTGTATGCAGAACTTCCTG
TATTTTCTTTGTC------TTATACTTGGAAATACAATGGAAA--------AGGAAATGCATGATTGTGTGGGTTTTTTT
GCTCCATTTCTTTCAGACATGTTT
```

GC12

```
>LTR2
--------------------------------------------------------------------------------
--------------------------------------------------------------------------------
--------------------------------------------------------------------------------
--------------------------------------------------------------------------------
--------------------------------------------------------------------------------
--------------------------------------------------------------------------------
-------------------------------------------TGTGGAAATAAGTTTAACCAGGCCTCT----TA----
---------------------CTTGACTAAGAGGAGAGACAAAGAGAGAGAAAGAGAAGAGAAAAAGATGCTGATGCTAT
GTGTTTC-AAAATGTTCTAAGATAAGAAAAAACCTTCATGCCTTGTTTTGTCTTGATATATTAAGCTCTGTGTGCAGATG
TTAACCTCCTCCTTTCCCCCGAACCGGAAGCTGATAACTGTTCCCA-AAACACAGTGGTTTTT---TTTCTG-AATAAGT
AAACTGCTTACGTGTGACAAACGAATGATCAACATCTTTCTGTTTCTCTCAAGGTTGCTTTGTCTGAACCCTGCATCTTT
TCTCGCTGTCTAAAGTATAAATACGCTTGTAAACTTGTAGAGGTC-AGAGTTCCTTTGA---------------------
----------------------------GAACCCTCCTTGTGATCACTTGTATAGCTTTAAATA-----AACCTAGATGG
CTCTGC----------------------CAATTCTAATACAACCACAACGCGAAGTTTGATTTCTTCCACA---------
--------------------------------------------------------------------------------
--------------------------------------------------------------------------------
--------------------------------------------------------------------------------
------------------------------------------------
>Exons_rc
CTTCCGTTTCTCCCCCTGGCCACACTGCAGAAGGCAAGCCATCTGTATCAAACC--------------------------
--------------------------------------------------------------------------------
--------------------------------------------------------------------------------
--------------------------------------------------------------------------------
--------------------------------------------------------------------------------
--------------------------------------------------------------------------------
--------------------------------------------------------------------------------
--------------------------------------------------------------------------------
--------------------------------------------------------------------------------
--------------------------------------------------------------------------------
--------------------------------------------------------------------------------
--------------------------------------------------------------------------------
--------------------------------------------------------------------------------
--------------------------------------------------------------------------------
--------------------------------------------------------------------------------
--------------------------------------------------------------------------------
--------------------------------------------------------------------------------
------------------------------------------CTAGCT
>Cpo_1426_180550-182100
CTTCTGTTTCTCCCCCTGGCCACACTGCAGAAGGCAAGCCATCTGTATCAAACC---CTGAAGGATTAAGTCAAACAGGA
AGAAAAATCCTTATCTGTTTATACTGTAGCAC-AAATAACTAGTATACCTGGACAACAGCTG------CACTGTATTTTT
GGTAATATCTTAAATCTAGCTGGATGTCAATG-----GTGTCAAAACAAAGCTTGCTGTCAGCTTGCTGTCAGTGTAAAT
AAGTTCTACCAAACCATGT-------------------------------------------------------------
AACAACATTGC-TTATCTGTAACTGAATT---------------------------------------------------
-----------------------------AACTGTGATTTTTATGCTCTA------------TAATAAAGAAAGATCCCT
TGACAAATTTTGACTCTGC----------ATTT---GTTG---TGTGGTAGTAGATTTGGTCGGGCTTTTGGCCTG----
```

```
---------------------CTTGACTAGGAAAACAGACAGAGACCACAAG----------------TGCTGATACTGC
TAACTTCCATTAAATGTTCTGCGATAAAGAATCCTGCATGCCTTGTTTTGTCTTGATATATTACCCTGTGCATGGAT---
--------------------GTTAAGTTTAT--TTTTCTCTGTGAGAAACCAGAAATTGATAACTATTTCTATAAGCACA
ACATTTCACTCTTCTTTAAGCCTGTATGTCTGCGTGTGTGTTACACCAGTGATTTACTGTCCAAGGTCGTACTTTTTTAA
TCTCTCTGTCTTAAGTATAAATATGCTTGTAAACGTATACAGTTCGAGGATTCCTTTGAAACAGACAAGTGGGCGATTCC
CCTTGCTG----------------TATGGCATTCTCCCTGTGATCAC-AGTATAGCTCTAAATA----AAGAAAAGCTGG
CTCTGT----------------------CAGTTCTGATTCAACCACAACGCGAAGTTTGATTTCTTCCACA---GTTA--
-GGTTAGGTTCCCTGTGGTTATTGCTG-GCTGTTCTGATGGCAGC------------AATTTTAATTGATTGATTTGG--
---------------------------------------------------------------CTTAAGCCTAAACAATA
TCATCTAGCACT--------------------------------GTTTTAACACTGTACAATAAA---------------
-------------AGCATTTAAGACTTAAGACAACATAC---CTAGCT
>Ote
CTTCTGTTTCTCCCCCTGGCCACACTGCAGAAGGCAAGCCATCTGTATCAAACC---CTGAAGGATTAAGTCAAACAGGA
AGAAAAATCCTTATCTGTTTATACTGTAGCACAAAATAACTAGTATACCTGGACAACAGCTG------CACTGTATTTTT
GGTAATATCTTATATCTAGCTGGATGTCAATG-----GTGCCAAAACAAAGCTTGCTGTCAGCTTGCTGTCAGTGTAAAT
AAGTTTTACCAAACCATGT-------------------------------------------------------------
AACAACATTGC-TTATCTGTAACTGAATT---------------------------------------------------
-----------------------------AACTGTGATTTTTATGCTCTA------------TAATAAAGAAAGATCCCT
TGACAAATTTTGACTGTGC----------ATTT---GTTG---TGTGGTAGTAGATTGGGTCGGGCTTCTGGCCTG----
---------------------CTTGACTAGGAAAACAGGCAGAGACCACAAG----------------TGCTGATACTGC
TAACTTCCGTTAAATGTTCTGCGATAAAGAATCCTGCATGCCTTGTTTTGTCTTGATATATTACCCTGTGCATGGAT---
--------------------GTTAAGTTTAT--TTTTCTCTCTGAGAAACCAGAAATTGATAACTATTTCTATAAGCACA
ACATTTCACTCTTCTTTAAGCCTGTATGTCTGCGTGTGTGTTACACCAGTGATTTACTGTCCAAGGTTGTACTTTTTTAA
--TCTCTGTCTTAAGTATAAATATGCTTGTAAACGTATAGAGTTCGAGGATTCCTTTGAAACAGACAAGTGTGTGATTCC
CCTTGCTG----------------TATGGCATTCTCCCTGT-GATCACAGTATAGCTCTAAATAAAGCAAGAAAAGCAGG
CTCTGT----------------------CAGTTCTGATTCAACCACAACGCGAAGTTTGATTTCTTCCACA---GTTG--
-GGTTAGGTTCCCTGTGGTTATTGCTG-GCTGGTCTGATGGTAGC------------AATTTTAATTGATTGATTTGG--
---------------------------------------------------------------TTTAAGCCT-AACAATA
TCATCTAGCACT--------------------------------GTTTTAACATTGTACAATAAA---------------
-------------AGCATTTAAGACTTAAGACAACATAC---CTAGCT
>Mca
CTTCTGTTTCTCCCCCTGGCCACACTGCAGAAGGCAAGCCATCTGTATCAAACC---CTGAAGGATTAAGTCAAACAGGA
AGAAAAATCCTTATCTGTTTATACTGTAGCAC-AAATAACTAGTATACCTGGACAACAGCTG------CACTGTATTTTT
GGTAATATCTTAAATCTAGCTGGATGTCAATG-----GTGTCAAAACAA-----------AGCTTGCTGTCAGTGTAAAT
AAGTTCTACCAAACCATGT-------------------------------------------------------------
AACAACATTGC-TTATCTGTAACTGAATT---------------------------------------------------
-----------------------------AACTGTGATTTTTATGCTCTA------------TAATAAAGAAAGATCCCT
TGACAAATTTTGACTGTGC----------ATTT---GTTG---TGTGGTAGTAGATTTGGTCGGGCTTCTGGCCTG----
---------------------CTTGACTAGGAAAACAGACAGAGACCACAAG----------------TGCTGATACTGC
TAACTTCCATTAAATGTTCTGCGATAAAGAATCCTGCATGCCTTGTTTTGTCTTGATATATTACCCTGTGCATGGAT---
--------------------GTTAAGTTTAT--TTT----TCTAAGAAACCAGAAATTGATAACTATTTCTATAAGCACA
ACATTTCACTCTTCTTTAAGCCTGTATGTCTGCATGTGTGTTACACCAGTGATTTACTGTCCAAGGTTGTACTTTTTTAA
--TCTCTGTCTTAAGTATAAATATGCTTGTAAACGTATAGAGTTCAAGGATTCCTTTGAAACAGACAAGTGGGTGATTCC
CCTTGCTG----------------TATGGCATTCTCCCTGTGGATCACAGTATAGCTCTAAATA----AAGAAAAGCTGG
CTCTGT----------------------CAGTTCTGATTCAACCACAACGCGAAGTTTGATTTCTTCCACA---GTTG--
-GGTTAGGTTCCCTGTGGTTATTGCTG-GCTGTTCTGATGGTAGC------------AATTTTAATGGATTGATTTGG--
---------------------------------------------------------------CTTAAGCCTAAACAATA
TCATCTAGCACT--------------------------------GTTTTAACATTGTACAATAAA---------------
-------------AGCATTTAAGACTTAAGACAACATAC---CTAGCT
>Tsc
CTTCCGTTTCTCCCCCTGGCCACACTGCAGAAGGCAAGCCATCTGTATCAAACC---CTGAAGGATTAAGTCAAACAGGA
AGAAAAATCCTTATTTGTTTATACTGTAGCAC-AAATAACTAGTATACCTGGACAACAGCTG------CACTGTATTTTT
GGTAATATCTTAAATCTAGCTGGATGTCAATG-----GTGTCAAAACAA-----------AGCTTGCTGTCAGTGTAAAT
AAGTTCTACCAAACCATGT-------------------------------------------------------------
AACAACATTGC-TTATCTGTAACTGAATC---------------------------------------------------
-----------------------------AACTGTGATTTTTATGCTCCA------------TAATAAAGAAAGATCCCT
TGACAAGTTTTGACCGTGC----------ATTT---GTTG---TGTGGTGGTAGATTTGGTCAGGCTTCTGGCCTG----
---------------------CTTGACTAGGAAAACAGACAGAGACCAGAAG----------------TGCTGATACTGC
TTATTTCAGTTAAATGTTCTGAGATAAAGAATCCTGCATGCCTTGTTTTGTCTTGATATGTTACCCTGTGCATGGAT---
--------------------GTTAAGTTTAT--TTTTCTCTCTAAGAAACCAGAAATTGATAACTATTTCTATAAGCACA
ATGTTTCACTCTTCTTTAAGCCTGTATGTCTGTGTGTGTGTTAGACCACTGATTTACTGTCCAAGGTCGTATTTTTCTCT
-CTCTCTGTCTTAAGTGTAAATATGCTTGTAAACTTATAGAGTTTGAGGATTCCTTTGAAACAGACAAGTGGGTGATTCC
CCTTGCTG----------------TATGGAATTCTCTCTGT-GATCACAGTATAGCTTTAAATA----AAGAAAAGCTGG
CTCTGT----------------------CAGTTCTGATTCAACCACAATGCGAAGTTTGATTTCTTCCACA---GTTG--
-GGTTAAGTTCCCTGTGGTCATTGCTG-GCTGTTCTGATGGTAGC------------AATTTTAACTGATTGATTTGG--
---------------------------------------------------------------CTTAAGCCTAAACAACA
TCATCTAGCACC--------------------------------GTTTTAACACTGTACAATGAA---------------
-------------AGTATTTAAGACTTAAGACAACATAC---CTAGCT
>Gga_scaffold24882
CTTCCGTTTCTCCCCCTGGCCACACTGCAGAAGGCAAGCCATCTGTATCAAACC---CTGAAGGATTAAGTCAAACAGGA
AGAAAAATCCTTATTTGTTTATACTGTAGCAC-AAATAACTAGTACACCTGGACAACAGCTG------CACTGTATTTTT
GGTAATATCTTAAATCTAGCTGGATGTCAATG-----GTGTCAAAACAA-----------AGCTTGCTGTCAGCGTAAAT
AAGTTCTAGCAAACCATGT-------------------------------------------------------------
AACAACATTGT-TTATCTGTAACTGAATC---------------------------------------------------
-----------------------------AACTGTGATTTTTATGCTCCA------------TAATAAAGAAAGATCCCT
TGACAAGTTTTGACCGTGC----------ATTT---GTTG---TGTGGTAGTAGATTTGGTCAGGCTTCTGGCCTG----
---------------------CTTGACTAGGAAAACAGACAGAGACCAGAAG----------------TGCTGATACTGC
TTATTTCAGTTAAATGTTCTGAGATAAAGAATCCTGCATGCCTTGTTTTGTCCTGATATATTACCCTGTGCATGGAT---
```

--------------------GTTAAGTTTAT--TTTTCTCTCTAAGAAACCAGAAATTGATAACTATTTCTATAAGCACA
ATGTTTCACTCTTCTTTAAGCCTGTATGTCTGTGTGTGTGTTAGACCACTGATTTACTGTCCAAGGTCGTATTTTTCTCT
-CTCTCTGTCTTAAGTGTAAATATGCTTGTAAACTTATAGAGTTTGAGGATTCCTTTGAAACAGACAAGTGGGCGATTCC
CCTTGCTG----------------TATGGAATTCTCCCTGT-GATCACAGTATAGCTTTAAATA----AAGAAAAGCTGG
CTCTGT----------------------CAGTTCTGATTCAACCACAATGGGAAGTTTGATTTCTTCCACA---GTTG--
-GGTTAGGTTCCCTGTGGTCATTGCTG-GCTGTTCTGATGGTAGC------------AATTTTAACTGATTGATTTGG--
---CTTAAGCCTAAACAACA
TCATCTAGCACC--------------------------------GTTTTAACACTGTACAATGAA---------------
-------------AGTATTTAAGACTTAAGACAACATAC---CTAGCT
>Cya
CTTCCGTTTCTCCCCCTGGCCACACTGCAGAAGGCAAGCCATCTGTATCAAACC---CTGAAGAATGAAGTCAAACAGGA
AGAAAAATCCTTATTTGTTTATACGGTAGCAC-AAATAACTAGTATACCTGGACAACAGCTG------CACTGTATTTTT
GGTAATATCTTAAATCTAGCTGGATGTCAATG-----GTATCAAAACAA-----------AGCTTGCTTTCAGTGTAAAT
ACGTTCTACCAAACCATGT---
AACAACATTGC-TTATCTGTAACCGGATC---
-----------------------------AACTGTAATTTTTATGCTCTA------------TAATAAAGAAAGATCCCT
TAACAAATTTTGACTGTGC----------ATTT---

--GTTG--
-GGTTAGGTTTCCTGTGGTTGTTGTTG-GCTGTTCTGTTGGTAGC------------AATTTTAAATGATNGATTTGG--
---CTTAAGCCTA---AATA
TCACCTAGCACT--------------------------------GTTTTAACATTGTACAATAAA---------------
-------------AGTATTTAAGACTTAAGACAACATAC---CTAGCT
>Asi_533324869
CTTCCGTTTCTCCCCCTGGCCACACTGCAGAAGGCAAGCCATCTGTATCAAACC---CTGAAGAATGAAGTCAAACAGGA
AGAAAAATCCTTATTTGTTTATACGGTAGCAC-AAATAACTAGTATACCTGGACAACAGCTG------CACTGTATTTTT
GGTAATATCTTAAATCTAGCTGGATGTCAATG-----GTGTCAAAACAA-----------TGCTTGCTGTCAGTGTAAAT
AAGTTCTACCAAACCATGT---
AACAACATTGC-TCATCTGTAACCGGATC---
-----------------------------AACTGTAATTTTTATGCTCTA------------TAATAAAGAAAGATCCCT
TGACAAATTTTGACTGTGC----------ATTT---

--GTTG--
-GGTTAGGTTTCCTGTGGTCATTGCTG-GCTGTTCTGTTGGTAGC------------AATTTTAATTGATTGATTTGG--
---CTTAAGCCTA---AATA
TCACCTAGCACT--------------------------------GTTTTAACATTGTACAATAAA---------------
-------------AGTATTTAAGACTTAAGACAACATAC---CTAGCT
>Ami_scaffold-8291
CTTCCGTTTCTCCCCCTGGCCACACTGCAGAAGGCAAGCCATCTGTATCAAACC---CTGAAGAATGAAGTCAAACAGGA
AGAAAAATCCTTATTTGTTTATACGGTAGCAC-AAATAACTAGTATACCTGGACAACAGCTG------CACTGTATTTTT
GGTAATATCTTAAATCTAGCTGGATGTCAATG-----GTGTCAAAACAA-----------TGCTTGCTGTCAGTGTAAAT
AAGTTCTACCAAACCATGT---
AACAACATTGC-TTATCTGTAACTGGATC---
-----------------------------AACTGTAATTTTTATGCTCTA------------TAATAAAGAAAGATCCCT
GGACAAATTTTGACTGTGC----------ATTT---

--GTTG--
-GGTTAGGTTTCCTGTGGTCATTGCTG-GCTGTTCTGTTGGTAGC------------AATTTTAATTGATTGATTTGG--
---CTTAAGACTA---AATA
TCACCTAGCACT--------------------------------GTTTTAACATTGTACAATAAA---------------
-------------AGTATTTAAGACTTAAGACAACATAC---CTAGCT
>Gallus_GB
CTTCTGTTTCTCCCCCAGGCCATACTGCAGAAGGCAGACCATCTGTGTCAAAAC---CTGAAAAACAAATTAAAAC---A
AGGAAAGTCCTTGTTTGCCTCTACTGAAGGGC-AGTAAATTCTAACATATTGATACTTGATGGCATGTTATTATA-TCCT
GAGAACAGCCTTAAGTTGGACACATGGCAAAGTACAAGTTCTATAACAG----------------------TACGCAACC
AAGCCTTATCAAGCCATGTTCAATAGCTCCAACTTGCAGGAAAATGCATTTTTTACATTCCATTATAAAGTTTAATGCTG
AATGATTTTATTTCAATTCCACTTGTTTTGTTTTTGCCCAAAGTGACCTTCCTTTCATCTTCCAAAGTATTTTCAAGTTC
CCAGCTCTGCAATACATCGATTACCTCCGAACTACAACTTTTACTTTTTATTACCTCACTACTAAGCAGCAAGAATTGCT
TCATTAATTTATAGGATGCCTAAAAACTTTTTT---TTTC---GATCGTACTAAAAGCATCCATGCTACCTTCAGAACTA
CCATTCCTATGTTTCCTTGACTTTGACATACATAACAGATTGGAATTACAAT----------------TGGTAATTCTGC
AGAT----------------------GAAAAAGAGGCTAACTTTTTTCCCCTCCCATCCTCTCCCCTAAGAACTGTTCAC
CAGGTACACAAGATAATGTGTTTAGTGAGAT--CTTTCTTTCTGAAGA---AGGCTTTAAAAGACAGAGCAGCAAGAGCA
TAGCCAAAATTTGTCCAGAGCCTTTTGTTAGGGATTTG------------------------GTGGTCATGCTGCATTTG

```
TTTGCATATCTTTCTTACATTAAAATTTATACAAACAAGACATTCTAGAATCTTCTAAAATAA----------TTATTTG
CCTTATTATGATCTTTTACGGTATTGCAGGACAAGCTTTTTGGACTTTGTTTTGTTTTAAATTAAATTGTAAAGCGATAG
ATCAATGACAAAACACAAGGTCACCAAACACATATAATGCAAAGAAATAATCAAGACTGTAGTAGCTTGAT---GATG--
-ATTTGGTATCTTGAAAAAAATGGCTGTGCTGTACTTCAAGAAGCTCAGCAGTACTTAGTGTAGTTTGATCCATCTGACC
ACCCACTTAAACAAACCAGACACCTAAAACGCACGGAGCTCATCATCCCTTTTTGTTCTACTTCTTTGCCATAACTGGCA
TCAGCTAACAATTAACCAGAAATAGAACCTCTTTCACGTGGTTATTTTAAGCATTATGCTTTCAGTGGTAGGCTACTTCT
ATACTCATTCCACAACATCCAAACGGTGTAAGAACTTAC---CTAGCT
```

GG01

```
>CR1-7B
--------------------------------------------------------------------------------
---------------------------------------------------------GCTGGCAGAGGGTAGGTTTAGGT
TAGACATCAGGAGGCGCTATTTCACGGTCAGGGCGGCT------------AGGGTCTGGAACCAACTTCCAAGGGAGGTG
GTGCAGGCTCCTACCCTGGGGGTTTTTAAGAGGAGGTTAGATGAACACCTTGCTGGGGTCGTTTGACCCCAGTACTCTTT
CCTGCCATGGCAGGGGGTCGGACTAGATGATCTGCTCAGGTCCCTTCCGACCCTAGAAACTATGAAACT-----------
--ATGAAACTA---------------------------------------------------------------------
--------------------------------------------------------------------------------
--------------------------------------------------------------------------------
-------------------------
>Exons_rc
--------------------------------------------------------------------------------
--------------------------------------------------------------------------------
--------------------------------------------------------------------------------
--------------------------------------------------------------------------------
--------------------------------------------------------------------------------
--------------------------------------------------------------------------------
--------------------------------------------------------------------------------
-CTTGTCCAGATAGATCAAGTATGCGCTTATGGGATTCTTTGCCATCGAAGATTTCAAAGGTGTATTTCCCTTTATCCTT
TTCAGTAGGTTCACATATCTGCATC
>Cpo_caffold03690
AAATATTGTTATGCATTATTTGAAATGAAATGTAGCTTTTATCTGTGAGTAAATAGAAATTTCATT----------AATA
CCTATGTTAATATCCTTTTCTTTTAAAACCTTTTAAAATGTATGTTACACCATACTGAAATCTGAAATGACAGCTACATG
TATAGAACCAAGCTCAG----------CCCTAGTGAAA------------------------------------------
--------------------------------------------------------------------------------
------------------------------------------------------------------------ATGAAT--
-TTAAGAATAAGGAAGCAGGTGAAATTTTACAACT------TGGCTAAATTTGACCACTAGC-----TTTCTATTTTTCC
AATAATTCTTCCAGTTAAAGCAGTTTA-----TAAAAATCCACCGTGCATTATGACTACCATATATTCAATATTCTAC--
-CTTGTCCAGATAGATCAAGTATGCGCTTATGGGATTCTTTGCCATCAAAGATTTCAAAGGTGTATTTCCCTTTATCCTT
TTCAGTAGGTTCACATATCTGCATC
>Ote
AAATATTGTTATGCATTATTTGAAATGCAATGTAGCTTTTATCTGTGAGTAAATAGAAATTTCATT----------AATA
CCTATGTTAATATCCTTTTCTTTTAAAACCTTTTAAAATGTATGTTACACCATACTGAAATCTGAAATGACAGCTACATG
TATAGAACCAAGCTCAG----------CCCTAGTGAAA------------------------------------------
--------------------------------------------------------------------------------
------------------------------------------------------------------------ATGAAT--
-TTAAGAATAAGGAAGCAAGTAAAATTTTACAACT------TGGCTAAATTTGACCACTAG------TTTCTATTTTTCC
AATAACTCTTCCAGTTAAAGCAGTTTA-----TAAAAATCCACCGTGCATTATGACTACCATATATTCAATATTCTAC--
-CTTGTCCAGATAGATCAAGTATGCGCTTATGGGATTCTTTGCCATCAAAGATTTCAAAGGTGTATTTCCCTTTATCCTT
TTCAGTAGGTTCACATATCTGCATC
>Mca
AAATATTGTTATGCATTATTTGAAATGAAATGTAGCTTTTATCTGTGAGTAAATAGAAATTTCATT----------AATA
CCTATGTTAATATCCTTTTCTTTTAAAACCTTTTAAAATGTATGTTACACCATACTGAAATCTGAAATGACAGCTACATG
TATAGAACCAAGCTCAG----------CCCTAGTGAAA------------------------------------------
--------------------------------------------------------------------------------
------------------------------------------------------------------------ATGAAT--
-TTAAGAATAAGGAAGCAAGTAAAATTTTACAACT------TGGCTAAATTTGACCACTAGC-----TTTCTATTTTTCC
AATAATTCTTCCAGTTAAAGCAGTTTA-----TAAAAATCCACCGTGCATTATGACTACCATATATTCAATATTCTAC--
-CTTGTCCAGATAGATCAAGTATGCGCTTATGAGATTCTTTGCCATCAAAGATTTCAAAGGTGTATTTCCCTTTATCCTT
TTCAGTAGGTTCACATATCTGCATC
>Tsc
AAATATTGTTGTGCATTATTTGAAATGAAATGTAGCTTTTATCTGTGAGTAAATAGAAATTTCATT----------CATA
CCTATGTTAATATCCTTTTCTTTTAAAACCTTTTAAA----ATGTTACACCATACTGAAATCTGAAACGACACCTACGTG
TATAGAACCAAGCTCAG----------CCCTAGTGAAA---ATGAAC---AGGGTCTGGAACCAGCTTCCAAGAGAAGTC
GTGCAGGCTCCTACCCTGGGGGGTTTTAAGAGGAGGTTAGATGAACACCTTGCTGGGGTCGTTTGACCCCAGTACTATTT
CCTGCCATGGCAGGGGGTCGGACTAGATGATCTACTCAGGTGCCTTCTGACCCTAGAATCTATGAAACT---ATGAAT--
-TTAAGAATAAGGAAGCGAGTAAAATTTTACAACT------TGGCTGAATTTGACCACTAGC-----TTTCTATTTTTCC
AATAATTCTTCCAGTTAATGCAGTTTA-----TAAAAATCCACCATGCATTATGACTACTATATATTCAGTATTCTAC--
-CTTGTCCAGATAGATCAAGTATGCGCTTATGGGATTCTTTGCCATCAAAGATTTCAAAGGTGTATTTCCCTTTATCCTT
TTCAGTAGGTTCACATATCTGCATC
>Gga_scaffold287
AAATATTGTTGTGCATTATTTGAAATGAAATTTAGCTTTTATCTGTGAGTAAATAGAAATTTCATT----------CATA
CCTATGTTAATATCCTTTTCTTTTAAAACCTTTTAAA----ATGTTACACCATACTAAAATCTGAAACGACAGCTACATG
TATAGAACCAAGCTCAG----------CCCTAGTGAAA---ATGAAT---AGGGTCTGGAACCAGCTTCCAAGAGAAGTG
GTGCAGGCTCCTACCCTGGGGGGTTTTAAGAGGAGGTTAGATGAACACCTTGCTGGGGTCGTTTGACCCCAGTACTATTT
CCTGCCATGGCAGGGGGTCGGACTAGATGATCTACTCAGGTCCCTTCCGACCCTAGAAACTATGAAACT---ATGAAT--
-TTAAGAATAAGGAAGCGAGTAAAATTTTACAACT------TGGCTGAATTTGACCACTAGC-----TTTCTATTTTTCC
AATAATTCTTCCAGTTAATGCAGTTTA-----TAAAAATCCACCATGCATTATGACTACTATATATTCAATATTCTAC--
-CTTGTCCAGATAGATCAAGTATGCGCTTATGGGATTCTTTGCCATCAAAGATTTCAAAGGTGTATTTCCCTTTATCCTT
```

```
TTCAGTAGGTTCACATATCTGCATC
>Cya
AAATATTGTTGTGCATTATTTGAAATGAAATGTAGCATTTATCTGTGAGTAAATAGAAATTTCATT----------CATA
CCTATGTTAATATTCTTTTCTTTTAAAACCTTTTAAA----ATCTTATACCATACTGAAATCTGAAATGACAGCTACATG
TATGGAA-------CAG----------ACCTAGTGAAA------------------------------------------
--------------------------------------------------------------------------------
------------------------------------------------------------------------ATGAAT--
-TTAAGAAT--------AAGTACAACTTTACAAGT------TGGCTGAATTTGACCACTAGA-----TTTCTATTTTTCC
AATAACTCTTCCAGTTAATGCAGTTTA-----TAAAAATCCACCATGCATTATGACTACCATATATATAATATTCTAC--
-CTTGTCCAGATAGATCAAGTATGCGCTTATGGGATTCTTTGCCATCGAAGATTTCAAAGGTGTATTTCCCTTTATCCTT
TTCAGTAGGTTCACATATCTGCATC
>Asi_533314752
AAATATTGTTGTGCATTATTTAAAATTAAATGTAGCATTTATCTGTGAATAAATAGAAATTTCATT----------CATA
CCTATGTTAATATTCTTTTCTTTTAAAACCTTTTAAA----ATCTTACACCATACTGAAATCTGAAACGACAGCTACATG
TATGGAA-------CAG----------ACCTAGTGAAA------------------------------------------
--------------------------------------------------------------------------------
------------------------------------------------------------------------ATGAAT--
-TTAAGAAT--------AAATAAAATTTTACAGCT------TGGCTGAATTTAACCACTAGC-----TTTCTATTTTTCC
AATAATTCTTCCAGTTAATGCAGTTTA-----TAAAAATCCACCATGCATTATGACTACCATATATATAATATTCTAC--
-CTTGTCCAGATAGATCAAGTATGCGCTTATGGGATTCTTTGCCATCGAAGATTTCAAAGGTGTATTTCCCTTTATCCTT
TTCAGTAGGTTCACATATCTGCATC
>Ami_scaffold-1786
AAATATTGTTGTGCATTATTTGAAATTAAATGTAGCATTTATCTGTGAGTAAATAGAAATTTAATT----------CATA
CCTATGTTAATATTCTTTTCTTTTAAAACCTTTTAAA----ATCTTACACCATACTGAAATCTGAAACGACAGCTACATG
TATGGAA-------CAG----------ACCTAATGAAA------------------------------------------
--------------------------------------------------------------------------------
------------------------------------------------------------------------ATGAAT--
-TTAAGAAT--------AAATAAAATTTTACAACT------TGGCTGAATTTGACCACTAGC-----TTTCTATTTTTCC
AATAATTATTCCAGTTAATGCAGTTTA-----TAAAAATCCACCATGCATTATGACTACCATATATATAATATTCTAC--
-CTTGTCCAGATAGATCAAGTATGCGCTTATGGGATTCTTTGCCATCGAAGATTTCAAAGGTGTATTTCCCTTTATCCTT
TTCAGTAGGTTCACATATCTGCATC
>Gallus_GB
AAACAATTGAGAGAGTTGT------------------------TGTCCATGAACATTATTTGCATTTTGGGCTCAGTATA
AGCATTTAAATACATATCCTTACTAAAGCCTTTTTAA--------------AAACAACAGTGTTAAACAATAGCAGAAGG
GA--AAACCAAACCCTGAATCCAGTGTCCCTAGTGGTA------------------------------------------
--------------------------------------------------------------------------------
--------------------------------------------------------------------------------
------------------AAAACAGTTATGCAACTAAATTGCACCTAATTTTGACCACAGGCTATTTTTACTGCATTCCT
CTTCTTACTCCTGATTAATTCTGTTTAATCTGTGAAAAGTCTCCATATA----------GGTATGATCTTAAACATAC--
-CTTGCCCAGACAGATCAAGTGTGCGTTTGAAGGATTCCTTGTCACTAAAGATTTCAAAGGTGTAGTTCCCCTTATCCTT
TTCAGTGGGTTCAGTTATCTGCAGC
```

GG03

```
>CR1-5B
--------------------------------------------------------------------------------
--------------------------------------------------------------------------------
--------------------------------------------------------------------------------
--------------------------------------------------------------------------------
--------------------------------------------------------------------------------
--------------------------------------------------------------------------------
--------------------------------------------------------------------------------
-----------------------------------------------TCATAGAATCATAGAAGTAGGGTCGGAAGGGAC
CTTGTAGATCTTCAAGTCCGACCCCCTGCCTGGGCAGGAGAAAAAC--------------------TGGGCTCAAATGAC
TCCAGCCAGGTAGGCATCAAGCCTCCTCTTAAAGACCC-----------------------------------CCAGGGT
AGGAGCCAGCACCACTTCCCTTGGAAGTTGGTTCCAGATCCTAGCCGCCCTGACTGTG----------------------
----------------
>Exons
--------------------------------------------------------------------------------
--------------------------------------------------------------------------------
--------------------------------------------------------------------------------
--------------------------------------------------------------------------------
--------------------------------------------------------------------------------
--------------------------------------------------------------------------------
--------------------------------------------------------------------------------
--------------------------------------------------------------------------------
--------------------------------------------------------------------------------
--------------------------------------------------------------------------------
--------------------------------------------------------------------------------
-------AAACTGCGG
>Cpo_scaffold00159
GTACCTTAGGAACCTCTCTGGTGTCTGCAGGAGGAACCTAAATGATACAGAATCTCAGACCCAGGATAGGTGTAACATTT
CTCCTGGTATATGAAAGTAACAGACAGATGATGCAGACCCATAAATGGCGTAACTGCTACTCTGATGG-AGAATCACCTC
TGAAAGAACTGGGGAAAACTCTGCAAGTGTATGTTATGCTGATTTAGATTTTGACTGTTCACATCCATCTGGTGGAGGTA
GTTGAAGGAACAAAGACTCTTGAACTGTTCCTGTGCTGCCTAAGTGTCAAGAGTA--AGTCACCCACTGTGCACAGGTAC
TTCTGGATAACTGTATGTCTGTCCGTTCCCAATCTGAGTGCAATTTACATCAGAACAGAACCCTAGTATGTGTACACCAG
GTATACGTTCATCTGTCTAGCACCTAAATGTTAAGATCTGTATCTACTGACCAGAACTGGAGAGA-------GAACAGAG
AACCCACTTTCTTTTTCATAAGATGATGT----AGACATTTATTTTTTTAGTGGAGGGTAGCGGGAAGAAGGAGGGTAGA
AGCAGCAGCTGAGGTGAGAGAGGCTTTCAAGCACCTTCCTT---------------------------------------
```

--ATTTCAGTATGTAATTTAAT---AGA-----TCCCTCC
AACCTTACATGTGGCAGGAAA----------TAACAGCTCTTGGCATTCTTAATAGCTCAGCTCTTTCC--TTGTTCTGT
GCAG---AAGCTGCGG
>Tsc
GTACCTTAGGAACCTCTCTGGTGTCTGCAGGAGGAACCTAAATGATACAGAATCTCAGACCCAGGATAGGTGTAACATTT
CTCTTGGTGTATGAAAGTAACAGACAGATGATGCAGACCCATAAATGGGGTAACTGCTACTCTGACGG-AGAATCGCCTC
TGAAAGAACTGGGGAAAACTCTGCAAGTGTATGTTATGCTGATTTAGATTTTGTCTGTTCACATCCATCTGGTGGAGGTA
GTTGAAGGAACAAAGACTCTTGAGCTGTTCCTGTGCTGCCTAAGTGTCAAGAGTAATAGTCACCCACTGTGCACAGGTAC
TTCTGGATAACTGTATGTCTGTCCATTCCCAATCTGAGTGCTATTTACATCAGAACAGAACCCTAGTATCTGTACACCAG
GTATATGTTCGTCTGTCTAGGACCTGAATTTTAAGATCTGTATCTACTGACCGGAACTGGGGAGA-------GAACAGAG
AACCCACTTTCTTTTTCATAAGATGATGT----AGACATTTATTTTTTCAGTGGAGGGTAGCGG---GAAGGAGGGTAGA
AGCAGCAGCTGAGGTGAGAGAAGCTTTCAAGCGCCTTCCTT---------------------------------------
----
--ATTTCAGTATGTAATTTAATAGAAGA-----TCCCTCC
AACCTTACATGTGGCAGGAAA----------TAACAGCTCTTGGCATTCTTAATAGCTCAGCTC----------------
----
>Gga_scaffold6225
GTACCTTAGGAACCTCTCTGGTGTCTGCAGGAGGAACCTAAATGATACAGAATCTCAGATCCAGGATAGGTGTAACATTT
CTCCTGGTGTATGAAAGTAACAGACAGATGATGCAGACCCATAAATGGGGTAACTGCTACTCTGACGG-AGAATCGCCTC
TGAAAGAACTGGGGAAAACTCTGCAAGTGTATGTTATGCTGATTTAGATTTTGTCTGTTCACATCCATCTGGTGGAGGTA
GTTGAAGGAACAAAGACTCTTGAGCTGTTCCTGTGCTTCCTAAGTGTCAAGTGTAATAGTCACCCATTGTGCACAGGTAC
TTCTGGATAACTGTATGTCTGTCCATTCCCAATCTGAGTGCAATTTACATCAGAACAGAACCATAGTATCTGTACACCAG
GTATATGTTTGTCTGTCTAGGACCTGAATTTTAAGATCTGTATCTACTGACCGGAACTGGGGAGA-------GGACAGAG
AACCCACTTTCTTTTTCATAAGATGATGT----AGACATTTATTTTTTCAGTGGAGGGTAGCGGGAAGAAGGAGGGTAGA
AGCAGCAGCTGAGGTGAGAGAGGCTTTCAAGCGCCTTCCTT---ATTTCATAGATTCATAGATTAAGGGTCGGAAGGGAC
CTAGTAGATCTTCTAGCCCGACCCCCCGCCAGGGCGGGAGAACAACAAAAAACCCCAAAAAGAGACGGGACTCAAATGAG
TCCAGCCAGGTAGGCATCCAGCCTCTTCTTAAAGACCCA---ATTTCAGTATGTAATTTAATAGAAGA-----TCCCTCC
AACCTTACATGTGGCAGGAAA----------TAACAGCTCTTGGCATTCTTAATAGCTCAGCTCTTTCC--TTGTTCTGT
GCAG---AAACTGCGG
>Asi_533442591
GTACCTTAGAAACCTCTCTGGTGTCTGCAGGAGGAACCCAAATGATAGAGAATCTCAGACCCAGGATAGGTGTAACATTT
CTCCTGGTGTATGAAAGTAAGAGACAGATGATGCAGACCCATAAATGGGGTAACTGCTCCTCTGATGGAAAAATCACCCC
TAAAAGAACTGGGGAAAACTCTGCAAGTGTATGTTATGCTGATTTAGATTTTGTCTGTTCACATCCATCTGG---AGGGA
GTTGAAGGAACAAAGACTCTTGAGCTGTTCCTGTGCTGCCTAAGTGTCAAGAGTAATAGTCACCCACTGTGCACAGGTAC
TTCTGGATAACTGTATGTCTGTCCATTGCTGATCTGAGTGCAATTTACATCAGAATAGAACCCTGGTATCTGTACACCAG
GTATATGTTAGTCT----AGGACCTGAATGTTAAGATCTGTATCTACTGACTGGAACTGGGGAGAGGGGAGGGGACAGAG
AACCCACTTTCTTTTTCATGAGATGATGTAGAGAGACATTTATTTTTTTAGTGGAGGGTAGTGGGAAGAAGGAGGGTAGA
AGAAGCAGCTGAGGTGAGAG--TCTTTCAAGCCCCTTCCTT---------------------------------------
----
--ATTTCAGTATGTAATTTAATAGAAGA-----TCCCTCC
AACCTTACATGTGGCAGGAAA----------TAACAGCTCTTGGCATTCTTACTAGCTCAGCTCTTTCC--TTGTTCTGT
GCAG---AAACTGCGG
>Ami_scaffold-730
GTACCTTAGAAACCTCTCTGGTGTCTGCAGGAGGAACCCAAATGATAGAGAATCTCAGACCCAGGATAGGTGTAACATTT
CTCCTGGTGTATGAAAGTAACAGACAGATGATGCAGACCCATAAATGGGGTAACTGCTCCTCTGATGGAAAAATCACCCC
TAAAAGAACTGGGGAAAACTCTGCAAGTGTATGTTATGCTGATTTAGATTTTGTCTGTTCACATCCATC---TGGAGGGA
GTTGAAGGAACAAACACTCTTGAGCTGTTCCTGTGCTGCCTAAGTGTCAAGAGTAATAGTCACCCACTGTGCATAGGTAC
TTCTGGATAACTGTATGTCTGTCCATTGCTGATCTGAGTGCAATTTACATCAGAATAGAACCCTGGTATCTGTACACCAG
GTATATGTTAGTCTGTCTAGGACCTGAATGTTAAGATCTGTATCTACTGACTGGAACTGGGGAGAGGGGAGGGGACAGAG
AACCCACTTTCTTTTTCATGAGATGATGTAGAGAGACATTTATTTTTTTAGTGGAGGGTAGTGGGAAGAAGGAGGGTAGA
AGAAGCAGCTGAGGTGAGAGAGTCTTTCAAGCCCCTTCCTT---------------------------------------
----
--ATTTCAGTATGTAATTTAATAGAAGA-----TCCCTCC
AACCTTACATGTGGCAGGAAA----------TAACAGCTCTTGGCATTCTTACTAGCTCAGCTCTTTCC--TTGTTCTGT
GCAG---AAACTGCGG
>Gallus_GB
GTACGT--

------------AAGATTCTTG---------------------------GCAGCACTGATCCCACACAGTGCCTTGA---
-----------------------------------GGGCGTCACCCATGTCAGA-------------ATCTTTAAAGCAG
--TCTCCCTCTCACAGCCGAGCAGG---------------
------------------AAAGAGGAGGG----GGGAGTTTACTTTTGCAGCAAAAGGCA-------GGCATGGATTTGA
GGAATCGGGTAATGTGA-------TTTTCAGCGTCTTTCTT---------------------------------------
----
------------------------------------TCC---ACGTCTGCATGTAACTTCATCAGGGAAGGAGAACCTTT
GTTTTTGGAGCTGTCAAAAGAAAAAAAACCCAAACAACTCCTGGCATTCTC-CTCACTCAGCCCTTCCCCTTTGTGCTTC
GCAG---AAAATACGG

GG10

>CR1-7
------------------------------------CAAGGATGGGCGGAGGAGAACAGGATGGGGTTTAACACAGACAA
GTGTAAGGTGATG-----------------------------CACCTGGGGAGGAACAATCGGCACCATACATACAGGCT
GGGGAACTCCCCTCTTGTCAGCACGGAGGTGGAAAAGGATCTGGGAGTCATTATTGACTCCAAGATGAACATGAGTCGGC
AATGTGGGGAGGCGGCTAGGAAGGCCAACTGCACCTTGTCGTGCATCCACAGATGCATCTCGAGCAGGTCCAAGGAGGTG
ATCCTCCCCCTCTATGCGGCACTGGTCAGGCCGCAGCTGGAGTACTGCGTCCAGTTCTGGGCGCCGCACTTTAAGAGGGA
TGTGGACAGCATCGAGAGGGTCCAGAGGAGGGCCACTCGTATGATCAAGGGACAGCAGGGTAGGTCCTATGAGGAAAGGT

TGCGGGACCTGAACCTGTTCAGCCTTCAAAAGAGAAGGCTGAGGGGGGATCTTGTGGCCTGTTATAAACTGGTTAGGGGG
GATCAGCAGGCGCTGGGGGAGTCCCTGTTCCCCCGAGCGCCGCAAGGAGTGACCAGGAACAATGGTCACAAGCTGGCGGA
GGGAAGGTTTAAGTTGGACATCAGGAGGCGCTACTTCACGGTCAGGGCGGCTAGGGTCTGGAACCAGCTTCCAAGTGAGG
TGGTGCAGGCTCCCACCCTGGGGGTTTTTAAGAGGAGGTTGGATGACCACCTTACTGGGGTCATTTGACCCCAGTGCTCT
TCCTGCCATGGCAGGGGGTCGGACTAGATGATCTGCTCAGGTCCCTTCCGACCCTAGAAACTATGAAACTA---------
-------TGAAA---------------------------------
>Cpo_7953_31441-32320
GCTGGGATGAATGGAGGACCCTGACATAGGACAGAAGAGGATAATGGGGAGAAAAGAGGGTATTTGTGAGGTGGAACAGA
AATAGAGGGAAGGAAGAGTATAAGCA--

--AGAGGG
GAA---ATGAGGGGAATGAGGCACCTGTCATAGGGGAAAAGAATG
>Tsc
GCTGGGATGAACGGAGGACCCTAACATAGGACAGAAGAGGATAATGGGGAGAAAAGAGGGTATTTGTGAGGAGGAACAGA
AATAGAGGGAAGGAAGAGTATAAGCA--

--AGAGGG
GAA---ATGAGGAGAATGAGGCACCTGTCATAGGGGAAAAGAATG
>Gga_15_9650-11250
GCTGGGATGAACGGAGGACCCTGACATAGGACAGAAGAGGATAATGGGGAGAAAAGAGGGTATTTGTGAGGAGGAACAGA
AATAGAGGGAAGGAAGAGTATAAGCA---AGAGGAGGAA---CACCTGGGGAGGAACAATCGGCACCACACATACAGGCT
GGGGAACTCCCCTCTTGTCAGCACGGAGGTGGAAAAGGATCTGGGAGTCATTATTGACTCCAAGATGAACATGAGTCGGC
AATGTGGGGAGGCGGCTAGGAAGGCCAACTGCACCTTGTCGTGCATCCACAGATGCATCTCGAGCAGGTCCAAGGAGGTG
ATCCTCCCCCTCTATGCGGCACTGGTCAGGCCGCAGCTGGAGTACTGCGTCCAGTTCTGGGCGCCGCACTTTAAGAGGGA
TGTGGACAGCATGGAGAGGGTCCAGAGGAGGGCCACTCGTATGATCAAGGGACAGCAGGGTAGGTCCTATGAGGAAAGGT
TGCGGGACCTGAACCTGTTCAGCCTTCAAAAGAGAAGGCTGAGGGGGGATCTTGTGGCCTGTTATAAACTGGTTAGGGGG
GATCAGCAGGCGCTGGGGGAGTCCCTGTTCCCCCGAGCGCCGCAAGGAGTGACCAGGAACAATGGTCACAAGCTGGCGGA
GGGAAGGTTTAAGTTGGACATCAGGAGGCGCTACTTCACGGTCAGGGCGGCTAGGGTCTGGAACCAGCTTCCAGGTGAGG
TGGTGCAGGCTCCCACCCTGGGGGTTTTTAAGAGGAGGTTGGATGACCACCTTATTGGGGTCATTTGACCCCAGTGCTCT
TCCTGCCATGGCAGGGGGTCGGACTAGATGATCTGCTCAGGTCCCTTCCGACCCTAGAAACTATGAAACTA---AGAGGG
GAA---ATGAGGAGAATGAGGCACCTGTCATAGGGGAAAAGAATG
>Asi_53345816
GCTGAGATGAACGGAGGACCCTGACATGGGGTAGAAGAGGATAATGGGGAGAAAAGAGGGTATTTGTGAGGAGGAACAGA
AATAGAGGGAAGGAAGAGTATAAGCA--

--AGAGGG
GAA---ATGAGGGGAATGAGGCACCTGTCATAGGGGAAAAGAATG
>Ami_3664_84401-85250
GCTGAGATGAACGGAGGACCCTGACATGGGGTAGAA---GACAGTGGGGAGAAAAGAGGGTATTTGTGAGGAGGAACAGA
AATAGAGAGAAGGAAGAGTATAAGCA--

--AGAGGG
GAA---ATGAGGGGAATGAGGCACCTGTCATAGGGGAAAAGAATG

GG12

>CR1-5B
--------------------CAGAAGGGTTTGGGAGAGACCTTGTTTCCCCTAGCGCCCCCTGGCATA------------
----ACAAGGAATAACGGCCACAAGTTGTTGGAGAGTAGGTTTAGATTAGACATCCGTAAGAACTACTTCACAGTCAGGG
CGGCTAGGATCTGGAACCAACTTCCAAGGGAAGTGGTGCTGGCTCCTACCCTGGGGGTCTTTAAGAGGAGGCTTGATGCC
TACCTGGCTGGAGTCATTTGAGCCCAGT-TTTTCTCCTGCCCAGGCAGGGGGTCGGACTTGAAGATCTACAAGGTCCCTT

```
CCGACCCTACTTCTATGATTCTATGA------------------------------------------------------
--------------------------------------------------------------------------------
-----------------------------------
>Cpo_2875_113841-115200
TCAGTCTAATGATCTAATGGTCTCTTTTGGCCTGTATCATAT---GTATCATAAAGTAGAATCATCTACATCA---TCTT
G-------------------------------------------------------------------------------
--------------------------------------------------------------------------------
--------------------------------------------------------------------------------
-------------------------------------TATATCATGAGTTCCAAATTCCAAATTCTTTGTAAGG-ACAAA
TCATATTTAGTAGCCTGGCAGGGGAGTGTCAAAAGACACAAGTATGTTTTGAGAGACTAATGGCAAAAAAA---------
-GTGTCAGGTGGAAGAGAACGCTACACACACATTC
>Mca
-----------------------------GCCTGTATCATATGAAGCATCACAGAGTAGAATCATCTACATCA---TCTT
G-------------------------------------------------------------------------------
--------------------------------------------------------------------------------
--------------------------------------------------------------------------------
-------------------------------------TATATCGTGAGTTCCAAATTCCAAATTCTTTGCAAGG-ACAAA
TCATATTTAGTAGCCTGGCAGGGGAGTGTCAAAAG--ACAAGTATGTTTTAAGAGACTAATGGCAAAAAAAAAAAAAAAA
AGGGTCGGGGGAAAAAAAGCACT----ACACTTCC
>Tsc
TCAGTCTAATGATCTAATGGTCTCTTTTGGCCTGTATCAGATGAAGCATCGCAAAGTAGAATCATCTACATCA---CCTT
G---ACAAGGAACAATGGACACAAATTATTAGAGAGTAGGTTTAGATTAGACATCCGCAAGAACTACTTCACAGTTAGAG
CAGCTAGGAGCTGGAACCAACTTCCTAGGGAAGTGGTGCTGGCTCCTACCCTGGGGATCTTTAAGAAGAGGCCTGATGAC
TACCTGGCTGGAGTCATCTGAGCCCAGTCTTTTCTCCCGCCCAGGCGGGGGGTTGGACTTGAAGATCTACAAGGTCCCTT
CCAACCCTACTTCTGTGATTCTATGA---TCTTG---TTGTATATCGTGAAAGAGTT-CAAATTCTTTGTAAGG-ACTAA
TCATATTTAGTAGCCTGGCAGGGGAGTGTC-AAAG--ACAAGTATGTTTTAAGAGACTAATGGCAAAAAAA---------
-GTGTCAGGTGGAAGAGAGCACT----ACACATTC
>Gga_85_5450-7050
TCAGTCTAATGATCTAATGGTCTCTTTTGGCCTGTATCAGATGAAGCATCGCAAAGTAGAATCATCTACATCA---CCTT
G---ACAAGGAACAATGGACACAAATTATTAGAGAGTAGGTTTAGATTAGACATCCGCAAGAACTACTTCACAGTTAGGG
CGGCTAGGAGCTGGAACCAACTTCCTAGGGAAGCAGTGCTTGCTCCTACCCTGGGGATCTTTAAGAAGAGGCCTGATGAC
TACCTGGCTGGACTCATTTGAGTCCAGTCTTTTCTCCCGCCCAGGCGGGGGGTTGGACTTGAAGATCTACAAGGTCCCTT
CCAACCCTACTTCTATGATTCTATGA---TCTTG---TTGTATATCGTGAAAGAGTT-CAAATTCTTTGTCAGG-ACTAA
TCATATTTAGTAGCCTGGCAGGGGAGTGTC-AAAGACACAAGTATGTTTTAAGAGACTAATGGCAAAAAAA---------
-GCGTCAGGTGGAAGAGAGCACT----ACACATTC
>Asi_533315637
TCAGTCTAATGACCTAATGGTTTCTTTTGGCCTGTATCATATGAAGCATCACAAAGTAGAATCATCTACATCA---TCTT
G-------------------------------------------------------------------------------
--------------------------------------------------------------------------------
--------------------------------------------------------------------------------
-------------------------------------TTGTATATCATGAAAGAGTTCCAAATTCTTTGTAAGGAAAAAA
TCATATTTAGTAGCCTGGCAGGGGAGTGTC-AAAGACACAAGTATGTTTTAAGAGACTAATGGCAAAAAA----------
-GTGTCAGGTGGAAGAGAGCACT------ACATTA
>Ami_12424_45951-47300
TCAGTCTAATGACCTAATGGTTTCTTTTGGCCTGTATCATATGAAGCATCACAAAGTAGAATCATCTACATCA---TCTT
G-------------------------------------------------------------------------------
--------------------------------------------------------------------------------
--------------------------------------------------------------------------------
-------------------------------------TTGTATATCATGAAAGAGTTCCAAATTCTTTGTAAGGAAAAAA
TCATATTTAGTAGTCTGGCAGGGGAGTGTC-AAAGACACAAGTATGTTTTAAGAGACTAATGGCAAAAAA----------
-GTGTCAGGTGGAAGAGAGCACT------ACATTA
```

GG13

```
>CR1-5B
--------------------------------------------------------------------------------
--------------------------------------------------------------------------------
-----------------------TCATA-------------GAATCATAGAAGTAGGGTCGGAAGGGACCTTGTAGATCT
TCAAGTCCGACCCCCTGCCTGGGCAGGAGAAAAACTGGGCTCAAATGACTCCAGCCAGGTAGGCATCAAGCCTCCTCTTA
AAGACCCCCAGGGTAGGAGCCAGCACCACTTCCCTTGGAAGTTGGTTCCAGATCCTAGCCGCCCTGACTGTGAAGTAGTT
CTTACGGATGTCTAATCTAAACCTACTCTCCAACAACTTGTGGCCGTTATTCCTTGTTATGCCAGGGGGCGCTAGGGGAA
ACAAGGTCTCTCCCAAACCCTTCTGGTCCCCCCTAGTGAGTTTATAGACGGTCACCAGGTCCCCCCTCAGCCTTCTCTTG
TGAAGGCTGAACAGGTTCAGGTCCCGCAGTCTCTCATTGTAGGGTCTGCCCTGTTGTCCCCGGATCATGCGGGTGGCCCT
CCTCTGGACCCTCTCAATGTTGTCCACATCCCTCCTGAAGTGGGGCGCACAGAACTGGACGCAGTACTCCAGCTGCGGCC
TGACCAGCGTCGCGTAGAGGGGGAGGATCACCTCCTTGGCCCTACTTGAGATGCACCTGTGGATGCACGACAAGGTCCGG
TTAGCCCTGCCAACCGTGACCTCGCATTGTCGGCCCATGTTCATCTTGGAGTCAAT----------------------AA
TGACTCCAAGATCCCTTTCTGCCTCCGTGCTCTCAAGAAGGGAGTTTCCCATCTTATAAGTGTGCTGCTGGTTACTACTG
-------------
>Cpo_3772_43841-44880
GATCTCAGTTCAGTGAGACCAGTGAATTTTCAACTTTATAGATCCATCACATAATCTTGTGTTCAATC----TTTTATGA
TTCTAAAGTATGGCATTTTTATATCAGAGAAATTGCTCTAGCATTAT-AAAAAAAAATACGGGTTGTTTTATGAAACAGT
GACGAAGCATTTAAAGAAAATATTCTTAG---------------------------------------------------
--------------------------------------------------------------------------------
--------------------------------------------------------------------------------
--------------------------------------------------------------------------------
--------------------------------------------------------------------------------
--------------------------------------------------------------------------------
--------------------------------------------------------------------------------
--------------------------------------------------------------------------------
```

```
-----------------------------------------------------------AATATA---TTATGCTCTTAT
GTTATGGTTAAGATAACTAGCAATAAATGTGTGTGATAAAAACCCCCTCTAAGAAGAACTTAATTTTTCTTAATACTTCC
TGCAGAATTTTTA
>Ote
GATCTCAGTTCAGTGAGACCAGTGAATTTTCAACTTTATAGATCCATCACATAATCTTGTGTTCAATC----TTTTATGA
TTCTAAAGTATGGCAGTTTTATATCAGAGAAATTGCTCTAGCATTAT-AAAAAATAATACAGGTTGTTTTATGAAACAGT
GACGAAGCATTTAAAGCAAATATTCTTAG---------------------------------------------------
--------------------------------------------------------------------------------
--------------------------------------------------------------------------------
--------------------------------------------------------------------------------
--------------------------------------------------------------------------------
--------------------------------------------------------------------------------
--------------------------------------------------------------------------------
--------------------------------------------------------------------------------
-----------------------------------------------------------AATATA---TTATGCTCTTAT
GTTATGGTTAAGATAACTAGCAATAAACGTGTGTGATAAAAACCCCCTCTAAGAA---CTTAATTTTTCTTAATACTTCC
TGCAGAATTTTTA
>Mca
GATCTCAGTTCAGTGAGACCAGTGAATTTTCAACTTTATAGATCCATCACATAATCTTGTGTTCAATC----TTTTATGA
TTCTAAAGTATGGCATTTTTATATCAGAGAAATTGCTCTAGCATTAT-AAAAAATAATACGGGTTGTTTTATGAAACAGT
GACGAAGCATTTAAAGCAAATATTCTTAG---------------------------------------------------
--------------------------------------------------------------------------------
--------------------------------------------------------------------------------
--------------------------------------------------------------------------------
--------------------------------------------------------------------------------
--------------------------------------------------------------------------------
--------------------------------------------------------------------------------
--------------------------------------------------------------------------------
-----------------------------------------------------------AATATA---TTATGCTCTTAT
GTTATGGTTAAGATAACTAGCAATAAATGTGTGTGATAAAAACCCCCTCTAAGAAGAACTTAATTTTTCTTAATACTTCC
TGCAGAATTTTTA
>Tsc
GATCTCAGTTCAGTGAGACCAGTGAATTTTCAACTTTATAGATCCATCACATAATCCTGTGTTAAATC----TTTTATGA
TTCTAAAGTATGGCATTTTTATATCAGAGAAATTGCTGTAGCATTATGAAAAAATAACATGGGTTGTTTTATGAAACAGT
GATGAAGCATTCAGAGCAAATATTCTTAG---AATATA---GAATCATAGAAGTAGGGTCGGAAGGGAGCTTGTAGATCT
TCAAGTCCGACCCCCCGCCTGGGCGGGAGCAAAACTGGACTCAAATGAGTCCAGCCAGGTAGGCATCAAGCCTCTTCTTA
AAGATCCCCAGGGTAGGAGCCAGCACCACTTCCCTAGGAAGTTGGTTCCAGCTCCTAGCCGCCCTAACTGTGAAGTAGTT
CTTGCGGATGTCTAATCTAAACCTACTCTCCAATAATTTGTGTCCATTATTCCTTGTCATGCCAGGAGGTGCTAGGGGAA
ACAGTGTCTCCCCCAAACCCTTCTGGTCCCCCCTAGTGAGTTTATAGACAGTCACCAGGTCCCCCCTCAGCCTTCTCTTG
TGAAGGCTGAACAAGTTTAGGTCCCACAGTCTTTCATTGTAGGGTCTGCCCTGTTGTCCCCGGATCATGCGGGTGGCCCT
CCTCTGGACTCTTTCAGTGTTGTCCACATCCCTCGTGAAATGGGGCGCACAGAACTGGACGCAGTACTCCAGCTGTGGCC
TGACTAGCGTCGCATAGAGGGGGAGGATCACCTCCCTGGCTGTACTTGAGATGCACCTATGGATGAACGACAAGGTCCGG
TTAGCCCTGCCAACCATGACCTCGCATTGTCGGCCCATGTTCATCTTGGAGTCAAT---AATATA---TTATGCTCTTAT
GTAATGGTTAAGATAACTAGCAATAAATGCGTGTGATAAAAAACCCCTCTAAGAAGAATTTAATTTTTCTTAATATTTCC
TGCAGAATTTTTA
>Gga_99_50150-51750
GATCTCAGTTCAGTGAGACCAGTGAATTTTCAACTTTATAGATCCATCACATAATCCTGTGTTAAATC----TTTTATGA
TTCTAAAGTATGGCATTTTTATATCAGAGAAATTGCTGTAGCATTAT-AAAAAATAACATGGGTTGTTTTATGAAACAGT
GATGAAGCATTTAGAGCAAATATTCTTAG---AATATA---GAATCATAGAAGTAGGGTCGGAAGGGAGCTTGTAGATCT
TCAAGTCCGACCCCCTGCCTGGGCGGGAGAAAAACTGGACTGAAATGAGTCCAGCCAGGTAGGCATCAAGCCTCTTCTTA
AAGATCCCCAGGGTAGGAGCCAGCACCACTTCCCTTGGAAGTTGGTTCCAGCTCCTAGCCGCCCTAACTGTGAAGTAGTT
CTTGCGGATGTCTAATCTAAACCTACTCTCCAATAATTTGTGTCCATTGTTCCTTGTCATGCCAGGGGGTGCTAGGGGAA
ACAGTGTCTCCCCCAAACCCTTCTGGTCCCCCCTAGTGAGTTTATAGACAGTCACCAGGTCCCCCCTCAGCCTTCTCTTG
TGAAGGCTGAACAGGTTTAGGTCCCGCAGTCTTTCATTGTAGGGTCTGCCCTTTTGTCCCCGGATCATGCAGGTGGCCCT
CCTCTGGACTCTTTCAGTGTTGTCCACATCCCTCGTGAAATGGGGCGCACAGAACTGGATGCAGTACTCCAGCTGTGGCC
TGACCAGCGTCGCATAGAGGGGGAGGATCACCTCCCTGGCTCTACTTGAGATGCACCTGTGGATGCACGACAAGGTCCGG
TTAGCCCTGCCAACCATGACCTCGCATTGTCGGCCCATGTTCATCTTGGAGTCAAT---AATATA---TTATGCTCTTAT
GTTATGGTTAAGATAACTAGCAATAAATGCGTGTGATAAAAAACCCCTCTAAGAAGAACTTAATTTTTCTTAATATTTCC
TGCAGAATTTTTA
>Asi_533382713
GATCTCAGTTCAGTGAGACCAGTGAATTTTCAATGTTATAGATCCATCACATAATCCTGTGTTAAATC-TTCTTTTACGA
TTTTAAAGTATGGCGTTTTTATATTAGAGAAATTGCTCTAGCATTAT-AAAAAATAGTATGGGTTTTTTTATGAAACGGT
GATGAAGCATTTAAAGCAAATATTCTTAG---------------------------------------------------
--------------------------------------------------------------------------------
--------------------------------------------------------------------------------
--------------------------------------------------------------------------------
--------------------------------------------------------------------------------
--------------------------------------------------------------------------------
--------------------------------------------------------------------------------
--------------------------------------------------------------------------------
-----------------------------------------------------------AATATA---TTATGCTCTTAT
GTTATGGTTAAGATGACTAGTAATAAATGTGCATGATAAAAACCCCCTGTAAGAAGAACTTAATTTTTCATAATACTTCC
TGCAGAATTTTTA
>Ami_9339_50351-51300
GATCTTAGTTCAGTGAGACCAGTGAATTTTCAATGTTATAGATCCATTGCATAATCCTGTGTTAAATCTTTTTTTTACGA
TTTTAAAGTATGGCGTTTTTATATTAGAGAAATTGCTCTAGCATTAT-AAAAAATAGTATGGGTTTTTTTATGGAACGGT
GATGAAGCATTTAAAGCAAATATTCTTAG---------------------------------------------------
--------------------------------------------------------------------------------
```


---AATATA---TTATGCTCTTAT
GTTATGGTTAAGATGACTAGTAATAAATGTGCTTGATAAAAACCCCCTGTAAGAAGAACTTAATTTTTCGTAATACTTCC
TGCAGAATTTTTA

GG14

>CR1-5B

---TCATAG-
-----AATCATAGAAGTAGGGTCGGAAGGGACCTTGTAGATCTTCAAGTCCGACCCCCTGCCTGGGCAGGAGAAAAAC--
------------------TGGGCTCAAATGACTCCAGCCAGGTAGGCATCAAGCCTCCTCTTAAAGACCCCCAGGGTAGG
AGCCAGCACCACTTCCCTTGGAAGTTGGTTCCAGATCCTAGCCGCCCTGACTGTGAAGTAGTTCTTACGGATGTCTAATC
TAAACCTACTCTCCAACAACTTGTGGCCGTTATTCCTTGTTATGCCAGGGGGCGCTAGGGGAAACAAGGTCTCTCCCAAA
CCCTTCTGGTCCCCCCTAGTG-------------------AGTTTATAGACGGTCACCAGGTCCCCCCTCAGCCTTCT--
----

>Cpo_12416_22401-23680
AAAGGCTTGCAAATTGAGAATATTAAAAAAAAACT--TCCCAAAACAAACAAACCCCAATCTCCCC------AGCACCACC
TAAAATATAAAATGTAGCTCTTTTCTTGGAATGGAAAATAGATGGAGAAGAACCTATCACTC------------------
----

------------------------AGCTTC---CCTCAGTGGGTTAAATATCTACTTCCCATCAGCTAATCAGCCCCAGC
AATAAGGACTCATCACCAGGATTATATTGCATTTCTGTGAATCAGCAGTCTACTGATGGCAATTCCTAACCACAGTGAAT
AAACA
>Tsc
AAAGTCTTGCAAATTGAGAATATTAAAAAAAAAACCTCCCAAAAACAAACAAA--CAAATCTCCCCTTC---ACCACCACC
TAAAATATAAAATGTAGCTCTTTTCTTGGAATGGAAAATAGATGGAGAAGAACCTATCACTC------------------
----

------------------------AGCTTC---CCTCAGTGGGTGAAATATCTACTTCCCATCAGCTAATCAGCCCCAGC
AATAAGGACTCATCACCAGGATTATATTGCATTTCTGTGAATCAACAGTCTACTGATGGCAATTCCTAACCACAGTGAAT
AAACA
>Gga_164_359500-361100
AAAGTCTTGCAAATTGAGAATATTAAAAAAAAAACCTTCCAAAAACAAACAAACCCCAATCTCCCCTTC---ACGACCACC
TAAAATATAAAATGTAGCTCTTTTCTTGGAATGGAAAATAGATGGAGAAGAACCTATCACTC---AGCTTC---ATAGTT
TCATAGTTTCATAGATTTAGGGTCGGAAGGGACCTAGTAGATCTTCTAGCCCAACCCCCCGCCAGGGCGGGAGAACAACA
AAACCCCAAAACAAGAAACGGGACTCAAATGAGTCCAGCCAGGTAGGCATCCAGCCTCTTCTTAAAGACCCCCAGGGTAG
GGGCCAGCACCACTTCCCTTGGAAGTTGGTTCCAGCTCCTAGCCGCCCTAACTGTGAAGTAGTTTTTGCGGATGTCCAAT
CTATACCTCCTCTCCATCAACTTGTGCCCATTACTCCTTGTTATGCCAGCGGGTGCTAGGCATAACAAGGAGGATATAAC
AAGTGGATATTTCACCCACTG---AGCTTC---CCTCAGTGGGTGAAATATCTACTTCCCATCAGCTAATCAGCCCCAGC
AATAAGGATTCATCACCAGGATTATATTGCATTTCTGTGAATCAACAGTCTACTGATGGCAATTCCTAACCACAGTGAAT
AAACA
>Asi_533402431
AAAGGCTTGCAAATTGAGAATATAAAAAAAAAA---CCCCCAAACAAACAAACCCCAATCTCCCCC-----ACCCCCACC
TAAAATATAAAATGTAGCTCTTTTCTTGGAATGGAAAATAGATGGAGAAGAACCTATCGCTC------------------
----

------------------------AGCTTC---CCTCAGTGGGTGAAATATCTACTTCCCATTAGCTAATCAGCCCCAGC
AATGAGGACTCATCATCAGGATTATATTGCATTTCTGTGAATCAACTGTCTACTGATGGCAATTCCTAACCACAGTGAAT
AAACA
>Ami_9908_985001-986250
AAAGGCTTGCAAATTGAGAATATAAAAAAAAAAA---CCCAAAACAAACAAACCCCAATCTCCCCACCCCAACCCCCACC
TAAAATATAAAATGTAGCTCTTTTCTTGGAATGGAAAATAGATGGAGAAGAACCTATCGCTC------------------
----

------------------------AGCTTC---CCTCAGTGGGTGAAATATCTATTTCCCATTAGCTAATCAGCCCCAGC
AATGAGGACTCATCATCAGGATTATATTGCATTTCTGTGAATCAACTGTCTACTGATGGCAATTCCTAACCACAGTGAAT
AAACT

CG62

>CR1-2


```
--------------------------------------------------------------------------------
--------------------------------------------------------------------------------
--------------------------------------------------------------------------------
--------------------------------------------GAAGAATTTCTTTACTG-TCCGAGCCCCAAGGTCTG
GAATAGCCTGCCATCGGAGGTGGTTCAAGCACCTACATTGAACA------------------------------------
--CCTTCAAGAGAAAA-TTGGATGCTTATCTTGCTGGGATCCTATGACCCCAGCTGACTTCCTGC-CCCTCGGGCAGGGG
GCTGGACTCGATGATCTTCCGAGGTCCCTTCCAGCCCTAATGTCTATGAAATCTATGAA---------------------
--------------------------------------------------------------------------------
--------------------------------------------------------------------------------
--------------------------------------------------------------------------------
--------------------------------------------------------------------------------
--------------------------------------------------------------------------------
--------------------------------------------------------------------------------
--------------------------------------------------------------------------------
--------------------------------------------------------------------------------
------------------------------------------------------------------
>Cpo_scaffold42343_21986-23377_RC
TCCAGGTAATAGACATGAATGACTATCAGAGACGACGATTTGCTTCCCATATCATTGACAGTTTGTTTAACACTGTCACT
GACAAGAAGATTGCTATTTTAGGATTTGCATTTAAAAAAGACACGGGAGACACAAG---GTAACGAGATTCATCAGTC-T
TGTTAACAGTACTTACCTATCATTGGCTCCATCGACATTGCAAAAACAGTTGTT--------AAACAATTATTTCAATTG
TATGCATGGA-AAAGGACAACAAATAAATTACCTGTTATATCTGAA------------CTAGTT-----TATTCTGGGTT
GAATGCCTGAGGCCAAA-----------TTGTTTTTTTTTTAAAGCAGATTTCTAGAAAGCTTTGCAGATGTGTCTAGAG
CACAGATAATACAATGGTGAAGACCCACAAGTACTATACTTACGTTGACAGAAAACCCACTGAACTGAGTGAAAGCAAA-
---TTAA--TATAAAACATGCAGATACTTTCTCTGCCCCTTTAGC-------------TGGAAACA------TTAACATA
GCCTACACCTTAGCA-TTAATTTCCATAAGAGGCTTAGCCACTACCATGGCTCTCCAGCTATAGTCAGGATTATCCATG-
--CCTCCAAGAGAAAGCTTGGATGTTTATCTTGCTGGGATCCTATGACCCCTGCTGACTTCCTGTGACCTGGGGCAGGGG
GCTGGACTTGATGATCTTCTGAGGTCCCTTCCAACCCTAATATCTATGAAATCCGTCAA---CAAATTCCCTTTTAATGT
AAGAGGCATTCCCATTGCATCTAGTGCTTGCTTGGAGCCAAGTGGGTGTCCTTGCTGGAATGAATCATCGATAACTATTC
TAATACA--TAATCCTGAGATTTTAGTATCTGTTTGATGTGACTCG-GCAGAAGAGTCCTGCATGATCAAACAGGTCTTG
CAATCTGTGACTATCTAGCACAGCTTGGCCTTGTTCACACAACTTACCTGAACTGTGTTCTTGGTACAGTCTAAATATGA
TGTAAACAGTGTTTAAGGTATGTCTTACAAATTTATCCTTTAAACTCTGTAATGGGGATTTTTTTTTGTCTGGCAACTAG
AAACTAATGTTGGGGTGTTTTTTTATTTGTTGGGGGTTTTTTAACTTCTGAAACCACATTTTTCTAATG-TTGCTTTTTT
GCTGCAA--------------TGTGATAATCAATGCAAAACCTGTTCAAAAGCTCTAAGAGCA--------TACAACTGA
AAGTGTTCGTATATGCCTTTATCGAAAATAAACCCAAACATTT-GTAAGGCTCATTAATTGGTTCATT-----------T
ATTTCCTTTTAG---AGAGTCTTCCAGTATCTACATCAGCAAGTATCTAATGGATGAAGGAGCAAAACTCCATATATATG
ATCCTAAAGTGCCAAGGGAACAAATCATCCTGGATCTCTCGCATCCAGGCGTCTCGGAAGATGACC
>Gga_scaffold17770
TCCAGGTAATAGACATGAATGACTATCAGAGACGACGATTTGCTTCCCATATCATTGACAGTTTGTTTAACACTGTCACT
GACAAGAAGATTGCTATTTTAGGATTTGCATTTAAAAAAGACACGGGAGACACAAG---GTAACGAGATTCATCAGTC-T
TGTTAACAGTACTTACCTATCATTGGCTCCATCGACATTGCAAAAACAGTTGTT--------AAACAATTATTTCAATTG
TATGCATGGA-AAAGGACAACAAATAAATTACCTGTTATATCTGAA------------CTAGTT-----TATTCTGGGTT
AAATGCCTGAGGCCAAATTGTTTTGTTTTTGTTTTTTTTTTAAAGCAGATTTTTAGAAAGCTTTGCAGATATGTCTAGAG
CACGGATAATACAATGGTGAAGACCCACAAGTACTATACTTACATTGACAGAAAACCCACTGAACTGAGTGAAAGCAAAC
TATCTAA--TATAAAACATGCAGATACTTTCTCTGCCCCTCTAGC-------------TGGAAACA------TTAACATA
GCCTACACCTTGGCA-TTAATTTCCATAAGAGGCTTAGCCACTGTCATGGCTCTCCAGCTATAGTTGGGATCATCCATG-
--CCTCCAAGAGAAATTTTGGATGTTCATCTTGCTGGGATCCTATGACCCCTGCTGACTTCCTGTGACCTAGGGCAGGGG
GCTGGACTTGATAGTCTTCTGAGGTCCCTTCCAACCCTAATGTCTATGAAATCCCTCAA---CAAATTCCCTTTTAATGT
AAGAGGCATTCCCATTGCATCTAGTGCTTGCTTGGAGCCAAGTGGGTGTCCTTGCTGGTATGAATCATCGATAACTGTTC
TAATACA--TAATCCTGAGATTTTAGTATCTGTTTGATGTGACTCG-GCAGAAGAGTCCTGCATGATCAAACAGGTCTTG
CAATCTGTGAATATCTAGCAGAGCTTGGCCTTGTTCACACAACTTACCTAAACTGTGTTCTTGGTACAGTCTAAATATGA
TGTAAACAGTGTTTAAGGTATGTCTTACAAATGTATCCTTTAAACTCTGTAATGGA---TTTTTTTTGTCTGGCAACTAG
AAACTAATGTTGGGGTGTTTTTTTATTTGTTGGGGTTTTTTTAACTTTTGAAACCACATTTTTCTAACG-TTGCTTTTTT
GCTGCAA--------------TGTGGTAATCAATGCAAAACCTGTTCAAAAGCTCTAAGAGCA--------TACAACTGA
AAGTGTTCGTATATGCCTTTGTTCAAAATAAACCCAAACATTT-GTAAGGCTTATTAATTGGTTCATT-----------T
ATTTCCTTTTAG---AGAGTCTTCCAGTATCTACATCAGCAAGTATCTAATGGATGAAGGAGCAAAACTCCATATATATG
ATCCTAAAGTGCCAAGGGAACAAATCATCCTGGATCTCTCGCATCCAGGCGTCTCGGAAGATGACC
>Ami_JH731248_267734-269121
TCCAGGTAATAGACATGAATGACTATCAGAGACGACGATTTGCTTCCCATATCATTGACAGTTTGTTTAACACTGTCACT
GACAAGAAGATTGCTATTTTAGGATTTGCATTTAAAAAAGACACGGGAGACACAAG---GTAATGAGATGCATCAGTCTT
TGTTAACAGTACAAACCTATCATTGGCTCCATCGACATTGCAAAAACAGTTGCT--------AAACAATTATTTCAATTG
TATGAATGGA-AAAGGACAACAAATAAATTACCTGTTATATTTGAA------------CTAGTT-----TATTCTGGGTT
AAATGTCTGAGGCCAAATTGTT------TTGTTTTTTGTTTACAGCAGATTTTTAGAAAGCTATGCAGATATGTCTAGAA
CACAGATAATACAATGGTGAAGACCCACAAGTACTGTACTTGCGTTGACAGAAAACCCACTGAACTGAGTGAAAGCAAAC
GATCTAA--TATAAAACATGCAGATACTTTCTCTGCCCCTCTAGC-------------TGGAAACA----TTTTAACATA
GCCTACACCTCAGCA-TTAATTTCCATAAGAGGCTTGACCACTTCCATGGCTCTCCAGCTATAGTCAGGATCATCCATG-
--CCTCCAAGAGAAA-TTTGGATGTTTATCTTGCTGGGATCCTATGACCCTTGCTGATTTCCTGCGACCTGGGGCAGGGG
GCTGGACTCGA---TCTTCTGAGGTCCCTTCCAGCCCTAATGTCTATGAAATTCCTCAA---CCAATTACCTTTTAATGT
AAGAGGCATTCCCATTGCATTTAGTGCTTGCTTGGAGCCAAGTGGGTGTCCTTACTGGTATGAATTATCGATAATTATTC
TAATACA--TAATCCTGAGATTTTAGCATCTGTTTGATGCGACTCG-GCAGAAGAGTCCTGCATGATCAAACAGGTCTTG
CAATCTGTGGCTATCTAGCACAGCTTGGCCTTGTTCACACAACTTAACTAAACTGTGTTCTTGGTACAGTTTAAATATGA
TGTAAACAGTGTTTAAGGTTTGTCTTACAAATGTATCCTTTAAACTCTGTAATGGGG--ATTTTTTTGTTTGACAACTAG
AAACTAATGTTG----------TTTTTTTTTGTTGTTTTTTTAACTTTTGAAACCACACTTCTCTAACATTTGCTTTTTT
GCTGCTA--------------TGTGGTGATCAATGCAAAACCCGTTCAAAAGCTCTAACAGTG--------TACAACTGA
AAGTGTTTGTAGATGCCTTTGTCCAAAATAAACCCAAACATTT-GTAAGGCTCATTAATTGGTTCGTT-----------T
ATTTCC-TTTAG---GGAGTCTTCCAGTATCTACATCAGCAAGTATCTAATGGATGAAGGAGCAAAACTCCATATATATG
ATCCTAAAGTGCCAAGGGAACAAATCATCCTGGATCTCTCGCATCCAGGCGTCTCGGAAGATGACC
>Asi_AVPB01116058_18900-20247
TCCAGGTAATAGACATGAATGACTATCAGAGACGACGATTTGCTTCCCATATCATTGACAGTTTGTTTAACACTGTCACT
```

```
GACAAGAAGATTGCTATTTTAGGATTTGCATTTAAAAAAGACACGGGAGACACAAG---GTAATGAGATGCATCAGTCTT
TGTTAACAGTACGTACCTATCATTGGCTCTATCGACATTGCAAAAACAGTTGCT--------AAACAATTATTTCAATTG
TATGAATGGA-AAAGGACAACAAATAAATTACCTGTTAT--CTGAA------------CTAGTT-----TATTCTGGGTT
AAATGCCTGAGGCCAAATTGTT------TTGTTTTTTATTTAAAGCAGATTTTTAGAAAGCTATGCAGATATGTCTAGAA
CACGGATAATACAATGGTGAAGACCCACAAGTACTGTACTTATGTTGACAGAAAACCCACTGAACTGAGTGAAAGCAAAC
GATCTAA--TATAAAACATGCAGATACTTTCTCTGCCCCACTAGC-------------TGGAAACA-----TTTAACATA
GCCTACACCTCAGCA-TTAATTTCCATAAGAGGCTTGACCACTTCCATGGCTCTCCAGCTATAGTCAGGATCATCCATG-
--CCTCCAAGATAAATTT-GGATGTTTATCTTGCTGGGATCCTATGACCCTTGCTGATTTCCTGTGGCCTGGGGCAGGGG
GCTGGACTCGAT---CTTCTGAGGTCCCTTCCAGCCCTAATGTCTATGAAATTCCTCAA---CCAATTCCCTTTTAATGT
AAGAGGCATTCCCATTGCATTTAGTGCTTGCTTAGAGCCAAGTGGGTGTCCTTACTGGTATGAACTATCGATAATTATTC
TAATACA--TAATCCTGAGATTTTAGCATCTGTTTG---TGACTCG-GCAGAAGAGTCCTGCATGATCAAACAGGTCTTG
CAATCTGTGACTATCTAGCACAGCTTGGCCTTGTTCACACAACTTAACTAAACTGTGTTCTTGGTACAGTCTAAATATGA
TGTAAACAGTGTTTAAGGTTTGTCTTACAAATGTATCCTTTAAACTCTGTAATGGGG--ATTTTTTTGTTTGACAACTAG
AAACTAATGTTGGTG---TTTTTTGTTTGTT---GTTTTTTTAACTTTTGAAACCACACTTCTCTAACA-TTGCTTTTTT
GCTGCTA--------------TGTGGTGATCAATGCAAAACCCGTTCAAAAGCTCTAACAGCG--------TACAACTGA
AAGTGTTTGTAGATGCCTTTGTCCAAAATAAACCCAAACATTT-GTAAGGCTCATTAATTGGTTCATT-----------T
ATTTCC-TTTAG---GGAGTCTTCCAGTATCTACATCAGCAAGTATCTAATGGATGAAGGAGCAAAACTCCATATATATG
ATCCTAAAGTGCCAAGGGAACAAATCATCCTGGATCTCTCGCATCCAGGCGTCTCGGAAGATGACC
>Gallus_GB_Chr4_68877817-68878797
TTCAGGTAATAGACATGAATGATTATCAGAGGCGCAGATTTGCTTCCCGTATTATTGACAGCCTGTTCAATACTGTCACT
GATAAGAAGATTGCTATCTTAGGATTTGCATTCAAAAAAGATACTGGGGACACAAG---GTAAGAAAGTGAATTCAAT-T
AATTAATATGGATTCCCTTGCTACAGCTTGCTACAGCTACATTTGCTACAGCTGTGCAGCTAAACCCACCAGTTCAGTTC
TGTTAACAAAAACAGGTCAGACATGGGATCCTTTACCTCTTTTGAA--TCAATGAT-TCATATTA----TAGTGCTTCTT
AGATGGGTGAGACAG-------------TTGATTTTT--------------------GAGCTCTACA-ACATACATGAAT
CGTTCAGAAATTAA---------------------------------ACAGATAACTTGTTCTATTTAGT----------
-CCCTAACTTGCAAAAGTAACAGCTGTTTCTTGAGTCCTTCCAGT-----------AATGCTAAGA----CTTTTGCA--
--------GCTGACT-CAAATTGCTATTAGAAGCCAAGGTTCCATCTTTGTTTTCC--------------CCAAAGATG-
--------------------------------------------------------------------------------
-------------------------------------------------------------GCTAATTCTCTTTTCTTGT
AAGAGGCA---GTCTTGATACAATTCTCCCCAACTACAGTTTGCATGG--------------------------------
-CATGCT--TAGGAGTGATGTTTTGTTACCTAT--------GGTCA-GTAGGAGTA--------------------CTGG
GAAGTGGTATTTATCT-----------------CTTACATGATTCAGC--------------------------ATAAGA
GATACAAACTGTTTAAA------------------------------------------ATTACTCTCCTTGGC------
-----------------------------------------------------------CTCCCTCAAGACTTTTATTTT
GCTGCTGGCATAGATAATTGCTAAGACATTTAATATAAAACCAGTAC-AAAGCTCTGGCACTGAGTATCATGACAATTGA
AGATTT-------------------------------GTATTA-TTAAAGCGTATCAGCTGGTTCTTTAACTGTTTAACT
CTCTTC-TGTAG---AGAGTCATCCAGCATCTACATTAGCAAGTATTTAATGGATGAAGGAGCAAAGCTCCATATCTATG
ACCCTAAAGTACCGAAGGAACAAATCATCTTGGATCTGTCACATCCAGGTGTCTCAGAAGATAACC
>Turkey_GB_chr4_52191232-52192287
TTCAGGTGATAGACATGAATGATTATCAGAGGCGGAGATTTGCTTCCCGTATTATTGACAGCTTGTTCAATACTGTCACC
GATAAGAAGATTGCTATCTTAGGATTTGCATTCAAAAAAGATACTGGGGACACAAG---GTAAGAAAGTGAATTCGAT-T
AATTAACATGGATTCCCT--------------------GCACTAGATACAGCTGTGCAGCTAAACCCATAAGTTCACTTC
TATTAACAAAAACAGCTCAGATATGGGATCCTTAACCTCATTTGAA--TCAATGAT-TTGTATTA----TAGTGCTTCTT
AAATGGGTGAGACAG-------------TTGATTCTT--------------------GATCTCTACA-ACATACCTGAAT
CATTCAGAAATTAA---------------------------------ACAGATAACTTGTTCTATTTAGT----------
-CCCTA-CTTGCAAAAG------GTGCTTCTTGAGTCCTTCCAGT-----------AAAGAATGCTAACACTTTTGCA--
--------GCTGACT-CAAATTGCTATTAGAAGCCAAAATTCCATCTTTGTT-TCC--------------CCAAAGATG-
--------------------------------------------------------------------------------
-------------------------------------------------------------GCTAATTCTCTTTTCTTAT
AAGAGACA---GTCTTGATACAGTTCTCTCCAACTACAATTTGCATGA--------------------------------
-CATATT--TAGGAGAGATGCCTTGTTACCTAT--------GGTCA-GTAGCAGTA--------------------CTGC
GACTTGGTACCTATTT-----------------CTTCCATGATTCAGC---------------------------AAAGA
GATGCAAACTTTTTAAA------------------------------------------ATTACTCTCCTTGGC------
-----------------------------------------------------------CTCCCTCAAGACTTTTATTTT
GCTGCTGGCATAGATTATTGCTAAGACATTTAATGTAAAACCAGTAT-AAAGCTCTGACATTGAGCATTACGACAATTGA
AGATTT-------------------------------GTGTTA-TTAAAACATACCAGCTGGTTCTTTAACTGTTTAATT
CTCTTC-TGTAG---AGAGTCATCTAGCATCTACATTAGCAAGTATTTAATGGATGAAGGAGCAAAGCTCCATATCTATG
ACCCTAAAGTACCAAAGGAACAAATCATCTTGGATCTCTCACATCCAGGTGTCTCAGAAGACAACC
>Taeniopygia_GB_chr4_48120997-48121955
TTCAGGTAATAGACATGAATGATTATCAGAGAAGAAGATTTGCTTCCCGCATTATTGACAGCTTGTTTAATACTGTCACT
GACAAGAAGATTGCTATTTTAGGGTTTGCTTTCAAAAAGGATACTGGGGACACAAG---GTAAGCAAGCAAGCTCAGT-T
CACC-GCATGGATTCC-TTAC----------AGCTGTCACCTGCCACCCAGCTA-------ATGCCAACCACTGAACTGA
ATTGAT-------GGCTGCATATTGGGACCCTATTTTTTTGTGACT-GCATTGGGTTGTAACAAGGAT-TAGTCTTTCTT
AAATGGGTGAG--AA-------------GTGATTCTT--------------------GACCTCTACA-GCAGACCTGAGT
CATGCAGAAGTTGC---------------------------------AGATGATTCTAGGCCTTATTAAT----------
-TTGTA----CAAGCAAAGATAGATTTTGACAGACTCCTTCCATTCTTCAGAAAAGAATGCAAACA----CTTTTGCA--
--------GCTGGTTCCAAGTTGCTTTGTGAAACCAAAGCT--------------T--------------CCTGAAATG-
--------------------------------------------------------------------------------
-------------------------------------------------------------GTTCATTCTGA-CCTCACA
GGAGAACAA---ACTTGTAACAA--CTCTTCAACTGCAGTTTGTGGGG--------------------------------
-AAAGCTAATAAACTCTAGGCCTTTACAATTTT--------GGTCATTTAGGAGTC--------------------ATCA
GACTCATTATC--TTT-----------------CTTGGATGATTCAGC--------------------------AAAAGA
GTTACAGACCTTTTAAT------------------------------------------ATTATTCTCCTTGGT------
-----------------------------------------------------------GTCCATCAAGATTACACATTT
CCTGCTGGCAT-GGTTGCCACTAAGATATTTAATGTAAAAATTATATAAAACTCTGGCATTGCATATCATGATACATGA
GAACTT-------------------------------TCATTACTTGGAACAAGCAACCTGGTTCTTTAACTGATTGATT
CCCTTC-CTTAG---AGAGTCATCCAGTATCTACATTAGCAAGTATTTAATGGATGAAGGAGCAAAGCTGCATATCTATG
ACCCTAAAGTACCTAAGGAACAAATCATCTTAGATCTCTCACATCTGGGTGTCTCAGAAGATAACC
```

CG66

```
>CR1-9B_Ami
--------------------------------------------------------------------------------
--------------------------------------------------------------------------------
--------------------------------------------------------------------------------
----------------------------------------------------------------------------TTTA
TCAAGGGAGGACAACAGGGAATTGGGATGCGCTATTTACCAGAGCGCCCCAGGGAGTAACTAGGAATAATGGGTGCAAAC
TAGTGGAGAGTAGATTTAGGTTAGACATTAGGAAGAAATTTTTTACAGTAAGGGTGGCCAGAATCTGGAATGGGCT----
----------------TCCAAGAGAGGTGGTGCTATCACCTAACTTGGAGGTCTTCAAGAGGAGGCTTGATAGTCACCTG
GCTGGGGTCATCTGACCTCGGTCCTCTTTCCTGCCAGGGGCAGGGGGTCGGACACGATGATCCATTGTGGTCCCTTCCGA
CTCTACAATCTATGAATCTATGAA--------------------------------------------------------
--------------------------------------------------------------------------------
--------------------------------------------------------------------------------
--------------------------------------------------------------------------------
--------------------------------------------------------------------------------
--
>Cpo_scaffold35426_69784-70728
GAAGAGATATGTCCTGAAGTTGAAGGTGAAGCCACTCAGTCCTGCCTCTGGGCTTCAGCCATCAATGTCAGAGACAAGTA
TATTTATGTGACACAGCCCAGGCAAAATAGAATACTGATTATTGATATCCAGACCCAGAAAGTCATGCAG---GTATGTA
TCAAGGGAGAAATGTGTTCATGGACCATAAAAAAGTGTTGATATGCCAACA---AATTAGGTTCCTGCCCCGTGGAGCTG
CTCCCTCTGCTAATACATTGGGCATGTACTGTTTTGACCCATCAGGTAACTCCAAGTCAGTGACCAGGCTATGATCTCAG
TGTAAGAAAAACTGTT--TTATGGCCTAGATAGTCTTTGCACTGTGCCTGGAGCCAAGTTTGCAGCTATCTCGATTCAAA
AATATGTCTAACCTACACTTGCTGAGCATAATCTTAGTTACAGCCAGGTTGAAACACATGCCATTGCCCAGGCATAACTC
AAGCAGAAACTGG---TACAAGAGAGGTGGTACTATCACCTAACTTGGAGGTCTTCTAGAGAAGGCTTGCTAGACACCTG
GCTGGAGTCATCTGGCCTCGGTCTTCTTTCCTGCCATGGGCAGGGG-------------ATCCTTTGTGGTTCCTTCTGA
CTCTACAGTCTATGAATCTATGG---TTCCTGTTCTACTGACTACAAGTTCTAATGTGAGACGAGTGCTCA---------
--------CACTACTTTCCTTGCCAAAGCAGAGGAAGTGGTACTTTATAGCCATATTTCTAG---------CAATGTTTG
CAAGTCAATATTACCATA----------------------------AATCTGCATGAGCC-----ACCTTTAATTTGCTA
AATAAAATTAAACA--CTTA------TATTTCTGTTCTTTGAAG---TCTATGGATGTTGATCCCCTACCTACCAAATTA
CATTATGATAAGTCACATGACCAAGTCTGGGTCCTTAGCTGGGGTGATATGCAGAAGTCTCATCCAACGCTGCAGGTGAG
TT
>Gga_scaffold21841
GAAGAGATATGTCCTGAAGTTGAAGGTGAAGCCACTCAGTCCTGCCTCTGGGCTTCAGCCATCAATGTCAGAGACAAGTA
TATTTATGTGACGCAGCCCAGGCAAAATAGAATACTGATTATTGATATCCAGACCCAGAAAGTCATGCAG---GTATGTA
TCAAGGGAGAAATGTGTTCATGGACCATAAAAAAGTGTTGATATGCCAACA---AATTAGGTTCCTGCCCCGTGGAGCTG
CTGACTCTGCTAATAAATTGGGCGTGTACTGTTTTGACCCATCAGGTAACTCCAAGTCAGTGACCAGGCTATGATCTCAG
TGTAAGAAAAACTGTT--TTATGGCCTAGATAGTCTTTGCACTGTGCCTGGAGCCAAGTTTGCAGCTATCTCGATTCAAA
AATATGTCTAACCTACACTTGCTGAGCGTAATCTTAGTTACAGCCAGATTGAAACACATGCCATTGCCCAGGCATAACTC
AAGCAGAAACTGG---TACAAGAGAGGTGGTACTATCACCTAACTTGGAGGTCTTCTGGAGGAGGCTTGCTAGACACCTG
GCTGGGGTCATCTGACCTCGGTCCTCTTTCCTGCCAGGGGCAGGGG-------------ATCCTTTGTGGTCCCTTCTGA
CTCTACAATCTATGAATCTATGG---TTCCTGCTCTACTGACTACAAGTTTTAGTGTGAGACGAGTGCTCA---------
--------TACTACTTTCCTTGCCAAAGCAGAGGAAGTGGTACTTTATGGCCATGTTTCTAG---------CAATGTTTG
CAAGTCAATATTACCATA----------------------------AATCTGCATGAGCC-----ACCTTTAATTTGCTA
AATAAAATTAAACA--CTTA------TATTTCTGTTCTTTGAAG---TCTGTGGATGTTGATCCCCTACCTACCAAATTA
CATTATGATAAGTCACATGACCAAGTCTGGGTCCTTAGCTGGGGTGATATGCAGAAGTCTCATCCAACACTGCAGGTAAG
TT
>Ami_JH731145_355472-356412
GAAGAGATATGTCCTGAAGTTGAAGGTGAAGCCACTCAGTCCTGCCTCTGGGCTTCAGCCGTCAATGTCAGAGACAAGTA
TATTTATGTGACGCAGCCCAGGCAAAATAGAATACTGATTATTGATATCCAGACCCAGAAAGTCATGCAG---GTATGCA
TCAAGCGAGAAATGTGTTCATGGACCATAAAAAAGTGATGATATGCCAACA---AATTAGGTTCCTGCTCAGTGGAGCTG
CTGACTCTGCTAATAAGTTGGGCATGTACTGTTTTCACCCATCAGGTAACTCCAAGTCAGTGACCAGGCTATGACCTCAG
TGTAAGAAAACCTGTT--TTATGGCCTAGATAGTCTTTGCACAGTGTCTGGAGCCAAGTTTGCAGCTATCTAGATTCAAA
AATATGTCTAACCTACATTTGCTGAGCCTAATCTTAGTTACAGCCAGGTTGAAACACATGCCATTACCCAGGCATAACTC
AAGTTGAAACTGG---TC--AGAGAGGTGGTGCTATCACCTAACTTGGAGGTCTTCAAGAGGAGGCTAGATAGTCACCTG
GCTGGGGTCATCTGACCTTGGTCCTCTTTCCTGCCAGGGGCAGGGG-------------ATCCTTTGTGGTCCCTTCCGA
CTCCACAATCTATGACTCTATGG---TCCCTATTCTACTGACTATAAGTTCTAGTGTGAGACGAGCGCTCA---------
--------CGCTCCTTTCCTTGCCAAAGCAGAGGAAGTGGTACTTCATGGCCATGTCTCTAG---------CGATGTTTG
CAAGTCAATGTTACCATA----------------------------AATCTGCATGAGCC-----ACCTTTAATTTGCTG
AATAAAATTAAACA--CTTC------TATTTCTGTTCTTTGAAG---TCTGTGGATGTTGATCCCCTACCTACCAAATTA
CATTATGATAAGTCACATGACCAAGTCTGGGTCCTTAGCTGGGGTGATATGCAGAAGTCTCATCCAACACTGCAGGTAAG
TT
>Asi_AVPB01078359_17859-18803
GAAGAGATATGTCCTGAAGTTGAAGGTGAAGCCACTCAGTCCTGCCTCTGGGCTTCAGCCATCAATGTCAGAGACAAGTA
TATTTATGTGACACAGCCCAGGCAAAATAGAATACTGATTATTGATATCCAGACCCAGAAAGTCATGCAG---GTATGTA
TCAAGGGAGAAATGTGTTCATGGACCATAAAAAAGTGATGATATGCCAAAA---AATTAGGTTCCTGCCCAGTGGAGCTG
CTGACTCTGCTAATAAATTGGGCAGGTACTGTTTTGACCCATCAGGTAACTCCAAGTCAGTGACCAGGCTATGACCTCAG
TGTAAGAAAAACTGTT--TTATGGCCTAGATAGTCTTTGCACAGTGTCTGGAGCCAAGTTTGCAGCTATCTAGATTCAAA
AATATGTCCAACCTACACTTGCTGAGCCTAATCTTAGTTTCAGCCAGGTTGAAACACATGCCATTGCCCAGGCATAACTC
AAGTTGAAACTGG---TCCAAGAGAGGTGGTACTATCACCTAATTTGGAGGTCTTCAAGAGGAGGCTTGACAGTCACCTG
GCTGGGGTCATCTGACCTCGGTCCTCTTTCCTGCCAGGGGCAGGGG-------------ATCCTTTGTGGTCCCTTCCGA
CTCCACAATCTATGAATCTATGG---TCCCTGTTCTACTGACTATAAGTTCTAGTGTGAGACGAGTGCTCA---------
--------CGCTCCTTTCCTTGCCAAAGCAGAGGAAGTGGTACTTCATGGCCATGTCTCTAG---------CGATGTTTG
CAAGTCAATGTTACCATA----------------------------AATCTGCATGAGCC-----ACCTTTAATTTGCTG
AATAAAATTAAACA--CTTC------TATTTCTGTTCTTTGAAG---TCTGTGGATGTTGATCCCCTACCTACCAAATTA
CATTATGATAAGTCACATGACCAAGTCTGGGTCCTTAGCTGGGGTGATATGCAGAAGTCTCATCCAACACTGCAGGTAAG
TT
>Gallus_GB_chr13_15739435-15740263
```

GAGGAGATCTGTCCCAGAGTGGAAGGGGAAGGCGCTCAGTCCTGCCTCTGGGCCTCCGCAGTCAATGTCCGAGACAAATA
TATCTATGTGACACAGCCAAAGCAGAACAGAGTAGTGGTCATCGACATCCAGACACAGAAAGCTGTGCAG---GTACCT-
ACAAGGGGAACTCTTCATTCACTGATGGCAAAAGACT-TTGCTATGGGAAAACAAAAGTAGGTTTCTGCCTGGTGGATCT
GACATCACTCTGCGTGA------TGTTA---TTTTCACCCATGGGGTCACGCTGTGTTATTCGCTGTTCTGAAACCATAC
TGCCAGGAAAAGAATCCCTTATGAGCTGCTTATCATCATCTCAGGGCTGTGTCTGCAGTTGGGTTTGCAGCTACCCTGGT
AAAATGGTGCTCCT-----------------TCACCATTCTGAGCAGGTCAGGAAGAACGAGCTGATGCAGCTGTGGCTG
GGGCAGAAGTTGG---

--------------------------CCCCTGAGCTGCTGGCTGCGAGGAGCAGAGCAGGACGTGCAGCCAGAGAACGGA
GCTGAGCAGCCAGCTTTTCTATTGCTGGAGCTGTTGCTGCTCCCCAGATTGATTTGAAGGTAACCATACAAAGCTTACAA
ATGAGTGTAGCAGTGGTACAGGGGAATGCCAGCAGTGAAGTAATACAACTGCCATGTGCTTAGGGACATTTGTGTCACTA
CATAAACATGTATCATCTCC------TTTTTTTCTTTTTCCAAG---TCTGTGGATGTGGACCCATTGCCAACCAAACTG
CATTACGACAAATCCCATGACCAGGTGTGGGTGCTGAGCTGGGGTGATATGCGGCGGTCATCGCCAACGCTGCAGGTGAG
TC
>Turkey_GB_chr15_16263546-16264353
GAGGAGATCTGTCCCAGAGTGGAAGGGGAAGGCACTCAGTCCTGCCTCTGGGCCTCCGCAGTCAATGTCCGAGACAAATA
CATCTATGTGACACAGCCTAAGCAGAACAGAGTAATGGTCATCGACATCCAGACACAGAAAGCTGTGCAG---GTACCT-
ACAAAGCTAACTCTTTATTCACTGATGGCAAAAGACT-TTGCTATGGGAAAACAAAAGTAGGTTTCTGTCCAGGGGATCT
GACGTCACTCTGCGTGA------TGCT-GCACCTGTGTTATTTTCACCCATGGACCACAGTGTTATTCTGAAACCACAC
TGCCGGGAAAAGAATCCCTTATGAGCTGCTTATCATCATCTCAGGGCTGCGTCTGCAGTTGGGTTTGCAGCTACCCTGGT
AAAATGGTGCTCCT-----------------TCACCATTCTGAGCAGGTCAGGAAGAATGAGCTGATGCAGGTGGGGCTG
GGGCA---GTTGA---

--------------------------GCCCTGAGTTGCTGGTTGCAAGGAACAGAGCAAGATGTGCAGCCAGAGAATGGA
GCCGAGCAGCCAGCTTTTCTATTGCTGGAGCTGCTGCTGATCCCCAGATTGATTTGAAGGTAACTGTACAAAGCTTACAA
ATGAGTGTAGCAGTGGTACATGGGAATGTCAGCAGTGAGCTAATATAACTGCCATGTGCCTAGGGACATTTGTGTCACTA
TGTAAACATGTATCATCTCCCATTTTATTTTATTTTTTTTTAAG---TCTGTGGACGTGGACCCTTTGCCAACCAAACTG
CATTATGACAAATCCCACGACCAGGTGTGGGTGTTGAGCTGGGGTGATATGCGGCGATCGTCACCAACACTACAGGTGAG
TC

CG77

>CR1-2_Crp

---TGGAGTACTGCGTCCAGTTCTGGGCGCCGCACTTCAAGAGGG--------------------------------ATG
TGGACAACATGGAGAGGGTCCAGAGGAGGGCCACTCGCATGATCAGGGGGCAGCAGGGCAGGCCCTACGAGGAGAGGCTA
CGGGACCTGAACCTGTTCAGCCTCCACAAGAGAAGGCTGAGGGGGGATCTAGTGGCCTGTTACAAACTAGTCAGGGGGGA
CCAGCAGGCACTGGGGGAGTCCCTGTTCCCCCGAGCACTGCCAGGAGTGACCAGGAAATAACGGTCACAAGCTGGCAGAG
GGTAGATTCAGATTAGACATCAGGAGGCGCTACTTCACTGTCAGGGCGGCTAGGATCTGGAACCAACTTCCAAGCGAAGT
GGTGCTGGCTCCTACCCTGGGGGTCTTTAAGAGGAGGCTTGACGAWCACCTTGCTGGGGTCGTTTGACCCCAGTACTCTT
TCCTGCCATGGCAGGGGGTCGGACTTGATGATCTGCTCAGGTCCCTTCCGACCCTACTAACTATGAAACTATGAAACTAT
G---

>Cpo_scaffold36431_7453-8526_RC
TTTCTTCCTTCTTCTCCAG---ATAAGTTTGATGCATACATGTAAACCTTCAACAAGGCCTTTCAGAGCATCTAGTTCTC
ACTTTTTCTTCCTGTTGGTACTGTTGATTGGGCTCATGCTGGCTTTTATCCCCTTGGGACTCAGCATGGCACA--GTAAG
TGATGATTT-CATATTTATAAAGTTTCATTTATTTATTTGCTTGCTT-----GAAGCTTGTTGGGGGATGGACTCATTTT
TAAATGATACATTAGAA----AATATACCTGCATACAATGCAAAAGGAAATAAATACTTATTGACAGTGAGCTTTTCTGT
TTTCAAAAATGCATTGTAATTCAAACCAAAGGAACTTATTATATGCTCAATA-GGATTGGATTTTTACGTTC-----ATG
TGGCTAGCATTGAGGAGGCCCAGACGAGGGCCACTCACATGGTCAAGGGGCAGCAGGGCAGGCCCTATGAGGGGAGGCTA
AAGGGCCTAAACCTGTTCAGCCTCCACAAGAGAAGGCTGAGAGGGGATCTGGTGGCCATTGACAAACTCACCAGGGGGGA
CCAGCGAGAATTGGGGGAGGCTCTGTTACCCTGGACACCACCCAGGGTTACTAGGA-ATAATGGCCACAAATTGTTGGAG
AGAAGGGTCAGGCTGGACATCAGTATTCATTACTTTACAGTTAGGGCTGCCAGGCTCTGGAATGGGCTCCCAAGGGAGGT
AGTGCTCTTTCCTACCTTGGGGGCCTTCAAGAGGAGGCTGGACAGATATTTGGCTGGGGATATATGACTACGGCACTCAT
TCCTACCT-------GGGTCGGACCTGATGGTCTGTTTAGGTCCCTTCCAACCCTAAGCGCTATGAAACTAT-AAACTAT
G---AAATGATTGCCTCTTTATTTTTCAGTACAGCATCTCTTTTCTGTTTGGATTTTTTA-------ACAG---GATCCC
CTCCTCTAAAGCATGTGGGCCATTTATTAATTTCAATACTTCGTGGGCTGTTATTCCAGAGACAGTAGATGGACTTCCAA
GAGGACTTCAGAAGTTCCTTTATGGGATGGCATCAGAAGCCTTTGCAGTGCCTTTCTTCATGGTTATGTG---GTGAGTG
>Gga_scaffold3464
TCTTTTCCTTCTTCTCCAG---ATAAGTTTGATGCATACATGTAAACCTTCAACAAGGCCTTTCAGAGCATCTAGTTCTC
ATTTTTTCTTCCTGTTGGTGCTGTTGATTGGGCTCATGCTGGCTTTTATCCCCTTGGGACTCAGCATGGCACA--GTAAG
TGATGATTT-CATATTTATAAAGTTTCACTTATTTATTTGCTTGGTT-----GAAGCTTGTTGGGGGGTGGACTCATTTT
TAAATGATACATTAGAA----AATATACCTACATACAATGCAAAAGGAAGTAAATACTT---GACAGTGAGCTTTTCTGT
TTTCAAAAATGCGTTGTAATTCAAACCAAAGGAACTTATTATATGCTCAATA-AGATTGGATTTTTATGTTG-----ATG
TGGCTAGCATTGAGAGGGTCCAGAGGAGGGCCACTTGCATGGTCAAGGGGCAGTGGGGCAGGCCCTATGAGGGGAGGCTA
AAGGGCCTAAACCTGTTCAGCCTCCACAAGAGAAGGCTGAGAGGGGATCTGGTGGCCATTGACACACTCACCAGGGGGGA
CCAGCAAGAATTGGGGGAGGCTCTGTTACCCCGAACACTACCCAGGGTTACTAGGA-ATAATGGCCACAAATTGTTAGAG
AGAAGGTTCAGGCTGGACATCAGTAGACATTACTTTACAGTTAGGGCTGCCAGGCTCTGGAATTGGCTCCCAAGGGAGGT
AGTGCTCTTTCCTACCTTGGGGGCCTTCAAGTGGAGGCTGG----------------GGAGATATGACTACGGCACTCAT
TCCTACCT-------GGGTCAGACCTGATGGTCTGTTTAGGTCCCTTCCAACCCTAAGC----------------ACTAT
G---AAACTATTGCCTCTTTATTTTTCAGTACAGCATCTCTTTTCTGTTTGGATTTTTTA-------ACAG---GATCCC
CTCCTCTAAAGCATGTGGGCCATTTATTAATTTCAATACTTCGTGGGCTGTTATTCCAGAGACAGTAGATGGACTTCCAA
CAGGACTTCAGAAGTTCCTTTATGGGATGGCATCAGAAGCCTTTGCAGTGCCTTTCTTCATGGTTATGTG---GTGAGTG
>Ami_JH731451_5453-6528_RC
TCTGTTCCTTCTGCTCCAG---ATAAGTTTGATGCATACATGTAAACCTTCAACAAGGCCTTTCAGAGCATCTAGTTCTC

```
ACTTTTTCTTCCTGTTGGTGCTGTTGATTGGGCTCATGCTGGCTTTTATCCCCTTGGGACTCAGCATGGCTCA--GTAAG
TGATGATTT-CATATTTATAAAGTTTTATTTATTTATTTGCTTGCTT-----GAAATTTGTTGGAGGGTGGACTCATTTT
TAAATGTTACATTAGAAG-AAAATACACCTACATACAATGCAAAAGGAAATAAATACTTATTGACAATGAGCTTTTCTAT
TTTCAAAAATGCATTGTAATTCAAACCAAAGGAACTTACTATGTGCTCAGTA-AGATTGGATTTCTAGGTTG-----ATG
CAGCTAGCAATGAGAGGGTCCAGAGGAGGGCCACTTGCATGGTCAAGGGGCAGCAGGGCGGGCCTTATGAGGAGAGGCTG
AAGGGCCTAAACCTATTCAGCCTCCACAAGAGAAGGCTGAGAGGGGATCTGGTGGCCATTGACAAACTCACT-GGGGGGA
CCAGCAAGAATTGGGGGAGGCTCTGTTCCTCCGGGCANCACCCAGGGTTACTAGGA-ATAATGGCCACAAATTGTTAGAG
AGAAGGTTCAGGCTGGACATCAGCGGACATTACTTTACAGTTAGGGCTGCCAGGCTCTGGAATGGGCTCCCAAGGGAGGT
AGTGCTCTTTCCTACCTTGGGGACCTTCAAGAGGAGGCTGGACAGACATTTGGCTGGGGAGATATGACTGCGGCACTCGT
TCCTGCCT-------GGGTCGGACCTGATGGTCTGTTTAGGTCCCTTCCAACCCTAAGCACTATGAAACTATAA-ACTAT
G---AAACTATTGCCTCTTTATTTTTCAGTACAGCCTCTCTCTTCTGTTTGGATTTTTTA-------ACAG---GATCCC
CTCCTCTAAAGCATGTGGGCCATTTATTAATTTCAATACTTCGTGGGCTGTTATTCCAGATACAGTAGATGGACTTCCAA
CAGGACTTCAGAAGTTTCTTTATGGGATAGCATCAGAAGCCTTTGCAGTGCCTTTCTTCATAGTTATGTG---GTGAGTG
>Asi_AVPB01125999_3952-2885
TCTCTTCCTTCTTCTCCAG---ATAAGTTTGATGCATACATGTAAACCTTCAACAAGGCCTTTCAGAGCATCTAGTTCTC
ACTTTTTCTTCCTGTTGGTGCTGTTGATTGGGCTCATGCTGGCTTTTATCCCCTTGGGACTCAGCATGGCTCA--GTAAG
TGATGATTT-CATATTTATAAAGTTTTATTTTATTTATTTATTTGCTTGCTTGAAATTTGTTGAAGGGTGGACTCATTTT
TAAATGATACATTAGAAG-AAAATACACCTACATACAATGCAAAAGGAAATAAATACTTATTGACAATGAGCTTTTCTGT
TTTCAAAAATGCATTGTAATTCAAACCAAAGGAACTTA---TGTGCTCAGTA-AGACTGGGTTTTTACGTTG-----ATG
CAGCTAGCAATGAGAGGGTCCAGAGGAGGGCCACTTGCATGGTCAAGGGGCACCAGGGCAGGCCTTATGAGGAGAGGCTG
AAGGGCCTAAACCTATTCAGCCTCCACAAGAGAAGGCTGAGAGGGGATCTGGTGGCCATTGACAAACTCACC-GGGGGGA
CCAGCAAGAATTGGGGGAGGCTCTGTTCCCCCGGGCACCACCCGGGGTTACTAGGA-ATAATGGCCACAAATTGTTAGAG
AGAAGGTTCAGGCTGGACATC----------ACTTTACAGTTAGGGCTGCCAGGCTCTGGAATGGGCTCCCAAGGGAGGT
AGTGCTCTTTCCTACCTTGGGGGCCTTCAAGAGGAGGCTGGACAGACATTTGGCTGGGGAAATATGACTGCGGCACTTGT
TCCTGCCT-------GGGTCGGACCTGATGGTCTGTTTAGGTCCCTTCCAGCCCTAAGCACTATGAAACTATAA-ACTAT
G---AAACTATTGCCTCTTTGTTTTTCAGTACAGCCTCTCTCTTCTGTTTGGATTTTTTA-------ACAG---GATCCC
CTCCTCTAAAGCATGTGGGCCATTTATTAATTTCAATACTTCGTGGGCTGTTATTCCAGATACAGTAGATGGACTTCCAA
CAGGACTTCAGAAGTTCCTTTATGGGATAGCATCAGAAGCCTTTGCAGTGCCTTTCTTCATAGTTATGTG---GTGAGTG
>Gallus_GB_chr14_8476745-8477338_RC
GCTCATTTTTTCACTCCAG---ATCAGTTTGATACACACGCGTAAACCTGCAAGTAGACCTATCAGAGCATCAAGTTCCA
ATTTTTTCTTCTTGGCGGTGCTGTTGATTGGGCTCATCTTAGCTTTTGTTCCTTTGGGAGTCAGCATAGCACT--GTAAG
TGACAATTT-AACCTCTGTGAAGTTGGCATTTGCTTTAAGGTTATCA-----GTCTCCTAGAAACAGATGAA-TAACTTT
AAAATAATAAAATAGAAT-ACTATATACCTGCTTATAAT--AAAATCAAATTATTATTT------------CATTTATAT
AAACAAAAAAATACTGTTTTTAAGATGAGGTACACTT-TTACCCTATCAGCA-AGATAGTGCTGGCTTGTGT--------
--------------------------------------------------------------------------------
--------------------------------------------------------------------------------
--------------------------------------------------------------------------------
--------------------------------------------------------------------------------
--------------------------------------------------------------------------------
--------------------------------------------------------------------------------
---TGGATTATTTTCTCTTTACATTTTGGTGTAGCATCTCATTTATCTTCTGATTTTTTTTTTTTTCCACAG---TATCTC
CTCTTCCAAGGCATGTGGGCCATTCAGGAATTTTAATACTTCCTGGGCCATTGTTCCATATACAATACTTGAGTTTCCAA
TAGGTCTGCAGAAATTCCTTTATGGCATAGCATCAGAAGCCTTTGCAGTGCCTTTTTTTGTGATTGCCTG---GTAAGTG
>Taeniopygia_GB_chr14_16052911-16053492
ACTCACCTTCCCTCTTCAG---ATTGTTTTGATACACACACGCAAACCTGCAGCAAGGCCTATAAGGGCATCAAGCTCCA
ACTTTTTCTTCCTGGTGGTGCTGTTGATTGGGCTCCTCTTGGCTTTTATCCCTCTGGGATTCAGCATAGCACG--GTAGG
TGACAATTTTAAGTTCTGTGAAGTTGACACTTGTTCTAAGCTTGTCA-----GTTCTGTAAAAGCAGACACA-ACACTTT
AGAATGATTAATGGAATAAAATATATAGGGTAT--AACACACAAACGTATAGCTTAGTT------------CATATA---
--AACAAAAATCACAGTTTTATAGACATAGGACACTT-TTACTGCATCACCAGACTGGTGCTGGGCTTGTGC--------
--------------------------------------------------------------------------------
--------------------------------------------------------------------------------
--------------------------------------------------------------------------------
--------------------------------------------------------------------------------
--------------------------------------------------------------------------------
--------------------------------------------------------------------------------
---TGGATTATTTTATCTTTACATTTGGGTGTAACATCTTTCT---GTTCTGCTTCTTTG-------ACAG---TATCCC
CTCTTCCAAGGCATGTGGGCCGTTCAGGAATTATAACACTTCGTGGGAAGTTATTCCAGATACAATACTTCAGTTTCCAA
CAGGTCTGCAGCAGATCCTTTTTGGCATTGCATCAGAAGCTTTTGCAGTGCCTTTTTTTGTGATCCTTTG---GTGAGTA
>Turkey_GB_chr16_8800878-8801465_RC
GCTCATTTTTTCTCTCCAG---ATCAGTTTGATACACACATGTAAACCTGCAAGTAGGCCTATCAGAGCATCAAGTTCCA
ATTTTTTCTTCTTGGTGGTGCTGTTGATTGGGCTCATCTTAGCTTTCATTCCTTTGGGAGTCAGCATAGCACA--GTAAG
TAACAATTT-AACTTCTGTGAAGTTGGCATTTGCTTTAAGGTTGTCA-----GTTCCCTAGAAACAGATTAATAATTTAT
AAATAATAAAATAGAATACCGTGTACCTGCCTATAATAAAATCAAACTTTTATTTAGTT------------TATATA---
--AACAAAAAAATACCATTTTTAAGATGAGGTACGCTTTTACCCTATCAACAAGATAGTGCTGCCTTGCGTT--------
--------------------------------------------------------------------------------
--------------------------------------------------------------------------------
--------------------------------------------------------------------------------
--------------------------------------------------------------------------------
--------------------------------------------------------------------------------
--------------------------------------------------------------------------------
----GGATTATTTTATCTTTACATTTTGATGTAACATCTCATTTATCTTCTGTTTTTTTTTC------ACAG---TATCTC
TTCTTCTAAGGCATGTGGGCCATTCAGGAATTTTAACACTTCATGGGCTGTTGTTCCAGATACAATACTTGGGTTTCCAA
TAGGTCTGCAGAAATTCCTTTTTGGCATAGCATCAGAAGCTTTTGCAGTGCCTTTTTTTCTGGTTGCCTG---GTAAGTA
```

CG87

```
>CR1-6C_Ami_RC
--------------------------------------------------------------------------------
--------------------------------------------------------------------------------
```


--TAGATTCATAGATTCATAGATG-TAGGGTCGGAA
GGGACCT-AGCAGATC-TTCAAGTCCGACCCCCTGCCCTGGGCAGGAGAG--AATACTGGGCTCAWATG---------AC
CC--

>Cro_scaffold38216_61906-62955_RC
ATTTTTAG---GAAAATGAGAGGTTGAAGCAGGAGATATTTGAGAAGAGCAGCCGGATTGAGGAGCAGAACGAGAAGATC
AGTGAACTGATTGAACGGAATCAGAG---GTGAGCTGTAGGAAAGATAAGGCTATTGTCCACTATAGCTGGACCTATCAA
AATTGTGCTTTTGGTTGGCTCTGTGGGGAAGGAGAA---AAAACTCACTGAAAA-TAAAATGTTGGCTCCCTCTAGGAAG
CCAGCAGCCCTTGTGGTCACTGTGTCCAGCTGTGCAGCCTACTGGGAGGAGATGGGGAGTGGGCTGTGTGCCAGATGCCC
CCT-TGCGGCAAGTCAGTGGGCAG-------------------------------CTGCTTTGCCTTCAGTAGACCCCTA
GCTGTAGACCTCAGACCTAAAGACCTGGTTGCTGAATGGCACATCTGCTGTTGACTTAACTGAAAGGAAGG----TCCTC
CAATACGTAATGCTCTTGTCCATCCTTCTGTAACCTCTTCCACTTGACTGGAGTAGGTACTATCACTGCTCATGCAAATC
CCATCAAGGGCATTCAGAGAAGGATAGTCTCCCAAGGAAGGCAGTGAAAGCT---TTATTAC--TTGGGCTGTCAAACAT
CTGATTGTGCTTCAGGAGCACTGCTCCAATGG--GGAACAATGGAGTAGATGTCCTAATT---GAGCTCATCTGTCCCTA
ACTGCAGATTCATCATACTTCTTCCAAAGTCCAGATGCATTTG---TATTTAAGACATTTCATAGATA-CAGGGTTGGAA
GGGACCTAAGTAAATCTATCAAGTCCAACCCCCTGCCCTGGGCAGGAGAGAGAATAGAGGAACACAAAATTATTCTGGAA
CTTACTCTTGTGAGGGGGAGGCTTTGTAA-------TATCACCTTTCCTGTGTCAGGGGCTTT-----------------
---CATTTCAAATCCTCTTCTT
GCGTATGTGCTCATG-GCACTCATTTCGCCTTCTCTTCCAG---GTGTGTTGAACAGAGTAACCTGCTGATGGAGAAAAG
AAACAGCTCACTGCAAACAACAAATGAGAACACACAGGCAAGAGTGTTGCATGCAGAACAGGAGAAG---GTA
>gavGan_scaffold25408
ATTTTTAG---GAAAATGAGAGGTTGAAGCAGGAGATATTTGAGAAGAGCAGCCGGATTGAGGAGCAGAACGAGAAGATC
AGTGAACTGATTGAACGGAATCAGAG---GTGAGCTGTAGGAAAGATAAGGCTATTGTCCACTATAGGTGGACCTATCAA
AATTGTGCTTTTGGTGGGCTCCGTGGGGGAGGAGAA---AAAACTCACTGAAAA-TAAAATGTTGGCTCCCTCTAGGAAG
CCAGCAGCCCTTGTGGTCACTGTGTCCAGCTGTGCAGTCTACTGGGAGGAGATGGGGAGTGGGCTGTGTGCCAGATGCCC
CCT-TGCAGCAAGTCAGTGGGCAG-------------------------------CTGCTTTGCCTTCAGTAGACCCCTA
GCTGTAGACCTCAGACCAAAAGACCTGGTTGCTGAATGGCACATCTGCTGTTGACTTAACTGAAAGGAAGG----TCCTC
CAATACGTAATGCTCTTGTCCATCCTTCTGTAACCTCTTCCACTCGACTGGAGTAGGTACCATCAGTGTTCATGCAAATC
CCATCAAGGGCATTCAGAGAAGGATAGTCTCCCAAGGAAGGCAGTGAAAGCT---TTATTAC--TTGGGCTGTCAAACAT
CTGATTGTGCTTCAGGAACACTGCTGCAATGG--GGAACGATGGAGTAGATGTCCTAAAT---GAGCTCATCTGTCCCTA
ACTGCAGATTCATCATACTCCTTCCAAAGCCCAGATGCATTTG---TATTTAAGACATTTCATAGATA-CAGAGTTGGAA
GGGAC-------------TCAAGTCCAACCCCCTGCCCTGGGCAGGAGAGAGAATAGTGGAACACAAAATTATTCTGGAA
CTTACTCTTGTGAGGAGGAGGCTTTGTAA-------TATCACCTTTCCTGTGTCAGGGGCTTT-----------------
---CATTTCAAATCCTCTTCTT
GTGTATGTGCTCATG-GCACTCATTTCGCCTTCTCTTCCAG---GTGTGTTGAACAGAGCAACCTGCTGATGGAAAAAAG
AAACAGCTCACTGCAAACAACAAATGAGAACACACAGGCAAGAGTGTTGCATGCAGAACAGGAGAAG---GTA
>Ami_JH734874_259042-260088
ATTTTTAG---GAAAATGAGAGGTTGAAGCAGGAGCTATTTGAGAAGAGCAACCGGATTGAGGAGCAGAACGAAAAGATC
AGTGAACTGATTGCACGGAATCAGAG---GTGAGCTGTAGGAAAGATATGGCTATCGTCCACTATAGGTAGACCTATCGA
AATTGTGCTTTTGGTTGGCTCCGTGGGGGAGGAGAAAAGAAAACTCACTGAAAATTAAAATGCTGGCTCCCTCTAGGAAG
CCAGCAGCCCTTGTGGTCA----------GTGTGCAGCCTGCTGGGAGGAGATGGGGAGTGGGCTGTGTGCCAGATGCCT
CCT-TGCGGCAAGTCAATGGGCAG-------------------------------CTGCTTCGACTTCAGTAGACCCCTA
GCTGTAGACCTCAGACCTAAAGATCTGGTTGCTGAATGGCACATCTGCTGTTGACTTAACTGAAAGGAAGG----TCCTC
CAGTATGTAATGCTCTTGTCCATCCTTCTGTAACCTCTTCCACTCGACTGGAGTAGGTACTATCAGTGTTCATGCAAATC
CCATTAAGGGCCTTCAGAGAAGATTAGTCTCCCAAGGAAGGTAGTGAAAGCT---TTATTGC--TTGGGCTGTCAAACAT
CTAATTGTGCTTCAGGAGCACTGCTGCAATGG--GGAACGATGGAGCAGATGTCCTAATT---GAGCTCATCCATCCCTA
ACTACAGATTCATCATACTCCTTCCAAAGTCCAGGTGCATTTG---TATTTAAGACATTTCATAGATG-TAGGGTTGGAA
GGGACCTAAGCAGATCA-TCAAGTCCGACCCCCTGCCCTGGGCAG--GAGAGAATACTGGAACACAAAATTATTCTGGAA
CTTACTCCTTTGAGGGGGAGGCTTTGTAATAAAGACTATCATCTTTTCTGTGTCAGGGGCTTT-----------------
---CATTTCAAATCCTCTTCTC
-TGTATGTGCTCATG-GCACTCATTTCGCCTTCTCTTTCAG---GTGTGTTGAACAGAGCAACCTGCTGATGGAGAAAAG
AAACAACTCACTGCAAACAACAAATGAGAACACACAGGCAAGAGTGTTGCATGCAGAACAGGAGAAG---GTA
>Asi_AVPB01134430_2199-3254
ATTTTTAG---GAAAATGAGAGGTTGAAGCAGGAGATATTTGAGAAGAGCAACCGGATTGAGGAGCAGAACGAGAAGATC
AGTGAACTGATTGCACGGAATCAGAG---GTGAGCTGTAGGAAAGATATGGCTATCGTCCACTATAGGTGGACCTATCGA
AATTGTGCTTTTGGTTGGCTCCGTGGGGGAGGAGAAAAGAAAACTCACTGAAAATTAAAATGCTGGCTCCCTCTAGGAAG
CCAGCAGCCTTTGTGGTCGCTG--CCCAGCTGTGCAGCCTGCTGGGAGGAGATGGGGAGTGGGCTGTGTGCCAGATGCCC
CCT-TGCGGCAAGTCAATGGGCAG-------------------------------CTGCTTCGACTTCAGTAGACCCCTA
GCTGTAGACCTCAGACCTAAAGATCTGGTTGCTGAATGGCACATCTGCTGTTGACTTAACTGAAAGGAAGG----TCCTC
CAGTATATAATGCTCTTGTCCATCCTTCTGTAACCTCTTCCACTCGACTGGAGTAGGTACTATCAGTGTTCATGCAAATC
CCATTAAGGGCCTTCAGAGAAGATTAGTCTCCCAAGGAAGGTAGTGAAAGCT---TTATTGC--TCGGGCTGTCAAACAT
CTAATTGTGCTTCAGGAGCACTGCTGCAATGG--GGAACCATGGAGCAGATGTCCTAATT---GAGCTCATCCATCCCTA
ACTACAGATTCATCATACTCCTTCCAAAGTCCAGATGCATTTG---TATTTAAGACATTTCATAGATG-TAGGGTTGGAA
GGGACCTAAGTAGATCA-TCAAGTCCGACCCCCTGCCCTGGGCAG--GAGAGAATACTGGAACACAAAATTATTCTGGAA
CGTACTCCTTTGAGGGGGAGGCTTTGTAATAAAGACTATCATCTTTTCTGTGTCAGGGGCTTT-----------------
---CATTTCAAATCCTCTTTTT
GTGTATGTGCTCATG-GCACTCATTTCGCCTTCTCTTTCAG---GTGTGTTGAACAGAGCAACCTGCTGATGGAGAAAAG
AAACAACTCACTGCAAACAACAAATGAGAACACACAGGCAAGAGTGTTGCATGCAGAACAGGAGAAG---GTA
>Gallus_GB_chr17_1516780-1517678

```
TTCTTTAG---GAGAATGAGAGACTGAAGCAAGAGATATTTGAGAAGAGCAGTCGAATTGAGGAGCAGAATGAAAAGATC
AGTGAGCTGATAGAGCGGAATCAGAG---GTAAGTGGAAGGTA------TACTGTTGTCTGCACCTGCAGGCTTTCT---
-ATCTTACATTTGCTTAG--------------------------------------------------------------
---------------------------------------------------------------ATGTAACTAGGAGGTGT
CCTATCTCTAGAAT-GGAGAGAAGTTCTGATTTATTTATTATTTTTTGTACTAAAATATTGGTATTTTTTTAATCCCCTT
-----CTGTCTTCACAGGGCATATTTCCAAATGAGATCTTACAGTAAACATGAATATGTAAAAGGGAAAGA----TCTCA
TGCTACTTTCTTTGAGTATGCTTCCTCTGCT---------TCTGGAGGTTACTTTGCTTTTGCTAGCCTTTTAGTAAAGT
GGAAGAAATCATACAGGAAGTGATGTTCCTAGTTCCAGTGAGTTCCAGTGTTGTGTTCCATTAAGTTTACAAAGGGGAAT
AACTTAGTCAAGGAAACAGCAGAAACCAGTGG------------------TTGCCAGGCTATCAAAATTCTTGGCTGTGT
GCTAGGAGTCCTAGAATAGTGGCAGAGAGGTAAAGCACATATC-------------------------------------
----------------------------------------------------------------------TGAACTGATG
TATTCTGTGCTGAAGGCTGGGAACACCCAT------TCCACCCTGT------GGAGGCACTTCTTAGTGCAGGACACGAT
ACAGAATTGTCTGGGACTTCTCAGTAATGAAAGCTGATTTGAAATCCTTTGTCTATCTCATCACTTCTTGCTCCATTTTT
GTATTTATATAAATGCATGTGCCGTCTTCCCCTGTTTACAG---ATATGTCGAACAAAGTAACTTGTTAATGGAGCAGAG
GAACCACTCACTACAAACAACAAACGAAAACACACAGGCAAGAGTGTTGCATGCAGAACAGGAGAAG---GTA
>Turkey_GB_chr19:1361275-1362174_RC
TTCTTTAG---GAGAACGAGAGACTGAAGCAGGAGATATTTGAGAAGAGCAGTCGAATTGAGGAGCAGAATGAAAAGATC
AGTGAGCTGATAGAGCGGAATCAGAG---GTAAGGAGTGGAAG------GTGTCTGCAGCTGCAAAGAGTTTCTATC---
-TTACATTTGCTTAGATG--------------------------------------------------------------
----------------------------------------------------------------TAACTAGGAGGTGTCC
TATCTCTAGAATGGAGAGAAGTTGTAATTTATTTATTTACTTGGTTTTGGTACTAAAATATTGGTGTTTTTTAATCCCCC
TCTGTCT----TTGCAGGGCATATCTCCAAATGAGATCTTACAGTAAACATGAATATGTAAAAGGGAAAGA----TCTCC
TGCCACTTTCTTTGAGCATACTTCCTCTGCT---------TCTGGGGGTTATTTTGCTTTTGCTAGCCTTTTAGTAAAGT
GGATGAAATTGTACAGGAAGTAGTGTTCTTG-TTCCAGTGAGTTCCAGTGTTGCGTTCCATTAAATTTATGAAGGGGAAT
AACTTATTCAAGGAAAAAGCAGAAAGCAGTGG------------------TTACCAGGCTATCAAAAATCTCGACTGTGT
GGTAGGAATCGTAGACTAGTGGCAGAGGCAAAGCACATATCTG-------------------------------------
----------------------------------------------------------------------AACTGACATA
ATCTGTGCTGAAGGCTGGGAACTTCCATT--------GCACCCTGTGGAGGTAAGGCACTTCTT-GGTGCAGGATGTGAT
ACAGAATTGTCTGGGACTTCTCATTAGTGAAAGCTGATTTGAAATGCTTTGTCTGTCTCATCACTTCTTGCTCCGTTTGT
GTATTTATATAAGTG--CTTCTGCCATCTTCCTGTTTACAG---ATATGTTGAACAAAGTAACTTGTTAATGGAGCAGAG
GAACCACTCACTGCAAACAACAAATGAAAACACACAGGCAAGAGTGTTGCATGCAGAACAGGAGAAG---GTA
>Taeniopygia_GB_chr17_1353365-1354175
TGCTTTAG---GAGAATGAGAGGCTGAAGCAGGAGATATTTGAGAAGAGCAGTCGGATTGAGGAGCAGAATGAGAAGATC
AGTGAGTTGATTGAACGCAACCAGAG---GTAAGGGGTGGGAG------CCTCATTGCATTTCCAGACTCTGCAGCT---
-TACACTTGGGCATAACT--------------------------------------------------------------
----------------------------------------------------------------------------GAGG
GGTGTTTGTTAGGAGACAGCCATATTCTGTATATTTTGAATTTTTTTTTCCTAAGGTACTTAAAGTGTAGGATTCCTCCC
ACCCTTT--CTCTGAAGGGCTTATCCCCAAAACTCTGATTTACAGTAAACATGAATGTGTAAAAGGGGAACAAAATCCCT
CATGCTGTCTTTGAGCACATTTGCCCTG----------CTTCTGGGAGTTTCTTTATTTGTGCTGGCTCTTTAGTGAGGT
G-AAGAAATTGTAGGGGACGTGGTGCCCTTTGTG--AAGTTCAGCTGCTACCAAGTTACCAAAA-CTCTTGGAGCATGAG
GCATTTTCAGAGAGGTGAAGCACTTATCCCAG------------------CTGATAAAATCTCTG---CTGTGCCAGGCA
CAGCCCTCCCACCCCTCTGAGATCAGCCTCTTAGCACAGCACA-------------------------------------
----------------------------------------------------------------------TGCTGTGAAA
TTGTCTGGGACTTGTCACGAGTTCAGCAGAGCTCCTGTGCTTTGGAATATCACTTTTCTGTCT-----------------
--------------------------------------------------------------GAGCCCTGCCTCCAAGCC
CTGTGTTTCCAGCAATGCCCACGGAGTCTCCCTGTTCACAG---GTATGTGGAGCAGAGTAACCTGCTGATGGAGCAGAG
GAACCACTCCCTGCAGACAACAAACGAGAACACACAGGCAAGAGTGTTGCATGCAGAGCAGGAGAAG---GTA
```

Additional file 3

>CR1-10B_AMi
CTTGCACCACGGCCGAAAGGGACCT-
AGGGGTAGTGATCGACCATCGCATGAACATGAGCCGGCAGTGCGATGCTGTGGCCAGCAGGGCAAACAA
CACGCTGGCATGCATTAACCGTTGCATCTCATGCAAAACTAAGGAAGTGATACTCCCACTGTACTCCGCAC
TGGTGAGACCGCAGCTGGAGTACTGCGTCCAGTTCTGGGCGCCGCA--
CTTCAACAAGGACGTGGAAAAACTTGAGAGGGTCCAGAGAAGAGCCAC--
CCGTATGATCAGGGACTTGCAAGACAAGCCATACGAGGAGAGGCTGAGGGACTTGGGCCTCTTCAGCCTA
CAGAAGAG--AAGGCTGAGAGGGGAC--------TTGGTAGCGGCTTACCGCTACATCAGGGGA---
GTACATCAAGAACTCGGTGAACAGCTGTTCACCAGGGCACCCCTGGGGAAGACCAGGAG-
CAATGGATACAAACTCCTGGAAGG---
CCGCTTCAGGCTCAATTCCAGGAAAAACTCCTTCACAGTCAGGGTGTCCAGACTGTGGAATAAACTCCCTC
CAGTGGTGGTGCAGTCACCTACCCTGGAAA-
TCTTCAAAAGGAGACTGGACAGTCACCTCGCTGGGGTCACTTGATTTCCTGCCT-AGTG--CA-----
GGGGGACTGGA-CCCGATGATCT-ACAAGGTCCCTTCCAGC--CCCTAA
>CR1-10B_CPB
GAAGGAGTACGGCAGAAAGGGATCT-
AGGGGTTATAGTGGACCACAAGCTAAATATGAGTCAACAGTGTGATGCTGTTGCAAAAAAAGCAAACAT
GATTCTGGGATGCATTAACAGGTGTGTTGTGAGCAAGACACGAGAAGTCATTCTTCCGCTCTACTCTGCGC
TGGTTAGGCCTCAGCTGGAGTATTGTGTCCAGTTCTGGGCACCGCA--
TTTCAAGAAAGATGTGGAGAAATTGGAGAGGGTCCAGAGAAGAGCAAC--
AAGAATGATTAAAGGTCTTGAGAACATGACCTATGAAGGAAGGCTGAAAGAATTGGGTTTGTTTAGTTTG
GAAAAGAG--AAGACTGAGAGGGGAC--------ATGATAGCAGTTTTCAGGTATCTAAAAGGG---
TGTCATAAGGAGGAGGGAGAAAACTTGTTCACCTTAGCCTCTAAGGATAGAACAAGAAG-
CAATGGGCTTAAACTGCAGCAAGG---
GAGGTTTAGGTTGGACATTAGGAAAAAGTTCCTAACTGTCAGGGTGGTTAAACACTGGAATAAATTGCCT
AGGGAGGTTGTGGAATCTCCATCTCTGGAGA-
TATTTAAGAGTAGGTTAGATAAATGTCTATCAGGGATGGTCTAGGGTCCTGCCA-TGAGGGCA-----
GGGGA-CTGGA-CTCGATGACCTCTCGAGGTCCCTTCCAGT--CCTAG-
>CR1-10D_AMi
CCAGCACTACAAATGAAAGGGATCT-
GGGGGTAACAATAGACCCCAGCATGAATATGAGCCGGCAATGCGACGCAGTAGCCGCTAGGGCCAATAA
AACTCTGGCATGCATCAATCGATGCATCTCCAGTAAATCCAAAGAAGTGATTCTCCCGCTTTACTCAACAT
TGGTGCGGCTGCAGCTGGAGTATTGCGTCCAGTTCTGGGCGCCGCA--
CTTCAACAAGGATGTGCTAAAGCTCGAGAGGGTCCAGAGAAGGGCCAC--
CCGCATGATCAGGGACTTGCATGGCAAGCCATATGAGGAGAGGCTGAGGGATCTCGGACTCTTCAGCCTA
AAGAAAAG--AAGGCTGAGAGGGGAC--------CTGATTGCGGCTTACCGCTATATCAGAGGC---
GTACATCAGGGGCTCGGTGAACAACTGTTCACCAGGGTGCCCTGGGGGAAAACTAGAAA-
CAACGGCCACAAACTCCTGGAAGA---
TAGTTTCAGGCTGAACATTAGGAAAAACTTCTTTACAACTAGGGTGTCCAGACTATGGAACAAGCTCCCC
CCAGAGGTGGTGCAATCATCTACCCTAGAGG-
TCTTCAAGAGAAGACTGGACGGTCACCTGGCTGGGGTCATCTGATTTCCTGCTC--ATG--CA-----GGGGG-
CTGGA-CCCGATGATCCTTCAGGGTCCCTTCCAGC--CCT-AT
>CR1-10_AMi
CTAGCACCACGAATGAAAGGGACCT-
GGGGGTAATAATAGACCACAGTATGAATATGAGCCGGCAGTGCGATGCTGTAGCCAGTAGGGCAAATAA
TACTCTGGCATGCATCAATCGATGCATCTCCAGCAAAACCAAGGAGGTGATTCTTCCGCTCTACTCAGCAC
TGGTGAGACCGCAGCTGGAGTACTGCGTCCAGTTCTGGGCACCACA--
CTTTAACAAGGACGTGGACAAGCTTGAGAGAGTCCAAAGAAGAGCCAC--
CCGTATGATCAGAGTCTTAAAAGGCAAGCCATACGAGGAAAGGCTGAGGGATCTGGGACTCTTCAGCCTG
AGGAAAAG--AAGGCTGAGAGGGGAC--------CTGGTAGCAGCTTACCGCTACACTAGGGGA---
GTACATCAAGGGCTCGGTGAGCAACTGTTCACCAGGGCACCCGAGGGGAAAACCAGGAG-

TAATGGCCACAAACTCCTGGAAGA---
TCGATTCAGGCTCAACATTAGGAAAAACTTCTTCACAGTCAGGGTGTCCAGACTGTGGAATAAGCTCCCTC
CAGAGGTGGTGCAATCGCCTACCCTGGAAA-
TCTTCAAGAGGAGACTAGACAGTCACCTTGCTGGGGTCACCTGATTTCCTGCTT-GGTG--CA-----GGGGG-
CTGGA-CCCGATGATCTTCCGAGGTCCCTTCCGGC--CTT--A
>CR1-10_CPB
GAAGGAGTACTGCGGAAAGGGATCT-
GGGGGTCATAGTGGACCACAAGCTAAATATGAGTCAACAGTGTAACGCTGTTGCAAAAAAAGCGAACAT
CATTCTGGGATGTATTAGCAGGAGTGTTGTAAGCAAGACACGAGAAGTAATTCTTCCGCTCTACTCCGCGC
TGATTAGGCCTCAACTGGAGTATTGTGTCCAGTTCTGGGCGCCACA--
TTTCAGGAAAGATGTGGACAAATTGGAGAAAGTCCAGAGAAGAGCAAC--
AAAAATGATTAAAGGTCTAGAAAACATGACCTATGAGGGAAGATTGAAAAAATTGGGTTTGTTTAGTCTG
GAAAAGAG--AAGACTGAGAGGGGAC--------ATGATAACAGTTTTCAAGTACGTAAAAGGT---
TGTTACAAGGAGGAGGGAGAAAAATTGTTCTTCTTAACCTCTGAGGATAGGACAAGAAG-
CAATGGGCTTAAATTGCAGCAAGG---
GAGGTTTAGGTTGGACATTAGGAAAAACTTCCTAACTGTCAGGGTGGTTAAGCACTGGAATAAATTGCCT
AGGGAGGTTGTGGAATCTCCATCATTGGAGA-
TTTTTAAGAGCAGGTTAGACAAACACCTGTCAGGGATGGTCTAGAGTCCTGCCA-TGAGTGCA-----
GGGGA-CTGGA-CTAGATGACCTCTCGAGGTCCCTTCCAGT--CCTATG
>CR1-10_Crp
GCAGCACAGAGGCAGAGAGGGATCT-
TGGAGTCATGATTGACTCCATGATGAATATAAGTTGCCAATGCGGTGAAGAAATAGGCAGAGCCAACCAC
ACCCTGTCATACATCCACAGATGCATCTCAAGCAGGTCCAAGGAGGTGATCCTCCCCCTCTATGCGGCGCT
GGTCAGGCCGCAGCTGGAGTACTGTGTCCAGTTCTGGGCGCTGCA--
CTTCAAGAGGGATGCGGAGAACCTGGAGAAGGTCCAGAGGAGGGCCAT--
GCACATGGTTGGGGGCCTCTGTGGCCAGCCCTGTGAGAAGAGGCTGAGGGACCTGAATCTCTTCAGTATC
TGCAAGAG--GAGACTGAGGGGGGAT--------CTTGTGGCTGCCCACAGCCTTATCAGGGGC---
CACCAGCAGGGACTCAGGGAAAGTCTGTTCACCAGGGC-CCCCTTGGAGTCACCAGGAA-
CAATGGCCACAAGCTGAGGGAGGA---
TAGGTTTAGGTTGGATATTAGGAAGAACTACTTCACTGTAAGGGTGGCCAGAGCCTGGAACAGGCTTCCC
AGASAGGTGGTGCTCTCACCTKCCCTGGAAA-
TCTTCAAGAGGAGACTGGATAGGCACCTGGCTGGGGTCTTATGAMTTCCTGCCC-ATGG--CA-----GGGGG-
TCAGA-CTMGATGATCTGTCTAGGTCCCTTCCGAC--TCA-CT
>CR1-11_AMi
CTAGCACCATGACCGAAAGAGACTT-
GGGGGTCATGATTGACCACAAGATGAATATGAGCCACCAATGCGATACCGCAGCTGGCAAAGCGAACAA
AACTCTGGCTTGCATCTACCGATGCATCTCGAGCAAGACCCAGGATGTCATCCTCCCGCTGTACTCGGCCT
TGGTGAGGCCGCAGCTGGAGTACTGCATCCAATTCTGGGCTCCACA--
ATTTAGGAAGGACGTGGAGAAGCTTGAAAGAGTCCAGAGGAGAGCCAC--
GCGCATGATCAGAGGGCAAGAGAACAGGCCTTATGAAGAGAGGCTGCGAGCTATGGGACTCTTCAGCCT
GGAGAAGCG--CAGGCTCAGGGGTGAC--------TTGGTGGCAGCCTATAAGTACATAAGGGGT---
GTGCATCAGGATCTGGGGGAACACCTGTTCACCAGAGCACCCCAGGGGATGACAAGGTC-
CAACGGTCACAAACTCCTGCAAGA---
CCATTTTAGGCTGGACATAAGGAAAAACTTCTTTACTGTCCGAGCCCCCAAGGCCTGGAACAGACTCCCC
CCAGAGGTGGTGCAAGCACCTACTCTGGACA-
CTTTCAAGAAACATTTGGACGTTTATCTTGCTGGGATCCTTTGATTCCTGCCCC-TTGG--GC-----
AGGGGGCTGGA-CCCGATGATCTTACGAGGTCCCTTCCAGC--CCT--A
>CR1-11_Crp
AAAT------GCTGGAGAAAGACCT-
GGGTGTACTAGTTGATCACAGGATGACTATGAGCTACCAGTGTGATGCAGCCATGAAAAAGGCAAATGCA
ATCCTAGGATGCATGAG--AAGGTATTTCCAGTAGAGATAGGGAAGTATTAATGCCATTGTACAAGG-
ACTGGTGAGACCTCATCTGGAATACTGTGTACAGTTCTGGTCACCCAT--
GTTCAAGAAAGATGAATTCAAACTGGAACAGGTGCAGAGAAGGGCTAC--
TAGGATGATCAGGGGAATGGAGAGCCTATCTTATGAGAGGAGACTAAAAGAGCTTGGCTTGTTTAGTCTA

GCAAAACG--AAGGCTGAGAGGGGAT--------ATGATTGCTGCCTGTAAATACATCAGGGGG---
GTAAACACCAGGGAGGGAGAAGAACTGTTTAAGCTAAAGGACAATGTTGGCACAAGAAC-
AAATGGGTATAAACTGGCCATGAA---
TAAATTTAGGCTGGAAATTAGAAGAAGGTTCCTAACCATCAGAGCAGTGAGGTTCTGGAACAGCCTTCCA
ATAGGAGTAGTGGGGGCAAAAAACCTAACTA-
GTTTTAAGATGGAGCTTGATCAGTTTATGAAAGGGATTATATGA-----------------CA-----GGAGG-T----------
--------------CCCTTCCAGT--CCTA--
>CR1-12B_CPB
AAGCGACAGAGGAGGAGAGAGACCT-GGGTGTGTGGGTCGATCACAGGATGACTATGAGCCACCA-
TGCGATGCAGCTGTGAAAAAGGCAAATGCGATCCTAGGATGTATCAGGCGAGGCATTTCTAGTAGAGATA
GAGAAGTATTAATGCCATTGTACAAGGCACTGGTGAGACCTCATCTGGAATACTGTGTACAATTCTGGTC
ACCCAT--
GTTCAAGAAAGATKAATTCAAACTGGAACAGGTGCAGAGAAGAGCTACGGTAGGATGATCAGGGGAATG
GAGGGCCTATTTTATGAGACGAGACTGGAAGAGCATGGCTTGTTTAGCCTAGCAAAAAG--
AAGGCTGAGAGGGGAT--------ATGATTGCTCTCTATAAATACATCAGAGGG---
GTAAACACCAGGGAGGGTGAAGAGCTATTTAAGCTAAAGGACAATGTTGGCACAAGAAC-
AAATGGGTATAAGCTGGCCMTGAA---
CAAATTCAGGCAGGAAATTAGAAGAAGGTTTCTAATCATCAGAGGAGTGAGGTTCTGGAACAGCCTCCCA
CTAGGAG--GTTGGGGCAATCAACTTAACTA-GTTTTAAGAGAG-GCTGGACACATT--
TGAGTGCGATTGTATGATGGCCTGTGA-TATA--CA-----GGAGG-TCAGA-CTAGATGATC--
TGGTGTGCCCTTCTGGC--CTTA--
>CR1-12_CPB
AAGTGACAGAGGAGGAGAGAGACCT-
GGGTGTGGGGGTCAATCACAGGATGACTATGAGCCACCAGTGTGATGCAGCCATGGAAAAGGCAAATGC
AATCCTAGGATGTATCAGGCGAGGCATTTCCAGTAGAGATAGAGAAGTATTAATGCCATTGTACAAGGCA
TTGGTAAGATCTCATTTGGAATACTGTGCACAATTCTGGTCACCCAT--
GTTCAAGAAAGATTAATTTAAACTGGAACAGGTGCAGAGAAGAGCTAC--
TAGGATGATCAGGGGAATGGAGCCTAT---
CTATGAGAGGAGACTGGAAGAGCTTGGCTTGTTTAGTCTAGCAAAAAG--AAGGCTGAGAGGGGAT--------
ATGATTGCTCTCTATAAATACATTAGGGG----
GTAAATACCAGGGAGGGTGAAGAGCTATTTAAGCTAAAGGACAATGTTGGCACAAGAAC-
AAATGGATATAAACTGGCCATGAA---
CAAATTCAGGCTGGAAATTAGAAGAAGGTTTCTAACCATCAGAGGGATGAGGTTCTGGAACAGCCTCCCA
ATAGGAGTTGTGGGGGCAAACAACTTAATTA-
GTTTTAAGAGAGAGAGCTGGACAAATTTATGAGTGCGATTGTATGATGGCCTGTGA-TATA--CA-----GGAGG-
TCAGA-CTAGATGATC--TGGTGGTCCCTTCTGGC--CTTA--
>CR1-12_Crp
CTGGCACAGCATCTGAAAGGGACCT-
GGGGGTGACAATCGACCAGAAGATGAACATGAGCCAACAGTGCGATGAAGTCGCCAGCAGGGCAAATAG
AACTCTGGCGTGCATCAGCCGATGTGTTGCCAATAGATCCAGGGAGGTGCTCCTCCCCCTCTATTCGGCAT
TGGTGAGGCCACAGCTGGAGTACTGTGTCCAGTTCTGGGCACCGCA--
CTTCAGGAAGGATGTGGATAAGCTTGAGAGGGTACAGAGAAGGGCCAC--
CCATATGATTAGGGGCCTGGAGGATAGGCCCTATGAGGAGAGACTCCGGGAACTGGGCCTGTTCAGTCTG
AGTAAGAG--AAGACTGAGAGGTGAC--------TTGATAGCTGCCTACAAGTATGTCAGGGGT---
GAGCACCAAGATCTAGGGGAGCAACTCTTCAGGAAGGCACCCCTTGGGAGGATGAGGAC-
CAATGGGCACAAGCTAATTGAGGG---
AAAATTTAGGCTGGACATAAGGAAAAACTTCTTTTCCATAAGGGTAACTAGAATCTGGAACACACTCCCA
GCAGAGGTGGTGCAGTCGCCATCCTTGGAGG-
TGTTCAAGAGGAGGCTGGACAAGCACCTCGTTGAGCTTGTTTGACCTCCTGCCC-ATGG--CA-----GGGGA-
CCGGA-CTTGATGATCTTGC-AGGTCCCTTCCAGT--CCT-TT
>CR1-13_AMi

AAMAAGGGATTAGAAAATAATACTGAATGTAGTACAATGCCATTATATAAACCGATGATGCA--
CTTCACTTGAATACCACGTTCAGCTCTGGTCACCCTA--TTTCAAAGAAATAGAGCAGAAAC----

AAGGAGTTCGAGACAGGCAAC--GAAAATGATTAGAGG--
CAGAGAGACTTCCATATGAAGAGAGAATGAAGA---CTGGACTGTTTAGTTTAGAGAGGAG--
ATGAATGAGGGGGCAT----------GATAGAGGTATACAAAATAATGAATGG------
ACAGGGGAGCAATCGGGAGCTCCTATTTACCCTTTCTCATAACACAAGAACAAGG---
ACATTCAATGAAATTGAAAGGCAA---
CAATTTAAAACTGATAAAAGGAAATGCTTTTTTTACACAATGCATAATTAACCTGTGGAACTCACTGCCAC
AAGATATCACTGAGGCCAAGAGCTTAGCAG-GATTCAAAAAG-
GATTAGACATTTATATGACTGACGGGGTTAGGGCCACTGTCA-GAGA--CA-----GGATA-CTGGA-
CTAGATGGAC--CACTGGTCATTCC------------
>CR1-14_AMi
CTGTTACCACACAGGAAAGAGATCT-
GGGAGTCATTGTGGACAGTTCGCTAAAAACATCAGCTCAGTGCTCAGCAGCCGTCAAAAAGGCAAACAA
AATGTTAGGGATTATTAAGAAGGGAATTGTAAACAAAACGGAAAGTATCATTATGCCCCTTTATAAATCC
ATGGTGCGTCCACACCTTGAATACTGTGCCCAGTTCTGGTCCCCACA--
CCTCAAAAAGGATATAGAAGAACTAGAGAAGGTACAGAGAAGGGCAAC--
AAGGATGATTAGCGGTATGGAGGGGCTTCCATATGAGGAGAGACTAAAGAGGCTAGGCCTATTCAGTTTA
GAAAAGAG--ACGCTTGAGGGGGGAC--------ATGATAAGGGTTTACAAAATACTAAATGGC---
GAAGAGAAAGTAAATAGGGATTTATTATTTACTC--TCTCACAATACAAGAACTAGGGG-
TCACAAAATGAAACTAGTAGGTAG---
TAAGTTTAAAACTAACAAAAGGAAGTTCTTTTTCACACAGCGTGTAATTAAAGTGTGGAACTCATTGCCAC
CAGATGTTGTGGAAGCTGACAGTTTAGCCA-
GATTCAAAAAGGGATTGGACAAATTCTTGAGCATGGGAGTTAGGGCCACTGTGT-GACA--CA-----GGATA-
CTGGG-CGAGATGGAC--CTATGGTCTGACCCAGTAAATGGCA
>CR1-14_Croc
AAAGCACAGAGGCGGAAAGGGATCT-
TGGAGTCATTATTGACTCCAAGATGAACATGAGCCGCCAATGCCAGACCGCAGCCAGCAAGGCCAGCCAT
ACCTTGTCATGCATCCAAAGATGCATCTCAAGCCGGTCCAGAGAGGTGATACTCCCCCTCTATGCGACTTT
GGTCAGGCCGCAGTTGGAGTACTGCGTCCAGTACTGGGCGCCGCA--
CTTCAAAAGGGATGTGGCCAGCCTGGAGAGGGTTCAGAGGAGGGCCAC--
CCGCTTGGTGAGAGGGCAGCAGGACAGGCCCTATGAGGAGAGACTGAGGGACCTGAACCTGTTCAGCCT
CAGCAAGAG--GAGGCTGAGGGGGGAC--------CTGGTGGCTGCCTACAAACTCATCAGGGGA---
GATCAACAGCAAATAGGAGA-GCCCTTTTCTCCCCAGCACCACCTGGGGTGACGAGGAA-
CAATGGTAATAAGCTGATGGAGAA---
TAGGTTTAGGTTAGAGATCAGAAGGCAATATTTTACAGTTAGGGTGGCCAAAATCTGGAACCAACTTCCC
AGGGAAGTGGTCCTCGCCCCTACCTTGGGCA-
AATTCAAGAGGAGGTTGGATGATCACCTGTCTGGGGTCTTGTGAATTCCTGCCT-GTGG--CA-----GGGGG-
TCAGG-CTAGATGATCTGTTCAGGTCCCTCCTGAC--CCT--A
>CR1-15_AMi
CTGTTACGACTCAGGAAAGAGACCT-
TGGAGTCATTGTAGATAGTTCTCTAAAAACGTCAGCTCAGTGTGCAGCGGCGACCAAAAAAGCCAATAAA
ATGTTGGGCATCATTAAGAAGGGAATTGAAAACAAAACGGAGAACATCATCATGCCATTATACAAATCCA
TGGTGCGCCCACATCTTGAATACTGTGTGCAGTTCTGGTCTCCGCA--
TCTCAAAAAGGATGTAGTAGAATTAGAAAAGGTACAGAGAAGGGCAAC--
CAAAATGATCAGGGGCATGGAGCAGTTGCCATACCAGGAGAGACTAAAAAGGCTAGGACTCTTCAGTTTG
GAAAGAAG----GCT---GAGGGGAT--------ATGATAGAGGTCTATAAAATCATGAAGGGT---
GTGGACCAAGTGAACAGGGAACTGTTGTTCACCAAGTCCCAGAATACTAGAACTAGGGG-
GCACCCACTGAAATTAGTAGGAGA---
CAGGTTTAAAACTAACAAGAGAAAGTACTTTTTTACGCAACGCATAGTTAACTTGTGGAATTCATTGCCAC
AGGAGGTGGTAGAGGCAGATAGCATAGCCA-
GGTTCAAAAAGGGACTAGACAAATTCATGAACAGAATGGTTGGA-CCACTGTCG-GAGA--CA-----GGATA-
CTGGG-CTAGATGGAC--CATTGGTCTGACCCAGT--ATGGCA
>CR1-16_Croc
GCAGCACAGAAGCGGAAAGGGATCT-
CGGAGTCATAGTGGACTCCAAGATGAACATGAGTCGTCAGCGTGACGAAATCATCAGCAAAGCTAACCGC

ACTTTATCATGCATCAGCAGATGCATGACAAATAGATCCAAGGAGGTGATACTTCCCCTCTATGCGGCATT
GGTCAGACCGCAGTTGGAGTACTGCGTCCAGTTTTGGGCGCCGTA--
CTTCAAGAAGGATGTTGATAGACTCGAGAGGGTCCAGAGGAGGGCCAC--
TCGTATGGTTAGGGGCTTACAGGACAAGCCCTATGAGGAGAGACTGAGGGACCTGGACCTCTTCAGCCTC
CGCAAGAG--AAGGCTGAGAGGTGAT--------CTTGTGGCCGCCTACAAATTCATTAGGGGG---
ACGCAGCAAGGGATCGGAGATGCTCTGTTCACCAGGGCGCCTCTTGGGGTAACAAGGAA-
CAATGGTCACAAACTGACAGAGAG---
CAGATTTAGGCTAGATATCAGGAAAAACTTCTTCACGGTAAGGGTGGCCAAAATTTGGAATGGGCTTCCA
AGGGAGGTGGTGCTCTCCCCTACCTTGGGGG-
TCTTTAAGAGAAGGCTGGATAGGCATCTGGCTGGGGTCATCTGATTTCCTGCCT-A-GG--CA-----GGGGG-
TCGGA-CTCGATGATCTGTTGAGGTCCCTTCCGAC--CCT-A-
>CR1-17_AMi
TCAACACGGTGGCAGAAAGGGATCT-
TGGAGTCATTGTTGACTCCAGGATGAACATGAGCCGCCAATGCGAGGAAGCGGTCAGTAAGGCTAACCGC
ACCTTGTCGTGCATCTACAGATGCATCACAAGCAGGTCCAGGGAGGTGATCCTTCCCCTCTATGCGGCATT
GGTCAGGCCGCAGTTGGAGTACTGCGTCCAGTTCTGGGCGCCGCA--
CTTCAAGAGGGATGTGGCTAGCATTGAGAGGGTCCAGAGGAGGGCCAC--
TCGCATGGTCAAGGGGCAGCGGGGCAGGCCCTACGAAGAGAGGCTAAAGGGCCTGAACCTATTCAGCCT
CCACAAGAG--AAGGCTGAGAGGGGAT--------CTGGTGGCCGTTTACAAACTCACCAGGGGG---
GACCAGCGGGAATTGGGGGAGGCTCTGTTCCCCCGGGCACCACCCGGGGTTACTAGGAA-
TAACGGCCACAAATTGTTAGAGAG---
AAGGTTCAGGCTGGACATCAGGAGACATTACTTTACAGTTAGGGCTGCCAGGCTCTGGAATGGGCTCCCA
AGTGAGGTGGTGCTCTCTCCTACCTTGGGGG-
TCTTCAAGAGGAGGCTGGACAGATATTTGGCTGGGGTGATATGAGTTCCTGCCC-GGGG--CA-----GGGGG-
TCGGA-CCTGATGATCTGTTTAGGTCCCTTCCGAC--CCT-A-
>CR1-17_Croc
CTAGCACCACGTCCGAAAGGGACCTGGGGGGTCATAATTGACCATAAAATGAATATGAGCCACCAGTGTG
ATGCTACAGTTAGCAGAGCAAACAATACGCTGGCATGCATCAACCGATGCATCTCAAGCAAAACTAAGGA
AGTTATTCTCCCGCTCTACTCAGCATTGGTGAGGCCGCAGCTGGAGTACTGCGTCCAGTTCTGGGCGCCAC
A--CTTCAAGAAGGATGTGGAGAAGCTTGAGAGAGTCCAGAGAAGAGCCAC--
TCGTATGATTAGAGGCCTGAAGAGCAAGCCATACGAGGAAAGGCTGACAGATATGGGACTGTTCAGCCTA
GAAAAGAG--AAGACTTTGGGGGGAT--------TTGGTGGCAGCTTACAAATATATCAGGGGT---
GAGCATCAGGGGCTAGGCGAACAACTATTCACCAAAGCGCCCCAGGGGAAAACCAGGAG-
CAACGGTCATAAACTCCTAGAAGA---
AGGTTTCAGACTGGATATAAGGAAAAACTTCTTCACGGTTCGTGTGACCAGACTCTGGAATAGACTCCCA
GCAGGGGTGGTGCAGGCACCTACCCTGGAGA-
TCTTCAAAAGGAGACTGGACGCACACCTTGCTGGGGTTATTTGATTTCCTGCCC-AGAG--CA-----
GGGGGGCTGGA-CCCGATGATCTCACGAGGTCCCTTCCAGC--CCT-AA
>CR1-17_Gav
TCAGCACAGAGGCGGAAAGGGATCT-
TGGAGTCATTGTTGACTCCAGGTTGAACATGAGCCGCCAATGCGAGGAAGCTGTCAGTAAGGCTAACCGC
ACCTTGTCATGCATCCACAGATGCATCACAAGCAGGTCCAGGGAGGTGATCCTCCCCCTCTATGCAGCATT
GGTCAGGCCGCAGTTGGAGTACTGCGTCCAGTTTTGGGCGCCGCA--
CTTCAAGAGAGATGTGGCTAGCATTGAGAGGGTCCAGAGGAGGGCCAC--
TCGCATGATCAGGGGGCAGCAGGGCAGGCCATACGAGGAGAGACTAGAGGGCCTGAACCTATTCAGCCT
CCACAAGAG--AAGGCTGAGGGGGGAT--------CTGGTGGCCGTCTACAAATTCACCAGGGGG---
GATCAGCAGGGAATAGGAGATGCCCTGTTCCCCCGGGCACCACACGGGGTAACTAGGAA-
TAATGGCCACAAGTTGATAGAGAG---
CAGGTTCAGGCTAGACATCAGGAGGCATTACTTTATGGTTAGGGTGGCTAGGACCTGGAACCAACTTCCA
AGGGAAGTGGTGCTCGCTCCTACCTTGGGGG-
TCTTTAAGAGGAGGCTGGATAGACACCTGACTGGGGTCATATGAATTCCTGCCC-AAGG--CA-----GGGGG-
TCGGA-CTTGATGATCTGTTTAGGTCCCTTCCGAC--CCT-A-
>CR1-18_Crp
GCAGCACAGCAGCGGAAAGGGATCT-

TGGAGTCATAGTTGACTCCAAGATGAACATGAGTCGTCAATGTGACGAAGTGATCAACAAGGCTAACCGC
ACTTTATCATGCATTAGCAGATGCATGACGAACAGGTCCAGGGAGGTGATGCTTCCCCTCTATGCGGCACT
GGTCAGGCCGCAGTTGGAGTACTGCGTCCAGTTTTGGGCGCCGCA--
CTTCAAGAGGGATGCGGAGAATCTTGAGAGGGTTCAGAGGAGGGCCAC--
TCGTATGGTCAGAGGCCTGCAGGCAAGGCCCTACGAGGAGAGACTGAGGGACCTAGATCTCTTCAGCCTT
CGCAAGAG--AAGGCTGAGAGGTGAT--------CTTGTGGCTGCCTATAAATTCATCAGGGGA---
GGGCAGCAGGGAATAGGAGATGCTCTATTTACTAGGGCACCCCCTGGAGTAACTAGGAA-
CAATGGCCACAAATTGATGGAGAG---
CAGATTTAGGTTGGACATTAGAAAGAACTTCTTCACAGTAAGGGTTGCCAGAATCTGGAATGGGCTTCCA
AGGGAGGTGGTGCTCTCCCCTACCTTGGGGG-
TCTTCAAGAGGAGACTGGACAAGCACCTAGCTGGGGTCATCTGATTTCCTGCCC-AGGG--CA-----GGGGG-
TCAGA-CTCGATGACCTACTGAGGTCCCTTCCGAC--CCT-A-
>CR1-1B_CPB
CTGTTACCACTCAAGAAAGAGATCT-
TGGAGTCACTGTGGATAGTTCTCTGAAAACATCCACTCAATGTGCAGCGGCAGTCAAAAAAGCGAACAGA
ATGCTGGGAATAATTAAGAAAGGGATAGATAATAGGACAGAAAATATCATGTTGCCTCTATATAAATCCA
TGGTACGCCCACATCTTGAATACTGTGTGCAGATGTGGTCGCCCCA--
TCTCAAAAAAGATATATTGGAATTGGAAAAGGTTCAGAAAAGGGCAAC--
AAAAATGATTAGGGGTATGGAACGGCTTCCGTATGAGGAGAGATTAATAAGACTGGGACTTTTCAGCTTG
GAAAAGAG--ACGGCTAAGGGGAGAT--------ATGATTGAGGTCTATAAAATCATGACTGGT---
GTAGAGAAAGTAGATAAGGAAGTGTTGTTTACTACTTCTCATAACACAAGAACTAGGGG-
TCACCAAATGAAATTAATAGGCAG---
CAGGTTTAAAACAAATAAAAGGAAGTATTTCTTCACACAACGCACAGTCAACCTGTGGAACTCCTTGCCA
GAGGATGTTGTGAAGGCCAAGACCATAACAG-
GGTTCAAAAAAGAACTAGATAAATTCATGGCCAGGATGGGCAGGGCCACTGTCGGAAGA--CA-----
GGATA-CTGGG-CTAGATGGAC--CTTTGGTCTGACCCAGT--AGGGCC
>CR1-1_ACo
ATAGTAGTAACTGTGAGAGGGATCT-
TGGAGTCCTAGTGGACAATCACTTAAATATGAGCCAACAGTGTCGTGCAGCTGCCAAAAAAGCCAATACA
ATCCTRGGCTGCATWAACAGAGGGATAGAATCAAGATCATGTGAAGTRTTAATACCACTTTATAAAGCCT
TGGTAAGGCCACACTTGGAATACTGCATCCAGTTTTGGTCACCACG--
ATGTAAAAAAGATGTTGAGACTCTAGAAAGAGTACAGAGAAGAGCAAC--
AAAGATGATTAGGGGACTGGAGGCTAAAACATATAAGGAACGGTTGCTGGAATTGGGTATGTCTAGTCTA
ATGAAGAG-AAGGACT-AGGGGGGAC--------ATGATAGCAGTGTTCCAATATCTCAGGGGC---
TGCCACAAAGAAGAGGGGGTCAAGCTATTCTCCAAAGCGCCTGAAGGCAGGACAAGAAG-
CAACGGATGGAAACTAATCAAGGA---
GAGAAGCAACCTAGAACTAAGGAGAAATTTCCTGACAGTGAGAACAATTAACCAGTGGAACAACYTGCC
TCCAGAAGTTGTGGGTGCTCCAWCACTGGAGG-
TTTTTAAGAAGAGACTGGACAGCCATTTGTCTGAAATGGTATAGTTTCCTGCCT--GAG--CA-----GGGGG-
TTGGA-CTAGAAGACC-TCCAAGGTCCCTTCCAAC--TCTATT
>CR1-1_CPB
CTACAACTAATCAGGAAAGAGATCT-
TGGAGTCATCGTGGATAGTTCTCTGAAGACGTCCACGCAGTGTGCAGCGGCAGTCAAAAAAGCAAACAGG
ATGTTAGGAATCATTAAAAAAGGGATAGAGAATAAGACGGAGAATATCTTATTGCCCTTATATAAATCCA
TGGTACGCCCACATCTTGAATACTGCGTACAGATGTGGTCTCCTCA--
TCTCAAAAAAGATATACTGGCATTAGAAAAGGTTCAGAGAAGGGCAAC--
TAAAATGATTAGGGGTTTGGAACGGGTCCCATATGAGGAGAGATTAAAGAGGCTAGGACTTTTCAGCTTG
GAAAAGAG--GAGACTAAGGGGGGAT--------ATGATAGAGGTATATAAAATCATGAGTGGT---
GTGGAGAAAGTGAATAAGGAAAAGTTATTTACTTGTTCCCATAATATAAGAACTAGGGG-
CCACCAAATGAAATTAATGGGCAG---
CAGGTTTAAAACAAATAAAAGGAAGTTCTTCTTCACACAGCGCACAGTCAACCTGTGGAACTCCTTGCCT
GAGGAGGTTGTGAAGGCTAGGACTATAACAG-
GGTTTAAAAGAGAACTAGATAAATTCATGGCCAGGATGGGTAAGGCCACTGTCGGTAGA--CA-----
GGATA-CTGGG-CTGGATGGAC--CTTTGGTCTGACCCAGT--ATGGCC

>CR1-1_Croc
GCAGCAGCTCTGCAGAAAAGGACCT-
GGGGGTTACAGTGGACAATAAGCTGAATATGAGCCAACAGTGTGCCCTTGTTGCCAAGAAGGCTAACGGC
ATACTGGGCTGCATTGGTAGGTGTGTTGCCAGCAGGTCAAGGGAAGTGATTATTCCCCTCTATTCAGCACT
GGTGAGGCCACATCTGGAGTACTGTGTTCAGTTTTGGGCCCCCCA--
CTACAGAAAGGATGTGGACAAATTGGAGAGAGTCCAGCGGAGGGCAAC--
AAAAATGGTGAGGGGGCTGGGGCACATGACTTATGAGGAGAGGCTGAGGGAACTGGGCTTATTTAGTCT
AGAGAAGAG--AAGACTGAGAGGGGAT--------TTAATAGCAGCCTTCAACTACCTGAAGGGG---
GGTTCCAAAGAGGATGGAGCTAGACTGTTCTCAGTGGTGGCAGATGACAGAACAAGGAG-
CAATGGTCTCAAGTTGCAGCAAGG---
GAAGTTTAGGTTAGATATTAGGAAGAATTTTCTCACTAGGAGGGTAGTAAAACACTGGAACAGGTTACCC
AGAGAGGTGGTGGAATCTCCATCCTTGGAGG-
TTTTCAAGACCCGGCTAGACAAAGCTTTGGCTGGGATGATCTAGGGTCCTGCTT-TGAG--CA-----GGGGG-
TTGGA-CTAGATGACCTCCTGAGGTCCCTTCCAAC--CCTAAT
>CR1-1_Gav
CCAGCACCACAACTGAACGAGACCT-
GGGTGTCATGACTGACAACAAAATGAACATGAGCCACCAATGCGATGCCGTAGCTGGCAGAGCTAACCA
AACACTGGCATGCATCCACCGAGGCATCTTGAGCAAGGCAAAGGATGTCATCCTCCCACTTTACTCGGCC
TTGGTGAGACCGCAACTGGAGTACTGCGTCCAGTTCTGGGTGCTGCA--
CTTCAAGAAGGATGTGGAGAAGCTCAAGAGAGTCCAGAGGAGGGCCAC--
ATGCATGATTAGAGGCCTGGAGACCAAGACGTATGAGGAGAGGCTGAGGGACTTGGGATTGTTCAGCCTG
GAGAAGAG--AAGACTCAGGGGGGAC--------TTGGTTGCAGCCTATAAGTATATCAGGGGC---
GTACATCAGGGTTTGGGGGAGCATCTATTCACCAAGGCCCCCCAGGGGAGGACAAGAAA-
TAATGGGCACAAGCTGATTGAAGA---
TCGCTTCAGGCTAGACATAAGGAAAAACTTCTTTACAAGCCGAGTGCCGAGGGTTTGGAACAGGCTCCCC
CCAGAAGTGGTACAGTCATCCACCCTGGCGA-
TCTTCAAAAAACGTCTTGATGCCCATCTTGCTGGGATCATTTGATTTCCTGCCCACGTG--CA-----
GGGGGGCTGGA-CCTGATGATCT-GTAAGGTCCCTTCCAGC--CCC-TA
>CR1-1_PMo
ATAGTAGTAACTGTGAGAGGGATCT-
CGGAGTCCTAGTGGACAATCACTTAAATATGAGCCAGCAGTGTGCTGCAGCTGCCAAAAAAGCCAACACA
GTGCTAGGCTGCATTAACAGAGGGATAGAATCAAGATCACGTGAAGTGTTAATACCACTTTATAATGCCT
TGGTAAGGCCACACTTGGAATACTGCATCCAGTTTTGGTCACCACG--
ATGCAAAAAAGATGTTGAGACTCTAGAAAGAGTGCAGAGAAGAGCAAC--
AAAGATGATTAGGGGACTGGAGGCTAAAACATATAAGGAACGGTTGC-GGAATTGGGTATGTCTAGT-
TAATGAAGAG-AAGGACTGAGGGGAGAC--------ATGATAGCAGTCTTCCAATACTTGAAGGGC---
TGCCACAGGGAAGAGGGCATTGATTTATTCTCCATAGCACCTGAGGGTAGGACAAGAA--
CAATGGGTGGAAGCTTTACAGAGA---GAGATCCAACT--
GAAATAAGGAGGAATTTCCTGACTGTGAGAAC--TTAAGCAGTGGAACAGCTCCCTCCTGGA-
GTTGTGGGTGCTCCATCAGCTGGAG-GTTTCAAGAAAGA--TGGACAGCCATTTGTC----
AAGGTATGAATTCCTGCCT-TGGG--CA-----GGGGG-TTGGA-CTAGAAGACC-TCCAAGGTCCCTTCCAAC--
CCTATG
>CR1-20_AMi
CTGGCACAGCATCTGAACGAGACCT-
GGGGGTCACAATCGACTGGAAGATGAATATGAGCCAACAGTGCGATGAGGTTGCCAGAAGGGCAAATAG
AACTCTGGTGTGCATCAGCCGATGTGTTGCCAATAGATCCAGGGAGGTGCTCCTCCCTCTCTATTCGGCGT
TGGTGAGGCCTCAGCTGGAGTACTGTGTCCAGTTCTGGGCACCGCA--
CTTCAGGAAGGATGTGGATAAGCTCGAGAGGGTACAGAGAAGGGCCAC--
CCATATGATTAAGGGCATGGAGGATAGGCCCTATGAGGAGAGACTCCAGGAACTGGGCCTGTTCAGTCTG
AGTAAGAG--AAGGCTGAGAGGTGAC--------TTGATAGCCGCCTACAAGTACGTCAGGGGT---
GAGCACCAAGATCTAGGGGAGCAACTCTTCAGGAAGGCACCTCAAGGGAGGACGAGGAC-
CAATGGGCATAAGCTAATAGAGGG---
TAAATTCAGGCTGGACATAAGGAAAAACTTCTTCTCTGTAAGGGTCACCAGAATCTGGAACACACTCCCA
GCAGAGGTGGTGCAGTCACCCTCCTTGGAGG-

TGTTCAAGATGAGGCTGGACAAGCACCTTGTTGAGCTCGTCTGACTTCCTGCCC-ATGG--CA-----GGGGA-
CTGGA-CTTGATGATCTTAC-AGGTCCCTTCCGGT--CCT-AT
>CR1-2B_CPB
GCAACAGTTCTGCAGAAAAGGACCT-
GGGGATTACAGTGGACGAGAAGCTGGATATGAGTCAGCAGTGTGCCCTTGTTGCCAAGAAGGCCAACAG
CATATTGGGCTGTATTAGTAGGAGCATTGCCAGCAGATCGAGGGAAGTGATTATTCCCCTCTATTCGGCAC
TGGTGAGGCCACACCTGGAGTATTGCGTCCAGTTTTGGTCCCCCCA--
CTACAGAAGGGATGTGGACAAATTGGAGAGAGTCCAGCGGAGGGCAAC--
GAAAATGATTAGGGGGCTGGGGCACATGACTTACGAGGAGAGGCTGAGGGAACTGGGCTTATTTAGTCTG
CAGAAGAG--AAGAGTGAGGGGGGAT--------TTGATAGCAGCCTTCAACTACCTGAAGGGG---
GGTTCCAAAGAGGATGGAGCTAGGCTGTTCTCAGTGGTGGCAGATGACAGAACAAGGAG-
CAATGGTCTCAAGTTGCAGTGGGG---
GAGGTCTAGGTTGGATATTAGGAAACACTATTTCACTAGGAGGGTGGTGAAGCACTGGAATGGGTTACCT
AGGGAGGTGGTGGAATCTCCATCCTTAGAGG-
TTTTTAAGGCCMGGCTTGACAAAGCCCTGGCTGGGATGATTTAGGGTCCTGCTT-TGAG--CA-----GGGGA-
TTGGA-CTAGATGACCTCCTGAGGTCTCTTCCAAC--CCTAAT
>CR1-2B_Croc
CTAGCACCACGGCTGAAAGAGACTT-
GGGGGTCATGATTGACCACAAGATGAACATGAGCCACCAATGCGATGTCGCAGCTGGTAAAGCAAACAA
AACTCTGGCTTGCATCCATAGATGCTTCTCAAGTAAATCTCAGGATGTCATCCTCCCGCTGTACTCGGCCT
TGGTGAGGCCGCAGCTGGAGTACTGCATCCAATTCTGGGCTCCGCA--
ATTCAAGAAGGATGTCGAGAAGCTTGAGAGAGTCCAGAGGAGAGCCAC--
GCGCATGATCAGAGGGCAAGAGAACAGGCCTTATGAAGAGAGGCTGAGAGCCATGGGACTCTTCAGCCT
GGAAAAGCG--CAGGCTCAGGGGGGAC--------CTGGTGGCTGCCTATAAGTACATAAGGGGT---
GTACATCAGGATCTGGGGGAACGTCTGTTCACCAGAGCGCCCCAAGGGAAAACAAGGAC-
CAACGGTCATAAACTCCTCCAAGA---
CCATTTTAGGCTGGACATAAGGAAAAACTTCTTTACTGTCCGAGCCCCCAAGACCTGGAATAGACTCCCTC
CAGAGGTGGTGCAGGCACCTACTCTGGACT-
CATTCAAGAAACGTTTGGATGCTTATCTTGCTGGGATCCTTTGATTCCTGCCCC-TGGG--GC-----
AGGGGGCTGGA-CTCGATGATCTT-CGAGGTCCCTTCCAGC--CCT--A
>CR1-2B_Crp
TCAGCACAGAGGCAGAAAAGGATCT-
TGGAGTCATTATTGATTCCAAGATGAACATGGGCCACCAATGTGAGGACGCGGTCAGTAAGGCTAACCGC
ACCTTGTCATGCATCCACAGATGCATCACGAGCAGGTCCAAGGAGGTGATCCTCCCCCTCTATGCAACATT
GGTCAGGCCGCAGTTGGAGTACTGCATCCAGTTCTGGGCGCCGCA--
CTTCAGGAGGGATGTGGACAGCATTGAGAGGGTCCAGAGGAGGGCCAC--
TCACATGATCAGGGGGCAGCAGGGCAGGCCCTACGAGGAGAGGCTACGGGACCTGAACCTGTTCAGCCT
CCACAAGAG--AAGGCTGAGAGGGGAT--------CTGGTGGCCATCTATAAACTTGCCAGGGGG---
GACCAGCAGGCAATGGGAGAGTCCCTGTTCCCCCGAGCACTACCAGGAGTAAC-AGGAA-
TAACGGCCACAAGTTGACTGAGAG---
TAGGTTCAGGCTAGACATCAGGAGGCACTACTTCACAGTCAGGGCGGCTAGGATCTGGAACCAACTTCCA
AGGGAAGTGGTGCTCGCTCCTACCCTGGGGG-
TCTTTAAAAGGAGGCTAGATAATCACCTAGCTGGGGTCATTTGATTTCCTGCCC-ATGG--CA-----GGGGG-
TCGGA-CTTGATGACCTGCTCAGGTCCCTTCCGAC--CCT-A-
>CR1-2C_Croc
CTAGCACTACGGAAGAAAGAGACTT-
GGGGGTCATCATTGACCACAAGATGAACATGAGCCTGCAATGCGATGCTGCGGCTAGTAAAGCGACCAA
AACGCTGGCTTGCATCCATAGATGCTTCTCAAGCAAATCCCGGGACGTCATTCTCCCCTTGTACTCGGCCT
TGGTGAGGCCGCAGCTGGAGTACTGCGTCCAGTTTTGGGCTCCACA--
ATTCAAAAAGGATGTGGAGAAGCTTGAGAGAGTCCAGAGAAGAGCCAC--
GCGCATGATCAGAGGTCAGGAAAACAGACCTTACGATGACAGGCTGAGAGCCATGGGGCTCTTTAGCCTG
GAAAAGCG--CAGGCTCAGGGGTGAT--------CTGATGGCCACCTATAAGTTTATCAGGGGT---
GACCACCAGGATCTGGGGGAACGTTTGTTCACCAGAGCGCCCCAAGGGATGACGAGGTC-
GAACGGTCATAAACTACTGCAAGA---

CCGTTTCAGGCTGGACATAAGGAAGAATTTCTTTACTGTCCGAGCCCCCAAGGTCTGGAACAGCCTGCCAT
CGGAGGTGGTTCAAGCACCTACATTGAACA-
CCTTCAAGAGCAAATTGGATGCTTATCTTGCTGGGATCCTATGATTCCTGCCCT-TCGG--GC-----
GGGGGGCTGGA-CTCGATGATCTTCCGAGGTCCCTTCCAGC--CCT--A
>CR1-2_AMi
CTAGCACTACGGACGAAAGGGACTT-
GGGGGTCATGATTGACCACAAGATGAACATGAGCCTTCAATGTGATGCTGCGGCTAGTAAAGCGAGCAAA
ACGCTGGCTTGCATCCATAGATGCTTCTCAAGCAAATCCCAGGACGTCATTCTCCCGTTGTACTCGGCCTT
GGTGAGGCCGCAGCTGGAGTACTGCGTCCAGTTTTGGGCTCCACA--
ATTCAAAAAGGATGTGGAGAAGCTTGAGAGAGTGCAGAGGAGAGCCAC--
GCGCATGATCAGAGGTCAGGAAAACAGACCTTATGATGAGAGGCTGAGAGCCATGGGACTCTTCAGCCTG
GAAAAGCG--CAGGCTCAGGGGTGAT--------CTGGTGGCCGCCTATAAGTTTATCAGGGGT---
GCTCACCAGGATCTGGGGGAACGTCTGTTCACCAGAGCGCCCCAAGGGATGACAAGGTC-
GAACGGTCACAAACTCCTCCGTGA---
CCGTTTCAGGCTGGACATAAGGAAGAACTTCTTTACTGTCCGAGCCCCCAAGGTTTGGAATAGACTGCCG
CCGGAGGTGGTTCAAGCACCTACTTTGAACG-
CCTTCAAGAGACATTTGGATGTTTATCTTGCTGGGATCCTATGATTCCTGCCCC-TGGG--GC-----
AGGGGGCTGGA-CTCGATGATCTTCCGAGGTCCCTTCCAGC--CCT--A
>CR1-2_CPB
GCAGCAGTTCTGCAGAAAAGGACCT-
AGGGGTTACAGTGGACGAGAAGCTGGATATGAGTCAACAGTGTGCCCTTGTTGCCAAGAAGGCTAACGGC
ATTTTGGGCTGTATAAGTAGGGGCATTGCCAGCAGATCGAGGGACGTGATCGTTCCCCTCTATTCGACATT
GGTGAGGCCTCATCTGGAGTACTGTGTCCAGTTTTGGGCCCCACA--
CTACAAGAAGGATGTGGAAAAATTGGAAAGAGTCCAGCGGAGGGCAAC--
AAAAATGATTAGGGGGCTGGAGCACATGACTTATGAGGAGAGGCTGAGGGAACTGGGATTGTTTAGTCTG
CAGAAGAG--AAGAATGAGGGGGGAT--------TTGATAGCTGCTTTCAACTACCTGAAAGGG---
GGTTCCAAAGAGGATGGATCTAGACTGTTCTCAGTGGTACCAGATGACAGAACAAGGAG-
TAATGGTCTCAAGTTGCAGTGGGG---
GAGGTTTAGGTTGGATATTAGGAAAAACTTTTTCACTAGGAGGGTGGTGAAGCACTGGAATGGGTTACCT
AGGGAGGTGGTGGAATCTCCTTCCTTAGAGG-
TTTTTAAGGTCAGGCTTGACAAAGCCCTGGCTGGGATGATTTAGGGTCCTGCTT-TGAG--CA-----GGGGG-
TTGGA-CTAGATGACCTCCTGAGGTCCCTTCCAAC--CCTGAT
>CR1-2_Crp
TCAGTACGGAGGCGGAAAAGGATCT-
TGGAGTCATCATTGACTCCAAGATGAACATGGGCCGGCAGTGTGGGGATGCAGTCAGGAAGGCCAACCG
CACCTTGTCATGCATCCACAGATGCATCTCGAGCAGGTCCAAGGAGGTGATCCTCCCCCTCTATGCGGCAC
TGGTCAGGCCGCAGCTGGAGTACTGCGTCCAGTTCTGGGCGCCGCA--
CTTCAAGAGGGATGTGGACAACATGGAGAGGGTCCAGAGGAGGGCCAC--
TCGCATGATCAGGGGGCAGCAGGGCAGGCCCTACGAGGAGAGGCTACGGACCTGAACCTGTTCAGCCT
CCACAAGAG--AAGGCTGAGGGGGGAT--------CTAGTGGCCTGTTACAAACTAGTCAGGGGG---
GACCAGCAGGCACTGGGGGAGTCCCTGTTCCCCCGAGCACTGCCAGGAGTGACCAGGAAATAACGGTCA
CAAGCTGGCAGAGGG---
TAGATTCAGATTAGACATCAGGAGGCGCTACTTCACTGTCAGGGCGGCTAGGATCTGGAACCAACTTCCA
AGCGAAGTGGTGCTGGCTCCTACCCTGGGGG-
TCTTTAAGAGGAGGCTTGACGAWCACCTTGCTGGGGTCGTTTGATTTCCTGCC--ATGG--CA-----GGGGG-
TCGGA-CTTGATGATCTGCTCAGGTCCCTTCCGAC--CCT-A-
>CR1-2_XT
GCAAATCCATAATGGAGAAGGACCT-
TGGAGTCCTTGTAGATAATAAACTTGGCTGTAGCAAGCAATGCCAGGCAGCAGCTGCAAGGGCAAACAA
GGTTTTGAGCTGTATTAAAAGGGGTATAGATTCACGGGAGGAGGGGGTTATTCTTCCCCTTTACAGAGCG
CTGGTAAGGCCCCATCTAGAATATGCTGTTCAGTTTTGGTCTCCAGT--
GCTCAAACGGGACATTATTGAGTTAGAGAGGGTCCAGAGAAGGGCAAC--
TAAGCTGGTAAAGGGTATGGAAAGTCTCAGTTATGAAGAAAGACTGGCCAAGTTGGGTCTGTTTACACTG
GAGAAGAG--GCGCTTAAGAGGTGAC--------ATGATAACTATGTATAAATATATAAGGGGA---

TCATATAATAACCTTTCTAATGTTTTATTTACCAGTAGGTCCTTCCAACGGACACGAGG-
GCACCCACTCCGTTTAGAAGAAGG---
GAGGTTCCATTTAAATATTCGGAAAGGATTTTTTACNGTGAGAGCTGTGAAGTTGTGGAATTCCCTCCCCG
AATCAGTCGTGCTGGCTGATACATTATATA-
ACTTTAAGAAGGGGCTGGATGGATTCTTATTTGCCTTCCTCTGGCAGGTTATATATAGG--CA-T---TATGG-
TTGAA-CTTGATGGAC--GTATGTCTTTTTTCAAC--CCAA--
>CR1-3_Croc
ATGGCAGCACTATGGAAAAGGACTT-
GGGAGTCTTGGTAGATCACAGACTCAATATGAGTCTGCAGTGTGATGCAGCTGCACAAAAGGTGAATGCG
GTTTTGGGATGCATCAATAGAAGCATTAGGTGCAAGACACGGGAGGTGATAGTGCCTCTCTACTCGGCAC
TGGTTAGGCCTCATCTGGAGTACTGTGTGCAGTTCTGGGCTCCACA--
TTTTAAAAAGGACGTGGAGAAGTTAGAGAGGGTCCAGAGGCAGGCAAC--
AAAGATGATMAAGGGCTTGGAAGGCAAGTCATATGAGGAGAGGCTGAAGGAGCTAGGCATGTTCAGCTT
GCGGAAAAG--GCGCTTAAGAGGGGAC--------ATGATAGCAGTCTTCAAATACTTGAAGGGC---
TGCCATAAAGAAGAGGGAAAGCACCTTTTCTCTCTTGCTGCAGAGAGGAGGATGTGGAC-
CAATGGCTTGAAGTTGCAGCAAAG---
TAAGTTTAGATTGGATATCCAGAAAAACTTCTTCACTGTTAGAACAGTGAGGCAGTGGAATAGACTGCCT
AGGGAAGTTGTGGACTCTCCATCACTGGAGG-
TGTTCAAGAAGAGGTTGGACAGCCACTGGTCAGGGATGATCTAGGATCCTGCCT-CAGG--CA-----GGGGG-
TTGGA-CTAGATGACCTCTGGAGGTCCCTTCCAGC--CCTACT
>CR1-3_Gav
CTAGCACCATGACCGAAAGAGACTT-
GGGGGTCTTGACTGACCACAAGATGAACATGAGCCCCCAATGCAATGCTGTGGCCAGCAGAGCAAACCA
AACTCTGGCATGCATCCACCGATGCATCTCAAGCAAAACCAGGGATGTCATCCTCCTGCTTTACTCGGCCT
TAGTGAGGCCGCAGCTGGAGTACTGCATCCAGTTTTGGGCCCCCCA--
CTTCAGGAGGGATGTGGATAAGCTCGAGAGAGTCCAGAGGAGAGCCAC--
CCGTATGATCAAAGGCCAAGAGAGCAAGCCTTACGAGGAGAGGCTGAGGGACATGGGACTCTTCAGCCT
GGTGAAGAG--AAGGCTCAGGGGGGAC--------TTGGTGGCTGCCTATAAGTATATAAGGGGG---
GTGCATCAGGATCTGGGAGAACASTTGTTCACCAGGGCTCCCCAAGGGATAACTAGGTC-
TAATGGCCACAAACTTCTAGAAGG---
CCAATTCAGACTCGATATAAGGAAAAACTTCTTTACAGTCCAAGTGTCCAAGGTCTGGAACAGACTCCCC
CCAGAGGTGGTGCAAGCACCTACTCTGGACT-
CATTCAAAAGGCATTTGGATGTTTATCTTGCTGGGATCATTTGACTTCCTGCCT-CTGG--CA-----
GGGGGGCTGGA-CTCGATGATCTCACAAGGTCCCTTCCAGC--CCC-TA
>CR1-4B_CPB
AAGTAACGGAAGAGGAGAAGGACCT-
AGGGGTCCTGGTAGACCGCAGGATGACTATGAGTCGACAATGTGACGTGGCGGTGAAAAAAGCCAATGC
GGTCTTGGGATGCATTAGGCGAGGKATATCTAGTAGGGATAAGGAGGTSCTGCTTCCGTTGTACAAGGCG
CTGGTGAGACCWCATTTGGAGTACTGTGTGCAGTTCTGGTCTCCCAT--
GTTTAAAAAAGATGAACTCAAACTGGAACGGGTGCAG--

>CR1-4C_CPB
AAGTAACGGAGGAGGAGAAGGACCT-
CGGAGTCCTGGTTGACCGCAGGATGACTATGAGTCGGCAATGTGACGTGGCCGTGAAAAAAGCTAATGCG
GTCTTGGGATGCATTAGGCGAGGTATNTCTAGTAGGGATAAGGAGGTGCTGCTTCCGTTATACAAGGCAC
TGGTGAGACCTCATTTGGAGTACTGTGTGCAGTTCTGGTCTCCCAT--
GTTTAAAAAGGATGAANTCAAACTGGAACGGGTACAGAGAAGGGCCAC--
TAGGATGATCNGAGGAATGGAAAACCTGTCGTATGAAAGGAGACTCGAGGAGCTCGGTTTGTTTACCCTA
ACCAAAAG--AAGGCTGAGGGGGGAT--------ATGATTGCTCTCTTTAAATATATCAGAGGG---
ATAAATACCAGGGAGGGAGAGGAATTATTTCAGCTCAGTACTAATGTGGACACGAGAAC-
GAATGGATATAAACTGGCCGTNGG---

GAAGTTTAGGCTTGAAATTAGACGAAGGTTTCTAACCGTCAGAGGGGTGAAGTTTTGGAACAGCCTTCCG
AGGGAAACAGTGGGGGCGAAAGACCTCTCTG-
GCTTTAAGATTAAGCTNGATAAGTTTATGGAGGGGATGGTTTGATGGCCCGCAT-CATG--CG-----GGAGG-
TCAGA-CTAGATGATC-ATAATGGTCCCTTCTGAC--CTTA--
>CR1-4_CPB
AAGTAACAGAGGAGGAGAAGGACCT-
CGGAGTATTGGTTGATCACAGGATGACTATGAGCCGCCAATGTGATATGGCTGTGAAAAAAGCTAATGCG
GTCTTGGGATGCATCAGGCGAGGTATTTCCAGTAGAGATAAGGAGGTGTTAGTACCGTTATACAAGGCAC
TGGTGAGACCTCATCTGGAATATTGTGCGCAGTTCTGGTCTCCCAT--
GTTTAAGAAGGATGAATTCAAACTGGAACAGGTACAGAGAAGGGCTAC--
TAGGATGATCCAAGGAATGGAAAACCTGTCTTATGAAAGGAGACTCAAAGAGCTTGGCTTGTTTAGCCTA
ACCAAAAG--AAGGCTGAGGGGAGAT--------ATGATTGCTCTCTATAAATATATCAGAGGG---
ATAAATACCAGGGAGGGAGAGGAATTATTTAAGCTCAGTATCATTGTGGACACAAGAAC-
AAATGGATATAAACTGGCCATCAG---
GAAGTTTAGACTTGAAATTAGATGAAGGTTTCTAACCATCAGAGGAGTGAAGTTCTGGAATAGCCTTCCA
AGGGGAGCAGTGGAGGCAAAAGACATATCTG-
GCTTCAAGACTAAGCTTGATAAGTTTATGGAGGGGATGGTATGATGGCCTGCGT-TGTG--CA-----GGAGG-
TCAGA-CTAGATGATC-ATAATGGTCCCTTCTGAC--CTTA--
>CR1-4_Croc
CTAGCACCACGACCGAAAGAGACTT-
GGGGGTCATGATTGACCACAAGATGAACATGAGCCACCAATGCGATGCCGCAGCCGGCAAAGCAAATAA
AACTCTGGCTTGCATCTACCGATGCATCTCAAGCAAGACCCAAGAAGTCATCCTCCCGCTTTACTCGGCCT
TGGTGAGGCCGCAGCTGGAGTACTGCATCCAGTTCTGGGCTCCGCA--
CTTTAGAAAGGATGTGGAGAAGCTTGAGAGAGTCCAGAGGAGAGCCAC--
GCGCATGATTAGAGGTCAAGAGAACAGGCCTTATGAGGAGAGGCTGAGAGGCATGGGACTCTTCAGCCT
GGAGAAGCG--AAGGCTCAGGGGTGAC--------TTGGTGGCAGCCTATAAGTATATAAGGGGT---
GTGCATCAGGAACTGGGAGAACGTCTGTTCACCAGGGCACCCCAAGGGAAGACAAGGTC-
TAACGGTCACAAACTGCTGGAAGA---
CCGTTTTAGACTGGACATAAGGAAAAACTTCTTTACCGTCCGAGTCTCCAGAGTCTGGAATAGACTCCCCC
CAGAAGTGGTGCAAGCACCTACTCTGGACA-
CCTTTAAGAAACGCTTGGATGCTTATCTTGCTGGGGTCATCTGATTCCTGCCCC-TTGG--GC-----
AGGGGGCTGGA-CCCGATGATCTCGCGAGGTCCCTTCCAGC--CCT--A
>CR1-5B_AMi
CTAGCACCACATCTGAACGAGACCT-
GGGTGTCGTGATAGACAACAAAATGAACATGAGCCACCAATGCGATGCCGTAGCTGGCAGAGCTAATCA
AACACTGGCATGCATCTACCGAGGCATCTCGAGCAAGGCTAAGGATGTCATCCTCCCTCTCTACTCGGCCT
TGGTGAGACCGCAGCTGGAGTACTGCATCCAGTTCTGGGCGCCGCA--
CTTCAGGAAGGATGTGGAGAAGCTCGAGAGAGTCCAAAGGAGGGCCAC--
GCGCATGATTAGAGGCCTAGAGACCAGGTCATATGAGGAGAGGCTGAAAGACTTGGGACTGTTCAGCCTG
GAGAAGAG--AAGACTCAGAGGGGAC--------TTGGTGGCAGCCTATAAGTATATCAAGGGC---
GTACATCAGGGCCTGGGGGAGCATCTGTTCACCAAGGCCCCCCAGGGGAGGACAAGAAA-
TAATGGGCACAAGCTGATTGAGGA---
TCGCTTCAGGCTAGACATAAGGAAAAACTTCTTTACAAGTCGAGTGCCGAGGGTTTGGAACAGGCTCCCC
CCAGAGGTGGTACAGTCACCCACCCTGGCGA-
TCTTCAAAAAGCGTCTTGACGCCCATCTTGCTGGGGTCATCTGATTTCCTGCCCAAGTG--CA-----
GGGGGGCTGGA-CCCGATGATCT-GTAAGGTCCCTTCCAGC--CCC-TA
>CR1-5B_Crp
AGAGCACGGAGGCAGAAAGGGATCT-
TGGAGTCATTATTGACTCCAAGATGAACATGGGCCGACAATGCGAGGTCACGGTTGGCAGGGCTAACCGG
ACCTTGTCGTGCATCCACAGGTGCATCTCAAGTAGGGCCAAGGAGGTGATCCTCCCCCTCTACGCGACGCT
GGTCAGGCCGCAGCTGGAGTACTGCGTCCAGTTCTGTGCGCCCCA--
CTTCAGGAGGGATGTGGACAACATTGAGAGGGTCCAGAGGAGGGCCAC--
CCGCATGATCCGGGGACAACAGGGCAGACCCTACAATGAGAGACTGCGGGACCTGAACCTGTTCAGCCTT
CACAAGAG--AAGGCTGAGGGGGGAC--------CTGGTGACCGTCTATAAACTCACTAGGGGG---

GACCAGAAGGGTTTGGGAGAGACCTTGTTTCCCCTAGCGCCCCCTGGCATAACAAGGAA-
TAACGGCCACAAGTTGTTGGAGAG---
TAGGTTTAGATTAGACATCCGTAAGAACTACTTCACAGTCAGGGCGGCTAGGATCTGGAACCAACTTCCA
AGGGAAGTGGTGCTGGCTCCTACCCTGGGGG-
TCTTTAAGAGGAGGCTTGATGCCTACCTGGCTGGAGTCATTTGATCTCCTGCCC-A-GG--CA-----GGGGG-
TCGGA-CTTGAAGATCTACA-AGGTCCCTTCCGAC--CCT-A-
>CR1-5_AMi
CTAGCACCATGACCGAAAGAGACTT-
GGGGGTCTTGATTGACCACAAGATGAATATGAGCCACCAATGCGATGCTGTGGCTGGCAAAGCAAACCAA
ACTCTGGCGTGCATCCACCGATGCATCTCAAGCAAAACCAGGGATGTCATCCTCCCGCTTTACTCGGCCCT
GGTGAGGCCGCAGCTGGAGTACTGCGTCCAGTTTTGGGCCCCCCA--
CTTCAGGAGGGATGTGGAAAAGCTTGAGAGAGTCCAGAGGAGAGCCAC--
TCGTATGATCCAAGGCCTGGAGGGCAGGCCGTACGAGGAGAGGCTGAGGGACTTGGGACTCTTCAGTCTG
GAAAAGAG--AAGGCTCAGGGGGGAC--------TTGGTAGCAGCCTACAATTATATAAGAGGG---
GTACATCAGGACCTGGGAGAACAGCTGTTCACCAGAGCTCCCCAAGGGATAACAAGGTC-
TAACGGCCATAAACTCCAGGAAGG---
CCGATTTAGACTAGACATAAGAAAAAAGTTCTTTACGGTGCGAGTGTCCAGGGTCTGGAACAGACTCCCC
CCAGAGGTGGTGCAAGCACCTACTCTGGACT-
CTTTCAAAAAACATCTGGATATTTTTCTTGCTGGGGTCACTTGACTTCCCGCCT-ATGG--CG-----
GGGGGGCTGGA-CTCGATGATCTCACGAGGTCCCTTCCAGC--CCC-TA
>CR1-5_CPB
GCAGCAGCACTGCTGAGAAGGATCT-
GGGAGTTGTGGTGGATCACAACCTCAACATGAGTCAGCAATGCGATGCTGTTGCAAAAAAAGCAAATGCA
ATTTTAGGTTGCATTAACAGAGGCATAGCATGCAAGTCATGGGAGGTGATAGTACTGCTCTACTCGGTGCT
GGTTAGGCCTCAGCTGGAGTACTGTGTCCAATTTTGGTCACCAAT--
GTATAGAAAGGATGTAGAGAAACTGGAAAGGATCCAGAGGCGAGCGAC--
AAAGATGATCAAAGGGATGGAATGCAAGCCATATGAGCAAAGGCTGAAGGAACTGGGTATGTTTAGTTT
GGAAAAGAG--GAGATTAAGGGGGGAC--------ATGATAGCGGTCTTCAGATACTTGAAAGGC---
TGCCATAAAAAAGATGGAGAAAAGTTGTTCTCTTTTGCCACAGAGGGCAGGACAAGAGG-
CAATGGGTTCAAACTACAGCATAG---
CAGATTTAGATTAAATCTCAGGAAAAACTTCTTAACTGTAAGAACAGTAGGACAATGGAACAGACTGCCT
MGGGAGGTTGTGGAAGCTTCTTCACTGGAGG-
TTTTCAAAAGGAGGCTGGATAGCCATCTGTCTTGGATGGTTTAGAATCCTGCATCTTGG--CA-----GGGGG-
TTAGA-CTAGATGACC-CTTGCGGTCCCTTCTAAC--CCTATG
>CR1-5_Croc
AAAGCACGGTGGCAGAAAGAGATCT-
TGGAGTCATTATTGACTCCAAGATGAACATGGGCCGACAATGCGAGGACACGGTCAGTAGGGCTAACCGT
ACCTTGTCCTGCATCCACAGATGCATCACAAGCAGGGCCAAGGAGGTGATCCTCCCCCTCTATGCGACAC
TGGTCAGGCCGCAGCTGGAGTACTGCGTCCAGTTCTGGGCGCCGCA--
CTTCAGGAGGGATGTGGACAGCATTGAGAGGGTCCAGAGGAGGGCCAC--
TCGCATGATCCGGGGACAGCAGGGCAGACCCTACGAGGAGAGGCTACGGGACCTTAACCTGTTCAGCCTT
CACAAGAG--AAGGCTGAGGGGGGAC--------CTGGTGGCCATCTATAAACTTACTAGGGGG---
GACCAGCAGGGTTTGGGAGAGACCTTGTTCCCCCGAGCGCCTCCCGGAGTAACAAGGAA-
TAACGGCCATAAGTTGCTAGAGAG---
TAGGTTTAGACTAGACATTAGAAGGCACTACTTCACAGTCAGGGCGGCTAGGATCTGGAACCAACTTCCA
AGGGAAGTGGTGATGGCTCCTACCCTGGGGG-
TCTTTAAGAGGAGGCTTGACGTTTACCTGGCTGGGGTCATTTGATCTCCTGCCC-AGGG--CA-----GGGGG-
TCGGA-CTAGAAGATCTACA-AGGTCCCTTCCGAC--CCT-A-
>CR1-6C_AMi
AAAGCACAGTGGCAGAAAGAGATCT-
TGGAGTCATTATCGATTCCAAGATGAACATGGGCCGCCAATGTGAGGACACGGTCAGTAGGGCTAACCGC
ACCTTGTCATGCATCCACAGATGCATCACAAGCAGGGCCAAGGAGGTGATCCTCCCCCTCTATGCGACAC
TGGTCAGGCCGCAGTTGGAGTACTGCGTCCAGTTCTGGGCGCCGCA--
CTTCAGGAGGGATGTGGACAGCATCGAGAGGGTCCAAAGGAGGGCCAC--

TCGCATGATCCGGGGACAGCAGGGCAGACCCTACGAGGAGAGGCTACGGGACCTGAACCTGTTCAGCCTT
CACAAGAG--AAGGCTGAGAGGGGAC--------CTGGTGGCCATCTATAAACTTACCAGGGGG---
GACCAGCGGGGAATGGGAGAGACCCTGTTCCCCCGAGCACCTCCCGGAGTAACAAGGAA-
TAACGGCCATAAGTTGTTAGAGAG---
TAGGTTCAGGCTAGACATTAGAAGGCACTACTTCACAGTCAGGGCGGCTAGGATCTGGAACCAACTTCCA
AGGGAAGTGGTGCTGGCTCCTACCTTGGGGG-
TCTTTAAGAGGAGGCTTGATGATTACCTAGCTGGGGTCATWTGATCTCCTGCCC-AGGG--CA-----GGGGG-
TCGGA-CTTGAAGATCTGCT-AGGTCCCTTCCGAC--CCT-A-
>CR1-6D_AMi
AAAGCACGGTGGCAGAAAGGGATCT-
TGGAGTCATTATCGACTCCAAGATGAACATGGGCCGACAATGCAAGGACACGGTCAGTAGGGCTAACCGC
ACCTTGTCGTGCATCCACAGATGCATCACAAGCAGGGCCAAGGAGGTGATCCTCCCCCTCTATGCGACAC
TGGTCAGGCCACAGCTGGAGTACTGTGTCCAGTTCTGGGCACCGCA--
CTTCAGGAGGGATGTGGACAGCATTGAGAGGGTCCAGAGGAGGGCCAC--
CCGCATGATCCGGGGACAGCAGGGCAGACCCTATGAGGAGAGGCTATGGGACCTKAACCTGTTCAGCCTT
CACAAGAG--AAGGCTGAGGGGGGAC--------CTGGTGGCCATCTATAAACTTACTAGGGGG---
GACCAGTGGGGTWTGGGAGAGACCTTGTTCCCCCGAGCGCCTCCCGGAATAACAAGGAA-
TAATGGCCATAAGTTGTTAGAGAG---
TAGGTTCAGACTAGACATTAGAAGGCACTACTTCACAGCCAGGGCGGCTAGGATCTGGAACCAACTTCCA
AGGGAAGTGGTGCTGGCTCCTACCCTGGGGG-
TCTTTAAGAGGAGGCTTGATGATTACCTGGCTGGGGTCATTTGATCTCCTGCCC-AGGG--CA-----GGGGG-
TCGGA-CTWGAAGATCTGCA-AGGTCCCTTCCGAC--CCT-A-
>CR1-6F_AMi
WGAGCACAGAGGCGGAAAGGGATCT-
TGGAGTCATTATTGACTCCAAGATGAACATGAGCCGCCAATGCCAGACCGCAGCCAGCAAAGCCAACCAC
ACCTTGTCGTGCATCCAAAGATGCATCTCAAGCCAGTCCAGAGAGGTGATACTCCCCCTCTACGCGACATT
GGTCCGGCCACAATTGGAGTACTGTGTCCAGTTCTGGGCACCGCA--
CTTTAAGAAGGATGTGGCCTGCCTTGAGAGGGTTCAGAGGAGGGCCAC--
CCGCTTGGTTGGAGGGCAACAGGGCAGGCCCTATGAGGAGAGGCTGAGGGACCTGAACCTGTTCAGCCTC
AGCAAGCG--GAGGCTGAGGGGGGACTTGGTGGTTTGGTGGCTGCCTACAAACTCGTTAGGGGA---
GATCAGCAGCAAATAGGAAGGGCCCTATTCCCCCCAGCAGCACCTGGGGTGACGAGGAA-
CAATGGCCAGAAGCTGCTGGAGAA---
TAGGTTCAGGTTAGAGATTAGGAGGCACTATTTCACTGTCAGGGTGGCTAGGATCTGGAACCAACTTCCT
AGGGAAGTGGTCCTCGCTCCTACCTTGGGTA-
AATTCAAAAAGAGGTTGGATGAACACCTGTCTGGGGTCGTGTGAGCTCCGGCCA-GTGG--CG-----GGGGG-
TTAGA-CTAGATGATCTATTCAGGTCCCTTCTGAC--CCC-TA
>CR1-6_Croc
TCAGCACAGAGGCAGAAAAGGATCT-
TGGAGTCATTATTGATTCCAAGATGAACATGGGCCGCCAATGTGGGGACGCGGTCAGGAAGGCTAACCGC
ACCTTGTCATGCATCCACAGATGCATCACGAGCAGGTCCAAGGAGGTGATCCTCCCCCTCTATGCGACACT
GGTCAGGCCGCAGTTGGAGTACTGCGTCCAGTTCTGGGCGCCGCA--
CTTCAGGAGGGATGTGGACAGCATCGAGAGGGTCCAGAGGAGGGCCAC--
TCGCATGATCAGGGGGCAGCAGGGCAGGCCCTACGAGGAGAGGCTACGGGACCTGAACCTGTTCAGCCT
CCACAAGAG--AAGGCTGAGAGGGGAT--------CTGGTGGCCGTCTATAAACTGGCCAAGGGG---
GACCAGCAGGCAATGGGAGAGTCCCTGTTCCCCCGAGCACTACCGGGAGTAACAAGGAA-
TAACGGCCATAAGTTGACTGAGAG---
TAGATTCAGGCTAGACATCAGGAGGCACTACTTCACAGTCAGGGCGGCTAGGATCTGGAACCAACTTCCA
AGGGAAGTGGTGCTCGCTCCTACCCTGGGGG-
TCTTCAAAAGGAGGCTAGACGATCACCTAGCCGGGGTCGTTTGATTTCCTGCCC-ATGG--CA-----GGGGG-
TCGGA-CTTGATGATCTGCTCAGGTCCCTTCCGAC--CCT-A-
>CR1-7B_Gav
TCAGCACGGAGGCGGAAAAGGATCT-
TGGAGTCATCATTGACTCCAAGATGAACATGGGTCGGCAGTGTGGGGAGGCAGTTAGGAAGGCCAACCGT
ACCTTGTCATGCATCCACAGATGCATCTCGAGCAGGTCCAGAGAGGTGATCCTCCCCCTCTATGCGGCACT

GGTCAGGCCGCAGTTGGAGTACTGCGTCCAGTTCTGGGCGCCGCA--
CTTCAAGAGGGATGTGGACAGCATGGAGAGGGTCCAGAGGAGGGCCAC--
TCGCATGATCAGGGGACTGCAGGGCAGGCCCTATGAGGAGAGGCTACGGACCTGAACCTGTTCAGCCTC
CAAAAGAG--AAGGCTGAGGGGGGAT--------CTTGTGGCCTGTTACAAACTGGTCAGGGGG---
GATCAGCAGGCATTGGGGGAGTCACTGTTCCCCCGAGCACAGCCAGGAGTGACCAGGAA-
CAACGGTCACAAGCTGGCAGAGGG---
TAGGTTTAGGTTAGACATCAGGAGGCGCTATTTCACGGTCAGGGCGGCTAGGGTCTGGAACCAACTTCCA
AGGGAGGTGGTGCAGGCTCCTACCCTGGGGG-
TTTTTAAGAGGAGGTTAGATGAACACCTTGCTGGGGTCGTTTGATTTCCTGCC--ATGG--CA-----GGGGG-
TCGGA-CTAGATGATCTGCTCAGGTCCCTTCCGAC--CCT-A-
>CR1-7_CPB
AAAGCAGTGACTCTGAAAAGGATTT-
AGGGTCAGTAGTGGACAAGCAACTCAACATGAGCTCCCAGTGCGATGCTGTGGCAAAAAGGGCTAATGT
GATCCTTGGATGTATAAACAGGGGAATAGTA---
ATGAGTAGGGAGGTGATTTTACCTCTATGTATGGCATTGGTGAGACCAGTACTGGAATACTGCGTMCAGT
TCTGGTGTCCATA--TTTTAAAAAGGATGTTGAAAAATTGGAGAGGGTGCAGAAAAGAGCCAC--
AAAAATGATTTAAGGGCTGGAGAAAATGCCTTA-----
GGGGACTTAAAGAGCTCAATCTGTTTAGTTTATCAGAAAG--AAGATTGAGAGGTGAC--------
TTGATTACAGTGTA-AAGTACCTTCACAGG---
GAGAAAATACGGGGTACTAAAGGGCTCTTTAATCTAGCAGAGAAAGGCATAACAAGAAC-
CAATGGCTGGAAGCTGAAGCCAGA---
CAAATTCAAATTAGAAATTAGGCACACATTTTTAACAGTGAGGGTGATTAACCACTGGAACAAACTACCA
AGGGAAGTGGTGGATTCTCCATCTCTTGAAG-
TCTTCAGATCAAGACTGGATGCCTTTCTGGAAGATATGCTTTAGTGGCCTGTGA-TACA--CA-----GGAGG-
TCAGA-CTAGATGATC--TAATGGT-TCTTCTGGC--CTTA--
>CR1-7_Croc
GCGGCACGGAAGCGGAAAGGGATCT-
CGGAGTCCTAGTGGACTCCAAGATGAACATGAGTCGTCAGTGTGACGAAGTCATCAGCAAAGCTAACTGC
ACTTTATCGTGCATCAGCAGATGCATGACAAACAGAACCAAGGAGGTGATACTTCCCCTCTATCGGGCGC
TGGTCAGACCGCAGTTGGAATACTGCGTSCAGTTTTGGGCGCCACA--
CTTCAAGAGGGATGTGGATAACCTGGAGAGGGTCCAGAGAAGGGCCAC--
TCGTATGGTTAAGGGCTTGCAGGCCAAGCCCTACGAGGAGAGACTAGGGCACCTGGACCTCTTCAGCCTC
CGCAAGAG--AAGGTTGAGAGGCGAC--------CTTGTGGCTGCCTATAAATTCATCATGGGG---
GCGCAGAAGGGAATTGGTGAGGCTCTACTCACCAAGGCGCCCCTGGGGGTCACAAGAAA-
TAATGGCCATAAGCTAGCAGAGAG---
CAGATTTAGACTGGACATTAGGAAGAACTTCTTCACAGTTAGAGTGGCCAAAATCTGGAATGGGCTCCCA
AGGGAGGTGGTGCTCTCCCCTACCCTGGGGG-
TCTTCAAGAGGAGGTTAGATAGGCATCTGGCTGGGGTCATCTGATTTCCTGCCT-A-TG--CA-----GGGGG-
TCGGA-CTCGATGATCTATTGAGGTCCCTTCCGAC--CCT-A-
>CR1-7_Crp
CCAGCACGGAGACGGAAAGGGATCT-
TGGAGTCGTCATTGACTCCACGATGAACATGGGCCGGCAGTGTGGGGAGGCAGTCAAGAAGGCCAACCG
TACCCTGTCATGCATCCACAGATGCATCTCGAGCAGGTCCAGGGAGGTGATCCTCCCCCTCTATGCGGCAC
TGGTCAGGCCGCAGCTGGAGTACTGCGTCCAGTTCTGGGCGCCACA--
CTTCAAGAGGGACGTGGACGATATGGAAAGAGTCCAGAGGAGGGCCAC--
TCGTATGATCAGGGGTCAGCAGGGCAGGCCTTATGAGGAGAGGCTACGGACCTGAACCTGTTTAGCCTC
CACAAGAG--AAGGTTAAGGGGGGAC--------CTTGTGGCCTGCTACAAACTGGTCAGGGGG---
GATCAGCAGGTATTGGGGGAGTCCCTGTTCCCCCGTGCGCTGCCAGGAGTGACCAGGAA-
TAACGGTCACAAGCTGGCGGAGGG---
TAGGTTTAGATTAGATATTAGGAGGCGCTACTTCACAGTCAGGGCGGCTAGGGCCTGGAACCGGCTTCCA
GGGGAAGTGGTGCAGGCTCCTAGCCTGGGGA-
TTTTTAAAAGGAAGTTAGATGAGCACCTTGCCGGGGTCATCTGATTTCCTGCC--AAGG--CA-----GGGGG-
TCGGA-CTAGATGATCTACCTAGGTCCCTTCCGAC--TCT-A-
>CR1-7_Gav

TCAGCACGGAGGTGGAAAAGGATCT-
GGGAGTCATTATTGACTCCAAGATGAACATGAGTCGGCAATGTGGGGAGGCGGCTAGGAAGGCCAACTG
CACCTTGTCGTGCATCCACAGATGCATCTCGAGCAGGTCCAAGGAGGTGATCCTCCCCCTCTATGCGGCAC
TGGTCAGGCCGCAGCTGGAGTACTGCGTCCAGTTCTGGGCGCCGCA--
CTTTAAGAGGGATGTGGACAGCATCGAGAGGGTCCAGAGGAGGGCCAC--
TCGTATGATCAAGGGACAGCAGGGTAGGTCCTATGAGGAAAGGTTGCGGGACCTGAACCTGTTCAGCCTT
CAAAAGAG--AAGGCTGAGGGGGGAT--------CTTGTGGCCTGTTATAAACTGGTTAGGGGG---
GATCAGCAGGCGCTGGGGGAGTCCCTGTTCCCCCGAGCGCCGCAAGGAGTGACCAGGAA-
CAATGGTCACAAGCTGGCGGAGGG---
AAGGTTTAAGTTGGACATCAGGAGGCGCTACTTCACGGTCAGGGCGGCTAGGGTCTGGAACCAGCTTCCA
AGTGAGGTGGTGCAGGCTCCCACCCTGGGGG-
TTTTTAAGAGGAGGTTGGATGACCACCTTACTGGGGTCATTTGA-TTCCTGCC--ATGG--CA-----GGGGG-
TCGGA-CTAGATGATCTGCTCAGGTCCCTTCCGAC--CCT-A-
>CR1-8_AMi
CCAGCACGATGGCTGAAAGGGACCT-
CGGGGTTATGGTTGATCACAGGATGAACATGAGCCGCCAGTGCGACGCTGTAGTCAACAGAGCTAATAGC
ATACTGGGATGCATTAGCCGATGCGTCGCGAGCAGGGCTAAGGAAGTGATACTTCCTCTCTACTCGGCGC
TGGTGAGACCCCAGCTGGAGTACTGTGTCCAGTTCTGGGCGCCGCA--
CTTCAAAAAAGACGTGGAGAATCTTGAAAGGGTCCAGAGAAGGGCCAC--
AAGAATGATTAAGGGCCTGGAGGACAAACCTTATGAGGAAAGACTAAGGGATCTGGGACTGTTCAGCCT
GGGGAAGAG--AAGACTGAGGGGGGAC--------TTGGTGGCCGTCTATAAGTATGTAAGGGGT---
GAATATCGGGAGCTGGGAGAAAAACTGTTCACTAGGGCGCCCCAGGGGAGGACAAGGAG-
CAATGGCCATAAACTGTTAGAGGA---
TGGTTTCAGGTTGGATGTAAGGAAGAACTTCTTTACGGTAAGGGTGACCAGAATCTGGAATAGACTTCCC
AAGGAGGTGGTGCAGTCCCCAACCTTGGAAG-
TCTTCAAGAGGAGACTGGACAGACACCTTGCTGGGATCATTTGAATTCCTGCCC-AGAG--CA-----
GGGGGGTTGGA-CTCGATGATCTTTCGAGGTCCCTTCCAGC--CCT-TA
>CR1-8_CPB
GAAGCAGTGACTCTGAAAAAGATTT-
GGGGGTCATGGTGGATAATCAGCTGAACATGAGCTCCCAGTGCGATGCTGTGGCCAAAAGAGCTAATGCG
ATCCTGGGATGCATAAACAGGGGAATCTCGAGTAGGAGTAGAGAGGTTATTTTACCTCTGTATTTGGCAC
TGGTGCGACCGCTGCTGGAATACTGTGTCCAGTTCTGGTGTCCACA--
ATTCAAGAAGGATGTTGATAAATTGGAGAGGGTTCAGAGAAGAGCCAC--
GAGAATGATTAAAGGATTAGAAAACATGCCTTATAGTGATAGACTCAAGGAGCTCAATCTATTTAGCTTA
ACAAAGAG--AAGGTTAAGGGGTGAC--------TTGATTACAGTCTATAAGTACCTACATGGG---
GAACAAATATTTAATAAT---GGGCTCTTCAGTCTAGCAGAGAAAGGTATAACACGATC-
CAATGGCTGGAAGTTGAAGCTAGA---
CAAATTCAGACTGGAAATAAGGTGTACATTTTTAACGGTGAGAGTAATTAACCATTGGAACAATTTACCA
A-GGTCGTGGTGGATTCTCCATCACTGACAA-
TTTTTAAATCAAGATTGGATGTTTTTCTAAAAGATATGCTCTAGTGGCCTGTGT-TATA--CA-----GGAGG-
TCAGA-CTAGATGATC-ACAATGGTCCCTTCTGGC--CTTG--
>CR1-8_Gav
GTAGCACAACTGCGGAAAGGGATCT-
CGGAGTCGTAGTCGACTCCAAAATGAACATGAGTCACCAGTGTGATGAGGTAATAAGTAAAGCTAACCGC
ACCCTTTCTTGCATAAGTAGGTGCATCGCAAACAGGTCTAGGGAGGTGATACTTCCCCTCTATGCGGCATT
GGTCAGGCCGCAGCTGGAGTACTGCGTCCAGTTTTGGGCGCCGCA--
CTTCGGGAGGGATGTGGATGGCCTGGAGAGGGTTCAGAGGAGGGCCAC--
TCGTCTGGTTAAAGGCCTACAGAAAAAACCCTATGTGGACCGATTGGGAGACCTGAACCTCTTCAGCCTC
CGCAAGAG--GAGGCTGAGAGGTGAT--------CTCGTGGCCGCCTACAAACTCATC-GGGGG---
GGGCAGCAAGAAATAGGGGATACAATGTTTACCAGGGCGCCCGTCGGGGTAACTAGAAA-
TAATGGCCACAAACTGGAAGAGAG---
CAAATTCAGATTGGACATCAGGAAAAAATTCTTTACAGTGAGGGTTGCCAAAATATGGAATAAGCTTCCA
AGGGAGGTGGTGCTGTCCCCTTCCTTGGAGG-
TGTTTAAAAGGAGATTGGACAGACACCTGGCTGGGGTCATCTGAATTCCTGCCC-AGGG--CA-----GGGGG-

TCGGA-CTCGATGATCTAAT-AGGTCCCTTCCGAC--CCT-A-
>CR1-9B_AMi
GCAGCTCAGAGGCTGAGAGAGATCT-
TGGAGTCATAGTTGACTCCAAGATGAACATGAGCCGGCAGTGTAACGAGGCCATCAACAAGGCCAATCGC
ACCTTGTCGTGTATTAGCAGATGCATGACCAACAGATCGAGGGAGGTGATGCTCCCCCTCTATGCGGCATT
GGTCAGGCCGCAGTTGGAGTACTGTGTCCAGTTTTGGGCGCCGCA--
CTTCAAGAGGGACACGGAGAATCTGGAGAGGGTTCAGAGGAGGGCCAC--
TCGCATGATTAGTGGCCTCCGTGAAAGACCCTACGAGGGGAGGTTGAGAGATCTGGATCTCTTCAGCCTT
CACAAGAG--ACGTCTGAGGGGAGAC--------CTTGTAGCCGCCTATAAGTTTATCAAGGGA---
GGACAACAGGGAATTGG-GATGCGCTATTTACCAGAGCGCCCCAGGGAGTAACTAGGAA-
TAATGGGTGCAAACTAGTGGAGAG---
TAGATTTAGGTTAGACATTAGGAAGAAATTTTTTACAGTAAGGGTGGCCAGAATCTGGAATGGGCTTCCA
AGAGAGGTGGTGCTATCACCTAACTTGGAGG-
TCTTCAAGAGGAGGCTTGATAGTCACCTGGCTGGGGTCATCTGATTTCCTGCCA-GGGG--CA-----GGGGG-
TCGGA-CACGATGATCCATTGTGGTCCCTTCCGAC--TCT-A-
>CR1-9C_AMi
GCACCACAACAGTGGAAAGAGATCT-
CGGAGTCCTGGTTGACGCAAAAATGAACATGAGTCGCCAGTGTGACGAGGTAATAAGCAAGGCTAACTG
CACTCTTTCTTGCATCAGCAGGWGCATCACGAGCAGGTCTAGGGAAGTGATAATTCCCCTCTATGCAGCA
CTGGTGAGGCCGCAGATGGAGTACTGCGTCCAGTTTTGGGCGCCGCA--
CTTCAAGAGGGATGTGGATAACCTTGAGAGGGTTCAGAGGAGGGCTAC--
TCGTATGGTTGAGGGCCTGCAGGTAAGACCCTATGAAGACAGACTGAGGGACCTGAATCTCTTCAGCCTC
CGCAAGAG--AAGGCTGAGAGGTGAT--------CTTGTGGCTGCCTACAAACTCATCAGGGGG---
GGCCAGCAGGGAATAGGGGACACTCTGTTTACCAGGGCGCCCCTCGGGGTTACCAGAAA-
TAATGGCCACAAACTGAAGGAGAA---
CAGATTTAGATTGGACATAAGGAAGAAATTCTTTACGGTGAGGGTTGCCAAAATATGGAATGGGCTTCCA
AGGGAGGTGGTGCTTTCCCCTACCCTGGAGG-
TGTTCAAGAGGAGATTAGACAGGCATCTGGCTGGGGTTACTTAATTTCCTGCCT-GGAG--CA-----GGGGG-
TCGGA-CCCGATGATCTACC-AGGTCCCTTCCGAC--TCT-AG
>CR1-9D_AMi
GCTCCACTGAGGCAGAAAAAGATCT-
TGGAGTCACTGTTGACTCCAGGATGAACATGAGTCGCCAATGTGATGAAGCAGTTGGCAGAGCCAATCGC
ACCTTGTCGTGCATCCGTAGGTGCTTCACAAACAGGTCCAGGGAAGTGATTCTCCCCCTCTATGCAGCACT
GGTCAGACCGCAGCTGGAGTACTGTGTCCAGTTCTGGATGCCGCA--
GTTCAAGCGGGATGCGGACAACCTGGAGAGGGTCCAAAGAAGAGCCAC--
TCGCCTGATTAGAGGCCTGCAGGGAAGGCCCTATGAGGAAAGGCTAAGGGACCTTAACCTCTTCAGCCTC
TCCAAGAG--AAGGCTGAGAGGGGAC--------CTTGTGGCGGCGTACAAATTCATAAGGGGA---
GAGCAACATGAAGTAGGCGACGATCTGTTCACCAGGGCACCCCCTGGAATGACAAGGAA-
CAATGGCCATAAGCTACTAGAGAG---
AAGGTTTAGGCTGGACATTAGGAGAAAGTTCTTCACGGTCAGGGTTGCCAGAATCTGGAATGGGCTCCCA
AGGGAGGTGGTGCTCGCCCCATCCTTGGGGG-
TCTTCAAGAGGAGACTGGACAGGCACCTGGCTGGGATCACATGATTGCCTGCCA-GGGG--CA-----
GGGGG-TCAGA-CTAGATGGCCCACTGAGGTCCCTTCCAAC--TCT-AT
>CR1-9_AMi
GCAGCACAGCAGCGGAAAGGGATCT-
CGGAGTCATAGTTGACTCCAAGATGAACATGAGTCGTCAATGTGACGAAGCGATCAGTAAAGCTAACCGC
ACTTTATCATGCATTAGCAGGTGCATGACGAACAGGTCCAGGGAGGTGATACTTCCCCTCTATGCGGCACT
GGTCAGGCCGCAGTTGGAGTACTGCGTCCAGTTTTGGGCGCCGCA--
CTTCAAGAGGGATGTGGACAACCTCGAGAGGGTTCAGAGGAGGGCCAC--
TCGTATGGTTAGGGGCCTGCAGGCAAGGCCCTACGAGGAGAGACTGAGGGACCTGGATCTCTTCAGCCTC
CGCAAGAG--AAGGCTGAGAGGTGAT--------CTTGTGGCTGCCTACAAATTCATCAGGGGG---
GGGCAGCAAGGAATAGGAGATGCTCTGTTTACCAGGGCACCCCTTGGAGTAACTAGGAA-
CAACGGCCACAAACTGACGGAGAG---
CAGATTTAGGTTAGACATCAGGAAGAACTTCTTTACGGTAAGGGTTGCCAGAATCTGGAATGGGCTTCCA

AGGGAGGTGGTGCTCTCCCCTACCTTGGGGG-
TCTTTAAGAGGAGGCTGGACATGCACCTGGCTGGGGTCGTCTGATTTCCTGCCC-AGGG--CA-----GGGGG-
TCGGA-CTCGATGATCTGCTGAGGTCCCTTCCGAC--CCA-A-
>CR1-9_CPB
GTGGTAGTACTGCTGAAAAGGATCT-
GGGGGTTATAGTGGATCACAAATTGAATATGAGTCAACAATGTGATGCAGTTGCGAAAAAGGCTAATATC
ATTCTGGGGTGTATTAACAGGAGTGTCGTATGTAAGACATGGGAGGTAATTGTCCCGCTCTACTCGGCACT
GGTGAGGCCTCAGCTGGAGTACTGTGTCCAGTTCTGGGCGCCACA--
CTTTAGGAAAGATGTGGACAAATTGGAGAGAGTCCAGAGGAGAGCAAC--
AAAAATGATAAAAGGTTTAGAAAACCTGACCTATGAGGAAAGGTTAAAAAAACTGGGCATGTTTAGTCTT
GAGAAAAG--AAGACTGAGGGGGGAC--------CTGATAACAGTCTTCAAATATGTTAAGGGC---
TGTTATAAAGAGGACGGTGATCAATTGTTCTCCATGTCCACTGAAGGTAGGACAAGAAG-
TAATGGGCTTAATCTGCAGCAAGG---
GAGATTTAGGTTAGATATTAGGAAAAACTTTCTAACTATAAGGGTAGTTAAGCTCTGGAATAGGCTTCCA
AGGGAGGTTGTGGAATCCCCGTCACTGGAGG-
TTTTTAAGAACAGGTTGGACAAACACCTGTCAGGGATGGTCTAGGGTCCTGCCTCAGCG--CA-----GGGGG-
CTGGA-CTTGATGACCTCTCAAGGTCCCTTCCAGC--CCTACA
>CR1-9_Crp
CCAGCACCATGAATGAAAGGGACCT-
GGGGGTAATAATAGACCACAGTATGAACATGAGCCGGCAGTGTGATGCTGTAGCCAGCAGGGCAAACAA
TACCCTGGCATGCATCAATCGATGCATCTCCAGCAAAACCAAGGAAGTGATTCTTCCACTCTACTCGGCAT
TGGTGAGACCACAGCTGGAGTACTGCATCCAGTWCTGGGTGCCGCA--
CTTCAACAAGGACATGGTAAAGCTTGAGAGAGTCCAGAGAAGGGCCAC--
CCATATGATCAGAGACTTGCATGGCAAGCCATACGAGGAAAGGCTGAGGGATCTGGGACTCTTCAGCCTG
AAAAAGAG---AGGCTGAGAGGGGAC--------TTGGTAGCAGCTTACCACTACATCAGGGGA---
ATACATCAAGGGCTCGGTGAACAACTGTTCACCAGGGCACCCTTGGGGAAAACCAGGAG-
TAATGGCCACAAACTCCTGGAAGA---
CCATTTCAGGCTCAACATTAGGAAAAACTTCTTCACAGTTAGGGTGTCCAGACTGTGGAATAAGCTCCCTC
CAGAGGTGGTGCAATCGCCTACCCTGGAAA-
TCTTCAAGAGGAGACTGGACAGTCACCTTGCTGGGGTCACCTGATTTCCTGCCT-TGTG--CA-----GGGGG-
CTGGA-CCCGATGATCTTCCAAGGTCCCTTCCAGC--CTT--A
>CR1-B
NNNNNNNNNNNNNNNNNNNNNNNNN-
NNNNNNNNNNNNNNNNNNNNNNNNNNNNNNNNNNNNNNNNNNNNNNNNNNNNNNNNNNNNNNNNNNNN
NNNTCTCCTGGGCTCCATCATGAGAGGGGTGGCCAGCAGGGACCCTTTGGAGATTGTCCCTCTTTACTCTG
CTCTTGTGAGGCCCCTTTAAAAGTACTGTGTCCTTGTATGGAGCCCACA--
GTACAAGAAAGACAGAGAGCTGTTGGAGAGGGTCCAGAGGAGGGCCAT--
GAAGATGATCAGGGGGCTGGAGCACCTCCCCTATGAAGACAGGCTGAAGGAGCTGGTCTTATTCAGCCTG
GAGAAGAG--AAGGCTGCGGGGTGAC--------CTCATTGCAGCCTTTCAGTACCTGAAGGGA---
GCCTATAAACAGGAAGGGAGTAAACTCTTTGAAAGGGTAGATAACAGCAGGACAAGGGG-
GAACGGTTTTAAGTTGAAAGAGGG---
AAGATTTAGGTTGGATGTTAGGGGGAAGTTCTTTACCAGGAGAGTGGTGAGGTGCTGGAATAGGCTGCCC
TGAGAGGTTGTGGATGCCCCGTCCCTGGAGG-
TGTTCAAGGCCAGGTTGGATGAGGCCCTGGGCAACCTGGTCTAAGGCCCTGCC---TGG--CT-G---GGGGG-
TTGGAGATTCATGATC-CTTGAGGTCCCTTCCAAC--CCAGGC
>CR1-B2
AGAGTAGCCCTGCAGAGAAGGACCT-
GGGGGTCCTGGTGGATGAAAAACTTAACATGAGCCAGCAGTGTGCTCTTGCAGCTCGGAAAGCAAATGGT
ATCCTGGGCTCCATCAGAAGAGGGGTGGCCAGCAGGGACAGGGAGGTGATTGTCCCTCTCTACTCTGCCC
TTGTGAGGCCCCATCTGGAGTACTGCGTCCAGGTCTGGAGCCCCCA--
GTACAAGAAAGACAGGGAGCTGTTGGAGAGGGTCCAGAGGAGGGCCAC--
AAAGATGATCAGAGGGCTGGAGCACCTCCCCTACGAAGACAGGCTGAGGGAGCTGGGCTTGTTCAGCCTG
GAGAAGAG--AAGGCTGCGGGGTGAC--------CTCATTGCAGCCTTTCAGTACCTAAAGGGA---
GCCTATAAACAGGAGGGGAGTCAACTCTTTGAAAGGGTAGATAACAGCAGGACAAGGGG-

AAATGGTTTTAAGTTGAAGGAGGG---
AAGATTTAGGTTGGATGTCAGGGGGAAGTTCTTTACTATGAGAGTGGTGAGGTGCTGGAACAGGCTGCCC
AGAGAGGTTGTGGATGCCCCGTCCCTGGAGG-
TGTTCAAGGCCAGGTTGGATGGGGCCCTGGGCAGCCTGGTCTAGGGCCCTGCCT-GTGG--CA-G---
GGGGG-TTGGAGNTTCATGATC-CTTGAGGTCCCTTCCAAC--CCGGGC
>CR1-C
AGAGCAGCCCTGCGGAGAAGGACTT-
GGGGGTCCTGGTGGACGAGAAGCTGGACATGAGCCAGCAGTGTGCGCTTGCAGCCCGGAAGGCCAACTG
TGTTCTGGGCTGCATTAAAAAAGGGGTGGCCAGCAGGGAGAGGGAGGTGATTGTCCCCCTCTACTCAGCT
CTTGTGAGGCCCCATCTGGAGTACTGCGTCCAGGCCTGGGGCCCCCA--
GTACAGGAAGGACGTGGAGCTCTTGGAGCGGGTCCAGAGGAGGGCCAC--
TAAGATGATCAGAGGGCTGGAGCACCTCTCCTATGAGGAAAGGTTGAGGGAACTGGGCTTGTTTAGCTTG
GAGAAGAG--AAGGCTCCGGGGAGAC--------CTCATTGTGGCCTTCCAGTACTTGAAGGGA---
GCGTATAAACAGGAGGGGGAACGGCTGTTTACGAGGGTGGATAGTGATAGGACAAGGGG-
GAATGGTTTTAAACTGAGACAGGG---
GAGGTTTAGGTTAGATATTAGGAGGAAGTTTTTCACACAGAGGGTGGTGACGCACTGGAACAGGTTGCCC
AAGGAGGTTGTGGATGCCCCATCCCTGGAGG-
CATTCAAGGCCAGGCTGGATGTGGCTCTGGGCAGCCTGGTCTGGGACCCTGCAC-ATAG--CA-G---
GGGGG-TTGAAACTNGATGATC-ATTGTGGTCCTTTTCAAC--CCAGGC
>CR1-C2
AGAGCAGCCCTGCGGAGAAGGACTT-
GGGGGTCCTGGTGGACGAGAAGCTGGACATGAGCCAGCAGTGTGCGCTTGCAGCCCGGAAGGCCAACTG
TGTTCTGGGCTGCATTAAAAAAGGGGTGGCCAGCAGGGAGAGGGAGGTGATTGTCCCCCTCTACTCAGCT
CTTGTGAGGCCCCATCTGGAGTACTGCGTCCAGGCCTGGGGCCCCCA--
GTACAAGAAGGACGTGGAGCTCTTGGAACGGGTCCAGAGGAGGGCCAC--
TAAGATGATCAGAGGGCTGGAGCACCTCTCCTATGAGGAAAGGTTGAGGGAACTGGGCTTGTTTAGCTTG
GAGAAGAG--AAGGCTCCGGGGAGAC--------CTCATTGTGGCCTTCCAGTACTTGAAGGGA---
GCGTATAAACAGGAGGGGGAACGGCTGTTTACGAGGGTGGATAGTGATAGGACAAGGGG-
GAATGGTTTTAAACTGAGACAGGG---
GAGGTTTAGGTTAGATATTAGGAGGAAGTTTTTCACTCAGAGGGTGGTGACGCACTGGAACAGGTTGCCC
AAGGAGGTTGTGGATGCCCCATCCCTGGAGG-
CATTCAAGGCCAGGCTGGATGTGGCTCTGGGCAGCCTGGTCTAGGACCCTGCNC-ATAG--CA-G---
GGGGG-TTGAAACTAGATGATC-NTTGAGGTCCTTTTCAAC--CCAGGC
>CR1-C3
AGAGTAGCCCTGCTGAGAAGGACTT-
AGGGGTCCTGGTAGATGAAAAACTTAACATGAGCCAGCAGTGTGCGCTTGCAGCCCAGAAGGCCAATGG
TATCCTGGGCTGCATCAGAAGAGGGGTGGCCAGCAGGGCGAGGGAGGTGATTGTCCCCCTCTACTCTGCC
CTCGTGAGGCCCCATCTGGAGTACTGCGTCCAGGTCTGGGGCCCCCA--
GCACAGGAAAGATGTGGAGCTTTTGGAGNGGGTCCAGAGGAGGGCCAC--
GAAGATGATCNGAGGGCTGGAGCACCTCTCCTATGAAGACAGGCTGAAGGAGCTGGGCTTGTTCAGCCTG
GAGAAGAG--AAGGCTGCGGGGAGAC--------CTCATTGCGGCCTTCCAGTATTTAAAGGGA---
GNTTATAAACAGGAGGGAAATCAACTTTTTACACGGGTAGATAGTGATAGGACAAGGGG-
GAATGGTTTTAAACTAAAGGAGGG---
AAGATTTAGATTAGATGTCAGGGGGAAGTTTTTCACTGAGAGGGTGGTGAGGTGCTGGAACAGGTTGCCC
AGAGAGGCTGTGGATGCCCCGTCCCTGGAGG-
TGTTCAAGGCCAGGTTGGATGGGGCCCTGGGCAACCTGATCTAGAACCCTGCCT-GTGG--CA-G---
GGGGG-TTGGAACTTGATGATC-CTTGAGGTCCCTTCCAAC--CCAAGC
>CR1-C4
AGAGCAGCCCTGCGGAGAAGGACTT-
GGGGGTCCTGGTGGACGAAAAGCTGGACATGAGCCAGCAGTGTGCNCTTGCAGCCCGGAAGGCCAACAG
TATCCTGGGCTGCATCAAAAGAGGGGTGGCCAGCAGGGAGAGGGAGGTGATTGTCCCCCTCTACTCTGCC
CTTGTGAGGCCCCATCTGGAGTACTGCGTCCAGGCCTGGGGCCCCCA--
GCACAAGAAGGATGTGGAGCTGTTGGAGCGGGTCCAGAGGAGGGCCAC--
GAAGATGATCAGAGGGCTGGAGCACCTCTCCTATGAAGAAAGGTTGAGGGAGCTGGGCTTGTTCAGCTTG

GAGAAGAG--AAGGCTCCGGGGAGAC--------CTCATTGCGGCCTTCCAGTACTTGAAGGGA---
GCTTATAAGCAGGAGGGGGACCGACTTTTTACACGGTCTGATAGTGATAGGACAAGGGG-
GAATGGCTTTAAACTAAAAGAGGG---
GAGATTTAGGTTAGATGTTAGGAAGAAATTCTTTACTCAGAGGGTGGTGAGGCGCTGGCACAGGCTGCCC
AGAGAAGCTGTGGATGCCCCATCCCTGGAGG-
CGTTCAAGGCCAGGTTGGATGGGGCCCTGGGCAGCCTGATCTGGAGCCCTGCCC-ATGG--CA-G---
GGGGG-TTGGAACTAGATGATC-TTTAAGGTCCCTTCCAAC--CTAAGC
>CR1-D
AGCGCAGCCCTGCCGAAAAAGACCT-
GGGGGTACTGGTGGATGGGAAGCTGGACATGAGCCAGCAATGTGCCCTCGCAGCCCAGAAAGCCAACCG
TATCCTGGGCTGCATCAAAAGAAGCGTGGCCAGCAGGTCGAGGGAGGTGATCCTGCCCCTCTACTCTGCG
CTGGTGAGGCCTCACCTGGAGTACTGCGTCCAGATGTGGAGTCCTCA--
GTACAGGAGAGACATGGACCTGTTGGAGCGCGTCCAGAGGAGGGCCAC--
AAAAATGATCCAAGGGATGGAACACCTCTCCTACGAGGACAGGCTGAGAGAGCTGGGGCTGTTCAGCCT
GGAGAAGAG--AAGGCTNCGAGGTGAC--------CTGATAGCGGCCTTTCAGTATCTAAAGGGG---
AGCTACAGGAAAGAAGGGGACAGACTCTTTAGCAGGGTCTGTGGTGATAGAACAAGGGG-
AAATGGCTTCAAGCTCAAAGAGGG---
TAGATTTAGGTTGGATATAAGGAAAAAGTCTTTTACAGTGAGGGTGGTGAGGCACTGGAACAGGTTGCCC
AGAGATGTGGTTGATGCCCCGTCCCTGGAGA-
CTTTCAAGGCGAGGCTGGATCAGGCCCTGGGCAACCTGATCTAGGTCCCTGTTC-ATTG--CA-G---GGGAG-
TTGGA-CTAGATGACCTTTAAAGGTCCCTTCCAAC--TCTAAG
>CR1-D2
AGCACAGCCCTGCCGAAAAGGACCT-
GGGGGTACTGGTGGATGGCAAGCTGGACATGAGCCAGCAATGTGCCCTCGCAGCCCAGAAAGCCAACCG
TATCCTGGGCTGCATCAAAAGAAGCGTGGCCAGCAGGGCGAGGGAGGTGATCCTGCCCCTCTGCTCTGCG
CTGGTGAGGCCTCACCTGGAGTACTGCGTCCAGATGTGGAGTCCTCA--
GTACAGGAGAGACATGGACCTGTTGGAGCGCGTCCAGAGGAGGGCCAC--
AAAAATGATCCAAGGGATGGAACACCTCCCCTACGAGGACAGGCTGAGAGAGCTGGGGCTGTTCAGCCT
GGAGAAGAG--AAGGCTCCGGGGAGAC--------CTGAGAGCGGCCTTTCAGTATCTAAAGGGG---
GGCTGTAAGAAAGAAGGGGACAGACTCTTTAGCAGGGTCTGTTGTGACAGGACAAGGGG-
AAATGGTTTCAAACTAAAAGAGGG---
GAGATTTAGATTGGATATAAGGAAGAAGTTTTTTACAATAAGGGTGGTGAGGCACTGGAACAGGTTGCCC
AGAGAGGTGGTGGATGCCCCATCCCTGGAGA-
CATTCAAGGTCAGGCTGGACGGGGCTCTGAGCAACCTGATCTAGGTCCCTGTTC-ATTG--CA-G---GGGAG-
TTGGA-CTAGATGACCTTTAAGGGTCCCTTCCAAC--TCAAAC
>CR1-E_Pass
AGAGCAGCCCTGCGGAGAAGGACTT-
GGGGGTGATGGTTGATGAAAAACTCAACATGAGCCGGCAGTGTGCGCTCGCAGCCCAGAAAGCCAANCG
NATCCTGGGCTGCATCCAAAGGAGCGTGGCCAGCAGGTCGAGGGAGGNGATTCTGCCCCTCTGCTCTGCT
CTTGTGAGACCCCACCTGGAGCGCTGCGTCCAGCTCTGGTGTCCCCA--
GCATAAGAAGGACATGGAACTGTTGGAGCAAGTCCAGAGGAGGGCCAC--
GAAGTTGATAAGAGGACTGGAGCACCTCCCCTACGAAGACAGGCTGAGAAAGTTGGGGCTGTTCAGCCTG
GAGAAGAG--AAGGTTGCGTGGAGAC--------CTCATAGCAACCTTCCAGTATCTGAAGGGG---
GCCTACAGGGAAGCCGGAGAGGGACTCTTCGTCAGGAACTGTAGTGATAGGACAAGGAG-
TAATGGGTACAAACTGAAAGAGGG---
GAAATTTAGGTTAGATATTAGGAAGAAATTCTTTACTGTGAGGGTGGTGAGACACTGGAACAGGTTGCCC
AGGGAGGTTGTGGATGCCCCANCCCTGGCAG-
TGTTCAAGGCCAGGTTGGATAAGGCCTTGAGCAACCTGGTCTAGGTCCCTGCCC-ATGG--CAGG---
GGGGG-TTGGGACTAGATGATC-TTTAAGGTCCNTTCCAAC--CCTTAA
>CR1-F
AAGACTGTGTAGAGGAAATGGACCT-
GGGGGTGTTGATTGACACTAGACTGAACATGAGCCAGTGGTGTGCCCAGGTGGCCAAGAAGGCCAATGG
CATCCTGGCTTGTATCAGAAATAGTGTGGCCAGCAGGAACAGGGAAGTAATTGTCTCCCTGTACTCAGCA
CTGGCGAGGCTGCACCTTGAGTACTGTGTCCAGTTTTGGGCCCCTCA--

CTGTAAGAAAGACATTGAGGCCCTGGAGTGTGTCCAGAGGAGGGCAAC--
AAAGCTGGTGAGGGGTCTGGAACACAGGCCTTATGAAGAGTGGCTGAAGGAGCTGGGATTGTTCAGTCTG
GAGAAGAG--GAGGCTCAGGGGAGAC--------CTTATTGCTCTCTATAACTACCTGAAGGGA---
GGTTGTAGTGAGCTCGGGGTCAGCCTCTTCTCTCGTGTGAC-AGTGATAGAACGAGAGG-
GAATGGCTTCAAGCTGCGCCAGGG---
AAGATTCAGGCTGGACATTAGGAAATACTACTTCTCTGAAAGGGTGGTCAGGCACTGGAATGGGCTGCCC
AGAGTGGTGGTGGAGTCACCGAGCCTGGTGG-
TGTTTAAAGAGCATTTGGATGTTGTGTTGAGGGACATGGTTTAGCCATTGGTGA-AGGG--CG-----AATGG-
TTGGA-CTGGATC----CTGTGGGTCTTTTCCAAC--CTTAGC
>CR1-F2
AAGACTGTGTAGAGGAAATGGACCT-
GGGGGTGTTGATTGACGCTNGACTGAACATGAGCCAGCAGTGTGCCCAGGTGGCCAAGAAGGCCAATGG
CATCCTGGCTTGTATCAGAAATAGTGTGGCCAGCAGGAACAGGGAAGTAATTGTCCCCCTGTACTCAGCA
CTGGTGAGGCCGCACCTCGAGTACTGTGTCCAGTTTTGGGCCCCTCA--
CTGTAAGAAAGACATCGAGGCCCTGGAGCGTGTCCAGAGGAGGGCAAC--
AAAGCTGGTGAGGGGTCTGGAGCACAGGCCTTATGAGGAGCGGCTGAAGGAGCTGGGATTGTTCAGTCTG
GAGAAGAG--GAGGCTCAGGGGAGAC--------CTTATTGCTCTCTATAACTACCTGAAGGGA---
GGTTGTAGTGAGCTGGGGGTCGGCCTCTTCTCTCGTGTGACTAGTGATAGGACTAGAGG-
GAATGGCTTCAAGCTGCGCCAGGG---
AAGGTTCAGGCTGGACGTTAGGAAATACTACTTCTCTGAAAGGGTGGTCAGGCACTGGAATGGGCTGCCC
AGGGAGGTGGTGGAGTCACCGACCCTGGAGG-
TGTTCAAGGAACGTTTGGATGTTGTGTTGAGGGACATGGTTTAGCTATTGGTGA-TGGG--TG-----GATGG-
TTGGA-CTGGGTGATC-CTGTGGGTCTTTTCCAAC--CTTGGT
>CR1-G
ATGACTGTGAAGAGGAAAGGGACCT-
GGGGGTGTTGGTTGATGCTCGGCTGAACATGAGCCGACAGTGTGCCCAGGTGGCCAAGAGGGCCAATGCC
ATCCTGGCCTGCATTAGAAATAGTGTGGCCAGCAGGAGCAGGGAGGTAATCATCCCCCTGTACTCAGCAC
TGGTGAGGCCGCACCTCGAGTACTGTGTTCAGTTTTGGGCCCCTCA--
CTACAAGAAAGACATTGAGGCCCTGGAACGTGTCCAGAGAAGGGCAAC--
GAAACTGGTGAGGGGTCTGGAGCACAAGTCTTATGAGGAGCGGCTGAGGGAGCTGGGATTGTTTAGTCTG
GAGAAGAG--GAGGCTCAGGGGAGAC--------CTCATTGCACTCTACAACTTCCTGAAGGGA---
GGTTGTGATGAGGAGGGGTTTGGCCTCTTCTCCCAGGCAACAA---ACAGGACCCGAGG-
AAATGGCCACAAGTTGTACCAGAG---
GAGGTTTAGATTAGACATAAGGAAAAACTTTTTCTCTCAGAGAGTGGTCAGGCACTGGAATGGCCTGCCC
AGGGAGGTGGTGGAGTCGCCGTCCCTGGCAG-
TGTTCAAGAGGCGTCTGGATGAGGAGCTACGAGATATGGTTTAGGCAATGGTAA-TGGG--AG-----
GACGG-TTGGA-CTAGATGATC-TTGTAGGTCCTTTCCAAC--CTTG-T
>CR1-H
AAAGCTGCCTGATGGAAAGGGACCT-
TGGTGTACTGATGGACAGTCGGCTGAATATGAGCCAGCAGTGTGCCCAGGTGGCCAAGAAGGCCAATGGC
ATCCTGGCTTGTATCAGGAATGGTGTGGTGAGCAGGACTAGGGAAGTCATCCTGCCCCTGTACTCGGCAC
TGGTGAGGCCTCACCTCGAGTACTGTGTTCAGTTTTGGGCACCTCA--
GTACAGAAAGGACATTGAGGTGCTGGAGCAGGTCCAAAGAAGGGCAAC--
AAGGCTTGTGAAGGGCTTGGAGAATATGCCCTACGAGGAGAGACTGAAGGAACTGGGGCTGTTTAGTCTG
GGGAAAAG--GAGGCTGAGGGGAGAC--------CTTATTGCTCTCTTCCAATATCTGAAAGGT---
GCTTACAGCGAGAGCGGGGTTGGTCTCTTCTCACTGGTGACAGGTGACAGGACGAGGGG-
AAATGGCCTCAAGTTGCGCCAGGG---
TAAGTTTAGGTTGGATATCAGGAAANACTTCTTTACAGAAAGGGTTGTTAAGCACTGGAATAGGCTCCCC
AGGGAGGTGGTTGAGTCACCATCCCTGGATG-
TGTTTAAAAACCGTTTGGATGTGGTGCTCAGGGACATGATTTAGGGTAGTATGG-TTAG--GT-----CGTGG-
TTGGA-CTCGATGATC-TTTAAGGTCTTTTCCAAC--CTGAGC
>CR1-H2
AAAGCTGCCTGATGGAAAGGGACCT-
TGGTGTACTGTTGGACAGTCGGCTGAATATGAGCCAGCAGTGTGCCCAGGTGGCCAAGAAGGCCAATGGC

ATCCTGGCTTGTATCAGGAATGGTGTGGTGAGCAGGACTAGGGAAGTCATCCTGCCCCTGTACTCGGCATT
GGTGAGGCCTCACCTCGAGTACTGTGTTCAGTTTTGGGCACCTCA--
GTACAAGAAGGACATGGAGGTACTGGAGCAGGTCCAGAGAAGGGCAAC--
GAGGCTAGTGAAGGGCTTGGAAAATCAGCCCTATGAGGAGAGGCTGAGGGAGCTGGGGCTGTTTAGTCT
GGGGAAGAG--GAGGCTGAGGGGAGAC--------CTTATTACTCTCTTCCAGTACCTGAAAGGT---
GTTTACAGTGAGAGCGGGGTAGGTCTCTTCTCACTGGTGACAGGTGACAAGACGAGGGG-
AAATGGCCTCAAGTTGCGCCAAGG---
TAAGTTTAGGTTGGACGTTAGGAAACACTTCTTTACAGAAAGGGTGGTTAAGCACTGGAATAGGCTCCCC
AGGGAGGTGGTTGAGTCACCGTCCCTGGATG-
TGTTTAAGAGCCGTTTGGATGTGGTGCTCAGAGATATGATTTAGGGTACTATGG-TTAG--GC-----TGCGG-
TTGGA-CTTGATGATC-TTTGAGGTCCTTTCCAAC--CTGAGT
>CR1-I_Tgu
AGAGCTGTTCGGCAGAAAGGGACCT-
GGGGGTGCTGGTCGACAGCCGGCTGAACATGAGCCAGCAGTGTGCCCAGGTGGCCAAGAAGGCCAATGG
CATCCTGGCCTGTATCAAGAATAGTGTGGCCAGCAGGACCAGGGNAGTGATCGTCCCCCTGTACTCGGCA
CTGGTGAGGCCGCACCTCGAGTGCTGCGTTCAGTTCTGGGCCCCTCA--
CTTTAAAAAGGACATTGAGGTGCTGGAGCGTGTCCAGAGAAGGGCAAC--
AAGGCTCGTGAAGGGTCTAGAAAACGTGTCTTATGAGGAACGGCTGAGGGAGCTGGGTTTGTTTAGTCTC
GAGAAGAG--GAGGCTCAGGGGGGAC--------CTCATCGCTCTCTACAACTNCCTGAAAGGA---
GGTTGTAGTGAGGTGGGGGCCGGTCTCTTCTGCCGTGCCTGCAGTGAGAGGACCAGAGG-
AAATGGCCTTAAGCTGAGACAGGG---
GAGATTCAGATTAGATATTAGAAAAAAANTTTTCACTGTTAGGGTGGTCAGGCATTGGAATAGGTTGCCC
AGGGAGGTGGTGGAGTCACCATCCCTGGAGG-
TGTTTAAGAGGCGTCTGGATCTGGCGCTGGGTGATGTGGTTTAGTA----GTGC-TGGG--TG-----GACGG-
TTGGA-CTNGATGATC-TTAAAGGTCTCTTCCAAC--CTTGAT
>CR1-J1_Pass
AAAGCTGCCCAGCGGAAAAGGACCT-
GGGGGTGCTGGTCGACAGCCGGCTGAACATGAGCCAGCAGTGTGCCCAGGTGGCCAAGAAGGCCAATGG
CATCCTGGCCTGTATCAGCAATAGTGTGGCCAGCAGGACCAGGGCAGTGATTGTCCCCCTGTACTCGGCA
CTGGTGAGGCCACACCTCGAGTNCTGTGTCCAGTTCTGGGCCCCTCA--
CTNCAAGAAGGACATTGAGGTGCTGGAGCGTGTCCAGAGAAGGGCAAC--
GGAGCTGGTGAAGGGTCTGGAGCACAAGTCCTGTGAGGAGCGGCTGAGGGAGCTGGGGNTGTTTAGCCT
GGAGAAAAG--GAGGCTCAGGGGAGAC--------CTTATCGCTCTCTACAACTGCCTGAAAGGA---
GGNTGTAGCCAGGTGGGGGTCGGCCTCTTCTCCCAGGCAACCAGCGACAGGACGAGAGG-
ACATGGCCTCAAGCTGCGCCAGGG---
GAGGTTCAGGTTGGACATCAGGAAGAATTTCTTCACGGAAAGGGTGGTTAAGCATTGGAACGGGCTGCCC
AGGGAGGTGGTGGAGTCACCATCCCTGGAGG-
TGTTCAAGAAACGACTGGACGTGGCACTTAGTGCTATGGTCTA-ANGGTGGTGT-TCGG--TC-----AAAGG-
TTGGA-CTCGATGATC-TCGGAGGTCTTTTCCAAC--CTNAAT
>CR1-J2_Pass
AAAGCTGCCCGGCGGAAAAGGACCT-
GGGGGTGCTGGTCGACAGCCGGCTGAACATGAGCCAGCAGTGTGCCCAGGTGGCCAAGAAGGCCAATGG
CATCCTGGCCTGTATCAGCAATAGTGTGGCCAGCAGGACCAGGGCAGTGATTGTCCCCCTGTACTCGGCA
CTGGTGAGGCCGCACCTCGAGTGCTGTGTCCAGTTCTGGGCCCCTCA--
CTTCAGGAAGGACATTGAGGTGCTGGAGCGTGTCCAGAGAAGGGCAAC--
GGAGCTGGTGAAGGGTCTGGAGCACAAGTCCTATGAGGAGCGGCTGAGGGAGCTGGGGTTGTTTAGCCTG
GAGAAAAG--GAGGCTCAGGGGAGAC--------CTTATCGCTCTCTACAACTGCCTGAAAGGA---
GGTTGTAGCCAGGTGGGGGTCGGTCTCTTCTCCCAGGCAACAAGCGACAGGACGAGAGG-
AAACGGCCTCAAGTTGCGCCAGGG---
GAGGTTTAGATTGGATATTAGGAAAAATTTCTTCACTGAAAGGGTGGTCAGGCATTGGAACGGGCTGCCC
AGGGAGGTGGTGGAGTCACCGTCCCTGGAGG-
TGTTCAAAAGACGTGTGGATGTGGCACTTGGGGACATGGTTTAGATGGCGGTGC-TGGG--TT-----AACGG-
TTGGA-CTCGATGATC-TTAGAGGTCTTTTCCAAC--CTAAAT
>CR1-J3_Pass

ACAGCGGCCAGGCAGAAAGGGACCT-
GGGGGTGCTGGTCGACAGCNGGCTGAACATGAGCCAGCAGTGTGCCCAGGTGGCCAAGAAGGCCAATGG
CATCCTGGCCTGTATCAGGAATAGTGTGGCCAGCAGGACCAGGGAAGTGATTCTTCCCCTGTACTCGGCA
CTGGTGAGGCCGCACCTCGAGTGCTGTGTCCAGTTCTGGGCCCCTCA--
GTTCAGGAAGGACGTTGAGGTGCTGGAGCGTGTCCAGAGAAGGGCAAC--
GAGGCTGGTGAAGGGTCTGGAGCACAAGTCCTGTGAGGAGCGGCTGAGGGAGCTGGGGTTGTTTAGCCTG
GAGAAAAG--GAGGCTCAGGGGAGAC--------CTTATCACTCTCTACAACTNCCTGAAAGGA---
GGNTGTAGCCAGGTGGGGGTCGGTCTCTTCTCCCAGGCAACCAGCGACAGGACGAGAGG-
ACACAGTCTTAAGCTGCGCCAGGG---
GAGGTTTAGGTTGGACATTAGGAAGAANTTCTTCACAGAAAGGGTGATTGGGCATTGGAATGGGCTGCCC
AGGGAGGTGGTGGAGTCACCGTCCCTGGAGG-
TGTTTAAGGAAAGACTGGACGTGGCACTCAGTGCCATGGTCTA-AAGGTGGTGT-TNGG--TC-----ATAGG-
TTGGA-CTCGATGATC-TCAGAGGTCTTTTCCAAC--CTAGTT
>CR1-K1_Tgu
ACAGTGCCCAGGCAGAAAGGGACCT-GGGGGCGCTGGTCGACAGC-
GGCTGGACATGAGCCAGCAGTGTGCCCTGGTGGCCAA-AAGGCCAGTGGC-
TCCTGGCCTGGGTCAGGAATGGTGTGGCCAGCAGGAGCAGGGAGCTCATCCTGCCCCTGTGCTGGGCACT
GCTGAGGGCACACCTCGAGTGCTGCGCCCAGCTCT-GGCCCCTCA--
GTTTGGGAAGGACGTTGAGATGCTCGAGCACGTCCAGAGGA-GGCAGC--
GAGGCTGGAGAGGGGCTGGGAACACAAACCCTGTGAGGAACCCCTGAGGGAGCTGGGGGTGCTCAGCCT
GGAGAAAAG--GAGACTCAGGGGTGAC--------CTCATCACTCTCCGCAGCT-CCTGAAAGGT---
GGCTGTGCTCAGCT-GGGGCTGGGCTCTTTCTCCAGG----AACTGACAGAACCAGAGC-
ACACAGCCTCGAGCTGCGCCAAGG---
GAAATNCAGGTTGGATATTAGGAAAAAGTTTTTACAGAAAGGGTGATAAAGTTCTGGAATGG-
CTGCCCGGGGAGGTGGTGGAGTCACCATCCCTGGGTG-TGTTTAACAAA-
GCCTGGATGTGGCACTGGGTGCCAGGGTTNAG-TTGAGGTGT-TGGG--GC------TGGG-TTGGA-
CTCGATGATC-TTGAAGGTCTCTTCCAAC--CCGG-T
>CR1-K2_Tgu
ACAGTGCCCAGGCAGAAAGGGACCT-
GGGGGCGCTGGTCAACAGCNGGCTGAACGTGAGCCAGCAGTGTGCCCTGGTGGCCAAGAAGGCCAATGG
C-
TCCTGGCCTGGATCAGGAATGGTGTGGCCAGCAGGAGCAGGGAGGTCATTCTGCCCCTGTACTCGGCACT
GGTGAGGCCACACCTCGAGTGCTGTGTCCAGTTCT-GGCCCCTCA--
GTTTGGGAAGGACGTTGAGACGCTCGAGCGCGTCCAGAGGA-GGCAAC--
GAGGCTGGAGAGGGGCTGGGAACACAAACCCTGTGAGGAACCGCTGAGGGAGCTGGGGGTGCTCAGCCT
GGAGAAAAG--GAGACTCAGGGGTGAC--------CTCGTCACTCTCCACAGCT-CCTGAAAGGT---
GGCTGTGCTCAGCT-GGGGTTGGGCTCTTTCTCCAGGCAGC-ACTGACAGAACCAGAGG-
ACACAGCCTCAAGCTGCGCCAAGG---
GAAATATAGGTTGGATATTAGGAAAAAGTTTTTCACAGAAAGGGTGATAAAGTTCTGGAATGG-
CTGCCCGGGGAGGTGGTGGAGTCACCATCCCTGGGTG-
TGTTTAAAAAAAAGCCTGGATGTGGCACTGGGTGCCAGGGTTTAG-TTGAGGTGT-TGGG--GC------TGGG-
TTGGA-CTCGATGATC-TTGAAGGTCTCTTCCAAC--CCGG-T
>CR1-K3_Tgu
ACAGTGCCCAGGCAGAAAGGGACCT-
GGGGGCACTGGTCGACAGCCGGCTGAACATGAGCCAGCAGTGTGCCCTGGTGGCCAAGAAGGCCAATGG
C-
TCCTGGCCTGGATCAGGAATGGTGTGGCCAGCAGGAGCAGGGAGGTCATTCTTCCCCTGTACTCGGCACT
GGTGAGGCCACACCTCGAGTGCTGTGTCCAGTTCT-GGCCCCTCA--
GTTTGGGAAGGACGTTGAGACGCTCGAGCGCGTCCAGAGGA-GGCAAC--
GAGGCTGGAGAGGGGCTGGGAACACAAACCCTGTGAGGAACGACTGAGGGAGCTGGGGGTGTTCAGCCT
GGAGAAAAG--GAGACTCAGGGGTGAC--------CTTATCACTCTCTACAACTCCCTGAAAGGT---
GGCTGTGGTCAGGT-GGGGTTGGTCTCTTTCTCCAGGCAGCAACTGACAGAACGAGAGG-
ACACAGTCTCAAGCTGCGCCAAGG---
GAAATATAGGTTGGATATTAGGAAAAAGTTTTTCACGGAAAGAGTGATAAAGTACTGGAATGGTCTGCCC

```
GGGGAGGTGGTGGAGTCACCATCCCTGGATG-
TGTTTAAAAAAAGACTGGATGTGGCACTCGGTGCCATGGTTTAG-TTGAGGTGT-TAGG--GC-----ATGGG-
TTGGA-CTCGATGATC-TTGAAGGTCTCTTCCAAC--CTAG-T
>CR1-K4_Tgu
ACAGTGCCCAGGCAGAAAGGGACCT-
GGGGGTGCTGGTCGACAGCCGGCTGAACATGAGCCAGCAGTGTGCCCTGGTGGCCAAGAAGGCCAANGG
CATCCTGGCCTGTATCAGGAATGGTGTGGCCAGCAGGAGCAGGGAGGTCATTCTCCCCCTGTACTCGGCA
CTGGTGAGGCCACACCTTGAGTGCTGTGTCCAGTTCTGGGCCCCTCA--
GTTTGGGAAGGACGTTGAGACGCTTGAGCGCGTCCAGAGGAGGGCAAC--
GAGGCTGGTGAGGGGCTTGGAACACAAACCCTGTGAGGAACGGCTGAGGGAGCTGGGGGTGTTTAGCCT
GGAGAAAAG--GAGACTCAGAGGTGAC--------CTTATCACTCTCTACAACTCCCTGAAAGGT---
GGCTGTAGTCAGGTGGGGGTTGGTCTCTTTCTCCAGGCAGCAACTGACAGAACGAGAGG-
ACACAGTCTCAAGCTGCGCCAAGG---
GAAATATAGGTTGGATATTAGGAAAAAGTTTTTTACGGAAAGAGTGATAAAGTACTGGAATGGTCTGCCC
GGGGAGGTGGTGGAGTCACCATCCCTGGATG-
TGTTTAAAAAAAGACTGGATGTGGCACTCGGTGCCATGGTTTAG-TTGAGGTGT-TAGG--GC-----ATGGG-
TTGGA-CTCGATGATC-TTGAAGGTCTCTTCCAAC--CTAG-T
>CR1-L1_Tgu
ACAGTGTCCAGGCAGAAAGGGACCT-
GGGGGTGCTGGTCGACAGCCGGTTGGATATGAGCCAGCAATGTGCCTCGGTGGCCAAGAAGGCCAATGG
CATCCTGGCCTGCATTAGGAATTGTGTGACCAGCAGGAGTAGGGAGGTTATTCTTCCCCTATACTCGGCGC
TGGTGAGACCACATCTTGAGTGCTGTGTCCAGTTCTGGGCCCCTCA--
GTTCAGGAAGGACGTTGAGATGCTTGAGCGCGTCCAGAGGAGGGCAAC--
GAGGCTGGTGAGGGGCTTGGAACACAAGCCCTACGAGGAACGTTTGAGGGAGTTGGGGTTGTTTAGCCTG
GAGAAGAG--GAGGCTTAGAGGTGAC--------CTTATTGCTCTCTACAGCTTCCTGAAGGGA---
GGTTGTAGACAGGTGGGGGTCGATCTCTTCCACCGGGCAGCAACTGGCAGAACAAGAGG-
ACACAGTCTCAAGCTACGTCAGGG---
AAGGTTTAGGTTGGATATTAGGAAAAAAATTTTCACTGAAAGAATAATAAAATACTGGAATTGTCTTCCT
AGGGAGGTGGTGGAATCACCATCTCTGGATA-
TGTTTAAAAAAAGACTGGACTTGGCACTTGGTGCTATAGTCTAG-TTGTGGTGT-TAGG--GC-----ATAGG-
TTGGA-CTTGATGATC-TTAGAGGTCTCTTCCAAC--CTCA-T
>CR1-L2_Tgu
ACAGTGTTCAGGCGGAAAGGGACCT-
GGGGGTGCTGGTCGACAGCCGGTTGAATATGAGCCAGCAGTGTGCCTTGGTGGCCAAGAAGGCCAATGGC
ATCCTGGCCTGCATTAGGAATTGTGTGACCAGCAGGAGCAGGGAGGTCATTCTTCCCCTGTACTCGGCGCT
GGTGAGGCCGCACCTTGAGTGCTGTGTCCAGTTCTGGGCCCCTCA--
GTTTGGGAAGGACGTTGAGATGCTTGAGCGCGTCCAGAGGAGGGCAAC--
GAGGCTGGTGAGGGGCTTGGAACACAAGCCCTGTGAGGAACGTTTGAAGGAGCTGGGGTTGTTTAGCCTG
GAGAAGAG--GAGGCTTAGAGGTGAC--------CTTATTGCTCTCTACAACTTCCTGAAGGGA---
GGTTGTAGACAGGTGGGGGTCGGTCTCTTCCACCGGGCAGCAACTGACAGAACAAGAGG-
ACACAGTCTCAAGCTACGTCAGGG---
AAGGTATAGGTTGGATATTAGGAAAAAAATTTTCACCGAAAGAATAATAAAGTACTGGAATTGTCTTCCC
AGGGAGGTGGTAGAATCACCATCTCTGGATG-
TGTTTAAAAAAAGACTGGACATGGCACTTGGTGCTATAGTCTAG-TTGAGGTGT-TAGG--GC-----ATAGG-
TTGGA-CTTGATGATC-TTAGAGGTCTCTTCCAAC--CTCA-T
>CR1-L3A_Crp
GAAGCAGCGACTCTGAAAAGGACTT-
GGGGGTCACGGTGGATAATCAGCTGAACATGAGCTNCCAGTGCGACGCTGTGGCCAAAAGGGCTAATGC
GATCCTTGGATGCATNAACAGGGGAATCNCGAGCAGGACTAGGGAGGTNATATTNCCTCTCTATTCGGCA
CTGGTGAGACCGCNGCTGGAGTACTGCGTCCAGTTCTGGNNNCCACA--
CTTCAAGAAGGATGTTGANAAATTGGAGAGGGTTCAGAGAAGAGCCAC--
GAGAATGATTAAAGGACTGGAAAACATGCCTTATGGNGAGAGACTGAAGGAGCTCGATCTATTTAGCTTA
TCAAAGAG--AAGGCTGAGGGGTGAC--------TTGATCGCAGTCTATAAGTACCTACACGGG---
GAGCAGAAATCTGGTAATGGAGGGCTCTTCAGTCTAGCGGACAAAGGTATAACAAGANN-
```

CAACGGCCGNAAGTTGAAGCTAGA---
CAAATTCAGGCTAGANATNAGGAGNAACTTTTTNACAGTGAGGGTAGTTAAGCACTGGAACAGNTTACCA
AGGGANGTGGTGGANTCTCCATCNCTGGAGG-
TCTTTAAANCAAGATTGGATGCTTTTCTAGAAGACANGCTCTGGTGGCCTGCGT-TA-G--CA-----GGGGG-
TCGGA-CTAGATGATCTNCNGAGGTCCCTTCCGGC--CCTA--
>CR1-L3B_Crp
AAAGCAGTGACTCTGAAAAGGATTT-
AGGGGTCATAGTGGACAAGCAACTCAACATGAGCTCCCAGTGCGATGCTGTGGCAAAAAGGGCTAATGC
GATCCTTGGATGTATAAACAGGGGAGTAGCGAGTAGGAGCAGGGAGGTGATTTTACCTCTGTATACGGCA
TTGGTGAGACCGATACTGGAATACTGCGTNCAGTTCTGGTGTCCACA--
TTTTAAAAAGGATGTTGAAAAATTGGAGAGGGTGCAGAAAAGAGCCAC--
AAAAATGATTCGAGGGCTGGAGAAAATGCCTTACAGTGAGAGACTTAAAGAGCTCAATCTGTTTAGCTTA
TCAAAAAG--AAGATTGAGAGGTGAC--------TTGATTACGGTGTATAAGTACCTTCACGGG---
GAGAAAATACCGGGTACTAAAGGGCTCTTTAATCTAGCGGAGAAAGGCATAACAAGAAC-
CAATGGCTGGAAGCTGAAGCCAGA---
CAAATTCAAATTAGAAATAAGGCACANATTTTTAACAGTGAGGGTGATTAACCACTGGAACAAACTACCA
AGGGAAGTGGTGGATTCTCCATCTCTTGATG-
TCTTCAGATCAAGACTGGATGCCTTTCTGGAAGATATGCTTTAGTGGCCTGTGN-TATA--CA-----GGAGG-
TCAGA-CTAGATGATC--TAATGGTCCCTTCTGGC--CTTA--
>CR1-X1
AGAGCAGCCCCGCGGAAAGGGATCT-
GGGGGTTCTGGTTGACGGCAAGTTGAATNTGAGCCAGCAGTGTGCCCTGGCAGCCCAAAGGGCCAACCGT
ACCCTGGGGTGCACCAGGCCCAGCACTGCCA-
CCGGGCGAGGGGAGGGGTTGTCCCGCTCTGCTCTGCGCT-
GTGCGGCCTCACCTCCAGCACTGGGTGCAGGTTTGGGTGCCACA--
ATATAAGAAGGACATAAAACTATTAGAGAGCGTCCAAAGGAGGGCTAC--
AAAGATGGTGAAGGGTCTGGAGGGCAAGGTGTGTGAGGAGCGGCTGAGGTCCCTGGGTTTGCTCAGCCC
AGAGCAGAG--GAG-CTGAGGGGAGGC--------CTCATGGCGG-CTGCAGCTCCTCACAGGGA--------
GCGGAGGGGCAGCGCTGAGCTCTGCTCTCTG--TGACAGCGACAGGGCCCGAGG-
GAACGGCATGGAGCTGTGTCAGGG---GAGGGGCAGC-TGGGGGTTAGGGANAGGGTCTGCACCA-
GAGGGCGGTGGGC--ATGGAACAGGCTGCCCAGGGCAGTGGGCACGGCCCCGAG-CTGCCGG-
AGTTCAAGGAGCGTTTGGACAGCGCTCTCAGACATAGGGTTTGAGGTCCTGTGT-GGAG--CC-----AGGAG-
TTGGA-CTCGGTGATCCTTGTGGGTCCCTTCCAAC--TCGGGA
>CR1-X1_Pass
AGAGCAGCGCCGCGGAAAGGGACCT-GGGGGTCCTGGTCGATGGCAAGTTGAACATGAGCCAGCAGTGC-
-
CCTGGCAGCCAGGAGGGCCAACCCTGTCCTGGGGGGCATCAGGCACAGCATCGCCAGCCGGGCAAGGGA
GGGGATTGTCCCGCTCTGCTCTGCGCTGGGGCGGCCTCACCTCGAGTGCTGGGGGCAGTTTTGGGCGCCAC
A--ATATAAGAAAGACATTAAGCTATTAGAGAGCGTCCAAAGGAGGGCAAC--
GAGGATGGTGAAGGGCCTTGAGGGGAAGCCGTATGAGGAGCGGCTGAGGTCACTTGGTCTGTTCAGCCTG
GAGAAGAG--GAGACTGAGGGGAGAC--------CTCATTGCAGTCTACAACTTCCTCGTGAGG---
GGAAGAGGAGGGGCAGGCACTGATCTCTTCTCTGTGGTGACCAGTGACAGGACCCGAGG-
GAATGGCCTGAAGCTGTGTCAGGG---
GAGGTTTAGGTTGGATATCAGGAAAAGGTTCTTCACCCAGAGGGTGGTTGGGCACTGGAACAGGCTCCCC
AGGGAAGTGGTCACAGCACCAAGCCTGACAG-
AGTTCAAGAAGCGTTTGGACAATGCTCTCAGGCACATGGTGTGAGGTCCTGTGC-AGGG--CC-----AGGAG-
TTGGA-CTCGATGATCCTTGTGGGTCCCTTCCAAC--TCAGCA
>CR1-X2
AGAGCAGCCCCGCGGAAAGGGATCT-
GGGGGTTCTGGTTGACAGCAAGTTGAACATGAGTCAGCAGTGTGCCCTGGCAGCCAGAAGGGCCAACCGT
ACCCTGGGGTGCATCAGGCCCAGCACTGCCAGCCGGGCGAGGGAAGGGATTGTCCCGCTCTGCTCTGCGC
TGGTGCGGCCTCACCTCGAGCACTGTGTGCAGTTTTGGGCGCCACA--
ATATAAGAAGGACATAAAACTATTAGAGAGCGTCCAAAGGAGGGCTAC--
GAAGATGGTGAAGGGTCTAGAGGGCAAGACGTATGAGGAGCGGCTGAGGTCCCTTGGTTTGTTCAGCCCA

GAGCAGAG--GAG-CTGAGGGGAGGC--------CTCATGGCGG-CTGCAGCTCCTCACAGGGA--------
GCGGAGGGGCAGCGCTGAGCTCTGCTCTCTG--TGACAGCGACAGGACCCGAGG-
GAACGGCATGGAGCTGCGTCAGGG---GAGGGTCAGG-TGGGGGTTAGGAAAAGGTTCTTCACCA-
GAGGGTGGTGGGC--ATGGAACAGGCTCCCCAGGGCAGTGGTCACGGCCCCGAG-CTGCCGG-
AGTTCAAGAAGCATTTGGACAACGCTCTCAGACATAGGGTTTGAGGTCCTGTGT-GGAG--CC-----AGGAG-
TTGGA-CTCGATGATCCTTGTGGGTCCCTTCCAAC--TCGGGA
>CR1-X2_Pass
AGAGCAGCCCTGCGGAAAGAGATCC-GGGGGTTCGGGTCGATGGCAAGTTGAACATGAGTCAGCAGTGC--
CCTGGCAGCCAGGAGGGCCAACCGTGTCCTGGGGTGCATCAGGCACAGCATCGCCAGCCGGGCGAGGGA
GGGGATTGTCCCGCTCTGCTCTGCACTGGGGCGGCCTCACCTCGAGTNCTGGGTGCAGTTTTGGGCGCCNC
A--ATACAAGAAGGACATCAAACTATTAGAGTGTGTCCAGAGGAGGGCGAC--
CAAGATGGTGAAAGGTCTCGAGGGCAAGACTTANGAGGAGCGGCTGAGGTCACTTGGTTTGTTCAGCTTG
GAGAAGAG--AAGGCTGAGGGGTGAC--------CTCATCGCAGTCTACGNCTTCCTCAAGGGG---
GGCAGCGGAGGGGGAGGTGCTGATCTCCTCTCTCTGGTGACCAGCGATAGGACACGAGG-
AAATGGAATGAAGCTGCGTCAGGG---
GAAGTTCAGATTGGACATTAGGAAAAGGTTCTTCACTGAGAGGGTGGTCGGTCACTGGAACAGGCTCCCC
AGGGAAGTGGTCACGGCACCAAGCCTGTCAG-
AGTTCAAGGAGCGTCTGGACGATGCTCTTAGTCATATGGTTTAGAGTCCTGCGA-GGAG--CA-----GGGAG-
TTGGA-CTCGATGATCCTTATGGGTCCCTTCCAAC--TTGAGA
>CR1-X3_Pass
AGAGCAGCCCTGCAGAAAGGGATCT-
GGGGGTGCTGGTCGGCAGCAGGCTCAGTGTGAGCCAGCAGTGTGCCCTGGCAGCCAAGAGGGCAAACCC
CATCCTGGGGTGCATCAAACACAGCATCACCAGCCGGTCAAAAGAGGTGATTATCCCGCTGTATTCAGCG
TTGGTGCGGCCTCACCTTGAGCGCTGTGTGCAGTTCTGGGCCCCACA--
ATTTAAGAAGGATGTGAAGGTCCTTGAATGCGTCCAGAGGAGGGCAAC--
AAAGCTGGTGAAAGGGCTGGAAGGAATGTCCTGTGAGGAGCGGCTGAGGACTCTGGGCTTGTCTAGTTTG
GAGAAAAG--GAGGCTGAGGGGCGAC--------CTCATTGCTCTCTACAGCTTCCTGAGGAGG---
GGAAGTGGAGAGGGAGGTGCTGANCTCTTCTCCCTGGTATCCAGTGACAGGACGCGTGG-
GAATGGTTCAAAGCTGCGCCAGGG---
GAGGTTCAGACTGGACATTAGGAAGCATTTCTTTACCGAGAGGGTGGTCAAACACTGGAACAGGCTTCCT
AGAGAGGTGGTCGATGCCCCAAGCCTGTCAG-
TGTTTAAGAGGCATTTGGACAATGCCCTTAATAACATGCTTTAAAGCCCTGAAG-TGGT--CA-----GGCAG-
TTGGA-CTAGATGATCGTTGTAGGTCCCTTCCAAC---TGAAA
>CR1-Y
AGAGGAGCTCTGCGGAGAAGGACCT-
GGGGGTCCTGGTGGACGACAGGTTGGCCATGAGCCAGCAGTGTGCCCTGGTGGCCAAGAAGGCCAATGG
GATCCTGGGGTGCATTAAAAGGAGCGTGGCCAGCAGGTCAAGGGAGGTGATCCTCCCCCTCTACTCTGCC
CTGGTCAGGCCTCACCTGGAGTACTGTATCCAGTTCTGGGCTCCCCG--
GTACAAAAAAGACAGGGATCTCCTGGAGAGAGTCCAGCGGAGGGCCAC--
AAAGATGATAAAGGGCCTGGAGCATCTCCCCTATGAGGAAAGGCTGAGCGACCTGGGTCTGTTCAGCCTT
GAGAAAAG--AAGACTCAGAGGGGAT--------CTTATTAATGTTTATAAATATCTTAAGTGT---
GGGAGTCAAAGGGACATGGCCAACCTCTTTTCAGCGGTCTGTGGGGACAGGACAAGGGG-
AAACGGCCATAAACTGGAGCATAG---
GAAGTTCCGCACCAATATGCGAAGGAACTTCTTCACAGTGAGGGTGACGGAGCACTGGAACAGGCTGCCC
AGGGAGGTTGTGGATTCTCCTTCTCTGGAGA-
TATTCAAGACCCGCCTGGACGCCTACCTGTGCAGCCTGCTGTAGGGGCCTGCTT---TG--CA-G---GGGGG-
TTGGA-CTCGATGATCTCTGGAGGTCCCTTCCAGC--CCCTAC
>CR1-Y1_Aves
AAAGCAGCTTNGCAGAAAAGGACCT-
GGGGGTCCTGGTGGACACCAAGCTGAACATGAGCCAGCAATGTGCCCTTGCGGCAAAGAAGGCNAACGG
TATCCTGGGCTGCATTAGGNAGAGCGTTGCCAGCAGGTCGAGGGAGGTGATCCTTCCCCTCTACTCAGCA
CTGGTGAGGCCACACCTGGAGTGCTGTGTCCAGTTCTGGGCTCCCCA--
GTACAAGAGAGACATGGACATACTGGAGAGAGTCCAGCGAAGGGCCAC--
GAAGATGATTAAGGGACTGGAGCATCTCTCCTATGAGGAAAGGCTGAGAGAGCTGGGACTGTTCAGCCTG

GAGAAGAG--AAGGCTCAGGGGGGAT--------CTTATCAATGTGTATAAATACCTGAAGGGA---
GGGTGCAAAGAGGACGGAGCCAGGCTCTTTTCAGTGGTGCCCAGTGACAGGACCAGAGG-
CAATGGGCACAAACTGAAACACAG---
GAGGTTCCCTCTGAACATCAGGAAACACTTTTTCACTGTGAGGGTGACCGAGCACTGGCACAGGTTGCCC
AGAGAGGTTGTGGAGTCTCCATCCTTGGAGA-
TATTCAAAAGCCGTCTGGACACGGTCCTGGGCAACCGGCTCTAGGGCCCTGCTT--GAG--CA-G---GGGGG-
TTGGA-CCAGATGACCTCCAGAGGTCCCTTCCAAC--CTCAGC
>CR1-Y2
AGAGGAGCTCTGCAGAGAGGGACCT-
GGGGGTCCTGGTGGACAACAGGTTGGCCATGAGCCAGCAGTGTGCCCTGGTGGCCAAGAAGGCCAATGG
CATCCTGGGGTGCATTAAAAAGAGCGTGGCCAGCAGGTCAAGGGAGGTGATCCTCCCCCTCTACTCTGCC
CTGGTGAGGCCTCACCTGGAGTACTGTGTCCAGTTCTGGGCTCCCCG--
GTACAAAAAAGACAGGGATCTCCTGGAAAGAGTCCAGCGGAGGGCCAC--
AAAGATGATAAAGGGCCTGGAGCACCTCTCTTATGAGGAAAGGCTGAGCGACCTGGGTCTGTTCAGCCTT
GAGAAAAG--AAGACTGAGAGGGGAT--------CTGATCAATGTCTATAAATATCTAAGGTGC---
GGGAGGCAAAGGGACGAGGCCAGACTCTTTTCAGCGGTGCGTGGCGATAGGACAAGGGG-
AAACGGCCACAAACTGAAGCATAG---
GAAGTTCCGCACAAATGTGCGTAAGAACTTCTTCACAGTAAGGGTGACGGAGCACTGGAACAGGCTGCCC
AGAGAGGTTGTGGAGTCTCCTTCTCTGGAGA-
CGTTCAAGACCCGCCTGGACGCCTACCTGTGCAACCTGGTCTAGGAGCCTGCTT--TGG--CA-G---GGGGG-
TTGGA-CTNGATGATCTCTAGAGGTCCCTTCCAGC--CCCTAC
>CR1-Y2_Aves
AAAGCAGCTTTGCAGAAAAGGACCT-
GGGGGTCCTGGTGGACANCAAGCTGANCATGAGCCAGCAATGCGCCCTTGCGGCAAAGAAGGCCAACAG
CATCCTGGGCTGCATTAGGAAGAGCGTTGCCAGCAGGTCGAGGGAGGTGATCCTTCCCCTCTGCTCAGCA
CTGGTGAGACNACATCTGGAGTGCTGTGTCCAGTTCTGGGCTCCCCA--
GTACAAGAGAGACACGGACATACTGGAGCGAGTCCAGCGAAGGGCCAC--
GAAGATGATTAAGGGACTGGAGCATCTGNCATACGAGGAGAGGCTGAGAGAGCTGGGACTGTTCAGCCT
GGAGAAGAG--AAGGCTCAGGGGAGAT--------CTTATCAATGTGTATAAATACCTGATGGGA---
GGGAGTAAAGAAGACGGAGCCAGACTCTTCTCAGTGGTGCCCAGTGANAGGACAAGAGG-
CAATGGGCACAAACTGAAATACAG---
GAAATTCCATTTAAACATAAGGAAANATNTTTTTACTGTGAGGGTGGTCAAACACTGGAACAGGTTGCCC
AGAGAGGTTGTGGAGTCTCCATCCTTGGAGA-
TATTCAAAACCCGACTGGACACGGCCCTGAGCAACCTGCTCTAGGACCCTGCTT--GAG--CA-G---GGGGG-
TTGGA-CTAGACGATCTCCAGAGGTCCCTTCCAAC--CTCAAC
>CR1-Y3
AGAGGAGCTCTGCGGAGAAGGACCT-
GGGGGTCCTGGTGGACAACAGGTTGGCCATGAGCCAGCAGTGTGCCCTTGTGGCCAAGAAGGCCAATGGT
ATCCTGGGGTGCATTAAAAAGAGCGTGGCCAGCAGGTCGAGGGAGGTGATCCTCCCCCTCTACTCTGCCC
TGGTGAGGCCACATTTAGANTACTGTGTCCAGTTCTGGGCTCCCCA--
GTTCAAAAAAGACAGGGATCTCCTAGAAGGAGTCCAGCGGAGGGCCAC--
AAAGATGATAAAGGGCCTGGAGCATCTCCCGTATGAGGAAAGGCTGAGTAACCTGGGTCTGTTCAGCCTG
GGGAAAAG--AAGACTGAGAGGGGAT--------
CTGATAAATGTTTATAAATATCTAAAGGGAGGTGGGAGGCAAATGGATGAGGCCAGGCTCTTCTCGGTGG
TGTGTAGCGATAGGACAAGGAG-TAATGGCCTAAAACTTGAACATAG---
GAAGTTCCGTACTAACATGCGGAAGAACTTCTTTACGGTAAGGGTGACGGAGCACTGGAACAGGTTGCCC
AGAGAGGTTGTGGAGTCTCCTTCTATGGAGA-
TATTCAAGACCCGTCTGGACGCCTACCTGTGCGACCTATTGTAGGTACCTGCTT--TAG--CA-G---GGGGG-
TTGGA-CTCGATGATCTCTTGAGGTCCCTTCCAAC--CCCTGC
>CR1-Y4
AGAGGAGCTCTGCAGAGAAGGACCT-
GGGTGTCCTGGTGGACAACAGGTTGGCCATGAGCCAGCAGTGTGCCCTTGTGGCCAAGAAGGCCAATGGT
ATCCTGGGGTGCATTAAAAAGAGCGTGGCCAGCAGGTCGAGGGAGGTGATCCTCCCCCTCTACTCTGCCC
TGGTGAGGCCACATCTGGAGTACTGTGTCCAGTTCTGGGCTCCCCA--

GTTCAAGAAAGACAGGGANCTCCTAGAGAGAGTCCAGCGGAGGGCCAC--
AAAGATGATGAGGGGCCTGGAGCATCTCCTNTATGAGGAAAGGCTGAGAGACCTGGGACTGTTCAGCCTG
GAGAAGAG--AAGACTGAGAGGGGAT--------CTCATCAATGCTTATAAATATCTAAAGGGA--
TGGGAGTCAAGTGGATGGGGCCAGGCTCTTTTCAGTGGTGCGCAGCGACAGGACAAGGGG-
CAATGGGCANAAACTGGAACACAG---
GAAGTTCCATACGAACATGAGGAAGAACTTCTTTACTGTGAGGGTGACGGAGCACTGGAACAGGCTGCCC
AGAGAGGTTGTGGAGTCTCCTTCTCTGGAGA-
TATTCAAGACCCGCCTGGACGCCTTCCTGTGCGACCTGCTGTAGGAACCTGCTT--TAG--CA-G---GGGGG-
TTGGA-CTCGATGATCTCTAGAGGTCCCTTCCAAC--CCCTGC
>CR1-YB1_Tgu
AAAGCAGCTCTGCGGGGAAGGACCT-
GGGGGTCCTGGTGGACAACGAGCTGTCCATGAGCCAGCAGTGTGCCCTGGGGGCCAAGAAGGCCAATGG
GATCCTGGGGTGCACCGGGAAGAGCATTGCCAGCAGGTC-
AGGGAGGTGATCCTGCCCCTCTGCTCAGCCCTG--
TGAGGCACATCTGGAGTGCTGTGTCCAGCTCTGGGCTCCTCA--
GGACAGGAGGGACACGGAGCTCCTGGAGCGGGGCCAGCGGAG-GCTGC--
GGAGATGATGAAGGGCCTGGAGCATCTCCCTGNCGAGGAAAGGCTGAGGGAGCTGGGGCTGCTCAGCCT
CGAGAGGAGCCCCAGCTGAGAGGGGCC--------CTCAGCCCTGGGTGTCCCTGTCTGCAGGGA---
GGGCTCAGAGCA---GGGCCCAGGCTCTGCTCCGGGG-GCCCAGCAATGGCACCAGA-G-
GAACGGGCAGGGACTG-AGCCCAG---GAAGTTCCACCTGGACATGAGGCAGAACTTCTTTCCTGTGC-
AGTGACCGAGCACTGAGACAGATTGTCCAGAGAGGGTGTGGAGTCTCCCTCACTGGGGA-
TATTCCAGAACCGTCTGGACACAATCCTGTGCCCTGTGCTCTGGGGCCCTGCTG--GAG--CA-G---GGAGG-
TGGGA-CCAGATGACCCACTGTGGTCCCTTCCAGC--CTGACC
>CR1-YB2_Pass
AAAGCAGCTCTGCGGAGAAGGACCT-
GGGGGTCCTGGTGGACAACAAGCTGTCCATGAGCCAGCAGTGTGCCCTTGTGGCCAAGAAGGCCAATGGT
ATCCTGGGGTGCATTAGGAAGAGCATTGCCAGCAGGTCGAGGGAGGTGATCCTGCCCCTCTACTCAGCCC
TGGTGAGGCCACATCTGGAGTGCTGTGTCCAGTTCTGGGCTCCTCA--
GNACAAGAGAGACATGGAGCTCCTGGAGCGGGTCCAGCGGAGGGCNAC--
AAAGATGATTAAGGGACTGGAGCATCTCTCTTACGAGGAAAGGCTGAGGGAGCTGGGCCTGTTCAGCCTC
GAGAAGAG--ACGACTGAGAGGGGAC--------CTCATCAATGTCTGTNAGTATCTGAAGGGA---
GGGTGTCAAGAGGATGGAGCCAGGCTCTTCTCGGTGGTGCCGAGCAATAGGACAAGAGG-
CAACGGGCAGAAACTGATGCACAG---
GAAGTTCCACCTGAACATGAGGAAGAACTTCTTTACTGTGCGGGTGACCGAGCACTGGAACAGATTGCCC
AGAGAGGTTGTGGAGTCTCCCTCACTGGAGA-
TATTCAAGAACCGTCTGGACGCAATCCTGTGCCATGTGCTCTAGGACCCTGCTT--GAG--CA-G---GGAGG-
TTGGA-CCAGATGACCCACTGTGGTCCCTTCCAAC--CTGACC
>CR1-Y_Pass
AGAGCAGCTCTGCGGAGAAGGACCT-
GGGAGTCTTGGTGGATGACAAGCTGACCATGAGCCGGCAGTGTGCCCTTGCGGCCAGGAGGGCCAGTGGT
ATCCTGGGGTGCATCGGGAAGAGTGTGGCCAGCAGGTCGAGGGAGGTGATCCTGCCCCTCTACTCGGCCC
TGGTGAGGCCACATCTGGAGTGCTGTGTCCAGTTCTGGGCTCCTCA--
GTACAAGAAAGACAAGGAGCTACTGGAGAGGGTCCAGCGGAGGGCCAC--
AAAGATGATNAGGGGTCTGGAGCATCTCTCTTATGAGGAGAGACTGCGGGAGCTGGGCCTGTTTAGTCTG
GAGAAGAG--AAGACTGAGAGGGGAT--------CTCATCAATGCATATAAATATCTCAAAGGC---
GGGTGCCAAGAGGATGGTGCCAGACTCTTTTCAGTGGTGCCCAGCGACAGGACGAGGAG-
CAATGGCCATAAACTAAAACACAA---
GAAGTTCCACCTCAACATGAGGAAGAACTTCTTTACATTGAGGGTGGCAGAGCACTGGAACAGGCTGCCC
AGGGAGGTCGTGGAGTCTCCCTCTCTGGAGA-
CATTCAAAACCCACCTGGACGCGTTCCTGTGTNACCTGCTCTAGGACCCTGCCT--TGG--CA-G---GGGGG-
TTGGA-CTAGATGATCTCCAGAGGTCCCTTCCAAC--CCTAAC
>CR1-Z1_Pass
GGAGCAGCTCTGTGGAAAGGGACCT-
GGGGGTCCTGGTGGACAACAAGCTCAATATGAGTGAACAGTGTGCTGCTGCGGCAAAGAAAGCCAACAG

GATGCTGGGCTGCATCAACAAGGGCATCACCAGCAGAGATAAAGAAGTCATTATCCCACTCTACTCAGCG
CTTGTCAGGCCACACCTGGAATACTGTGTTCAGTTTTGGTCCCCGCT--
ATACAAAAAAGATGTGGACAGGCTGGAGAGGGTCCAGAGAAGGGCCAC--
AAAGATGATCAAAGGACTGGGAAGCCTGCCATGTGAGGAAAGGCTGAGAGAACTGGGTTTGTTCAGCCTT
GAGAAAAG--AAGGCTTAGGGGAGAC--------CTTATCACCATGTTCCAGTATTTAAAGGGT---
GGCTACAAAGAAGATGGAGACTCCCTTTTTACAAGGAGTCACATGGAAAAGACGAGGGG-
TAATGGGTACAAGTTACTCCTGGG---
GAGATTCCGATTGGACACAAGAGGAAAATTTTTCACAATGAGAACAATCAGCCATTGGAATAATCTCCCC
AGGGAAGTGGTGGATTCCCCAACGTTGGACA-
CTTTTAAGATTCGGCTGGACAGGGTGCTGGGCCATCTTGTCTAG-ACCGTGCTT-TTGC--CAAG---AAAGG-
TTGGA-CCAGATGATC-CTTGAGGTCCCTTCCAAC--CTGG--
>CR1-Z2_Pass
GGAGCAGCCTTGCTGAAAGGGACCT-
GGGGGTCCTGGTGGACAACAAGCTGANCATGAGTCAGCAGNGCGCCGCTGCAGCAACGAAGGCAAATCG
GATCCTGGGCTGCATCCGCAGGGGCATTACTAGCAGAGATAGAGACGTGATCATCCCACTCTACTCAGCG
CTTGTCAGGCCGCACCTGGAGTACTGTGTCCAGTTCTGGTCCCCACA--
ATTCAAGAAAGACGCGGACAGACTGGAGAGGGTCCAAAGGAGGGCCAC--
GAAGATGATCAAAGGGCTGGAGAACCTGCCCTGTGAGGAAAGACTGAAGGAGTTAGGTCTTTTCTCCCTG
GAGAAGAG--AAGGCTTAGGGGGGAC--------CTCATCACAGTATTCCAGTACTTAAAGGGC---
GGCTACAAAGAGGACGGAGGCTCTCTCTTCACAAGGAGCCACATGGAGAAGACAAGGGG-
CAACGGGTACAAGTTGCACCGGGA---
GAGGTTTCATCTCGATATAAGAAAGAAATTTTTTACAGTGAGAACAATCANTCACTGGAACAACCTCCCC
AGGGATGTGGTAGAGTCCCCATCACTGGAGG-
TTTTCAAGACGCGATTGGACAGGGTGCTAGATAATCTCATCTAG---GCTCCCT-TTCC--CACG---AAAGG-
TTGGA-CCAGATGATCTTTCGAGGTCCCTTCCAAC--CTGGGC
>CR1_1a_XT
GGGTATCCTTAATGGAGAAGGATCT-
AGGGGTTTTTGTAGATNACAAGTTGTCTAATTCCAGGCAGTGTCATTCTGTGGCTACTAAAGCAAATAAAG
TGCTGTCTTGTATAAAAAAGGGCATTGACTCAAGGGATGAGAACATAATTTTGCCTCTTTATAGGTCCCTG
GTAAGGCCTCACCTTGAGTATGCAGTGCAGTTTTGGGCTCCAGT--
CCTTAAGAAGGATATTAATGAGCTGGAGAGAGTGCAGAGACGTGCAAC--
TAAACTGGTAAAGGGGATGGAAGATTTAAGCTATGAGGTTAGACTGTCGAGGTTGGGGTTGTTTTCTCTG
GAAAAGAG--GCGCTTGCGAGGGGAC--------ATGATTACTCTGTACAAGTACATTAGAGGG---
GATTATAGGCAGNTGGGGGATGTTCTTTTTTCCCATAAAAACAATCAGCGCACCAGAGG-
TCACCCCTNTAGATTAGAGGAACG---
GAGCTTCCATTTGAAGCAGCGTAGGTGGTTTTTCACGGTGAGGGCAGTGAGGTTGTGGAATGCCCTTCCTA
GTGATGTGGTAATGGCAGACTCTGTTAATG-
CCTTTAAGAGGGGCCTGGATGAGTTTTTGTATGTGAGTGTATAGTGTGCTGGGTTTACT--CG-G---ATGGG-
TTGAA-CTTGATGGAC--NATGGTCTTTTTTCAAC--CCTATG
>CR1_1b_Xt
GGGTNTCCTTAATGGAGAAGGATCT-
AGGGGTTTTTGTTGATAACAAGTTGTCTAATTCCAGGCAGTGTCATTCTGTGGCTACTAAAGCAAATAAAG
TGCTGTCTTGTATAAAAAAGGGCATTGACTCAAGGGATGAGAACATAATTTTGCCCCTTTATAGGTCCCTG
GTAAGGCCTCACCTTGAGTATGCAGTGCAGTTTTGGGCTCCAGT--
CCTTAAGAAGGATATTAATGAGCTGGAGAGAGTGCAGAGACGTGCAAC--
TAAACTGGTTAAGGGGATGGAAGATTTAAACTATGAGGTGAGACTGTCGAGGTTGGGGTTGTTTTCTCTG
GAAAAGAG--GCGCTTGCGAGGGGAC--------ATGATTACTCTGTACAAGTACATTAGAGGG---
GATTATAGGCAGATGGGGGATGTTCTTTTTTCCCATAAAAACAATCAACGCACCAGAGG-
TCACCCCTTTAGATTAGAGGAACG---
GAGCTTCCATTTGAAGCAGCGTAGGTGGTTTTTCACGGTGAGGGCAGTGAGGTTGTGGAATGCCCTTCCTA
GNGATGTGGTAATGGCAGATTCTGTTAATG-
CCTTTAAGAGGGGCCTGGATGAGTTCTTGTATGTGAGTGTATAGTGTGCTGGGTTTACT--TG-G---ATGGG-
TTGAA-CTTGATGGAC--ACTGGTCTTTTTTCAAC--CCTATG
>CR1_AC_1

ACAGCAGTGTGTGCGAAAAAGACCT-
TGGAGTCCTCGTGGACAACAAGTTAAACATGAGCCAACAATGTGATGCGGCTGCTAAAAAAGCCAATGG
GATTCTGGCCTGCATCAATAGGGGAATAGCGTCTAGATCCAGGGAAGTTATGCTCCCCCTCTATTCTGCCT
TGGTCAGACCACACCTGGAATACTGTGTCCAATTTTGGGCACCACA--
GTTGAAGGGAGATGTTGACAAGCTGGAAAGCGTCCAGAGGAGGGCGAC--
TAAAATGATTAAGGGTCTGGAGAACAAGCCCTATGAGGAGCGGCTTAAAGAGCTGGGCATGTTTAGCCTG
CAGAAGAG--AAGGCTGAGAGGAGAC--------ATGATAGCCATGTACAAATATGTGAAGGGA---
AGTCATAGGGAAGAGGGAGCAAGCTTGTTTTCTGCTGCCCTGCAGACTAGGACACGGAA-
CAATGGCTTCAAACTACAGGAAAG---
GAGATTCCACCTGAACATCAGGAAGAACTTCCTCACTGTGAGAGCTGTTCGACAGTGGAACTCTCTCCCC
GGGGCYGTGGTGGAGGCTCCTTCCTTGGAGG-
CTTTTAAGCAGAGGCTGGATGGCCATCTGTCGGGGGTGCTTTGATTTCCTGCTTCTTAG--CA-----GGGGG-
TTGGA-CTAGATGGCC-CATGTGGTCTCTTCCAAC--TCTACT
>CR1_Mam
CAGTTACGACCCAGGAAAGGGATCT-AGGAGTCATTGTAGACTATTCCCTGAAGACATCAGCCCAATGT---
GCTGTAGCCAAAAAGGCCAACAAAATNCTGGGCATCATCAGGAAGGGNATTGGAAACAAAACAGAAAAC
ATTATCCTACCTTTGTGCAAAACCATAGTATATCCACACCTGGAATACTGTGTGCAGTTCTGGTTGCCGCA
NCCCTCAAGAAAGNCATANCAGAGCTGAGAAGGGTAAT--------------TAAAATGATCAAGGAGATGGA-
AGGCTTTNATAAGAGGCTGAGCTGNTTTGTTTAAAA--CTTTAGTCTGGAAAGACG--
AAGGCTGAGAGGGGAT--------ATGATCAAAGTCTATAAAACCATGAAGGGT---
ATGGATAAGGTGAACATAGACTT---GTTCACCAAATCCCAGAATACTAGAACTAGG---
GCAGCCTTTGAAGCTTGAAAGAAATTTTAGGGACAAATNAAANAAAAGGTGTAGACTCATTNTACAGNG
GG--

>L3
GCAGCAGTTCACGTGAAAAAGACCT-
AGGGGTTTTAGTTGACCACAAGCTCAATATGAGCCAACAGTGTGATGTGGCTGCCAAAAAAGCTAATGCA
ATCTTAGGCTGCATTAATAGAAGTATAGTGTCCAGAWCAAGGGAGGTAATAGTCCCGCTGTACTCTGCGC
TGGTCAGACCACATCTGGAGTATTGCGTTCAGTTCTGGGCACCACA--
TTTTAAGAGGGACATTGACAAACTGGAGCGCGTCCAGAGAAGAGTGAC--
CAGGATGGTGAAGGGTCTGGAAACCATGTCATATGAGGAACGGTTGAAGGAACTGGGGATGTTTAGCCTG
GAGAAGAG--AAGACTTAGAGGAGAC--------ATGATAGCTGTCTTCAAATATTTGAAGGGC---
TGTCATGTGGAAGAGGGATTAGACTTATTCTGTGTGGCTCCAGAGGGCAGAACTAGGAT-
CAGTGGGTGGAAGTTACAGGGAGG---
CAGATTTCAGCTCAATATAAGGAAGAACTTTCTAACAATCAGAGCTGTCCAAAAATGGAATGGGCTGCCT
TGGGAGGTAGTGAGCTCCCTGTCACTGGAGG-
TATTCAAGCAGAGGCTGGATGACCACTTGTCAGGGATGTTGTAGATTCCTGCAT-TGGA--TG-----GGAGN-
TTGGA-CTAGATGACC-TCTAAGGTCCCTTCCAAC--TCTGAG
>L3b

AAAAGGATGTGGAGAANTTGGAGAGAGTTCAGAGGAGAGCNAC--
AAAGATGATTAAAGGGTTGGAAAATGGACCTATGAGGAAAGGTTAAAGGAACTGGGATTATTCAGCCT
GGAGAAGAG--AAGGCTGAGGGGNGAC--------TTAATAACAGTCTTCAAGTATATGAAGAGT---
TATTACATAGAGGATGGTGACCAGCTGTTCTCCATCTCCACTGAGGACAGAACAAGAGG-
AAATGGGCTTAAATTGCAGCATGA---
GGGATTTAGGTTAGATATAAGGAAGAATTTCCTGACAGTGAGAGCTGTTAAATACTGGAATGGGTTGCCG
AGGGAGATTGTGGAATTTCCTTCTCTGGAGAGNCTTTAAAAATAGAATGNATNCTCATCTGTCTGGGATG
NTTTA-AATCCTGCCCAAAGN--TNGGNGNANGGA-TTGGA-CTAGATGACCTCT-GAGGTCCTTTCCAAT--
TCTGAG
>L3_ME
ACAGCAGTTTGTCTGAAAAAGATCT-
GGGGGTTTTAGTGGACTGCAAGCTCAATATGAGTCAGCAGTGTGATATGGCAGCCAAAAAAGCTAATGTA
ATCTTGGGCTGCATTAAGAGAGGCATAGCTTCCAGGAATAAGGAGGTGATAGTCCCACTGTACTCTGCCC

TGGTCAGACCACATCTGGAGTATTGTGTTCAGTTCTGGGTACCACA--
TTTTAGGAAGGACATTGATAAGCTGGAGAGTATCCAGAGGAGGGCAAC--
CAGGATGGTGAAGGGCCTTGAGTTCATGCCATATGAGGATCAGTTGAAGGAACTGGGGATGTTTAGCCTG
GAGAAGAG--AAGACTCAGGGGGGAC--------ATGATAGCTGTCTTCAAGTATTTGAAGGGC---
TGTCATGTGGAAGAGGGATTAGACTTGTTCTGTTTGGCCCCAGAGGGCAGAACTAGGAG-
CAATGGGTGGAAGTTGCAAAGAGG---CAAATTTAGGCTTGATGTAAGGAAAAACTTCCT---
AATTAGAGCTGTCCAAAAGTGGAATGGACTGCCTTAGAAGGTGGTGGGTTCCCTCTCACTGGAGG-
TCTTCAAGCAGAGGCTGGATGACCACTTGTCAGATATGTTRTAGATTCCTTT---------TGAG-GTATGGA-
TTGGA-CTAGATGGYC-ACTGAGGTTCCTTCCAAC--TCTAAA
>PlatCR1
CAGTCACGACTCAGGAAAGAGATCT-
CGGAGTCATTGTTGACTGTTCTTTTAAATCATCGGTCCAATGTGCGGCAGCAGCCAAAAAGGCCAACCGA
ATGCTGGGCATCATCAGGAAGGGGATAGAAAACGAAACAAAAGGCATAGTTTTGCCACTATATAAAACC
ACGGTACGCCCGCACCTGGAATACCGCGTGCAGTTCTGGTCGCCGCA--
TCTTAGGAAGGACATAATAGAGCTGGAGAAGGTACAGAGAAGGGCAAC--
CAAGATGATCGGGGGGATGGAGAAGCTTCCGTACGAGGATAGACTGAAAAGATTAGGACTCTTCAGTCTG
GAAAGACG--AAGGCTGAGAGGGGAC--------ATGATTGAAGTCTACAAAATCATGAAGGGT---
GTGGACAGGGCGAACACGGAATTGTTGTTCACCAAATCCCACAACACCAGGACGAGGGG-
GCACCCACTGAAGCTTGAAGGTGG---
TAGGTTCAAAACAAACAAAAGGAAACACTTCTTCACACAGCGGGTGGTAAGCATATGGAATTCGTTACCA
CAGGAAGTTGTGCAGGCCGAAAATATCAGTA-
GGTTCAAGAGGGGTTTGGATAAATTCATGAGAGGGAAAGTTAGGGCCACTGTCG-GAGA--CA-----
GAATA-CTGGG-CTGGATGGAC--CATTGGTCTGACCCAGT--AATGGC
>PlatCR1_old1

GTCGGAGAGGCGGAGAAGCTTCCGNACGAGGACGGACCGAAAGGGTCGGGACTCTGCGGNCCGGAGAG
ACG--CGGGCCGAGAGGGGAC--------AGGACTGGAGTTTACGGAACCGCGAAGGGG---
GCGGACAGGGCGAACACGGAATTGCCGTTCCCCAAATCCCGCGCCACCAGGACGAGGGG-
GCACCCGCTGGAGCTGGAGGGCGG---
CAGGTTCAAAACAAACGNAAGGAAACACTTCTTCNCCCAGCGGGGGGTGAGCACGTGGAATCCGTTCCCA
CAGGGAGCCGTGCA-GCCGGAAACACCGGCA-
GGTTCGAGAGGGGTTTGGATAGATCCATGGGAGGGAGAGTCAGGGCCGCTGTCG-GAGA--CA-----
GAGTC-CTGGG-CCGGATGGGC--CGCGGGGCTGACCCGGG--AATGGC
>PlatCR1_old2

ATCGGGGGAATGGAGAAGCTTCCGTAGGAGGAGAGACCGAAAGGATTAGGACTCTTCAGTCTGGAAAGA
CG--CAGGCCGAGAGGGGAC--------GGGATTGAAGTTTACAGAATCATGAAGGGN---
GCGGGCAGGGCGAACACGGAATTGTCGTTCNCCAAATCCCACGCCACCGGGACGAGGGG-
GCACCCACTGAAGCTTGAAGGTGG---
CAGGTTCAAAACAAACGAAAGGAAACGCTTCTTCACCCAGCGGGCGGTAAGCGTATGGAATTCGTTNCCA
CGGGAAGTTGTGCAGGCCGAAAATATCAGCA-GGTTCAAGAGGGGTTTGGATAGATTCGCGAGAGGG-----
----GCCGCCGTCG-GAGA--CA-----GAATC-CTGGG-CCGGACGGAC--CGCGGGTCCGACCCAGT--AATGGC
>Plat_L3
GCACAAGTACAGCAGAAAAAGACCT-
GGGGGTCATAGTCGACCANAAGCTGAATATGAGTCAGCAATGTAGTGCCGTTATTAAAAAAGCCAACGCG
GTACTGGGATGTATTAACAGGAGTATGACATGCAAGAGTCGGGAAGTAATCCTTCCGCTATACTCTGCAC
TGGTCAGACCCTTATTAGAGTACCGTGTCCAGTTTTGGTCACCGCA--
CTTTAAAAAGGATGTGGAGAAACTGGAGAGGGTCCAGAGAAGAGCGAC--
AAAAATGATTAAAGGGATGGAAAATAGGTCCTATGAGGAAAGGTTAAAGGAATTGGGGTTGTTTAGCCT
GGAGAAGAG--AAGGCTAAGGGGTGAC--------TTAATAACTGTCTTCAAGTATATGAAGGGT---

TTTTATGGGGAGGACGCTGACCAGTTGTTCTCCGTGTCCACNGAGGACCGAACAAGAGG-
AAATGGGCTTAAATTAAAGCAGGA---
GAGATTTCGGTTGGACATAAGGAAGAACTTCTTGACCGTCGGGGTGATAAAACACTGGAACGGGCTACCG
AGGGAGGTTGTGGAGTCTCCATCCCTGGAGA-
TCTTTAAGAAAAGAATAGACGNCCATCTGTCCTGGATGGTTTAGTCACCTGCCTGGAGG--CA-----GGGGG-
CTGGA-CCAGATGACCTCTCGAGGTCCCTTCCAGC--TCTANG
>Plat_L3b
GCGCGAGTACGGCAGAAAAAGATCT-
AGGGGTCATAGTGGACCACAAGCTGAACGCGAGTCAGCAATGTAGCGCCGTTGTTAAAAAAGCNAACGC
GATACCGGGATGCATNAATAGGAGTATAGCATGTAGGAACCGCGAGGTAATCCTCCCGTTATACTCAGCG
CTGGTCAGACCCTTACTAGAGTACTGCGTCCAGTTCTGGGCTCCGCA--
ACTTAAGAGGGACGTGGAGAACTTGGAGAGGGTTCAGAGGAGAGCCAC--
GAAGATGATTAAAGGGTTGGAAAATAAGACCTATGAGGAAAGGTTAAAGGAACTGGGATTATTTAGCCT
GGAGAAGAG--AAGGCTGAGGGGCGAC--------TTAATAACGGTCTTCAAGTATATGAAGGGT---
TATTATACGGAGGACGGTGACCAGCTGTTCTCCATCTCCACTGAGGACAGAACAAGAGG-
AAATGGGCTTAAACTGCAGCANGA---
GGGATTTAGGTTAGACATAAGGAAGAACTTCCCGACAGCGAGGGTTGTTAAACACTGGAACGGGTTACCG
AGGGAGGTTGTGGAATCTCCTTCCCTGGAGA-
TCTTTAAAAACAGGATAGATNCTCATCTGTCTGGGATGGTTTAGGGTCCTGCCTGGAGG--CA-----GGGGG-
ATGGA-CTAGATGACCTCTCGAGGTCCCTTCCAGC--CCTANG
>PSLINE
GCAGCAGTTCTGCAGAAAAGGATCT-
GGGGGTTACAGTGGATAAGAAGCTGGATATGAGTCAACAGTGTGCCCTGGTTGCCAAGAAGGCTAATGGC
ATATTGGGCTGCATTAGTAAGAGCATTGCCAGCAGATCGAGGGAAGTGATTATTYCCCTSTATTCGGCATT
GGTGAGGCCGCATTTGGAGTATTGTGTCCAGTTTTGGGCCCCCCA--
CTACAAAAAGGATGTGGAMAAGTTGGAGAGAGTCCAGCGGAGGGCAAC--
AAAAATGATWAGGGGGGCTGGARCACAAGACTTATGAGGAGAGGCTGAAGGAACTRGGCCTGTTTAGTCT
GCAGAAGAG--AAGAATGAGGGGGGAT--------TTGATAGCAGCCTTTAACTACCTGAAAGGG---
GGTTCCAGGGAGGATGGAGCTAGGCTGTTYCAGTGGTGGCAGATGACAGAACAAGGAG-
CAATGGTCTCAAGTTGCAGTGGGG---
GAGGTCTAGGTTGGACATTAGGAAAAAGTTTTTCACTAGGAGGGTGGTGAAGCATTGGAATGGGTTACCT
AGGGAGGTGGTGGCATCTCCATCTTTAGAGG-
TTTTTAAGCTGCGGCTTGACAAAACCCTCGCTGGGATGATTTAGRGTCCTGCTT-TGAG--CA-----GGGGG-
TTGGA-CTAGATGACCTCCTGAGGTCCCTTCCAAC--TCTAAT
